# Whole Genome Analysis and Targeted Drug Discovery Using Computational Methods and High Throughput Screening Tools for Emerged Novel Coronavirus (2019-nCoV)

**Published:** 2020-03-30

**Authors:** Hemanth Kumar Manikyam, Sunil K Joshi

**Affiliations:** *1 Faculty of Science, North East Frontier Technical University, Arunachal Pradesh, India; *2 Assistant Professor, Department of Paediatrics, University of Miami Miller School of Medicine, Miami, FL, USA.

**Keywords:** SARS-CoV-2, Coronavirus, Antiviral drugs, Docking tools, Ligands, Protease inhibitors, Polymerase inhibitors, BLASTn, SMART BLAST and WebDSV 2.0

## Abstract

A novel coronavirus designated as SARS-CoV-2 in February 2020 by World Health organization (WHO) was identified as main cause of SARS like pneumonia cases in Wuhan city in Hubei Province of China at the end of 2019. This been recently declared as Global Pandemic by WHO. There is a global emergency to identify potential drugs to treat the SARS-CoV-2. Currently, there is no specific treatment against the new virus. There is a urgency to identifying potential antiviral agents to combat the disease is urgently needed. An effective and quick approach is to test existing antiviral drugs against. Whole genome analysis and alignment carried out using BLASTn, SMART BLAST and WebDSV 2.0 had shown more than 238 ORF’s coding for proteins mostly origin from Bat SARS coronavirus and root genomic origin from Archaea. Molecular docking results against protein targets Furin, papain like proteases, RdRp and Spike glycoprotein had shown paritaprevir, ritonavir, entecavir and chloroquine derivatives are the best drugs to inhibit multi targets of coronavirus infection including natural compounds corosolic acid, baicalin and glycyrrhizic acid with minimal inhibitory concentrations. Thus we propose use of paritaprevir, entecavir, ritonavir and chloroquine derivatives as best drug combination along with niacinamide, folic acid and zinc supplements to treat novel coronavirus infection. We also propose use of plant protease inhibitors (PI’s) and Anti-IL8, IL-6, IL-2 as future drug models against coronavirus.

## INTRODUCTION

A novel coronavirus designated as SARS-CoV-2 in February 2020 by World Health organization (WHO) was identified as main cause of SARS like pneumonia cases in Wuhan city in Hubei Province of China at the end of 2019. This been recently declared as Global Pandemic by WHO. There is a global emergency to identify potential drugs to treat the SARS-CoV-2. Currently, there is no specific treatment against the new virus. There is a urgency to identifying potential antiviral agents to combat the disease is urgently needed. An effective and quick approach is to test existing antiviral drugs against SARS-CoV-2. Spike protein recognize and bind host receptors like ACE-2 and whose conformational changes facilitates fusion of viral envelop and host membrane leading viral entry into host cells. Replication of viral RNA occurs through RNA polymerase activity by n unique mechanism. Targeting protease like Spike protein for viral entry and polymerase for replication of virus in host cell can bring effective treatment against novel SARS-CoV-2.

**Figure 1.**
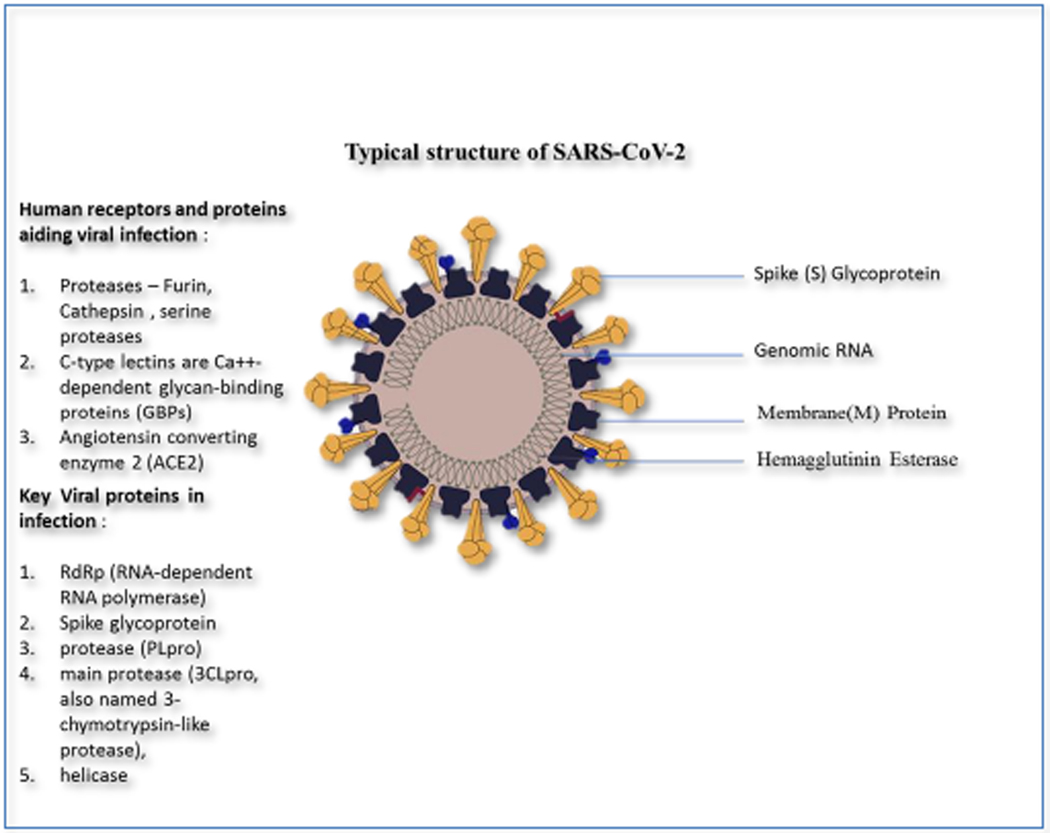
SARS-CoV-2 proposed viral proteins and human cell proteins aiding virus host cell entry and replication.

Coronavirus are enveloped with a positive RNA genome. Coronaviridae family of the order Nidovirales, having four genera (α, β, γ and δ). sThe SARS-CoV-2 seems be β genus and probable origin from bat and suspected to have an intermittent host. Structurally coronavirus contain spike (S) protein, envelope (E) protein, membrane (M) protein and nucleocapsid (N) protein. Viral entry through host receptor attachment promoted by spike protein leading to viral fusion to cell membrane of the host and leading to infection. Incubation period may range from 7 days to 21 days with flu like symptoms or sometimes go asymptomatic. Spike protein determines the viral entry and infection. Antiviral therapies targeting human immune system and direct coronavirus are the primary methods of treating the viral infection. Innate immunity of human immune system plays important role as primary defence mechanism against coronavirus infection and its replication. Interferon plays key role in controlling viral replication and immune presentation of viral antigens and to enhance immune responses. Viral entry and replication require human cell signal pathways, by blocking such signal pathways can bring anti-viral effect. Previously known coronavirus infections SARS and MERS causing virus used angiotensin converting enzyme 2 (ACE2) and DPP4 human receptors of human cells independently. Targeting RNA-dependent RNA polymerase (RdRP) of coronavirus is second line of treatment itself include preventing the synthesis of viral RNA through acting on the genetic material of the virus inhibiting virus replication. Activation of the viral spike protein (S) by host cell proteases is essential for viral host cell attachment and entry and the responsible enzymes are potential therapeutic targets. The cellular proteases like furin, cathepsin and receptors like C-type lectins are Ca++-dependent glycan-binding proteins (GBPs) a functional receptor-mediated endocytosis in Golgi bodies plays important role viral infection, replication and maturation as shown in (Figure 1).

Different strategies for developing drugs and treatment against SARS-CoV-2 include viral protein inhibitors and human cell receptor inhibitors to be studied extensively. Some interferon inhibition like ribavirin and cyclophilin were studied to treat coronavirus pneumonia. Interferon inhibition alone cannot treat the SARS-CoV-2, multi target therapy to be considered as effective way of treating which includes inhibition of receptor proteases like furins, viral proteins like spike (S) and Nsp12, a coronavirus, is an RNA-dependent RNA polymerase (RdRp) protein vital enzyme for coronavirus replication/transcription complex, which can inhibit both viral host cell entry and replication. As designing of novel molecules at present is time consuming and no present therapies existing to treat SARS-CoV-2, we propose use of existing antiviral and other drugs to treat the coronavirus infection. High-throughput screening, bioinformatics and AI based tools and methods to screen existing drug database is the fastest approach to discover drug leads against SARS-CoV-2 for example anti-retro viral drugs like Lopinavir and Ritonavir.

After determining the efficacy, the drugs can be approved through proper hospital based clinical trials for clinical treatment of patients. Viral encoding proteins and human cell proteins aiding viral host cell entry and replication were analysed by bioinformatics tools like Molecular docking and Swiss Dock protocols by conducting homology modelling and ligand preparations. SARS-CoV-2 Viral papain like protease, main protease, spike and RNA-dependent RNApolymerase (RdRp) and human furin human ACE2 and type-II transmembrane serine protease proteins were extensively used for targeted drug discovery. Virtual screening of proposed protein targets was docked against anti-HIV and anti-Hepatitis drugs were selected as ligands from drug database including some natural phytochemicals known for antiviral properties. The present study predicts wide range of drug leads that may inhibit this study predicts a variety of compounds that may inhibit novel SARS-CoV-2 coronavirus. Validation of successful drug leads should be studies for complete efficacy using proper in-vitro and in-vivo methods further to continue clinical studies.

## METHODS

### Methods & materials

#### Homology genome blast and genomes information

Whole genome of SARS-CoV-2 was obtained from NBCO Nucleotide database with reference number NC_045512.2. The nucleotide sequences were aligned using BLASTn sequence aligner and similarity search analysis with SARS-CoV-2 viral genomes submitted at NCBI from different samples of infected Cluster. MN908947 (complete genome) NC_045512 (reference sequence), LC522350 (gene region coded for RdRp), LC523807 (coded for N), LC523808 (coded for N), LC523809 (coded for N), LC528232 complete, LC528233 complete, LC529905 complete, LR757995 complete, LR757996 complete, LR757997 complete, gapped, LR757998 complete, MN938384 complete, MN938385RdRP, MN938386 RdRP, MN938387 S, MN938388 S, MN938389 S, MN938390 S, MN970003 RdRP, MN970004 RdRP, MN975262 complete, MN975263 RdRP, MN975264 RdRP, MN975265RdRP [1–3].

#### Open reading frame finder

ORF finder searches for open reading frames (ORFs) in the DNA sequence you enter. The program returns the range of each ORF, along with its protein translation. Use ORF finder to search newly sequenced DNA for potential protein encoding segments, verify predicted protein using newly developed SMART BLAST or regular BLASTP [1,4,5].

After genome alignment, the whole genome was searched for ORF domains using SMART BLAST. Quality parameters like minimal ORF length 75 with standard genetic code having ATG and initiation codons been set.

#### Alignment of nucleotide and amino acid sequence analysis

Nucleotide sequence editing was conducted using WebDSV 2.0. Protein alignment was done using Clustalw and protein to DNA sequence comparison done using pairwise alignment EMBL EBI tools [6,7]. The homology model prediction was carried out through searching in RCSB database included in Fold and Function Assignment System. Prediction Binding pockets was done online dockingserver.com. 3D structure structures are aligned by Autodock and pymol structure alignment tools.

## COMPUTATIONAL METHODS

Docking calculations were carried out using Docking Server. Gasteiger partial charges were added to the ligand atoms. Non-polar hydrogen atoms were merged, and rotatable bonds were defined [8–10].

Docking calculations were carried out on selected ligands to SARS-CoV-2 main protease PDB ID 6LU7, Human furin PDB ID6HZD, PDB ID 3E9S papain like protease and PDB ID 6NUR Nsp12 of SARS virus. Essential hydrogen atoms, Kollman united atom type charges, and solvation parameters were added with the aid of Autodock tools [11]. Affinity (grid) maps of Å grid points and 0.375 Å spacing were generated using the Autogrid program [11]. Swiss protein modelling and Autodock tools are used for protein clean. Autodock parameter set- and distance-dependent dielectric functions were used in the calculation of the van der Waals and the electrostatic terms, respectively [11–13].

Docking simulations were performed using the Lamarckian genetic algorithm (LGA) and the Solis & Wets local search method [12]. Initial position, orientation and torsions of the ligand molecules were set randomly. All rotatable torsions were released during docking. Each docking experiment was derived from 100 different runs that were set to terminate after a maximum of 2500000 energy evaluations. The population size was set to 150. During the search, a translational step of 0.2 Å and quaternion and torsion steps of 5 were applied.

## RESULTS

### Homology genome blast and genomes information

**Figure 2.**
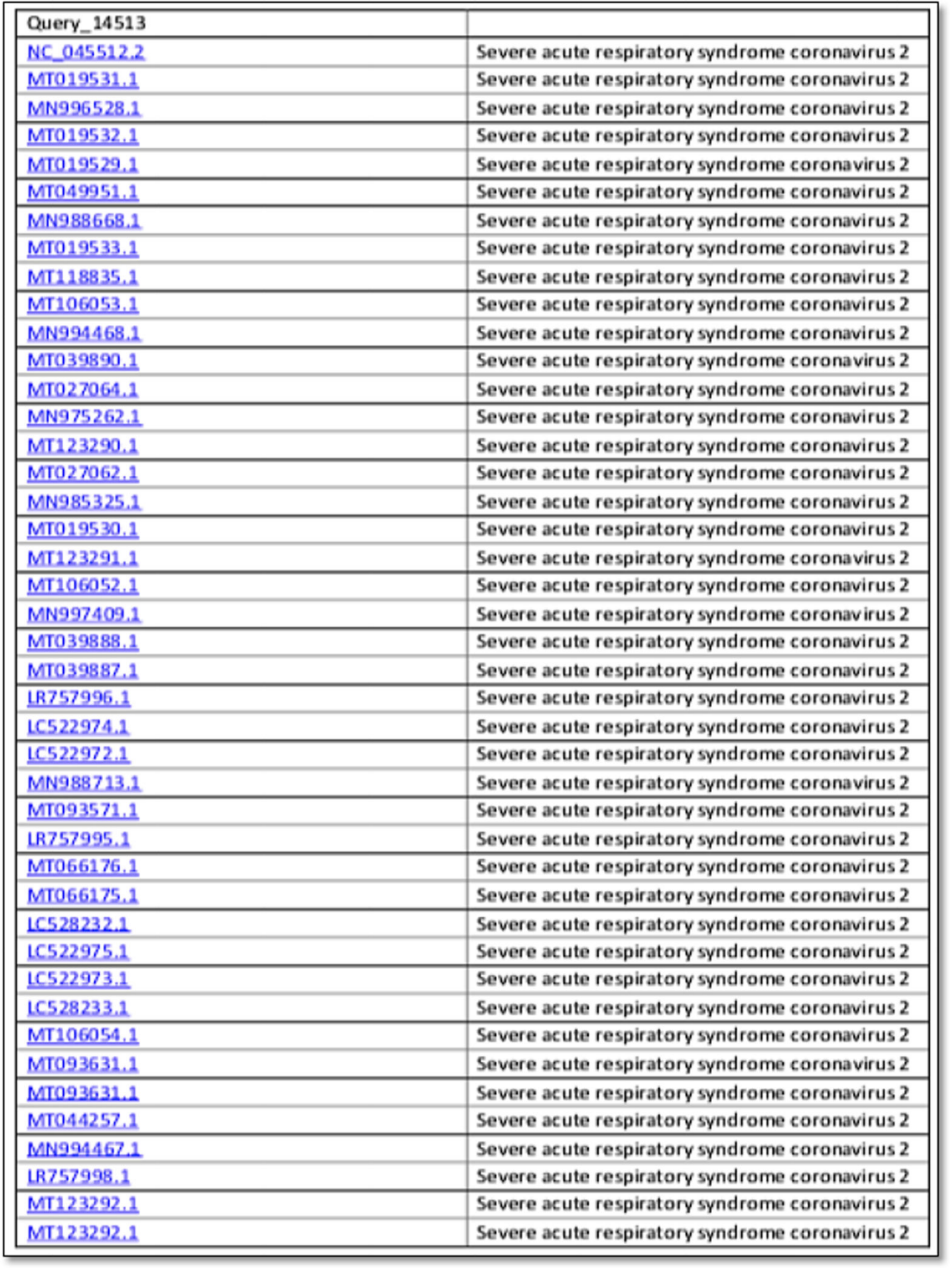

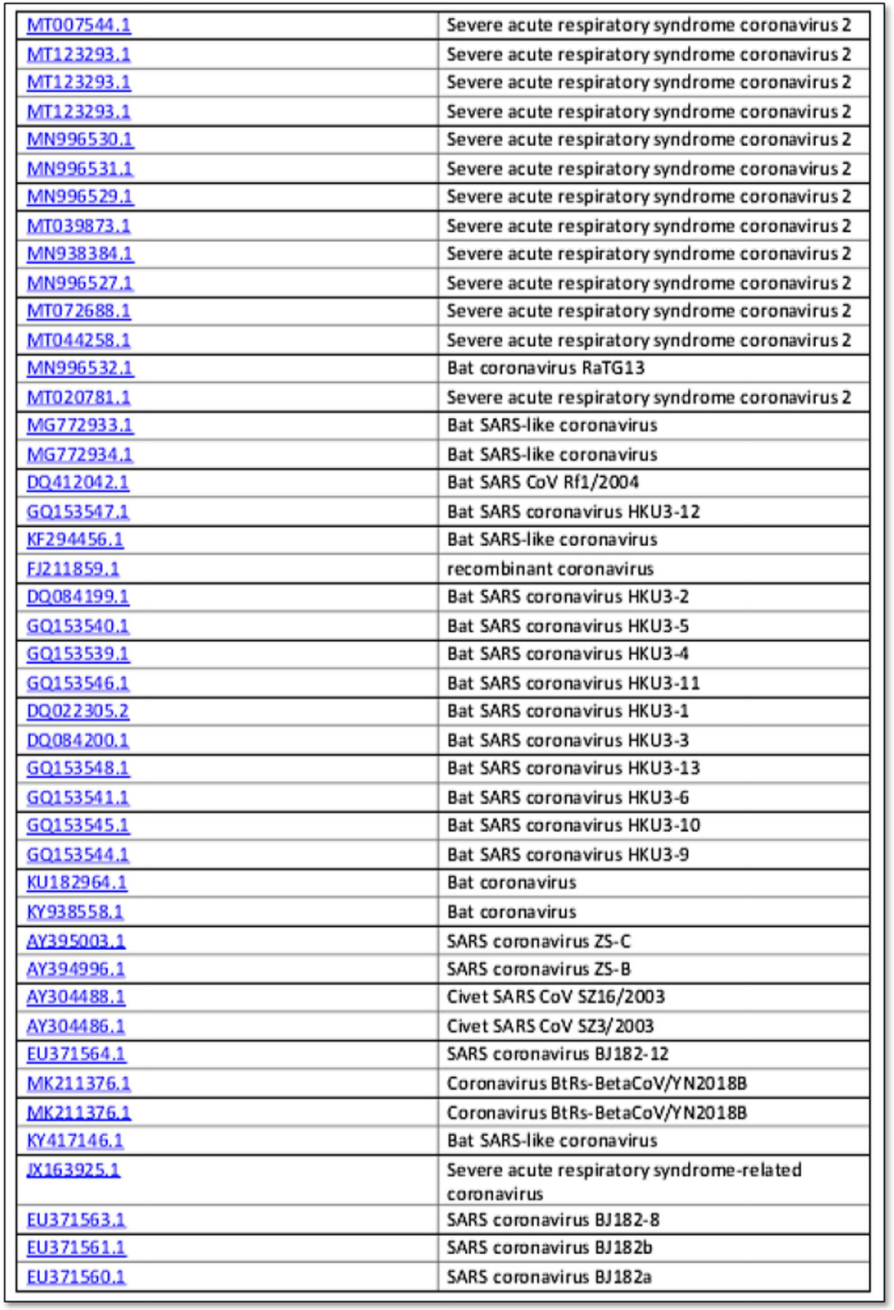

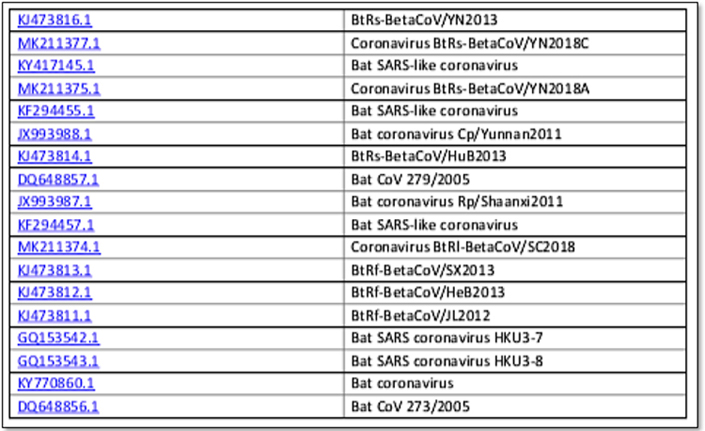
Multiple sequence alignment viewer of distance tree of genome of SARS-CoV-2 genetic ID MN908947.3.

**Figure 3.**
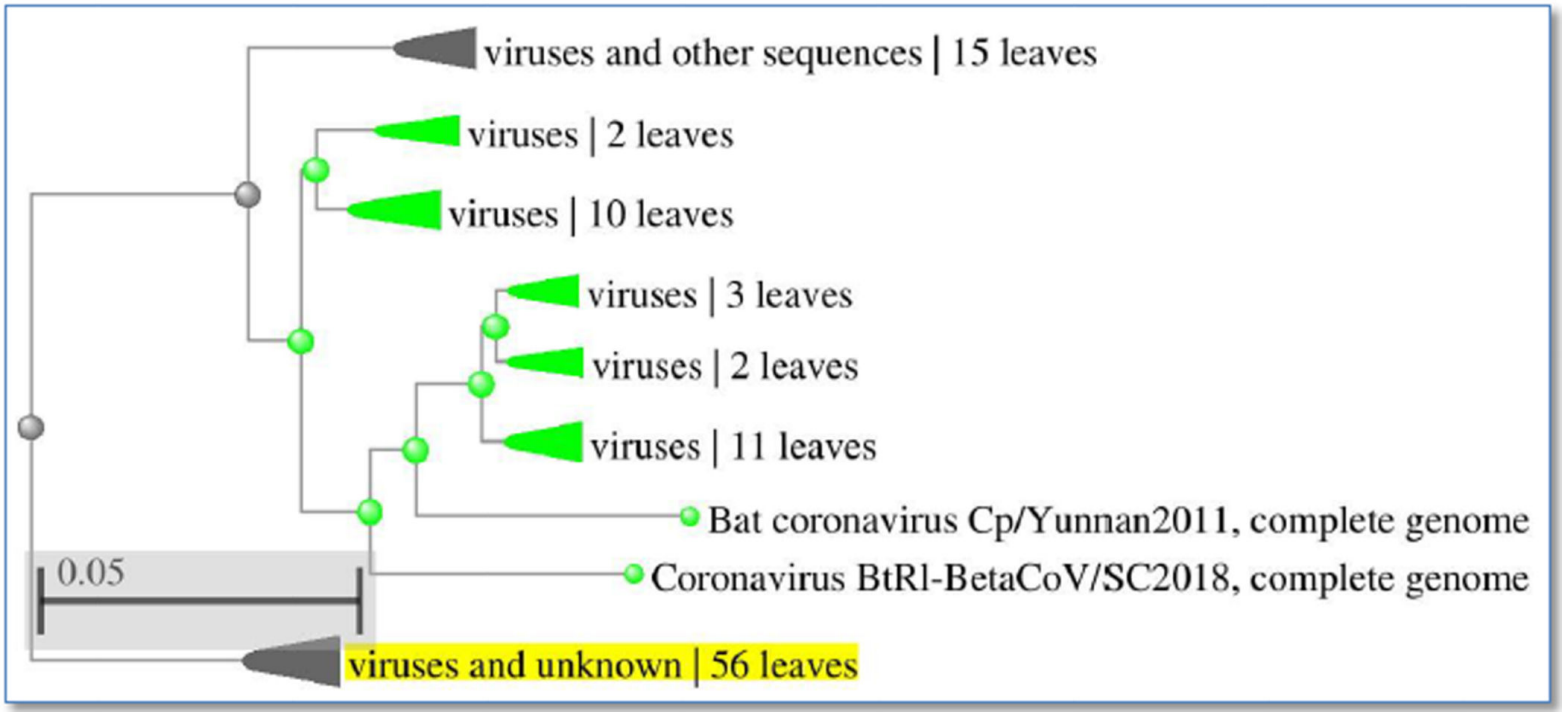
Distance tree of SARS-CoV-2 viral genome genetic ID MN908947.3 by blastn suite.

**Figure 4.**
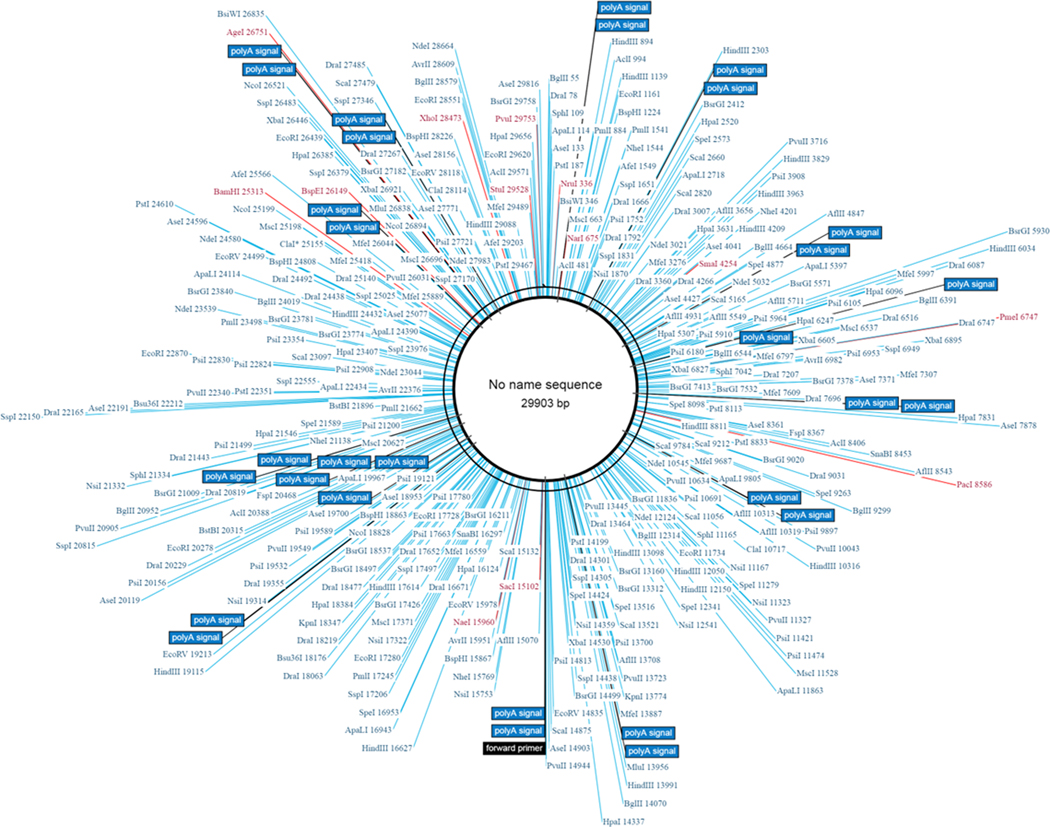
Circular genome-Forward primer sites in whole Genome of SARS-CoV-2.

**Figure 5.**
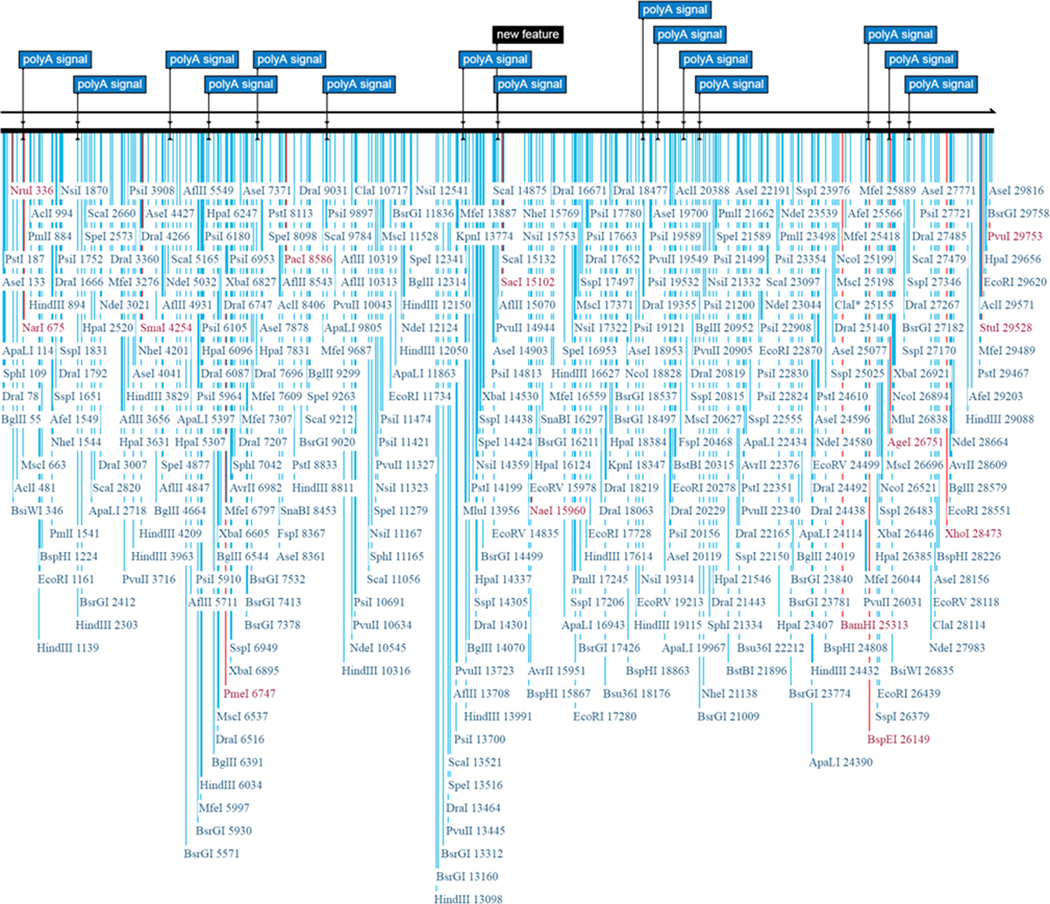
Linear genome-Forward primer sites in whole Genome of SARS-CoV-2.

Genetic ID MN908947 SARS-CoV-2 isolate Wuhan-Hu-1, complete genome after BLASTn similarity search had shown more similarity with many bat coronaviruses, some unknown virus and for some synthetic recombinant virus with genetic ID FJ211859.1 see Figure 2 and Figure 3 for whole genome and distance tree analysis. After whole genome alignment in WebDSV 2.0 tools, forward and reverse primers identified as shown in Figure 4 and Figure 5 both circular and linear alignments for 29903 bp.

### Open reading frame finder

SMART BLAST analysis shows more than 283 open reading frames shown in supplementary file orf finder-NCBI and in Table 1. ORF16, ORF5, ORF8 had shown most proteins coding for mono-ADP-ribosyltransferase PARP protein families, helicases, coronavirus family proteins NSP11 and NSP13, papain like viral protease, Pfam super family proteins of orthocoronaviridae, APA3 viroporin: Coronavirus accessory protein 3a, orf3a protein of coronaviridae. ORF120 coded for BAT SARS coronavirus HKU3, HKU3–2 and HKU3–9 mainly origin from Rhinolophus affinis an Intermediate horseshoe bat widely available in Asia. ORF238 codes for enzymes dimethylaniline monooxygenase. All positive strand ORF’s coded for Bat SARS coronavirus related proteins.

### Docking results

Selected paritaprevir, entecavir, ergotamine tartrate, telaprevir, dihydroergotamine, simeprevir, ergotamine alkaloid, telmisartan, ritonavir tartrate, fgi 106, corosolic acid, chloroquine, darunavir, nelfinavir, glycyrrhizic acid, baicalin, ritonavir, quilajja saponin, lopinavir, amprenavir, fosamprenavir, quercetin, remdesivir, pemetrexed, raltitrexed, sofosbuvir were docked against proteins SARS-CoV-2 main protease PDB ID 6LU7, Human furinPDB ID6HZD, PDB ID 3E9S papain like protease and PDB ID 6NUR Nsp12 (RdRp) in selective manner as mentioned in (Tables 2, 3 and 4).

Paritaprevir, chloroquine and ritonavir had shown strong multi target inhibition like spike proteins, proteases and furin. Natural compounds like baicalin, corosolic acid had shown multi target inhibition properties against spike proteins, proteases and furin.

## DISCUSSION

At present world is facing pandemic situation because of SARS-CoV-2 infection. There is an urgency to address this situation as no present treatment protocols are not been established. The only way to develop quick treatment protocols can be achieved by studying detailed case studies of SARS infections caused by influenza and non-influenza viruses and also studying existing antiviral drugs.

**Figure 6.**
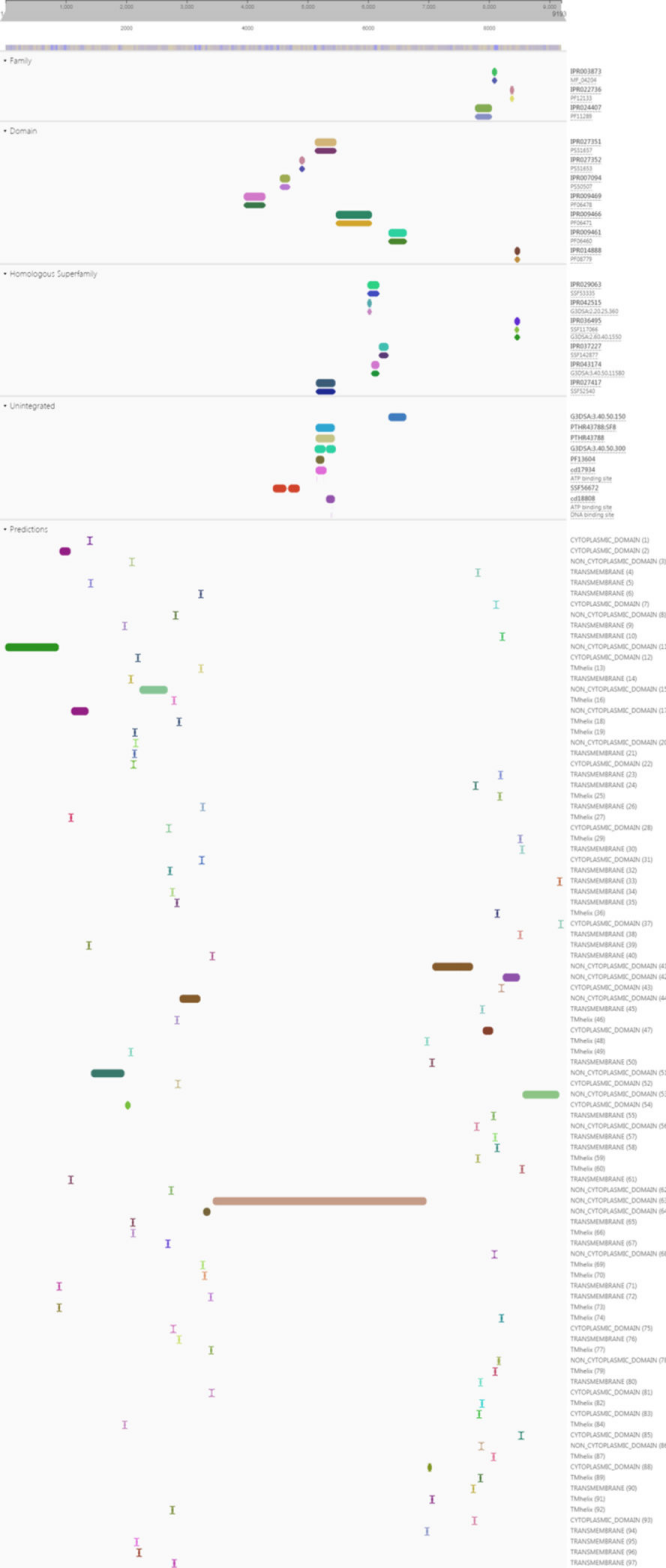
ProrVista tool analysis of ORF reads of SARS-CoV-2.

Computational and high throughput screening tools are the best aids to design and study the efficacy of existing antiviral drugs along with some anti-inflammatory drugs against SARS-CoV-2 targeted sites. Antiviral drugs like oseltamivir used against neuraminidase of SARS in last decade, favilavir an RNA-Dependent RNA polymerase (RdRp) inhibitor also showed effective against the SARS influenza virus. Recently Japan also proposed use of favipiravir and Avian flu drug to treat SARS-CoV-2 infection. Remdesivir a proposed drug to treat Ebola virus also been proposed to test against SARS-CoV-2. DNA and RNA inhibitors like sofosbuvir and anti-HIV drug compositions also been proposed at present to treat the present global pandemic caused by novel coronavirus. Most of the proposed drugs had shown either less efficacy or effective in some patients but not achieved complete success. In order to develop complete treatment protocol, one should understand the disease pathogenesis. As per case reports available study indicates respiratory outburst due to various inflammatory study indicates severe diarrhoea and respiratory outburst due to inflammatory factors causing death among novel coronavirus infected patients. As per our study we found CD4+ activation leading to TH1 and TH2 cytokines outburst in excessive leading to severe respiratory illness in patients affected by SARS-CoV-2. This virus has Orf zone indicating C lectin type binding receptors of host (Figure 4, 5 and 6) which may make this virus to escape MHC Class I antigen presentation leading to asymptomatic conditions in some patients. Interleukins like IL6, IL8 and IL2 along with TNFα might be main causative inflammatory leading respiratory failure. Based on available case study by [14] most of the patients admitted had shown difficulty in breathing, cough and fever with severe respiratory illness and pneumonia. In this study we propose use of multi target therapy which includes viral protein targets involving in host cell entry and replication and host cytokines. Viral proteins like spike, neuraminidase, main protease (3CLpro), papain like protease (PLpro) and RNA-Dependent RNA polymerase (RdRp) are the key viral protein targets [2]. Inhibition of spike (S) protein binding to ACE 2 will be key prophylactic drug discovery to control SARS-CoV-2.

## CONCLUSION

**Figure 7.**
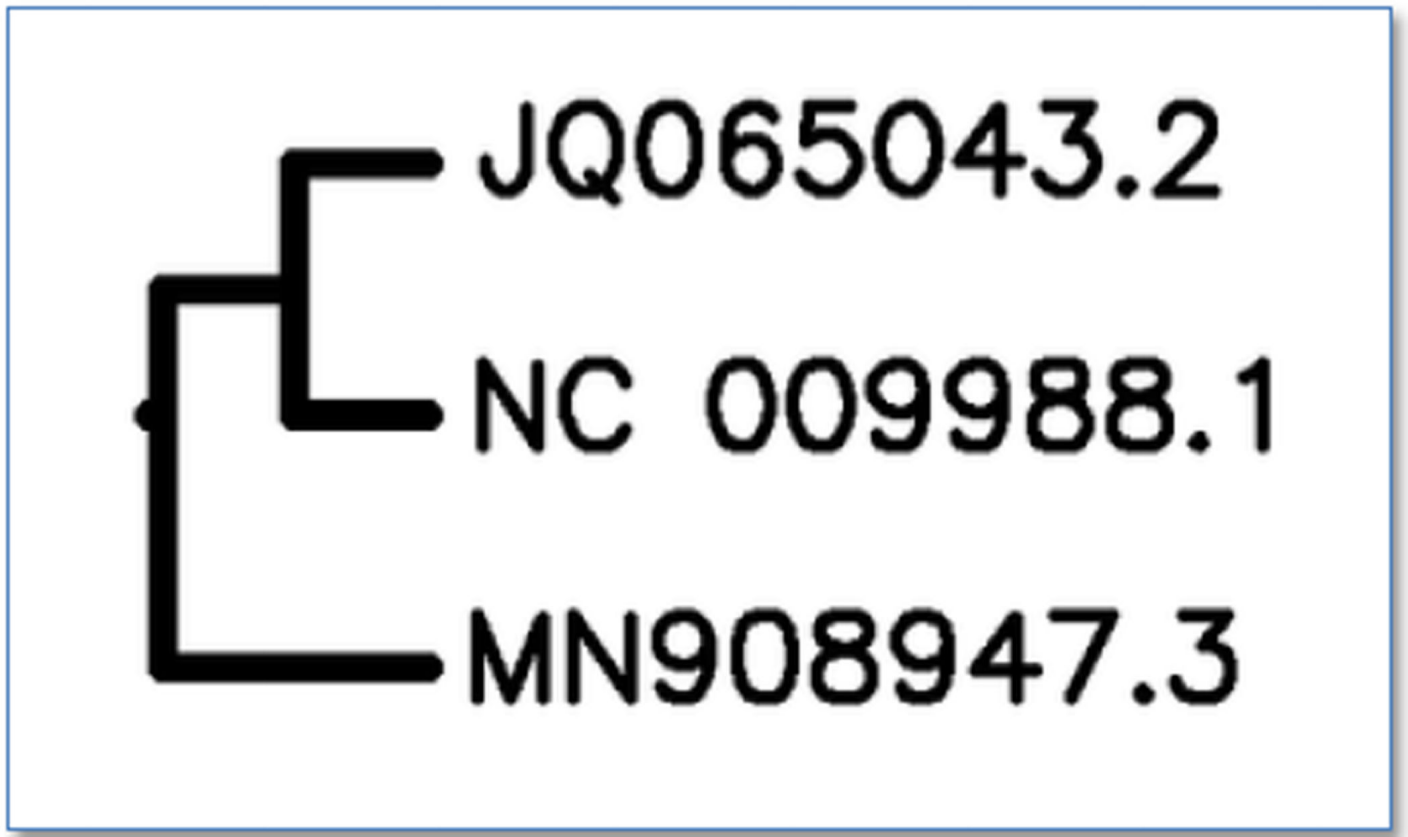
VISTA GENOME TOOL COMPRATIVE ANALYSIS-JQ065043.2–HKU2 swine corona virus, NC 009988.1 HKU15 SARS coronavirus from horseshoe bats (Rhinolophus) & MN908947.3 SARS-CoV-2.

The present used carried out using computational and high throughput screening tools in order to evaluate the whole Genome analysis of SARS-CoV-2 and identifying potential drugs to treat novel coronavirus influenza. Gene sequence was obtained from NCBI genome database [15,3] and Molbiol and other BLAST analysis tools were used to analyse genome wide study. Similarity search analysis had shown possible close species relation with BAT SARS Corona virus particularly from Intermediate horseshoe bat (Rhinolophus affinis) and some Beta Coronaviridae family. The data also suggest some possible cross species interaction of Delta coronavirus families and species jump from bats to intermediate host which is unknown or from porcine origin. VISTA Tools for Comparative Genomics had shown some phylogenetic origin of SARS-CoV-2 by chimeric recombination between HKU2 alpha Coronaviridae which caused severe Swine diarrhoea syndrome caused by Bat droppings and HKU15 a delta corona virus causing swine respiratory syndrome (Figure 7). Some genome wide analysis also matches with Recombination Clone of SARS Coronavirus with genetic ID FJ211859.1 which should be properly evaluated as future indication. Orf reading had shown more than main 238 Orf sites SARS coronavirus Orf3/3a (Figure 6) which is a characteristic protein for SARS Coronavirus family. Some other proteins include NS3/E, small non-structural proteins, well conserved among Coronavirus strains and a small uncharacteristic protein SARS_NS6 with small amino acid sequence. Drugs selected from zinc database like remdesivir, paritaprevir, sofosbuvir, ritonavir, lopinavir, chloroquine derivatives like hydroxychloroquine including natural molecules like glycyrrhizin, corosolic acid and baicalin were used as ligands in docking studies against viral proteins like spike, main protease (3CLpro). Papain like protease (PLpro), RNA dependent RNA polymerase. Docking results had shown paritaprevir, ritonavir and chloroquine derivatives as best drug leads against spike and proteases of SARS-Co-V2. Natural drugs like glycyrrhizin, corosolic acid and baicalin also shown strong binding affinity against spike and protease proteins of novel corona virus. From existing clinical data, we also propose use of anti-inflammatory drugs in treating the SARS-CoV-2 disease progression. In this study we propose for clinical study by combined use of paritaprevir, entecavir, ritonavir, and hydroxychloroquine along with anti-inflammatory drugs and also use of niacinamide, vitamin C, zinc supplements for possible good clinical outcome. We also propose study plant protease inhibitors (PI’s), glycoprotein-based antibodies and small molecules like Lysozyme hydrochloride, Oxamniquine and Nateglinide therapies.

## Supplementary Material

supplement1DISTANCE TREE RESULT OF SARS COV 2 GENOME

REVERSE PRIMERS IN WHOLE GENOME OF SARS CoV 2

supplement3orf best hits

supplement5SEQUENCE SIMILARITY HOMOLOGY

supplement4ORFfinder - NCBI

supplement7FORWARD PRIMERS IN WHOLE GENOME

supplement8SARS COV2 ALIGHNED WHOLE GENOME TO TRANSALTED ALIGHTMENT

supplement8SARS COV2 WHOLE GENOME ALIGHMENT AND SITES

supplement9Taxonomy

supplement2Factsheet_SmartBLAST

supplement10GenBank_ MN908947.3

supplement6Smart BLAST orf whole genome

## Figures and Tables

**Table 1.** Open reading frames in whole genome of SARS-CoV-2.

Label	Strand	Frame	Start	Stop	Length (nt | aa)
**ORF16**	+	2	266	13483	13218 | 4405
**ORF5**	+	1	13768	21555	7788 | 2595
**ORF42**	+	2	21521	25384	3864 | 1287
**ORF50**	+	2	28274	29533	1260 | 419
**ORF8**	+	1	25393	26220	828 | 275
**ORF117**	+	3	26499	27191	693 | 230
**ORF215**	−	2	1843	1391	453 | 150
**ORF278**	−	3	2712	2290	423 | 140
**ORF119**	+	3	27894	28259	366 | 121
**ORF12**	+	1	27394	27759	366 | 121
**ORF214**	−	2	2917	2561	357 | 118
**ORF220**	−	3	29151	28813	339 | 112
**ORF269**	−	3	6489	6187	303 | 100
**ORF120**	+	3	28278	28577	300 | 99
**ORF180**	−	2	23494	23198	297 | 98
**ORF98**	+	3	21918	22199	282 | 93
**ORF168**	−	2	29140	28862	279 | 92
**ORF234**	−	3	23349	23074	276 | 91
**ORF229**	−	3	25368	25099	270 | 89
**ORF161**	−	1	3263	3006	258 | 85
**ORF65**	+	3	2958	3206	249 | 82
**ORF121**	+	3	28710	28955	246 | 81
**ORF238**	−	3	20010	19765	246 | 81
**ORF217**	−	2	667	422	246 | 81
**ORF233**	−	3	23919	23680	240 | 79
**ORF193**	−	2	12355	12122	234 | 77
**ORF9**	+	1	26245	26472	228 | 75
**ORF97**	+	3	21639	21863	225 | 74
**ORF173**	−	2	25807	25586	222 | 73
**ORF56**	+	3	888	1097	210 | 69
**ORF102**	+	3	22884	23093	210 | 69
**ORF88**	+	3	10191	10400	210 | 69
**ORF21**	+	2	15461	15667	207 | 68
**ORF167**	−	2	29413	29207	207 | 68
**ORF140**	−	1	18185	17979	207 | 68
**ORF257**	−	3	11904	11701	204 | 67
**ORF171**	−	2	27007	26804	204 | 67
**ORF254**	−	3	12486	12283	204 | 67
**ORF181**	−	2	22111	21911	201 | 66
**ORF128**	−	1	26378	26181	198 | 65
**ORF132**	−	1	21038	20844	195 | 64
**ORF75**	+	3	6156	6350	195 | 64
**ORF68**	+	3	3912	4103	192 | 63
**ORF150**	−	1	13550	13359	192 | 63
**ORF225**	−	3	27225	27034	192 | 63
**ORF48**	+	2	26684	26872	189 | 62
**ORF100**	+	3	22539	22724	186 | 61
**ORF205**	−	2	6019	5834	186 | 61
**ORF232**	−	3	24111	23926	186 | 61
**ORF11**	+	1	27202	27387	186 | 61
**ORF25**	+	2	16616	16798	183 | 60
**ORF282**	−	3	315	133	183 | 60
**ORF260**	−	3	10995	10819	177 | 58
**ORF126**	−	1	29552	29376	177 | 58
**ORF164**	−	1	692	516	177 | 58
**ORF252**	−	3	13053	12880	174 | 57
**ORF253**	−	3	12765	12592	174 | 57
**ORF243**	−	3	18324	18151	174 | 57
**ORF147**	−	1	14486	14313	174 | 57
**ORF115**	+	3	25524	25697	174 | 57
**ORF55**	+	3	711	881	171 | 56
**ORF261**	−	3	10767	10597	171 | 56
**ORF74**	+	3	5919	6089	171 | 56
**ORF78**	+	3	7542	7709	168 | 55
**ORF250**	−	3	15159	14992	168 | 55
**ORF108**	+	3	23874	24041	168 | 55
**ORF87**	+	3	9951	10115	165 | 54
**ORF280**	−	3	1689	1528	162 | 53
**ORF196**	−	2	10384	10223	162 | 53
**ORF59**	+	3	1578	1739	162 | 53
**ORF67**	+	3	3594	3752	159 | 52
**ORF118**	+	3	27729	27887	159 | 52
**ORF192**	−	2	13273	13115	159 | 52
**ORF231**	−	3	24561	24406	156 | 51
**ORF95**	+	3	13311	13466	156 | 51
**ORF242**	−	3	18543	18388	156 | 51
**ORF36**	+	2	19148	19303	156 | 51
**ORF178**	−	2	24178	24023	156 | 51
**ORF125**	−	1	29840	29685	156 | 51
**ORF207**	−	2	5641	5486	156 | 51
**ORF275**	−	3	3963	3811	153 | 50
**ORF265**	−	3	8949	8797	153 | 50
**ORF237**	−	3	21135	20983	153 | 50
**ORF272**	−	3	5022	4873	150 | 49
**ORF52**	+	3	276	425	150 | 49
**ORF26**	+	2	16973	17122	150 | 49
**ORF190**	−	2	14059	13910	150 | 49
**ORF40**	+	2	20993	21142	150 | 49
**ORF157**	−	1	6404	6258	147 | 48
**ORF138**	−	1	19292	19146	147 | 48
**ORF256**	−	3	12078	11932	147 | 48
**ORF155**	−	1	10649	10503	147 | 48
**ORF135**	−	1	20150	20007	144 | 47
**ORF127**	−	1	28871	28728	144 | 47
**ORF146**	−	1	14651	14508	144 | 47
**ORF191**	−	2	13441	13298	144 | 47
**ORF70**	+	3	4692	4835	144 | 47
**ORF136**	−	1	19856	19713	144 | 47
**ORF244**	−	3	17994	17851	144 | 47
**ORF186**	−	2	16939	16799	141 | 46
**ORF17**	+	2	14288	14428	141 | 46
**ORF203**	−	2	7825	7688	138 | 45
**ORF112**	+	3	24606	24743	138 | 45
**ORF174**	−	2	24988	24851	138 | 45
**ORF197**	−	2	10177	10040	138 | 45
**ORF104**	+	3	23220	23357	138 | 45
**ORF189**	−	2	15703	15566	138 | 45
**ORF4**	+	1	10951	11088	138 | 45
**ORF202**	−	2	8371	8237	135 | 44
**ORF273**	−	3	4458	4324	135 | 44
**ORF33**	+	2	18392	18523	132 | 43
**ORF222**	−	3	27771	27640	132 | 43
**ORF76**	+	3	7236	7367	132 | 43
**ORF156**	−	1	7538	7407	132 | 43
**ORF86**	+	3	9201	9329	129 | 42
**ORF23**	+	2	16151	16279	129 | 42
**ORF105**	+	3	23385	23513	129 | 42
**ORF90**	+	3	12318	12443	126 | 41
**ORF43**	+	2	25457	25582	126 | 41
**ORF177**	−	2	24367	24242	126 | 41
**ORF133**	−	1	20576	20451	126 | 41
**ORF13**	+	1	28066	28191	126 | 41
**ORF6**	+	1	24688	24813	126 | 41
**ORF109**	+	3	24045	24170	126 | 41
**ORF122**	+	3	28962	29084	123 | 40
**ORF264**	−	3	9288	9166	123 | 40
**ORF148**	−	1	14027	13905	123 | 40
**ORF245**	−	3	17628	17506	123 | 40
**ORF14**	+	1	29173	29295	123 | 40
**ORF216**	−	2	1021	899	123 | 40
**ORF83**	+	3	8856	8975	120 | 39
**ORF114**	+	3	25329	25448	120 | 39
**ORF221**	−	3	28413	28297	117 | 38
**ORF18**	+	2	14636	14752	117 | 38
**ORF107**	+	3	23640	23756	117 | 38
**ORF169**	−	2	28522	28406	117 | 38
**ORF34**	+	2	18647	18763	117 | 38
**ORF279**	−	3	2046	1930	117 | 38
**ORF51**	+	2	29558	29674	117 | 38
**ORF110**	+	3	24213	24329	117 | 38
**ORF113**	+	3	24762	24875	114 | 37
**ORF200**	−	2	8785	8672	114 | 37
**ORF71**	+	3	5103	5216	114 | 37
**ORF208**	−	2	5011	4901	111 | 36
**ORF1**	+	1	5803	5913	111 | 36
**ORF142**	−	1	17276	17166	111 | 36
**ORF266**	−	3	8754	8644	111 | 36
**ORF45**	+	2	26060	26170	111 | 36
**ORF111**	+	3	24330	24440	111 | 36
**ORF28**	+	2	17606	17716	111 | 36
**ORF24**	+	2	16493	16603	111 | 36
**ORF54**	+	3	576	686	111 | 36
**ORF277**	−	3	2949	2842	108 | 35
**ORF153**	−	1	12083	11976	108 | 35
**ORF123**	+	3	29160	29267	108 | 35
**ORF124**	+	3	29343	29450	108 | 35
**ORF139**	−	1	18578	18471	108 | 35
**ORF159**	−	1	4298	4191	108 | 35
**ORF211**	−	2	3466	3359	108 | 35
**ORF210**	−	2	3685	3578	108 | 35
**ORF62**	+	3	2208	2312	105 | 34
**ORF204**	−	2	7336	7232	105 | 34
**ORF81**	+	3	7998	8102	105 | 34
**ORF267**	−	3	8148	8044	105 | 34
**ORF85**	+	3	9090	9194	105 | 34
**ORF201**	−	2	8581	8477	105 | 34
**ORF92**	+	3	12669	12773	105 | 34
**ORF184**	−	2	17377	17273	105 | 34
**ORF271**	−	3	5577	5473	105 | 34
**ORF106**	+	3	23520	23624	105 | 34
**ORF31**	+	2	18086	18187	102 | 33
**ORF53**	+	3	432	533	102 | 33
**ORF281**	−	3	1110	1009	102 | 33
**ORF224**	−	3	27456	27355	102 | 33
**ORF61**	+	3	1866	1967	102 | 33
**ORF63**	+	3	2583	2684	102 | 33
**ORF57**	+	3	1128	1229	102 | 33
**ORF255**	−	3	12255	12154	102 | 33
**ORF84**	+	3	8982	9083	102 | 33
**ORF240**	−	3	18978	18877	102 | 33
**ORF219**	−	3	29511	29410	102 | 33
**ORF152**	−	1	13058	12957	102 | 33
**ORF154**	−	1	10856	10755	102 | 33
**ORF116**	+	3	25968	26069	102 | 33
**ORF195**	−	2	11596	11498	99 | 32
**ORF283**	−	3	108	10	99 | 32
**ORF274**	−	3	4236	4138	99 | 32
**ORF235**	−	3	22599	22501	99 | 32
**ORF236**	−	3	21372	21274	99 | 32
**ORF199**	−	2	9493	9395	99 | 32
**ORF239**	−	3	19101	19003	99 | 32
**ORF268**	−	3	7419	7321	99 | 32
**ORF248**	−	3	15888	15790	99 | 32
**ORF263**	−	3	10371	10273	99 | 32
**ORF39**	+	2	20165	20263	99 | 32
**ORF187**	−	2	16306	16208	99 | 32
**ORF158**	−	1	5546	5448	99 | 32
**ORF141**	−	1	17543	17445	99 | 32
**ORF46**	+	2	26183	26281	99 | 32
**ORF10**	+	1	26812	26910	99 | 32
**ORF131**	−	1	21551	21453	99 | 32
**ORF47**	+	2	26456	26554	99 | 32
**ORF143**	−	1	16295	16200	96 | 31
**ORF213**	−	2	3190	3095	96 | 31
**ORF212**	−	2	3298	3203	96 | 31
**ORF276**	−	3	3450	3355	96 | 31
**ORF103**	+	3	23106	23201	96 | 31
**ORF259**	−	3	11154	11059	96 | 31
**ORF179**	−	2	23632	23537	96 | 31
**ORF66**	+	3	3207	3302	96 | 31
**ORF246**	−	3	17106	17011	96 | 31
**ORF194**	−	2	11842	11747	96 | 31
**ORF91**	+	3	12480	12572	93 | 30
**ORF228**	−	3	25602	25510	93 | 30
**ORF72**	+	3	5565	5657	93 | 30
**ORF44**	+	2	25892	25984	93 | 30
**ORF77**	+	3	7377	7469	93 | 30
**ORF188**	−	2	15991	15899	93 | 30
**ORF160**	−	1	3830	3738	93 | 30
**ORF7**	+	1	25195	25287	93 | 30
**ORF163**	−	1	1904	1812	93 | 30
**ORF270**	−	3	6072	5980	93 | 30
**ORF19**	+	2	14765	14857	93 | 30
**ORF206**	−	2	5779	5687	93 | 30
**ORF172**	−	2	26536	26447	90 | 29
**ORF249**	−	3	15696	15607	90 | 29
**ORF185**	−	2	17200	17111	90 | 29
**ORF80**	+	3	7893	7982	90 | 29
**ORF251**	−	3	14367	14278	90 | 29
**ORF176**	−	2	24595	24506	90 | 29
**ORF101**	+	3	22776	22865	90 | 29
**ORF129**	−	1	25454	25365	90 | 29
**ORF134**	−	1	20438	20349	90 | 29
**ORF227**	−	3	26127	26038	90 | 29
**ORF170**	−	2	28105	28016	90 | 29
**ORF175**	−	2	24811	24725	87 | 28
**ORF60**	+	3	1770	1856	87 | 28
**ORF22**	+	2	15812	15898	87 | 28
**ORF247**	−	3	16818	16732	87 | 28
**ORF183**	−	2	17854	17768	87 | 28
**ORF30**	+	2	17966	18052	87 | 28
**ORF144**	−	1	15617	15531	87 | 28
**ORF73**	+	3	5697	5783	87 | 28
**ORF35**	+	2	18764	18847	84 | 27
**ORF2**	+	1	8815	8898	84 | 27
**ORF32**	+	2	18284	18367	84 | 27
**ORF41**	+	2	21317	21400	84 | 27
**ORF58**	+	3	1476	1559	84 | 27
**ORF258**	−	3	11577	11494	84 | 27
**ORF151**	−	1	13277	13194	84 | 27
**ORF165**	−	1	227	144	84 | 27
**ORF182**	−	2	18703	18620	84 | 27
**ORF94**	+	3	13005	13088	84 | 27
**ORF166**	−	2	29500	29417	84 | 27
**ORF145**	−	1	15143	15060	84 | 27
**ORF130**	−	1	22352	22269	84 | 27
**ORF223**	−	3	27621	27538	84 | 27
**ORF137**	−	1	19700	19617	84 | 27
**ORF82**	+	3	8745	8825	81 | 26
**ORF162**	−	1	2561	2481	81 | 26
**ORF27**	+	2	17393	17473	81 | 26
**ORF29**	+	2	17831	17911	81 | 26
**ORF149**	−	1	13631	13551	81 | 26
**ORF226**	−	3	26226	26146	81 | 26
**ORF262**	−	3	10509	10429	81 | 26
**ORF49**	+	2	27875	27955	81 | 26
**ORF99**	+	3	22314	22394	81 | 26
**ORF79**	+	3	7758	7838	81 | 26
**ORF69**	+	3	4350	4430	81 | 26
**ORF89**	+	3	12126	12206	81 | 26
**ORF15**	+	2	59	136	78 | 25
**ORF209**	−	2	4408	4331	78 | 25
**ORF20**	+	2	14858	14935	78 | 25
**ORF3**	+	1	9541	9618	78 | 25
**ORF198**	−	2	9964	9887	78 | 25
**ORF218**	−	2	310	233	78 | 25
**ORF37**	+	2	19550	19627	78 | 25
**ORF38**	+	2	19664	19741	78 | 25
**ORF230**	−	3	24966	24889	78 | 25
**ORF96**	+	3	21117	21194	78 | 25
**ORF93**	+	3	12864	12941	78 | 25
**ORF64**	+	3	2793	2870	78 | 25
**ORF241**	−	3	18777	18700	78 | 25

**Table 2.** Identified potential drug leads against protease and replication polymerase novel corona virus targets.

S. No	Drug name	Target Viral Protein	ΔG (Free Energy of Binding) kcal/mol	Inhibition Constant Ki
1.	Paritaprevir	Proteases	−9.32	147.06 nM
2.	Ergotamine tartrate	Proteases	−9.23	171.72 nM
3.	Telaprevir	Proteases	−8.98	260.28 nM
4.	Dihydroergotamine	Proteases	−8.96	270.32 nM
5.	Simeprevir	Proteases	−8.61	489.77 nM
6.	Ergotamine alkaloid	Proteases	−8.54	1.85 uM
7.	Telmisartan	Proteases	−7.36	4.03 uM
8.	Ritonavir tartrate	Proteases	−7.30	4.48 uM
9.	FGI 106	Proteases	−7.14	5.82 uM
10.	Corosolic acid	Proteases	−7.09	6.33 uM
11.	Chloroquine	Proteases	−6.96	7.94 uM
12.	Darunavir	Proteases	−6.94	8.15 uM
13.	Nelfinavir	Proteases	−6.79	10.55 uM
14.	Glycyrrhizic acid	Proteases	−6.75	11.26 uM
15.	Baicalin	Proteases	−6.58	15.00 uM
16.	Ritonavir	Proteases	−6.39	20.64 uM
17.	quilajja saponin	Proteases	−6.16	30.59 uM
18.	Lopinavir	Proteases	−5.92	45.67 uM
19.	Amprenavir	Proteases	−5.82	54.06 uM
20.	Fosamprenavir	Proteases	−4.94	240.42 uM
21.	Quercetin	Proteases	−4.74	338.05 uM
22.	Remdesivir	Proteases	−4.53	475.88 uM
23.	Pemetrexed	RdRp (viral Replication)	−6.49	17.54 uM
24.	Raltitrexed	RdRp (viral Replication)	−6.71	12.08 uM
25.	Sofosbuvir	RdRp (viral Replication)	−5.40	30.89 uM

**Table 3.** Identified potential drug leads against human furin proteases for novel corona virus targets.

S. No.	Drug Name	Target Protein	ΔG (Free Energy of Binding) kcal/mol	Inhibition Constant Ki
1.	Chloroquine	Furin	−8.61 kcal/mol	487.42 nM
2.	Baicalin	Furin	−7.40 kcal/mol	3.75 uM
3.	Corosolic acid	Furin	−7.67 kcal/mol	2.41 uM
4.	Glycyrrhizic acid	Furin	−5.84 kcal/mol	52.76 uM (mild inhibitor)
5.	Paritaprevir	Furin	−10.02 kcal/mol	45.27 nM(strong inhibitor)
6.	Ritonavir	Furin	−7.91 kcal/mol	1.58 uM
7.	Remdesivir	Furin	−4.81 kcal/mol	300.08 uM

**Table 4.** Identified potential drug leads against papain like proteases of novel corona virus targets.

S. No	Drug Name	Target Protein	ΔG (Free Energy of Binding) kcal/mol	Inhibition Constant Ki
1.	Paritaprevir	papain like proteases	−7.09 kcal/mol	6.40 uM
2.	Lopinavir	papain like proteases	−4.25 kcal/mol	772.95 uM (weak inhibitor)
3.	Ritonavir	papain like proteases	−4.73 kcal/mol	339.64 uM
4.	Chloroquine	papain like proteases	−7.28 kcal/mol	4.61 uM
5.	Remdesivir	papain like proteases	−5.66 kcal/mol	70.56 uM (weal inhibition)
