# Supplementary figures and images for: Whole Genome Analysis and Targeted Drug Discovery Using Computational Methods and High Throughput Screening Tools for Emerged Novel Coronavirus (2019-nCoV)
Source: J Pharm Drug Res. Author manuscript; Available in PMC 2020 Jul 2. (PMC7331973)

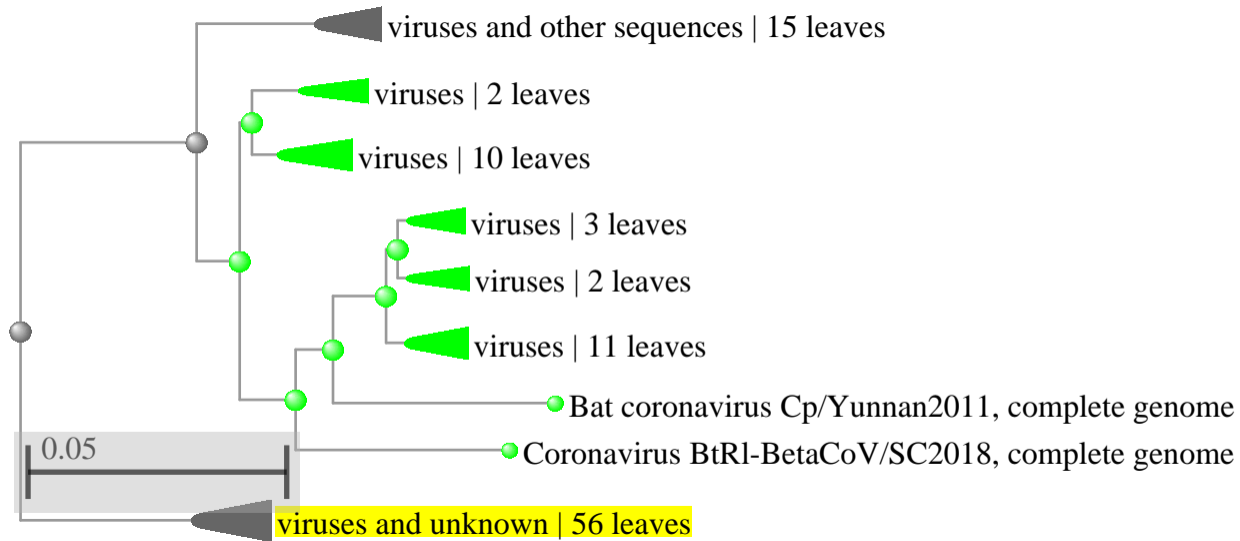

Supplement: supplement1DISTANCE TREE RESULT OF SARS COV 2 GENOME [file NIHMS1582187-supplement-supplement1DISTANCE_TREE_RESULT_OF_SARS_COV_2_GENOME.pdf]

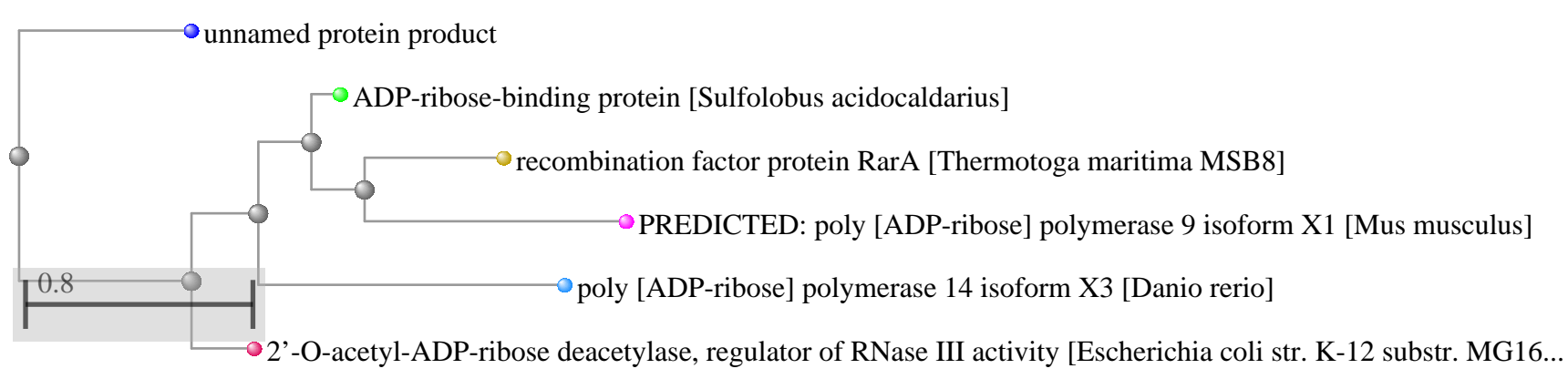

Supplement: supplement3orf best hits [file NIHMS1582187-supplement-supplement3orf_best_hits.pdf]
