# Supplementary material for: Whole Genome Analysis and Targeted Drug Discovery Using Computational Methods and High Throughput Screening Tools for Emerged Novel Coronavirus (2019-nCoV)
Source: J Pharm Drug Res. Author manuscript; Available in PMC 2020 Jul 2. (PMC7331973)
Supplement: supplement5SEQUENCE SIMILARITY HOMOLOGY [file NIHMS1582187-supplement-supplement5SEQUENCE_SIMILARITY_HOMOLOGY.pdf]

Query_14513	
NC_045512.2	Severe acute respiratory syndrome coronavirus 2
MT019531.1	Severe acute respiratory syndrome coronavirus 2
MN996528.1	Severe acute respiratory syndrome coronavirus 2
MT019532.1	Severe acute respiratory syndrome coronavirus 2
MT019529.1	Severe acute respiratory syndrome coronavirus 2
MT049951.1	Severe acute respiratory syndrome coronavirus 2
MN988668.1	Severe acute respiratory syndrome coronavirus 2
MT019533.1	Severe acute respiratory syndrome coronavirus 2
MT118835.1	Severe acute respiratory syndrome coronavirus 2
MT106053.1	Severe acute respiratory syndrome coronavirus 2
MN994468.1	Severe acute respiratory syndrome coronavirus 2
MT039890.1	Severe acute respiratory syndrome coronavirus 2
MT027064.1	Severe acute respiratory syndrome coronavirus 2
MN975262.1	Severe acute respiratory syndrome coronavirus 2
MT123290.1	Severe acute respiratory syndrome coronavirus 2
MT027062.1	Severe acute respiratory syndrome coronavirus 2
MN985325.1	Severe acute respiratory syndrome coronavirus 2
MT019530.1	Severe acute respiratory syndrome coronavirus 2
MT123291.1	Severe acute respiratory syndrome coronavirus 2
MT106052.1	Severe acute respiratory syndrome coronavirus 2
MN997409.1	Severe acute respiratory syndrome coronavirus 2
MT039888.1	Severe acute respiratory syndrome coronavirus 2
MT039887.1	Severe acute respiratory syndrome coronavirus 2
LR757996.1	Severe acute respiratory syndrome coronavirus 2
LC522974.1	Severe acute respiratory syndrome coronavirus 2
LC522972.1	Severe acute respiratory syndrome coronavirus 2
MN988713.1	Severe acute respiratory syndrome coronavirus 2
MT093571.1	Severe acute respiratory syndrome coronavirus 2
LR757995.1	Severe acute respiratory syndrome coronavirus 2
MT066176.1	Severe acute respiratory syndrome coronavirus 2
MT066175.1	Severe acute respiratory syndrome coronavirus 2
LC528232.1	Severe acute respiratory syndrome coronavirus 2
LC522975.1	Severe acute respiratory syndrome coronavirus 2
LC522973.1	Severe acute respiratory syndrome coronavirus 2
LC528233.1	Severe acute respiratory syndrome coronavirus 2
MT106054.1	Severe acute respiratory syndrome coronavirus 2
MT093631.1	Severe acute respiratory syndrome coronavirus 2
MT093631.1	Severe acute respiratory syndrome coronavirus 2
MT044257.1	Severe acute respiratory syndrome coronavirus 2
MN994467.1	Severe acute respiratory syndrome coronavirus 2
LR757998.1	Severe acute respiratory syndrome coronavirus 2
MT123292.1	Severe acute respiratory syndrome coronavirus 2
MT123292.1	Severe acute respiratory syndrome coronavirus 2

MT007544.1	Severe acute respiratory syndrome coronavirus 2
MT123293.1	Severe acute respiratory syndrome coronavirus 2
MT123293.1	Severe acute respiratory syndrome coronavirus 2
MT123293.1	Severe acute respiratory syndrome coronavirus 2
MN996530.1	Severe acute respiratory syndrome coronavirus 2
MN996531.1	Severe acute respiratory syndrome coronavirus 2
MN996529.1	Severe acute respiratory syndrome coronavirus 2
MT039873.1	Severe acute respiratory syndrome coronavirus 2
MN938384.1	Severe acute respiratory syndrome coronavirus 2
MN996527.1	Severe acute respiratory syndrome coronavirus 2
MT072688.1	Severe acute respiratory syndrome coronavirus 2
MT044258.1	Severe acute respiratory syndrome coronavirus 2
MN996532.1	Bat coronavirus RaTG13
MT020781.1	Severe acute respiratory syndrome coronavirus 2
MG772933.1	Bat SARS-like coronavirus
MG772934.1	Bat SARS-like coronavirus
DQ412042.1	Bat SARS CoV Rf1/2004
GQ153547.1	Bat SARS coronavirus HKU3-12
KF294456.1	Bat SARS-like coronavirus
FJ211859.1	recombinant coronavirus
DQ084199.1	Bat SARS coronavirus HKU3-2
GQ153540.1	Bat SARS coronavirus HKU3-5
GQ153539.1	Bat SARS coronavirus HKU3-4
GQ153546.1	Bat SARS coronavirus HKU3-11
DQ022305.2	Bat SARS coronavirus HKU3-1
DQ084200.1	Bat SARS coronavirus HKU3-3
GQ153548.1	Bat SARS coronavirus HKU3-13
GQ153541.1	Bat SARS coronavirus HKU3-6
GQ153545.1	Bat SARS coronavirus HKU3-10
GQ153544.1	Bat SARS coronavirus HKU3-9
KU182964.1	Bat coronavirus
KY938558.1	Bat coronavirus
AY395003.1	SARS coronavirus ZS-C
AY394996.1	SARS coronavirus ZS-B
AY304488.1	Civet SARS CoV SZ16/2003
AY304486.1	Civet SARS CoV SZ3/2003
EU371564.1	SARS coronavirus BJ182-12
MK211376.1	Coronavirus BtRs-BetaCoV/YN2018B
MK211376.1	Coronavirus BtRs-BetaCoV/YN2018B
KY417146.1	Bat SARS-like coronavirus
JX163925.1	Severe acute respiratory syndrome-related coronavirus
EU371563.1	SARS coronavirus BJ182-8
EU371561.1	SARS coronavirus BJ182b
EU371560.1	SARS coronavirus BJ182a

KJ473816.1	BtRs-BetaCoV/YN2013
MK211377.1	Coronavirus BtRs-BetaCoV/YN2018C
KY417145.1	Bat SARS-like coronavirus
MK211375.1	Coronavirus BtRs-BetaCoV/YN2018A
KF294455.1	Bat SARS-like coronavirus
JX993988.1	Bat coronavirus Cp/Yunnan2011
KJ473814.1	BtRs-BetaCoV/HuB2013
DQ648857.1	Bat CoV 279/2005
JX993987.1	Bat coronavirus Rp/Shaanxi2011
KF294457.1	Bat SARS-like coronavirus
MK211374.1	Coronavirus BtRl-BetaCoV/SC2018
KJ473813.1	BtRf-BetaCoV/SX2013
KJ473812.1	BtRf-BetaCoV/HeB2013
KJ473811.1	BtRf-BetaCoV/JL2012
GQ153542.1	Bat SARS coronavirus HKU3-7
GQ153543.1	Bat SARS coronavirus HKU3-8
KY770860.1	Bat coronavirus
DQ648856.1	Bat CoV 273/2005
