# Supplementary material for: Whole Genome Analysis and Targeted Drug Discovery Using Computational Methods and High Throughput Screening Tools for Emerged Novel Coronavirus (2019-nCoV)
Source: J Pharm Drug Res. Author manuscript; Available in PMC 2020 Jul 2. (PMC7331973)
Supplement: supplement4ORFfinder - NCBI [file NIHMS1582187-supplement-supplement4ORFfinder_-_NCBI.pdf]

ORFfinder PubMed ▾ Search

Open Reading Frame Viewer

Help

Sequence

ORFs found: 283 **Genetic code: 1** **Start codon: 'ATG' and alternative codons**

1 | Find: | Tools

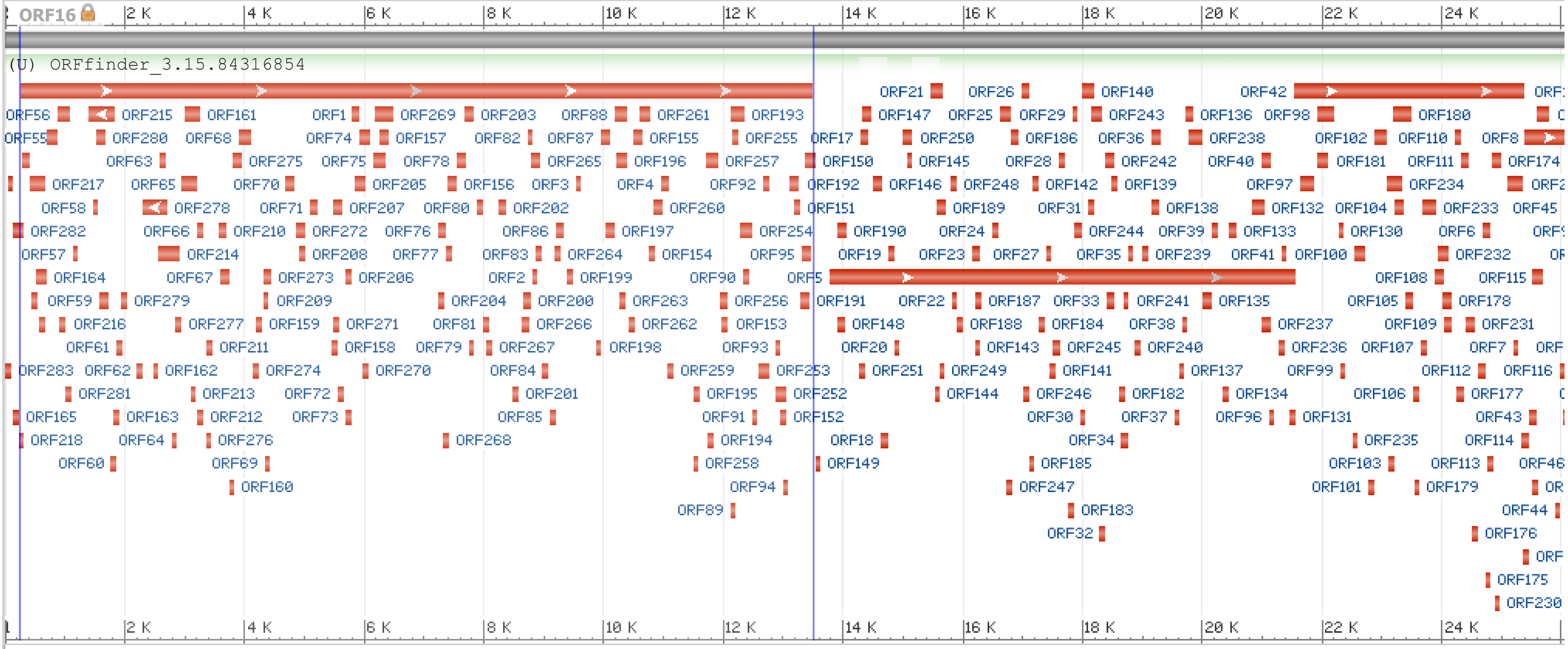

1: 1..30K (29,903 nt)

Six-frame translation...

ORF16 (4405 aa) Display ORF as... Mark

Mark subset... Marked: 0 Download marked set as Protein FASTA ▾

Label	Strand	Frame	Start	Stop	Length (nt | aa)

```
>lcl|ORF16
MESLVPGFNEKTHVQLSLPVLQVRDVLVRGFGDSVEEVLSEARQHLKDGT
CGLVEVEKGVLPQLEQPYVFIKRSDARTAPHGHVMVELVAELEGIQYGRS
GETLGVLVPHVGEIPVAYRKVLLRKNGNKGAGGHSYGADLKSFDLGDELG
TDPYEDFQENWNTKHSSGVTRELMRELNGGAYTRYVDNNFCGPDGYPLEC
IKDLLARAGKASCTLSEQLDFIDTKRGVYCCREHEHEIAWYTERSEKSYE
LQTPFEIKLAKKFDTFNGECPNFVFPLNSIIKTIQPRVEKKKLDGFMGRI
RSVYPVASPNECNQMCLSTLMKCDHCGETSWQTGDFVKATCEFCGTENLT
KEGATTCGYLPQNAVVKIYCPACHNSEVGPEHSLAEYHNESGLKTILRKG
GRTIAFGGCVFSYVGCHNKCAYWVPRASANIGCNHTGVVGEGSEGLNDNL
LEILQKEKVNINIVGDFKLNEEIAIILASFSASTSAFVETVKGLDYKAFK
QIVESCGNFKVTKGKAKKGAWNIGEQKSILSPLYAFASEAARVVRSIFSR
TLETAQNSVRVLQKAAITILDGISQYSLRLIDAMMFTSDLATNNLVVMAY
ITGGVVQLTSQWLTNIFGTVYEKLKPVLDWLEEKFKEGVEFLRDGWEIVK
FISTCACEIVGGQIVTCAKEIKESVQTFFKLVNKFLALCADSIIIGGAKL
KALNLGETFVTHSKGLYRKCVKSREETGLLMPLKAPKEIIFLEGETLPTE
VLTEEVVLKTGDLQPLEQPTSEAVEAPLVGTPVCINGLMLLEIKDTEKYC
ALAPNMMVTNNTFTLKGGAPTKVTFGDDTVIEVQGYKSVNITFELDERID
KVLNEKCSAYTVELGTEVNEFACVVADAVIKTLQPVSELLTPLGIDLDEW
SMATYYLFDESGEFKLASHMYCSFYPPDEDEEEGDCEEEEFEPSTQYEYG
```

**ORF16**	**+**	**2**	**266**	**13483**	**13218 | 4405**
ORF5	+	1	13768	21555	7788 | 2595
ORF42	+	2	21521	25384	3864 | 1287
ORF50	+	2	28274	29533	1260 | 419
ORF8	+	1	25393	26220	828 | 275
ORF117	+	3	26499	27191	693 | 230
ORF215	-	2	1843	1391	453 | 150
ORF278	-	3	2712	2290	423 | 140
ORF119	+	3	27894	28259	366 | 121
ORF12	+	1	27394	27759	366 | 121

ORF16

SmartBLAST

BLAST

Marked set (0)

SmartBLAST best hit titles...

BLAST

BLAST Database:

UniProtKB/Swiss-Prot (swissprot) ▼

Go back to the submitting page...

You are here: NCBI >

Help Desk

GETTING STARTED

NCBI Education
NCBI Help Manual
NCBI Handbook
Training & Tutorials

RESOURCES

Chemicals & Bioassays
Data & Software
DNA & RNA
Domains & Structures
Genes & Expression
Genetics & Medicine
Genomes & Maps
Homology
Literature

POPULAR

PubMed
Bookshelf
PubMed Central
PubMed Health
BLAST
Nucleotide
Genome
SNP
Gene

FEATURED

Genetic Testing Registry
PubMed Health
GenBank
Reference Sequences
Gene Expression Omnibus
Map Viewer
Human Genome
Mouse Genome
Influenza Virus

NCBI INFORMATION

About NCBI
Research at NCBI
NCBI News
NCBI FTP Site
NCBI on Facebook
NCBI on Twitter
NCBI on YouTube

Proteins
Sequence Analysis
Taxonomy
Training & Tutorials
Variation

Protein
PubChem

Primer-BLAST
Sequence Read Archive

Copyright | **Disclaimer** | **Privacy** | **Browsers** | **Accessibility** | **Contact**

National Center for Biotechnology Information, U.S. National Library of Medicine
8600 Rockville Pike, Bethesda MD, 20894 USA
