# Supplementary material for: Whole Genome Analysis and Targeted Drug Discovery Using Computational Methods and High Throughput Screening Tools for Emerged Novel Coronavirus (2019-nCoV)
Source: J Pharm Drug Res. Author manuscript; Available in PMC 2020 Jul 2. (PMC7331973)
Supplement: supplement7FORWARD PRIMERS IN WHOLE GENOME [file NIHMS1582187-supplement-supplement7FORWARD_PRIMERS_IN_WHOLE_GENOME.docx]

new feature

BglIIDraI

1 ATTAAAGGTTTATACCTTCCCAGGTAACAAACCAACCAACTTTCGATCTCTTGTAGATCTGTTCTCTAAACGAACTTTAA 80

TAATTTCCAAATATGGAAGGGTCCATTGTTTGGTTGGTTGAAAGCTAGAGAACATCTAGACAAGAGATTTGCTTGAAATT

SphIApaLIAseI

81 AATCTGTGTGGCTGTCACTCGGCTGCATGCTTAGTGCACTCACGCAGTATAATTAATAACTAATTACTGTCGTTGACAGG 160

TTAGACACACCGACAGTGAGCCGACGTACGAATCACGTGAGTGCGTCATATTAATTATTGATTAATGACAGCAACTGTCC

PstI

161 ACACGAGTAACTCGTCTATCTTCTGCAGGCTGCTTACGGTTTCGTCCGTGTTGCAGCCGATCATCAGCACATCTAGGTTT 240

TGTGCTCATTGAGCAGATAGAAGACGTCCGACGAATGCCAAAGCAGGCACAACGTCGGCTAGTAGTCGTGTAGATCCAAA

241 CGTCCGGGTGTGACCGAAAGGTAAGATGGAGAGCCTTGTCCCTGGTTTCAACGAGAAAACACACGTCCAACTCAGTTTGC 320

GCAGGCCCACACTGGCTTTCCATTCTACCTCTCGGAACAGGGACCAAAGTTGCTCTTTTGTGTGCAGGTTGAGTCAAACG

NruIBsiWI

321 CTGTTTTACAGGTTCGCGACGTGCTCGTACGTGGCTTTGGAGACTCCGTGGAGGAGGTCTTATCAGAGGCACGTCAACAT 400

GACAAAATGTCCAAGCGCTGCACGAGCATGCACCGAAACCTCTGAGGCACCTCCTCCAGAATAGTCTCCGTGCAGTTGTA

AclI

401 CTTAAAGATGGCACTTGTGGCTTAGTAGAAGTTGAAAAAGGCGTTTTGCCTCAACTTGAACAGCCCTATGTGTTCATCAA 480

GAATTTCTACCGTGAACACCGAATCATCTTCAACTTTTTCCGCAAAACGGAGTTGAACTTGTCGGGATACACAAGTAGTT

481 ACGTTCGGATGCTCGAACTGCACCTCATGGTCATGTTATGGTTGAGCTGGTAGCAGAACTCGAAGGCATTCAGTACGGTC 560

TGCAAGCCTACGAGCTTGACGTGGAGTACCAGTACAATACCAACTCGACCATCGTCTTGAGCTTCCGTAAGTCATGCCAG

561 GTAGTGGTGAGACACTTGGTGTCCTTGTCCCTCATGTGGGCGAAATACCAGTGGCTTACCGCAAGGTTCTTCTTCGTAAG 640

CATCACCACTCTGTGAACCACAGGAACAGGGAGTACACCCGCTTTATGGTCACCGAATGGCGTTCCAAGAAGAAGCATTC

polyA signal

MscINarI

641 AACGGTAATAAAGGAGCTGGTGGCCATAGTTACGGCGCCGATCTAAAGTCATTTGACTTAGGCGACGAGCTTGGCACTGA 720

TTGCCATTATTTCCTCGACCACCGGTATCAATGCCGCGGCTAGATTTCAGTAAACTGAATCCGCTGCTCGAACCGTGACT

721 TCCTTATGAAGATTTTCAAGAAAACTGGAACACTAAACATAGCAGTGGTGTTACCCGTGAACTCATGCGTGAGCTTAACG 800

AGGAATACTTCTAAAAGTTCTTTTGACCTTGTGATTTGTATCGTCACCACAATGGGCACTTGAGTACGCACTCGAATTGC

801 GAGGGGCATACACTCGCTATGTCGATAACAACTTCTGTGGCCCTGATGGCTACCCTCTTGAGTGCATTAAAGACCTTCTA 880

CTCCCCGTATGTGAGCGATACAGCTATTGTTGAAGACACCGGGACTACCGATGGGAGAACTCACGTAATTTCTGGAAGAT

PmlIHindIII

881 GCACGTGCTGGTAAAGCTTCATGCACTTTGTCCGAACAACTGGACTTTATTGACACTAAGAGGGGTGTATACTGCTGCCG 960

CGTGCACGACCATTTCGAAGTACGTGAAACAGGCTTGTTGACCTGAAATAACTGTGATTCTCCCCACATATGACGACGGC

AclI

961 TGAACATGAGCATGAAATTGCTTGGTACACGGAACGTTCTGAAAAGAGCTATGAATTGCAGACACCTTTTGAAATTAAAT 1040

ACTTGTACTCGTACTTTAACGAACCATGTGCCTTGCAAGACTTTTCTCGATACTTAACGTCTGTGGAAAACTTTAATTTA

1041 TGGCAAAGAAATTTGACACCTTCAATGGGGAATGTCCAAATTTTGTATTTCCCTTAAATTCCATAATCAAGACTATTCAA 1120

ACCGTTTCTTTAAACTGTGGAAGTTACCCCTTACAGGTTTAAAACATAAAGGGAATTTAAGGTATTAGTTCTGATAAGTT

HindIIIEcoRI

1121 CCAAGGGTTGAAAAGAAAAAGCTTGATGGCTTTATGGGTAGAATTCGATCTGTCTATCCAGTTGCGTCACCAAATGAATG 1200

GGTTCCCAACTTTTCTTTTTCGAACTACCGAAATACCCATCTTAAGCTAGACAGATAGGTCAACGCAGTGGTTTACTTAC

BspHI

1201 CAACCAAATGTGCCTTTCAACTCTCATGAAGTGTGATCATTGTGGTGAAACTTCATGGCAGACGGGCGATTTTGTTAAAG 1280

GTTGGTTTACACGGAAAGTTGAGAGTACTTCACACTAGTAACACCACTTTGAAGTACCGTCTGCCCGCTAAAACAATTTC

1281 CCACTTGCGAATTTTGTGGCACTGAGAATTTGACTAAAGAAGGTGCCACTACTTGTGGTTACTTACCCCAAAATGCTGTT 1360

GGTGAACGCTTAAAACACCGTGACTCTTAAACTGATTTCTTCCACGGTGATGAACACCAATGAATGGGGTTTTACGACAA

1361 GTTAAAATTTATTGTCCAGCATGTCACAATTCAGAAGTAGGACCTGAGCATAGTCTTGCCGAATACCATAATGAATCTGG 1440

CAATTTTAAATAACAGGTCGTACAGTGTTAAGTCTTCATCCTGGACTCGTATCAGAACGGCTTATGGTATTACTTAGACC

1441 CTTGAAAACCATTCTTCGTAAGGGTGGTCGCACTATTGCCTTTGGAGGCTGTGTGTTCTCTTATGTTGGTTGCCATAACA 1520

GAACTTTTGGTAAGAAGCATTCCCACCAGCGTGATAACGGAAACCTCCGACACACAAGAGAATACAACCAACGGTATTGT

PmlINheIAfeI

1521 AGTGTGCCTATTGGGTTCCACGTGCTAGCGCTAACATAGGTTGTAACCATACAGGTGTTGTTGGAGAAGGTTCCGAAGGT 1600

TCACACGGATAACCCAAGGTGCACGATCGCGATTGTATCCAACATTGGTATGTCCACAACAACCTCTTCCAAGGCTTCCA

SspIDraI

1601 CTTAATGACAACCTTCTTGAAATACTCCAAAAAGAGAAAGTCAACATCAATATTGTTGGTGACTTTAAACTTAATGAAGA 1680

GAATTACTGTTGGAAGAACTTTATGAGGTTTTTCTCTTTCAGTTGTAGTTATAACAACCACTGAAATTTGAATTACTTCT

PsiI

1681 GATCGCCATTATTTTGGCATCTTTTTCTGCTTCCACAAGTGCTTTTGTGGAAACTGTGAAAGGTTTGGATTATAAAGCAT 1760

CTAGCGGTAATAAAACCGTAGAAAAAGACGAAGGTGTTCACGAAAACACCTTTGACACTTTCCAAACCTAATATTTCGTA

DraISspI

1761 TCAAACAAATTGTTGAATCCTGTGGTAATTTTAAAGTTACAAAAGGAAAAGCTAAAAAAGGTGCCTGGAATATTGGTGAA 1840

AGTTTGTTTAACAACTTAGGACACCATTAAAATTTCAATGTTTTCCTTTTCGATTTTTTCCACGGACCTTATAACCACTT

NsiI

1841 CAGAAATCAATACTGAGTCCTCTTTATGCATTTGCATCAGAGGCTGCTCGTGTTGTACGATCAATTTTCTCCCGCACTCT 1920

GTCTTTAGTTATGACTCAGGAGAAATACGTAAACGTAGTCTCCGACGAGCACAACATGCTAGTTAAAAGAGGGCGTGAGA

1921 TGAAACTGCTCAAAATTCTGTGCGTGTTTTACAGAAGGCCGCTATAACAATACTAGATGGAATTTCACAGTATTCACTGA 2000

ACTTTGACGAGTTTTAAGACACGCACAAAATGTCTTCCGGCGATATTGTTATGATCTACCTTAAAGTGTCATAAGTGACT

2001 GACTCATTGATGCTATGATGTTCACATCTGATTTGGCTACTAACAATCTAGTTGTAATGGCCTACATTACAGGTGGTGTT 2080

CTGAGTAACTACGATACTACAAGTGTAGACTAAACCGATGATTGTTAGATCAACATTACCGGATGTAATGTCCACCACAA

2081 GTTCAGTTGACTTCGCAGTGGCTAACTAACATCTTTGGCACTGTTTATGAAAAACTCAAACCCGTCCTTGATTGGCTTGA 2160

CAAGTCAACTGAAGCGTCACCGATTGATTGTAGAAACCGTGACAAATACTTTTTGAGTTTGGGCAGGAACTAACCGAACT

2161 AGAGAAGTTTAAGGAAGGTGTAGAGTTTCTTAGAGACGGTTGGGAAATTGTTAAATTTATCTCAACCTGTGCTTGTGAAA 2240

TCTCTTCAAATTCCTTCCACATCTCAAAGAATCTCTGCCAACCCTTTAACAATTTAAATAGAGTTGGACACGAACACTTT

polyA signal

HindIII

2241 TTGTCGGTGGACAAATTGTCACCTGTGCAAAGGAAATTAAGGAGAGTGTTCAGACATTCTTTAAGCTTGTAAATAAATTT 2320

AACAGCCACCTGTTTAACAGTGGACACGTTTCCTTTAATTCCTCTCACAAGTCTGTAAGAAATTCGAACATTTATTTAAA

2321 TTGGCTTTGTGTGCTGACTCTATCATTATTGGTGGAGCTAAACTTAAAGCCTTGAATTTAGGTGAAACATTTGTCACGCA 2400

AACCGAAACACACGACTGAGATAGTAATAACCACCTCGATTTGAATTTCGGAACTTAAATCCACTTTGTAAACAGTGCGT

BsrGI

2401 CTCAAAGGGATTGTACAGAAAGTGTGTTAAATCCAGAGAAGAAACTGGCCTACTCATGCCTCTAAAAGCCCCAAAAGAAA 2480

GAGTTTCCCTAACATGTCTTTCACACAATTTAGGTCTCTTCTTTGACCGGATGAGTACGGAGATTTTCGGGGTTTTCTTT

HpaI

2481 TTATCTTCTTAGAGGGAGAAACACTTCCCACAGAAGTGTTAACAGAGGAAGTTGTCTTGAAAACTGGTGATTTACAACCA 2560

AATAGAAGAATCTCCCTCTTTGTGAAGGGTGTCTTCACAATTGTCTCCTTCAACAGAACTTTTGACCACTAAATGTTGGT

SpeI

2561 TTAGAACAACCTACTAGTGAAGCTGTTGAAGCTCCATTGGTTGGTACACCAGTTTGTATTAACGGGCTTATGTTGCTCGA 2640

AATCTTGTTGGATGATCACTTCGACAACTTCGAGGTAACCAACCATGTGGTCAAACATAATTGCCCGAATACAACGAGCT

ScaIApaLI

2641 AATCAAAGACACAGAAAAGTACTGTGCCCTTGCACCTAATATGATGGTAACAAACAATACCTTCACACTCAAAGGCGGTG 2720

TTAGTTTCTGTGTCTTTTCATGACACGGGAACGTGGATTATACTACCATTGTTTGTTATGGAAGTGTGAGTTTCCGCCAC

2721 CACCAACAAAGGTTACTTTTGGTGATGACACTGTGATAGAAGTGCAAGGTTACAAGAGTGTGAATATCACTTTTGAACTT 2800

GTGGTTGTTTCCAATGAAAACCACTACTGTGACACTATCTTCACGTTCCAATGTTCTCACACTTATAGTGAAAACTTGAA

ScaI

2801 GATGAAAGGATTGATAAAGTACTTAATGAGAAGTGCTCTGCCTATACAGTTGAACTCGGTACAGAAGTAAATGAGTTCGC 2880

CTACTTTCCTAACTATTTCATGAATTACTCTTCACGAGACGGATATGTCAACTTGAGCCATGTCTTCATTTACTCAAGCG

2881 CTGTGTTGTGGCAGATGCTGTCATAAAAACTTTGCAACCAGTATCTGAATTACTTACACCACTGGGCATTGATTTAGATG 2960

GACACAACACCGTCTACGACAGTATTTTTGAAACGTTGGTCATAGACTTAATGAATGTGGTGACCCGTAACTAAATCTAC

DraINdeI

2961 AGTGGAGTATGGCTACATACTACTTATTTGATGAGTCTGGTGAGTTTAAATTGGCTTCACATATGTATTGTTCTTTCTAC 3040

TCACCTCATACCGATGTATGATGAATAAACTACTCAGACCACTCAAATTTAACCGAAGTGTATACATAACAAGAAAGATG

3041 CCTCCAGATGAGGATGAAGAAGAAGGTGATTGTGAAGAAGAAGAGTTTGAGCCATCAACTCAATATGAGTATGGTACTGA 3120

GGAGGTCTACTCCTACTTCTTCTTCCACTAACACTTCTTCTTCTCAAACTCGGTAGTTGAGTTATACTCATACCATGACT

3121 AGATGATTACCAAGGTAAACCTTTGGAATTTGGTGCCACTTCTGCTGCTCTTCAACCTGAAGAAGAGCAAGAAGAAGATT 3200

TCTACTAATGGTTCCATTTGGAAACCTTAAACCACGGTGAAGACGACGAGAAGTTGGACTTCTTCTCGTTCTTCTTCTAA

MfeI

3201 GGTTAGATGATGATAGTCAACAAACTGTTGGTCAACAAGACGGCAGTGAGGACAATCAGACAACTACTATTCAAACAATT 3280

CCAATCTACTACTATCAGTTGTTTGACAACCAGTTGTTCTGCCGTCACTCCTGTTAGTCTGTTGATGATAAGTTTGTTAA

DraI

3281 GTTGAGGTTCAACCTCAATTAGAGATGGAACTTACACCAGTTGTTCAGACTATTGAAGTGAATAGTTTTAGTGGTTATTT 3360

CAACTCCAAGTTGGAGTTAATCTCTACCTTGAATGTGGTCAACAAGTCTGATAACTTCACTTATCAAAATCACCAATAAA

3361 AAAACTTACTGACAATGTATACATTAAAAATGCAGACATTGTGGAAGAAGCTAAAAAGGTAAAACCAACAGTGGTTGTTA 3440

TTTTGAATGACTGTTACATATGTAATTTTTACGTCTGTAACACCTTCTTCGATTTTTCCATTTTGGTTGTCACCAACAAT

3441 ATGCAGCCAATGTTTACCTTAAACATGGAGGAGGTGTTGCAGGAGCCTTAAATAAGGCTACTAACAATGCCATGCAAGTT 3520

TACGTCGGTTACAAATGGAATTTGTACCTCCTCCACAACGTCCTCGGAATTTATTCCGATGATTGTTACGGTACGTTCAA

3521 GAATCTGATGATTACATAGCTACTAATGGACCACTTAAAGTGGGTGGTAGTTGTGTTTTAAGCGGACACAATCTTGCTAA 3600

CTTAGACTACTAATGTATCGATGATTACCTGGTGAATTTCACCCACCATCAACACAAAATTCGCCTGTGTTAGAACGATT

HpaIAflII

3601 ACACTGTCTTCATGTTGTCGGCCCAAATGTTAACAAAGGTGAAGACATTCAACTTCTTAAGAGTGCTTATGAAAATTTTA 3680

TGTGACAGAAGTACAACAGCCGGGTTTACAATTGTTTCCACTTCTGTAAGTTGAAGAATTCTCACGAATACTTTTAAAAT

PvuII

3681 ATCAGCACGAAGTTCTACTTGCACCATTATTATCAGCTGGTATTTTTGGTGCTGACCCTATACATTCTTTAAGAGTTTGT 3760

TAGTCGTGCTTCAAGATGAACGTGGTAATAATAGTCGACCATAAAAACCACGACTGGGATATGTAAGAAATTCTCAAACA

HindIII

3761 GTAGATACTGTTCGCACAAATGTCTACTTAGCTGTCTTTGATAAAAATCTCTATGACAAACTTGTTTCAAGCTTTTTGGA 3840

CATCTATGACAAGCGTGTTTACAGATGAATCGACAGAAACTATTTTTAGAGATACTGTTTGAACAAAGTTCGAAAAACCT

PsiI

3841 AATGAAGAGTGAAAAGCAAGTTGAACAAAAGATCGCTGAGATTCCTAAAGAGGAAGTTAAGCCATTTATAACTGAAAGTA 3920

TTACTTCTCACTTTTCGTTCAACTTGTTTTCTAGCGACTCTAAGGATTTCTCCTTCAATTCGGTAAATATTGACTTTCAT

HindIII

3921 AACCTTCAGTTGAACAGAGAAAACAAGATGATAAGAAAATCAAAGCTTGTGTTGAAGAAGTTACAACAACTCTGGAAGAA 4000

TTGGAAGTCAACTTGTCTCTTTTGTTCTACTATTCTTTTAGTTTCGAACACAACTTCTTCAATGTTGTTGAGACCTTCTT

AseI

4001 ACTAAGTTCCTCACAGAAAACTTGTTACTTTATATTGACATTAATGGCAATCTTCATCCAGATTCTGCCACTCTTGTTAG 4080

TGATTCAAGGAGTGTCTTTTGAACAATGAAATATAACTGTAATTACCGTTAGAAGTAGGTCTAAGACGGTGAGAACAATC

4081 TGACATTGACATCACTTTCTTAAAGAAAGATGCTCCATATATAGTGGGTGATGTTGTTCAAGAGGGTGTTTTAACTGCTG 4160

ACTGTAACTGTAGTGAAAGAATTTCTTTCTACGAGGTATATATCACCCACTACAACAAGTTCTCCCACAAAATTGACGAC

NheIHindIII

4161 TGGTTATACCTACTAAAAAGGCTGGTGGCACTACTGAAATGCTAGCGAAAGCTTTGAGAAAAGTGCCAACAGACAATTAT 4240

ACCAATATGGATGATTTTTCCGACCACCGTGATGACTTTACGATCGCTTTCGAAACTCTTTTCACGGTTGTCTGTTAATA

SmaIDraI

4241 ATAACCACTTACCCGGGTCAGGGTTTAAATGGTTACACTGTAGAGGAGGCAAAGACAGTGCTTAAAAAGTGTAAAAGTGC 4320

TATTGGTGAATGGGCCCAGTCCCAAATTTACCAATGTGACATCTCCTCCGTTTCTGTCACGAATTTTTCACATTTTCACG

4321 CTTTTACATTCTACCATCTATTATCTCTAATGAGAAGCAAGAAATTCTTGGAACTGTTTCTTGGAATTTGCGAGAAATGC 4400

GAAAATGTAAGATGGTAGATAATAGAGATTACTCTTCGTTCTTTAAGAACCTTGACAAAGAACCTTAAACGCTCTTTACG

AseI

4401 TTGCACATGCAGAAGAAACACGCAAATTAATGCCTGTCTGTGTGGAAACTAAAGCCATAGTTTCAACTATACAGCGTAAA 4480

AACGTGTACGTCTTCTTTGTGCGTTTAATTACGGACAGACACACCTTTGATTTCGGTATCAAAGTTGATATGTCGCATTT

4481 TATAAGGGTATTAAAATACAAGAGGGTGTGGTTGATTATGGTGCTAGATTTTACTTTTACACCAGTAAAACAACTGTAGC 4560

ATATTCCCATAATTTTATGTTCTCCCACACCAACTAATACCACGATCTAAAATGAAAATGTGGTCATTTTGTTGACATCG

4561 GTCACTTATCAACACACTTAACGATCTAAATGAAACTCTTGTTACAATGCCACTTGGCTATGTAACACATGGCTTAAATT 4640

CAGTGAATAGTTGTGTGAATTGCTAGATTTACTTTGAGAACAATGTTACGGTGAACCGATACATTGTGTACCGAATTTAA

BglII

4641 TGGAAGAAGCTGCTCGGTATATGAGATCTCTCAAAGTGCCAGCTACAGTTTCTGTTTCTTCACCTGATGCTGTTACAGCG 4720

ACCTTCTTCGACGAGCCATATACTCTAGAGAGTTTCACGGTCGATGTCAAAGACAAAGAAGTGGACTACGACAATGTCGC

4721 TATAATGGTTATCTTACTTCTTCTTCTAAAACACCTGAAGAACATTTTATTGAAACCATCTCACTTGCTGGTTCCTATAA 4800

ATATTACCAATAGAATGAAGAAGAAGATTTTGTGGACTTCTTGTAAAATAACTTTGGTAGAGTGAACGACCAAGGATATT

AflIISpeI

4801 AGATTGGTCCTATTCTGGACAATCTACACAACTAGGTATAGAATTTCTTAAGAGAGGTGATAAAAGTGTATATTACACTA 4880

TCTAACCAGGATAAGACCTGTTAGATGTGTTGATCCATATCTTAAAGAATTCTCTCCACTATTTTCACATATAATGTGAT

AflII

4881 GTAATCCTACCACATTCCACCTAGATGGTGAAGTTATCACCTTTGACAATCTTAAGACACTTCTTTCTTTGAGAGAAGTG 4960

CATTAGGATGGTGTAAGGTGGATCTACCACTTCAATAGTGGAAACTGTTAGAATTCTGTGAAGAAAGAAACTCTCTTCAC

NdeI

4961 AGGACTATTAAGGTGTTTACAACAGTAGACAACATTAACCTCCACACGCAAGTTGTGGACATGTCAATGACATATGGACA 5040

TCCTGATAATTCCACAAATGTTGTCATCTGTTGTAATTGGAGGTGTGCGTTCAACACCTGTACAGTTACTGTATACCTGT

polyA signal

5041 ACAGTTTGGTCCAACTTATTTGGATGGAGCTGATGTTACTAAAATAAAACCTCATAATTCACATGAAGGTAAAACATTTT 5120

TGTCAAACCAGGTTGAATAAACCTACCTCGACTACAATGATTTTATTTTGGAGTATTAAGTGTACTTCCATTTTGTAAAA

ScaI

5121 ATGTTTTACCTAATGATGACACTCTACGTGTTGAGGCTTTTGAGTACTACCACACAACTGATCCTAGTTTTCTGGGTAGG 5200

TACAAAATGGATTACTACTGTGAGATGCACAACTCCGAAAACTCATGATGGTGTGTTGACTAGGATCAAAAGACCCATCC

5201 TACATGTCAGCATTAAATCACACTAAAAAGTGGAAATACCCACAAGTTAATGGTTTAACTTCTATTAAATGGGCAGATAA 5280

ATGTACAGTCGTAATTTAGTGTGATTTTTCACCTTTATGGGTGTTCAATTACCAAATTGAAGATAATTTACCCGTCTATT

HpaI

5281 CAACTGTTATCTTGCCACTGCATTGTTAACACTCCAACAAATAGAGTTGAAGTTTAATCCACCTGCTCTACAAGATGCTT 5360

GTTGACAATAGAACGGTGACGTAACAATTGTGAGGTTGTTTATCTCAACTTCAAATTAGGTGGACGAGATGTTCTACGAA

ApaLI

5361 ATTACAGAGCAAGGGCTGGTGAAGCTGCTAACTTTTGTGCACTTATCTTAGCCTACTGTAATAAGACAGTAGGTGAGTTA 5440

TAATGTCTCGTTCCCGACCACTTCGACGATTGAAAACACGTGAATAGAATCGGATGACATTATTCTGTCATCCACTCAAT

5441 GGTGATGTTAGAGAAACAATGAGTTACTTGTTTCAACATGCCAATTTAGATTCTTGCAAAAGAGTCTTGAACGTGGTGTG 5520

CCACTACAATCTCTTTGTTACTCAATGAACAAAGTTGTACGGTTAAATCTAAGAACGTTTTCTCAGAACTTGCACCACAC

AflIIBsrGI

5521 TAAAACTTGTGGACAACAGCAGACAACCCTTAAGGGTGTAGAAGCTGTTATGTACATGGGCACACTTTCTTATGAACAAT 5600

ATTTTGAACACCTGTTGTCGTCTGTTGGGAATTCCCACATCTTCGACAATACATGTACCCGTGTGAAAGAATACTTGTTA

5601 TTAAGAAAGGTGTTCAGATACCTTGTACGTGTGGTAAACAAGCTACAAAATATCTAGTACAACAGGAGTCACCTTTTGTT 5680

AATTCTTTCCACAAGTCTATGGAACATGCACACCATTTGTTCGATGTTTTATAGATCATGTTGTCCTCAGTGGAAAACAA

AflII

5681 ATGATGTCAGCACCACCTGCTCAGTATGAACTTAAGCATGGTACATTTACTTGTGCTAGTGAGTACACTGGTAATTACCA 5760

TACTACAGTCGTGGTGGACGAGTCATACTTGAATTCGTACCATGTAAATGAACACGATCACTCATGTGACCATTAATGGT

5761 GTGTGGTCACTATAAACATATAACTTCTAAAGAAACTTTGTATTGCATAGACGGTGCTTTACTTACAAAGTCCTCAGAAT 5840

CACACCAGTGATATTTGTATATTGAAGATTTCTTTGAAACATAACGTATCTGCCACGAAATGAATGTTTCAGGAGTCTTA

PsiI

5841 ACAAAGGTCCTATTACGGATGTTTTCTACAAAGAAAACAGTTACACAACAACCATAAAACCAGTTACTTATAAATTGGAT 5920

TGTTTCCAGGATAATGCCTACAAAAGATGTTTCTTTTGTCAATGTGTTGTTGGTATTTTGGTCAATGAATATTTAACCTA

BsrGIPsiIMfeI

5921 GGTGTTGTTTGTACAGAAATTGACCCTAAGTTGGACAATTATTATAAGAAAGACAATTCTTATTTCACAGAGCAACCAAT 6000

CCACAACAAACATGTCTTTAACTGGGATTCAACCTGTTAATAATATTCTTTCTGTTAAGAATAAAGTGTCTCGTTGGTTA

HindIII

6001 TGATCTTGTACCAAACCAACCATATCCAAACGCAAGCTTCGATAATTTTAAGTTTGTATGTGATAATATCAAATTTGCTG 6080

ACTAGAACATGGTTTGGTTGGTATAGGTTTGCGTTCGAAGCTATTAAAATTCAAACATACACTATTATAGTTTAAACGAC

DraIHpaIPsiI

6081 ATGATTTAAACCAGTTAACTGGTTATAAGAAACCTGCTTCAAGAGAGCTTAAAGTTACATTTTTCCCTGACTTAAATGGT 6160

TACTAAATTTGGTCAATTGACCAATATTCTTTGGACGAAGTTCTCTCGAATTTCAATGTAAAAAGGGACTGAATTTACCA

PsiI

6161 GATGTGGTGGCTATTGATTATAAACACTACACACCCTCTTTTAAGAAAGGAGCTAAATTGTTACATAAACCTATTGTTTG 6240

CTACACCACCGATAACTAATATTTGTGATGTGTGGGAGAAAATTCTTTCCTCGATTTAACAATGTATTTGGATAACAAAC

polyA signal

HpaI

6241 GCATGTTAACAATGCAACTAATAAAGCCACGTATAAACCAAATACCTGGTGTATACGTTGTCTTTGGAGCACAAAACCAG 6320

CGTACAATTGTTACGTTGATTATTTCGGTGCATATTTGGTTTATGGACCACATATGCAACAGAAACCTCGTGTTTTGGTC

BglII

6321 TTGAAACATCAAATTCGTTTGATGTACTGAAGTCAGAGGACGCGCAGGGAATGGATAATCTTGCCTGCGAAGATCTAAAA 6400

AACTTTGTAGTTTAAGCAAACTACATGACTTCAGTCTCCTGCGCGTCCCTTACCTATTAGAACGGACGCTTCTAGATTTT

6401 CCAGTCTCTGAAGAAGTAGTGGAAAATCCTACCATACAGAAAGACGTTCTTGAGTGTAATGTGAAAACTACCGAAGTTGT 6480

GGTCAGAGACTTCTTCATCACCTTTTAGGATGGTATGTCTTTCTGCAAGAACTCACATTACACTTTTGATGGCTTCAACA

DraIMscIBglII

6481 AGGAGACATTATACTTAAACCAGCAAATAATAGTTTAAAAATTACAGAAGAGGTTGGCCACACAGATCTAATGGCTGCTT 6560

TCCTCTGTAATATGAATTTGGTCGTTTATTATCAAATTTTTAATGTCTTCTCCAACCGGTGTGTCTAGATTACCGACGAA

XbaI

6561 ATGTAGACAATTCTAGTCTTACTATTAAGAAACCTAATGAATTATCTAGAGTATTAGGTTTGAAAACCCTTGCTACTCAT 6640

TACATCTGTTAAGATCAGAATGATAATTCTTTGGATTACTTAATAGATCTCATAATCCAAACTTTTGGGAACGATGAGTA

6641 GGTTTAGCTGCTGTTAATAGTGTCCCTTGGGATACTATAGCTAATTATGCTAAGCCTTTTCTTAACAAAGTTGTTAGTAC 6720

CCAAATCGACGACAATTATCACAGGGAACCCTATGATATCGATTAATACGATTCGGAAAAGAATTGTTTCAACAATCATG

PmeI/DraIMfeI

6721 AACTACTAACATAGTTACACGGTGTTTAAACCGTGTTTGTACTAATTATATGCCTTATTTCTTTACTTTATTGCTACAAT 6800

TTGATGATTGTATCAATGTGCCACAAATTTGGCACAAACATGATTAATATACGGAATAAAGAAATGAAATAACGATGTTA

XbaI

6801 TGTGTACTTTTACTAGAAGTACAAATTCTAGAATTAAAGCATCTATGCCGACTACTATAGCAAAGAATACTGTTAAGAGT 6880

ACACATGAAAATGATCTTCATGTTTAAGATCTTAATTTCGTAGATACGGCTGATGATATCGTTTCTTATGACAATTCTCA

XbaISspIPsiI

6881 GTCGGTAAATTTTGTCTAGAGGCTTCATTTAATTATTTGAAGTCACCTAATTTTTCTAAACTGATAAATATTATAATTTG 6960

CAGCCATTTAAAACAGATCTCCGAAGTAAATTAATAAACTTCAGTGGATTAAAAAGATTTGACTATTTATAATATTAAAC

AvrIISphI

6961 GTTTTTACTATTAAGTGTTTGCCTAGGTTCTTTAATCTACTCAACCGCTGCTTTAGGTGTTTTAATGTCTAATTTAGGCA 7040

CAAAAATGATAATTCACAAACGGATCCAAGAAATTAGATGAGTTGGCGACGAAATCCACAAAATTACAGATTAAATCCGT

7041 TGCCTTCTTACTGTACTGGTTACAGAGAAGGCTATTTGAACTCTACTAATGTCACTATTGCAACCTACTGTACTGGTTCT 7120

ACGGAAGAATGACATGACCAATGTCTCTTCCGATAAACTTGAGATGATTACAGTGATAACGTTGGATGACATGACCAAGA

7121 ATACCTTGTAGTGTTTGTCTTAGTGGTTTAGATTCTTTAGACACCTATCCTTCTTTAGAAACTATACAAATTACCATTTC 7200

TATGGAACATCACAAACAGAATCACCAAATCTAAGAAATCTGTGGATAGGAAGAAATCTTTGATATGTTTAATGGTAAAG

DraI

7201 ATCTTTTAAATGGGATTTAACTGCTTTTGGCTTAGTTGCAGAGTGGTTTTTGGCATATATTCTTTTCACTAGGTTTTTCT 7280

TAGAAAATTTACCCTAAATTGACGAAAACCGAATCAACGTCTCACCAAAAACCGTATATAAGAAAAGTGATCCAAAAAGA

MfeI

7281 ATGTACTTGGATTGGCTGCAATCATGCAATTGTTTTTCAGCTATTTTGCAGTACATTTTATTAGTAATTCTTGGCTTATG 7360

TACATGAACCTAACCGACGTTAGTACGTTAACAAAAAGTCGATAAAACGTCATGTAAAATAATCATTAAGAACCGAATAC

AseIBsrGIBsrGI

7361 TGGTTAATAATTAATCTTGTACAAATGGCCCCGATTTCAGCTATGGTTAGAATGTACATCTTCTTTGCATCATTTTATTA 7440

ACCAATTATTAATTAGAACATGTTTACCGGGGCTAAAGTCGATACCAATCTTACATGTAGAAGAAACGTAGTAAAATAAT

7441 TGTATGGAAAAGTTATGTGCATGTTGTAGACGGTTGTAATTCATCAACTTGTATGATGTGTTACAAACGTAATAGAGCAA 7520

ACATACCTTTTCAATACACGTACAACATCTGCCAACATTAAGTAGTTGAACATACTACACAATGTTTGCATTATCTCGTT

BsrGI

7521 CAAGAGTCGAATGTACAACTATTGTTAATGGTGTTAGAAGGTCCTTTTATGTCTATGCTAATGGAGGTAAAGGCTTTTGC 7600

GTTCTCAGCTTACATGTTGATAACAATTACCACAATCTTCCAGGAAAATACAGATACGATTACCTCCATTTCCGAAAACG

MfeI

7601 AAACTACACAATTGGAATTGTGTTAATTGTGATACATTCTGTGCTGGTAGTACATTTATTAGTGATGAAGTTGCGAGAGA 7680

TTTGATGTGTTAACCTTAACACAATTAACACTATGTAAGACACGACCATCATGTAAATAATCACTACTTCAACGCTCTCT

polyA signal

DraI

7681 CTTGTCACTACAGTTTAAAAGACCAATAAATCCTACTGACCAGTCTTCTTACATCGTTGATAGTGTTACAGTGAAGAATG 7760

GAACAGTGATGTCAAATTTTCTGGTTATTTAGGATGACTGGTCAGAAGAATGTAGCAACTATCACAATGTCACTTCTTAC

HpaI

7761 GTTCCATCCATCTTTACTTTGATAAAGCTGGTCAAAAGACTTATGAAAGACATTCTCTCTCTCATTTTGTTAACTTAGAC 7840

CAAGGTAGGTAGAAATGAAACTATTTCGACCAGTTTTCTGAATACTTTCTGTAAGAGAGAGAGTAAAACAATTGAATCTG

AseI

7841 AACCTGAGAGCTAATAACACTAAAGGTTCATTGCCTATTAATGTTATAGTTTTTGATGGTAAATCAAAATGTGAAGAATC 7920

TTGGACTCTCGATTATTGTGATTTCCAAGTAACGGATAATTACAATATCAAAAACTACCATTTAGTTTTACACTTCTTAG

7921 ATCTGCAAAATCAGCGTCTGTTTACTACAGTCAGCTTATGTGTCAACCTATACTGTTACTAGATCAGGCATTAGTGTCTG 8000

TAGACGTTTTAGTCGCAGACAAATGATGTCAGTCGAATACACAGTTGGATATGACAATGATCTAGTCCGTAATCACAGAC

8001 ATGTTGGTGATAGTGCGGAAGTTGCAGTTAAAATGTTTGATGCTTACGTTAATACGTTTTCATCAACTTTTAACGTACCA 8080

TACAACCACTATCACGCCTTCAACGTCAATTTTACAAACTACGAATGCAATTATGCAAAAGTAGTTGAAAATTGCATGGT

SpeIPstI

8081 ATGGAAAAACTCAAAACACTAGTTGCAACTGCAGAAGCTGAACTTGCAAAGAATGTGTCCTTAGACAATGTCTTATCTAC 8160

TACCTTTTTGAGTTTTGTGATCAACGTTGACGTCTTCGACTTGAACGTTTCTTACACAGGAATCTGTTACAGAATAGATG

8161 TTTTATTTCAGCAGCTCGGCAAGGGTTTGTTGATTCAGATGTAGAAACTAAAGATGTTGTTGAATGTCTTAAATTGTCAC 8240

AAAATAAAGTCGTCGAGCCGTTCCCAAACAACTAAGTCTACATCTTTGATTTCTACAACAACTTACAGAATTTAACAGTG

8241 ATCAATCTGACATAGAAGTTACTGGCGATAGTTGTAATAACTATATGCTCACCTATAACAAAGTTGAAAACATGACACCC 8320

TAGTTAGACTGTATCTTCAATGACCGCTATCAACATTATTGATATACGAGTGGATATTGTTTCAACTTTTGTACTGTGGG

AseIFspI

8321 CGTGACCTTGGTGCTTGTATTGACTGTAGTGCGCGTCATATTAATGCGCAGGTAGCAAAAAGTCACAACATTGCTTTGAT 8400

GCACTGGAACCACGAACATAACTGACATCACGCGCAGTATAATTACGCGTCCATCGTTTTTCAGTGTTGTAACGAAACTA

AclISnaBI

8401 ATGGAACGTTAAAGATTTCATGTCATTGTCTGAACAACTACGAAAACAAATACGTAGTGCTGCTAAAAAGAATAACTTAC 8480

TACCTTGCAATTTCTAAAGTACAGTAACAGACTTGTTGATGCTTTTGTTTATGCATCACGACGATTTTTCTTATTGAATG

AflII

8481 CTTTTAAGTTGACATGTGCAACTACTAGACAAGTTGTTAATGTTGTAACAACAAAGATAGCACTTAAGGGTGGTAAAATT 8560

GAAAATTCAACTGTACACGTTGATGATCTGTTCAACAATTACAACATTGTTGTTTCTATCGTGAATTCCCACCATTTTAA

PacI

8561 GTTAATAATTGGTTGAAGCAGTTAATTAAAGTTACACTTGTGTTCCTTTTTGTTGCTGCTATTTTCTATTTAATAACACC 8640

CAATTATTAACCAACTTCGTCAATTAATTTCAATGTGAACACAAGGAAAAACAACGACGATAAAAGATAAATTATTGTGG

8641 TGTTCATGTCATGTCTAAACATACTGACTTTTCAAGTGAAATCATAGGATACAAGGCTATTGATGGTGGTGTCACTCGTG 8720

ACAAGTACAGTACAGATTTGTATGACTGAAAAGTTCACTTTAGTATCCTATGTTCCGATAACTACCACCACAGTGAGCAC

8721 ACATAGCATCTACAGATACTTGTTTTGCTAACAAACATGCTGATTTTGACACATGGTTTAGCCAGCGTGGTGGTAGTTAT 8800

TGTATCGTAGATGTCTATGAACAAAACGATTGTTTGTACGACTAAAACTGTGTACCAAATCGGTCGCACCACCATCAATA

HindIIIPstI

8801 ACTAATGACAAAGCTTGCCCATTGATTGCTGCAGTCATAACAAGAGAAGTGGGTTTTGTCGTGCCTGGTTTGCCTGGCAC 8880

TGATTACTGTTTCGAACGGGTAACTAACGACGTCAGTATTGTTCTCTTCACCCAAAACAGCACGGACCAAACGGACCGTG

8881 GATATTACGCACAACTAATGGTGACTTTTTGCATTTCTTACCTAGAGTTTTTAGTGCAGTTGGTAACATCTGTTACACAC 8960

CTATAATGCGTGTTGATTACCACTGAAAAACGTAAAGAATGGATCTCAAAAATCACGTCAACCATTGTAGACAATGTGTG

BsrGIDraI

8961 CATCAAAACTTATAGAGTACACTGACTTTGCAACATCAGCTTGTGTTTTGGCTGCTGAATGTACAATTTTTAAAGATGCT 9040

GTAGTTTTGAATATCTCATGTGACTGAAACGTTGTAGTCGAACACAAAACCGACGACTTACATGTTAAAAATTTCTACGA

9041 TCTGGTAAGCCAGTACCATATTGTTATGATACCAATGTACTAGAAGGTTCTGTTGCTTATGAAAGTTTACGCCCTGACAC 9120

AGACCATTCGGTCATGGTATAACAATACTATGGTTACATGATCTTCCAAGACAACGAATACTTTCAAATGCGGGACTGTG

9121 ACGTTATGTGCTCATGGATGGCTCTATTATTCAATTTCCTAACACCTACCTTGAAGGTTCTGTTAGAGTGGTAACAACTT 9200

TGCAATACACGAGTACCTACCGAGATAATAAGTTAAAGGATTGTGGATGGAACTTCCAAGACAATCTCACCATTGTTGAA

ScaISpeI

9201 TTGATTCTGAGTACTGTAGGCACGGCACTTGTGAAAGATCAGAAGCTGGTGTTTGTGTATCTACTAGTGGTAGATGGGTA 9280

AACTAAGACTCATGACATCCGTGCCGTGAACACTTTCTAGTCTTCGACCACAAACACATAGATGATCACCATCTACCCAT

BglII

9281 CTTAACAATGATTATTACAGATCTTTACCAGGAGTTTTCTGTGGTGTAGATGCTGTAAATTTACTTACTAATATGTTTAC 9360

GAATTGTTACTAATAATGTCTAGAAATGGTCCTCAAAAGACACCACATCTACGACATTTAAATGAATGATTATACAAATG

9361 ACCACTAATTCAACCTATTGGTGCTTTGGACATATCAGCATCTATAGTAGCTGGTGGTATTGTAGCTATCGTAGTAACAT 9440

TGGTGATTAAGTTGGATAACCACGAAACCTGTATAGTCGTAGATATCATCGACCACCATAACATCGATAGCATCATTGTA

9441 GCCTTGCCTACTATTTTATGAGGTTTAGAAGAGCTTTTGGTGAATACAGTCATGTAGTTGCCTTTAATACTTTACTATTC 9520

CGGAACGGATGATAAAATACTCCAAATCTTCTCGAAAACCACTTATGTCAGTACATCAACGGAAATTATGAAATGATAAG

9521 CTTATGTCATTCACTGTACTCTGTTTAACACCAGTTTACTCATTCTTACCTGGTGTTTATTCTGTTATTTACTTGTACTT 9600

GAATACAGTAAGTGACATGAGACAAATTGTGGTCAAATGAGTAAGAATGGACCACAAATAAGACAATAAATGAACATGAA

9601 GACATTTTATCTTACTAATGATGTTTCTTTTTTAGCACATATTCAGTGGATGGTTATGTTCACACCTTTAGTACCTTTCT 9680

CTGTAAAATAGAATGATTACTACAAAGAAAAAATCGTGTATAAGTCACCTACCAATACAAGTGTGGAAATCATGGAAAGA

MfeI

9681 GGATAACAATTGCTTATATCATTTGTATTTCCACAAAGCATTTCTATTGGTTCTTTAGTAATTACCTAAAGAGACGTGTA 9760

CCTATTGTTAACGAATATAGTAAACATAAAGGTGTTTCGTAAAGATAACCAAGAAATCATTAATGGATTTCTCTGCACAT

polyA signal

ScaIApaLI

9761 GTCTTTAATGGTGTTTCCTTTAGTACTTTTGAAGAAGCTGCGCTGTGCACCTTTTTGTTAAATAAAGAAATGTATCTAAA 9840

CAGAAATTACCACAAAGGAAATCATGAAAACTTCTTCGACGCGACACGTGGAAAAACAATTTATTTCTTTACATAGATTT

PsiI

9841 GTTGCGTAGTGATGTGCTATTACCTCTTACGCAATATAATAGATACTTAGCTCTTTATAATAAGTACAAGTATTTTAGTG 9920

CAACGCATCACTACACGATAATGGAGAATGCGTTATATTATCTATGAATCGAGAAATATTATTCATGTTCATAAAATCAC

9921 GAGCAATGGATACAACTAGCTACAGAGAAGCTGCTTGTTGTCATCTCGCAAAGGCTCTCAATGACTTCAGTAACTCAGGT 10000

CTCGTTACCTATGTTGATCGATGTCTCTTCGACGAACAACAGTAGAGCGTTTCCGAGAGTTACTGAAGTCATTGAGTCCA

PvuII

10001 TCTGATGTTCTTTACCAACCACCACAAACCTCTATCACCTCAGCTGTTTTGCAGAGTGGTTTTAGAAAAATGGCATTCCC 10080

AGACTACAAGAAATGGTTGGTGGTGTTTGGAGATAGTGGAGTCGACAAAACGTCTCACCAAAATCTTTTTACCGTAAGGG

10081 ATCTGGTAAAGTTGAGGGTTGTATGGTACAAGTAACTTGTGGTACAACTACACTTAACGGTCTTTGGCTTGATGACGTAG 10160

TAGACCATTTCAACTCCCAACATACCATGTTCATTGAACACCATGTTGATGTGAATTGCCAGAAACCGAACTACTGCATC

10161 TTTACTGTCCAAGACATGTGATCTGCACCTCTGAAGACATGCTTAACCCTAATTATGAAGATTTACTCATTCGTAAGTCT 10240

AAATGACAGGTTCTGTACACTAGACGTGGAGACTTCTGTACGAATTGGGATTAATACTTCTAAATGAGTAAGCATTCAGA

AflIIHindIII/AflII

10241 AATCATAATTTCTTGGTACAGGCTGGTAATGTTCAACTCAGGGTTATTGGACATTCTATGCAAAATTGTGTACTTAAGCT 10320

TTAGTATTAAAGAACCATGTCCGACCATTACAAGTTGAGTCCCAATAACCTGTAAGATACGTTTTAACACATGAATTCGA

10321 TAAGGTTGATACAGCCAATCCTAAGACACCTAAGTATAAGTTTGTTCGCATTCAACCAGGACAGACTTTTTCAGTGTTAG 10400

ATTCCAACTATGTCGGTTAGGATTCTGTGGATTCATATTCAAACAAGCGTAAGTTGGTCCTGTCTGAAAAAGTCACAATC

10401 CTTGTTACAATGGTTCACCATCTGGTGTTTACCAATGTGCTATGAGGCCCAATTTCACTATTAAGGGTTCATTCCTTAAT 10480

GAACAATGTTACCAAGTGGTAGACCACAAATGGTTACACGATACTCCGGGTTAAAGTGATAATTCCCAAGTAAGGAATTA

NdeI

10481 GGTTCATGTGGTAGTGTTGGTTTTAACATAGATTATGACTGTGTCTCTTTTTGTTACATGCACCATATGGAATTACCAAC 10560

CCAAGTACACCATCACAACCAAAATTGTATCTAATACTGACACAGAGAAAAACAATGTACGTGGTATACCTTAATGGTTG

PvuII

10561 TGGAGTTCATGCTGGCACAGACTTAGAAGGTAACTTTTATGGACCTTTTGTTGACAGGCAAACAGCACAAGCAGCTGGTA 10640

ACCTCAAGTACGACCGTGTCTGAATCTTCCATTGAAAATACCTGGAAAACAACTGTCCGTTTGTCGTGTTCGTCGACCAT

PsiIClaI

10641 CGGACACAACTATTACAGTTAATGTTTTAGCTTGGTTGTACGCTGCTGTTATAAATGGAGACAGGTGGTTTCTCAATCGA 10720

GCCTGTGTTGATAATGTCAATTACAAAATCGAACCAACATGCGACGACAATATTTACCTCTGTCCACCAAAGAGTTAGCT

10721 TTTACCACAACTCTTAATGACTTTAACCTTGTGGCTATGAAGTACAATTATGAACCTCTAACACAAGACCATGTTGACAT 10800

AAATGGTGTTGAGAATTACTGAAATTGGAACACCGATACTTCATGTTAATACTTGGAGATTGTGTTCTGGTACAACTGTA

10801 ACTAGGACCTCTTTCTGCTCAAACTGGAATTGCCGTTTTAGATATGTGTGCTTCATTAAAAGAATTACTGCAAAATGGTA 10880

TGATCCTGGAGAAAGACGAGTTTGACCTTAACGGCAAAATCTATACACACGAAGTAATTTTCTTAATGACGTTTTACCAT

10881 TGAATGGACGTACCATATTGGGTAGTGCTTTATTAGAAGATGAATTTACACCTTTTGATGTTGTTAGACAATGCTCAGGT 10960

ACTTACCTGCATGGTATAACCCATCACGAAATAATCTTCTACTTAAATGTGGAAAACTACAACAATCTGTTACGAGTCCA

10961 GTTACTTTCCAAAGTGCAGTGAAAAGAACAATCAAGGGTACACACCACTGGTTGTTACTCACAATTTTGACTTCACTTTT 11040

CAATGAAAGGTTTCACGTCACTTTTCTTGTTAGTTCCCATGTGTGGTGACCAACAATGAGTGTTAAAACTGAAGTGAAAA

ScaI

11041 AGTTTTAGTCCAGAGTACTCAATGGTCTTTGTTCTTTTTTTTGTATGAAAATGCCTTTTTACCTTTTGCTATGGGTATTA 11120

TCAAAATCAGGTCTCATGAGTTACCAGAAACAAGAAAAAAAACATACTTTTACGGAAAAATGGAAAACGATACCCATAAT

SphI/NsiI

11121 TTGCTATGTCTGCTTTTGCAATGATGTTTGTCAAACATAAGCATGCATTTCTCTGTTTGTTTTTGTTACCTTCTCTTGCC 11200

AACGATACAGACGAAAACGTTACTACAAACAGTTTGTATTCGTACGTAAAGAGACAAACAAAAACAATGGAAGAGAACGG

SpeI

11201 ACTGTAGCTTATTTTAATATGGTCTATATGCCTGCTAGTTGGGTGATGCGTATTATGACATGGTTGGATATGGTTGATAC 11280

TGACATCGAATAAAATTATACCAGATATACGGACGATCAACCCACTACGCATAATACTGTACCAACCTATACCAACTATG

NsiIPvuII

11281 TAGTTTGTCTGGTTTTAAGCTAAAAGACTGTGTTATGTATGCATCAGCTGTAGTGTTACTAATCCTTATGACAGCAAGAA 11360

ATCAAACAGACCAAAATTCGATTTTCTGACACAATACATACGTAGTCGACATCACAATGATTAGGAATACTGTCGTTCTT

PsiI

11361 CTGTGTATGATGATGGTGCTAGGAGAGTGTGGACACTTATGAATGTCTTGACACTCGTTTATAAAGTTTATTATGGTAAT 11440

GACACATACTACTACCACGATCCTCTCACACCTGTGAATACTTACAGAACTGTGAGCAAATATTTCAAATAATACCATTA

PsiI

11441 GCTTTAGATCAAGCCATTTCCATGTGGGCTCTTATAATCTCTGTTACTTCTAACTACTCAGGTGTAGTTACAACTGTCAT 11520

CGAAATCTAGTTCGGTAAAGGTACACCCGAGAATATTAGAGACAATGAAGATTGATGAGTCCACATCAATGTTGACAGTA

MscI

11521 GTTTTTGGCCAGAGGTATTGTTTTTATGTGTGTTGAGTATTGCCCTATTTTCTTCATAACTGGTAATACACTTCAGTGTA 11600

CAAAAACCGGTCTCCATAACAAAAATACACACAACTCATAACGGGATAAAAGAAGTATTGACCATTATGTGAAGTCACAT

11601 TAATGCTAGTTTATTGTTTCTTAGGCTATTTTTGTACTTGTTACTTTGGCCTCTTTTGTTTACTCAACCGCTACTTTAGA 11680

ATTACGATCAAATAACAAAGAATCCGATAAAAACATGAACAATGAAACCGGAGAAAACAAATGAGTTGGCGATGAAATCT

EcoRI

11681 CTGACTCTTGGTGTTTATGATTACTTAGTTTCTACACAGGAGTTTAGATATATGAATTCACAGGGACTACTCCCACCCAA 11760

GACTGAGAACCACAAATACTAATGAATCAAAGATGTGTCCTCAAATCTATATACTTAAGTGTCCCTGATGAGGGTGGGTT

BsrGI

11761 GAATAGCATAGATGCCTTCAAACTCAACATTAAATTGTTGGGTGTTGGTGGCAAACCTTGTATCAAAGTAGCCACTGTAC 11840

CTTATCGTATCTACGGAAGTTTGAGTTGTAATTTAACAACCCACAACCACCGTTTGGAACATAGTTTCATCGGTGACATG

ApaLI

11841 AGTCTAAAATGTCAGATGTAAAGTGCACATCAGTAGTCTTACTCTCAGTTTTGCAACAACTCAGAGTAGAATCATCATCT 11920

TCAGATTTTACAGTCTACATTTCACGTGTAGTCATCAGAATGAGAGTCAAAACGTTGTTGAGTCTCATCTTAGTAGTAGA

11921 AAATTGTGGGCTCAATGTGTCCAGTTACACAATGACATTCTCTTAGCTAAAGATACTACTGAAGCCTTTGAAAAAATGGT 12000

TTTAACACCCGAGTTACACAGGTCAATGTGTTACTGTAAGAGAATCGATTTCTATGATGACTTCGGAAACTTTTTTACCA

HindIII

12001 TTCACTACTTTCTGTTTTGCTTTCCATGCAGGGTGCTGTAGACATAAACAAGCTTTGTGAAGAAATGCTGGACAACAGGG 12080

AAGTGATGAAAGACAAAACGAAAGGTACGTCCCACGACATCTGTATTTGTTCGAAACACTTCTTTACGACCTGTTGTCCC

NdeIHindIII

12081 CAACCTTACAAGCTATAGCCTCAGAGTTTAGTTCCCTTCCATCATATGCAGCTTTTGCTACTGCTCAAGAAGCTTATGAG 12160

GTTGGAATGTTCGATATCGGAGTCTCAAATCAAGGGAAGGTAGTATACGTCGAAAACGATGACGAGTTCTTCGAATACTC

12161 CAGGCTGTTGCTAATGGTGATTCTGAAGTTGTTCTTAAAAAGTTGAAGAAGTCTTTGAATGTGGCTAAATCTGAATTTGA 12240

GTCCGACAACGATTACCACTAAGACTTCAACAAGAATTTTTCAACTTCTTCAGAAACTTACACCGATTTAGACTTAAACT

BglII

12241 CCGTGATGCAGCCATGCAACGTAAGTTGGAAAAGATGGCTGATCAAGCTATGACCCAAATGTATAAACAGGCTAGATCTG 12320

GGCACTACGTCGGTACGTTGCATTCAACCTTTTCTACCGACTAGTTCGATACTGGGTTTACATATTTGTCCGATCTAGAC

SpeI

12321 AGGACAAGAGGGCAAAAGTTACTAGTGCTATGCAGACAATGCTTTTCACTATGCTTAGAAAGTTGGATAATGATGCACTC 12400

TCCTGTTCTCCCGTTTTCAATGATCACGATACGTCTGTTACGAAAAGTGATACGAATCTTTCAACCTATTACTACGTGAG

12401 AACAACATTATCAACAATGCAAGAGATGGTTGTGTTCCCTTGAACATAATACCTCTTACAACAGCAGCCAAACTAATGGT 12480

TTGTTGTAATAGTTGTTACGTTCTCTACCAACACAAGGGAACTTGTATTATGGAGAATGTTGTCGTCGGTTTGATTACCA

NsiI

12481 TGTCATACCAGACTATAACACATATAAAAATACGTGTGATGGTACAACATTTACTTATGCATCAGCATTGTGGGAAATCC 12560

ACAGTATGGTCTGATATTGTGTATATTTTTATGCACACTACCATGTTGTAAATGAATACGTAGTCGTAACACCCTTTAGG

12561 AACAGGTTGTAGATGCAGATAGTAAAATTGTTCAACTTAGTGAAATTAGTATGGACAATTCACCTAATTTAGCATGGCCT 12640

TTGTCCAACATCTACGTCTATCATTTTAACAAGTTGAATCACTTTAATCATACCTGTTAAGTGGATTAAATCGTACCGGA

12641 CTTATTGTAACAGCTTTAAGGGCCAATTCTGCTGTCAAATTACAGAATAATGAGCTTAGTCCTGTTGCACTACGACAGAT 12720

GAATAACATTGTCGAAATTCCCGGTTAAGACGACAGTTTAATGTCTTATTACTCGAATCAGGACAACGTGATGCTGTCTA

12721 GTCTTGTGCTGCCGGTACTACACAAACTGCTTGCACTGATGACAATGCGTTAGCTTACTACAACACAACAAAGGGAGGTA 12800

CAGAACACGACGGCCATGATGTGTTTGACGAACGTGACTACTGTTACGCAATCGAATGATGTTGTGTTGTTTCCCTCCAT

12801 GGTTTGTACTTGCACTGTTATCCGATTTACAGGATTTGAAATGGGCTAGATTCCCTAAGAGTGATGGAACTGGTACTATC 12880

CCAAACATGAACGTGACAATAGGCTAAATGTCCTAAACTTTACCCGATCTAAGGGATTCTCACTACCTTGACCATGATAG

12881 TATACAGAACTGGAACCACCTTGTAGGTTTGTTACAGACACACCTAAAGGTCCTAAAGTGAAGTATTTATACTTTATTAA 12960

ATATGTCTTGACCTTGGTGGAACATCCAAACAATGTCTGTGTGGATTTCCAGGATTTCACTTCATAAATATGAAATAATT

12961 AGGATTAAACAACCTAAATAGAGGTATGGTACTTGGTAGTTTAGCTGCCACAGTACGTCTACAAGCTGGTAATGCAACAG 13040

TCCTAATTTGTTGGATTTATCTCCATACCATGAACCATCAAATCGACGGTGTCATGCAGATGTTCGACCATTACGTTGTC

HindIII

13041 AAGTGCCTGCCAATTCAACTGTATTATCTTTCTGTGCTTTTGCTGTAGATGCTGCTAAAGCTTACAAAGATTATCTAGCT 13120

TTCACGGACGGTTAAGTTGACATAATAGAAAGACACGAAAACGACATCTACGACGATTTCGAATGTTTCTAATAGATCGA

BsrGI

13121 AGTGGGGGACAACCAATCACTAATTGTGTTAAGATGTTGTGTACACACACTGGTACTGGTCAGGCAATAACAGTTACACC 13200

TCACCCCCTGTTGGTTAGTGATTAACACAATTCTACAACACATGTGTGTGACCATGACCAGTCCGTTATTGTCAATGTGG

13201 GGAAGCCAATATGGATCAAGAATCCTTTGGTGGTGCATCGTGTTGTCTGTACTGCCGTTGCCACATAGATCATCCAAATC 13280

CCTTCGGTTATACCTAGTTCTTAGGAAACCACCACGTAGCACAACAGACATGACGGCAACGGTGTATCTAGTAGGTTTAG

BsrGI

13281 CTAAAGGATTTTGTGACTTAAAAGGTAAGTATGTACAAATACCTACAACTTGTGCTAATGACCCTGTGGGTTTTACACTT 13360

GATTTCCTAAAACACTGAATTTTCCATTCATACATGTTTATGGATGTTGAACACGATTACTGGGACACCCAAAATGTGAA

13361 AAAAACACAGTCTGTACCGTCTGCGGTATGTGGAAAGGTTATGGCTGTAGTTGTGATCAACTCCGCGAACCCATGCTTCA 13440

TTTTTGTGTCAGACATGGCAGACGCCATACACCTTTCCAATACCGACATCAACACTAGTTGAGGCGCTTGGGTACGAAGT

PvuIIDraISpeIScaI

13441 GTCAGCTGATGCACAATCGTTTTTAAACGGGTTTGCGGTGTAAGTGCAGCCCGTCTTACACCGTGCGGCACAGGCACTAG 13520

CAGTCGACTACGTGTTAGCAAAAATTTGCCCAAACGCCACATTCACGTCGGGCAGAATGTGGCACGCCGTGTCCGTGATC

13521 TACTGATGTCGTATACAGGGCTTTTGACATCTACAATGATAAAGTAGCTGGTTTTGCTAAATTCCTAAAAACTAATTGTT 13600

ATGACTACAGCATATGTCCCGAAAACTGTAGATGTTACTATTTCATCGACCAAAACGATTTAAGGATTTTTGATTAACAA

13601 GTCGCTTCCAAGAAAAGGACGAAGATGACAATTTAATTGATTCTTACTTTGTAGTTAAGAGACACACTTTCTCTAACTAC 13680

CAGCGAAGGTTCTTTTCCTGCTTCTACTGTTAAATTAACTAAGAATGAAACATCAATTCTCTGTGTGAAAGAGATTGATG

PsiIAflIIPvuII

13681 CAACATGAAGAAACAATTTATAATTTACTTAAGGATTGTCCAGCTGTTGCTAAACATGACTTCTTTAAGTTTAGAATAGA 13760

GTTGTACTTCTTTGTTAAATATTAAATGAATTCCTAACAGGTCGACAACGATTTGTACTGAAGAAATTCAAATCTTATCT

KpnI

13761 CGGTGACATGGTACCACATATATCACGTCAACGTCTTACTAAATACACAATGGCAGACCTCGTCTATGCTTTAAGGCATT 13840

GCCACTGTACCATGGTGTATATAGTGCAGTTGCAGAATGATTTATGTGTTACCGTCTGGAGCAGATACGAAATTCCGTAA

polyA signal

MfeI

13841 TTGATGAAGGTAATTGTGACACATTAAAAGAAATACTTGTCACATACAATTGTTGTGATGATGATTATTTCAATAAAAAG 13920

AACTACTTCCATTAACACTGTGTAATTTTCTTTATGAACAGTGTATGTTAACAACACTACTACTAATAAAGTTATTTTTC

MluIHindIII

13921 GACTGGTATGATTTTGTAGAAAACCCAGATATATTACGCGTATACGCCAACTTAGGTGAACGTGTACGCCAAGCTTTGTT 14000

CTGACCATACTAAAACATCTTTTGGGTCTATATAATGCGCATATGCGGTTGAATCCACTTGCACATGCGGTTCGAAACAA

BglII

14001 AAAAACAGTACAATTCTGTGATGCCATGCGAAATGCTGGTATTGTTGGTGTACTGACATTAGATAATCAAGATCTCAATG 14080

TTTTTGTCATGTTAAGACACTACGGTACGCTTTACGACCATAACAACCACATGACTGTAATCTATTAGTTCTAGAGTTAC

14081 GTAACTGGTATGATTTCGGTGATTTCATACAAACCACGCCAGGTAGTGGAGTTCCTGTTGTAGATTCTTATTATTCATTG 14160

CATTGACCATACTAAAGCCACTAAAGTATGTTTGGTGCGGTCCATCACCTCAAGGACAACATCTAAGAATAATAAGTAAC

PstI

14161 TTAATGCCTATATTAACCTTGACCAGGGCTTTAACTGCAGAGTCACATGTTGACACTGACTTAACAAAGCCTTACATTAA 14240

AATTACGGATATAATTGGAACTGGTCCCGAAATTGACGTCTCAGTGTACAACTGTGACTGAATTGTTTCGGAATGTAATT

DraISspI

14241 GTGGGATTTGTTAAAATATGACTTCACGGAAGAGAGGTTAAAACTCTTTGACCGTTATTTTAAATATTGGGATCAGACAT 14320

CACCCTAAACAATTTTATACTGAAGTGCCTTCTCTCCAATTTTGAGAAACTGGCAATAAAATTTATAACCCTAGTCTGTA

HpaINsiI

14321 ACCACCCAAATTGTGTTAACTGTTTGGATGACAGATGCATTCTGCATTGTGCAAACTTTAATGTTTTATTCTCTACAGTG 14400

TGGTGGGTTTAACACAATTGACAAACCTACTGTCTACGTAAGACGTAACACGTTTGAAATTACAAAATAAGAGATGTCAC

SpeISspI

14401 TTCCCACCTACAAGTTTTGGACCACTAGTGAGAAAAATATTTGTTGATGGTGTTCCATTTGTAGTTTCAACTGGATACCA 14480

AAGGGTGGATGTTCAAAACCTGGTGATCACTCTTTTTATAAACAACTACCACAAGGTAAACATCAAAGTTGACCTATGGT

BsrGIXbaI

14481 CTTCAGAGAGCTAGGTGTTGTACATAATCAGGATGTAAACTTACATAGCTCTAGACTTAGTTTTAAGGAATTACTTGTGT 14560

GAAGTCTCTCGATCCACAACATGTATTAGTCCTACATTTGAATGTATCGAGATCTGAATCAAAATTCCTTAATGAACACA

14561 ATGCTGCTGACCCTGCTATGCACGCTGCTTCTGGTAATCTATTACTAGATAAACGCACTACGTGCTTTTCAGTAGCTGCA 14640

TACGACGACTGGGACGATACGTGCGACGAAGACCATTAGATAATGATCTATTTGCGTGATGCACGAAAAGTCATCGACGT

14641 CTTACTAACAATGTTGCTTTTCAAACTGTCAAACCCGGTAATTTTAACAAAGACTTCTATGACTTTGCTGTGTCTAAGGG 14720

GAATGATTGTTACAACGAAAAGTTTGACAGTTTGGGCCATTAAAATTGTTTCTGAAGATACTGAAACGACACAGATTCCC

14721 TTTCTTTAAGGAAGGAAGTTCTGTTGAATTAAAACACTTCTTCTTTGCTCAGGATGGTAATGCTGCTATCAGCGATTATG 14800

AAAGAAATTCCTTCCTTCAAGACAACTTAATTTTGTGAAGAAGAAACGAGTCCTACCATTACGACGATAGTCGCTAATAC

PsiIEcoRVScaI

14801 ACTACTATCGTTATAATCTACCAACAATGTGTGATATCAGACAACTACTATTTGTAGTTGAAGTTGTTGATAAGTACTTT 14880

TGATGATAGCAATATTAGATGGTTGTTACACACTATAGTCTGTTGATGATAAACATCAACTTCAACAACTATTCATGAAA

polyA signal

AseIPvuII

14881 GATTGTTACGATGGTGGCTGTATTAATGCTAACCAAGTCATCGTCAACAACCTAGACAAATCAGCTGGTTTTCCATTTAA 14960

CTAACAATGCTACCACCGACATAATTACGATTGGTTCAGTAGCAGTTGTTGGATCTGTTTAGTCGACCAAAAGGTAAATT

14961 TAAATGGGGTAAGGCTAGACTTTATTATGATTCAATGAGTTATGAGGATCAAGATGCACTTTTCGCATATACAAAACGTA 15040

ATTTACCCCATTCCGATCTGAAATAATACTAAGTTACTCAATACTCCTAGTTCTACGTGAAAAGCGTATATGTTTTGCAT

AflIISacI

15041 ATGTCATCCCTACTATAACTCAAATGAATCTTAAGTATGCCATTAGTGCAAAGAATAGAGCTCGCACCGTAGCTGGTGTC 15120

TACAGTAGGGATGATATTGAGTTTACTTAGAATTCATACGGTAATCACGTTTCTTATCTCGAGCGTGGCATCGACCACAG

ScaI

15121 TCTATCTGTAGTACTATGACCAATAGACAGTTTCATCAAAAATTATTGAAATCAATAGCCGCCACTAGAGGAGCTACTGT 15200

AGATAGACATCATGATACTGGTTATCTGTCAAAGTAGTTTTTAATAACTTTAGTTATCGGCGGTGATCTCCTCGATGACA

15201 AGTAATTGGAACAAGCAAATTCTATGGTGGTTGGCACAACATGTTAAAAACTGTTTATAGTGATGTAGAAAACCCTCACC 15280

TCATTAACCTTGTTCGTTTAAGATACCACCAACCGTGTTGTACAATTTTTGACAAATATCACTACATCTTTTGGGAGTGG

15281 TTATGGGTTGGGATTATCCTAAATGTGATAGAGCCATGCCTAACATGCTTAGAATTATGGCCTCACTTGTTCTTGCTCGC 15360

AATACCCAACCCTAATAGGATTTACACTATCTCGGTACGGATTGTACGAATCTTAATACCGGAGTGAACAAGAACGAGCG

15361 AAACATACAACGTGTTGTAGCTTGTCACACCGTTTCTATAGATTAGCTAATGAGTGTGCTCAAGTATTGAGTGAAATGGT 15440

TTTGTATGTTGCACAACATCGAACAGTGTGGCAAAGATATCTAATCGATTACTCACACGAGTTCATAACTCACTTTACCA

15441 CATGTGTGGCGGTTCACTATATGTTAAACCAGGTGGAACCTCATCAGGAGATGCCACAACTGCTTATGCTAATAGTGTTT 15520

GTACACACCGCCAAGTGATATACAATTTGGTCCACCTTGGAGTAGTCCTCTACGGTGTTGACGAATACGATTATCACAAA

15521 TTAACATTTGTCAAGCTGTCACGGCCAATGTTAATGCACTTTTATCTACTGATGGTAACAAAATTGCCGATAAGTATGTC 15600

AATTGTAAACAGTTCGACAGTGCCGGTTACAATTACGTGAAAATAGATGACTACCATTGTTTTAACGGCTATTCATACAG

15601 CGCAATTTACAACACAGACTTTATGAGTGTCTCTATAGAAATAGAGATGTTGACACAGACTTTGTGAATGAGTTTTACGC 15680

GCGTTAAATGTTGTGTCTGAAATACTCACAGAGATATCTTTATCTCTACAACTGTGTCTGAAACACTTACTCAAAATGCG

NsiI

15681 ATATTTGCGTAAACATTTCTCAATGATGATACTCTCTGACGATGCTGTTGTGTGTTTCAATAGCACTTATGCATCTCAAG 15760

TATAAACGCATTTGTAAAGAGTTACTACTATGAGAGACTGCTACGACAACACACAAAGTTATCGTGAATACGTAGAGTTC

NheI

15761 GTCTAGTGGCTAGCATAAAGAACTTTAAGTCAGTTCTTTATTATCAAAACAATGTTTTTATGTCTGAAGCAAAATGTTGG 15840

CAGATCACCGATCGTATTTCTTGAAATTCAGTCAAGAAATAATAGTTTTGTTACAAAAATACAGACTTCGTTTTACAACC

BspHI

15841 ACTGAGACTGACCTTACTAAAGGACCTCATGAATTTTGCTCTCAACATACAATGCTAGTTAAACAGGGTGATGATTATGT 15920

TGACTCTGACTGGAATGATTTCCTGGAGTACTTAAAACGAGAGTTGTATGTTACGATCAATTTGTCCCACTACTAATACA

AvrIINaeIEcoRV

15921 GTACCTTCCTTACCCAGATCCATCAAGAATCCTAGGGGCCGGCTGTTTTGTAGATGATATCGTAAAAACAGATGGTACAC 16000

CATGGAAGGAATGGGTCTAGGTAGTTCTTAGGATCCCCGGCCGACAAAACATCTACTATAGCATTTTTGTCTACCATGTG

16001 TTATGATTGAACGGTTCGTGTCTTTAGCTATAGATGCTTACCCACTTACTAAACATCCTAATCAGGAGTATGCTGATGTC 16080

AATACTAACTTGCCAAGCACAGAAATCGATATCTACGAATGGGTGAATGATTTGTAGGATTAGTCCTCATACGACTACAG

HpaI

16081 TTTCATTTGTACTTACAATACATAAGAAAGCTACATGATGAGTTAACAGGACACATGTTAGACATGTATTCTGTTATGCT 16160

AAAGTAAACATGAATGTTATGTATTCTTTCGATGTACTACTCAATTGTCCTGTGTACAATCTGTACATAAGACAATACGA

BsrGI

16161 TACTAATGATAACACTTCAAGGTATTGGGAACCTGAGTTTTATGAGGCTATGTACACACCGCATACAGTCTTACAGGCTG 16240

ATGATTACTATTGTGAAGTTCCATAACCCTTGGACTCAAAATACTCCGATACATGTGTGGCGTATGTCAGAATGTCCGAC

SnaBI

16241 TTGGGGCTTGTGTTCTTTGCAATTCACAGACTTCATTAAGATGTGGTGCTTGCATACGTAGACCATTCTTATGTTGTAAA 16320

AACCCCGAACACAAGAAACGTTAAGTGTCTGAAGTAATTCTACACCACGAACGTATGCATCTGGTAAGAATACAACATTT

16321 TGCTGTTACGACCATGTCATATCAACATCACATAAATTAGTCTTGTCTGTTAATCCGTATGTTTGCAATGCTCCAGGTTG 16400

ACGACAATGCTGGTACAGTATAGTTGTAGTGTATTTAATCAGAACAGACAATTAGGCATACAAACGTTACGAGGTCCAAC

16401 TGATGTCACAGATGTGACTCAACTTTACTTAGGAGGTATGAGCTATTATTGTAAATCACATAAACCACCCATTAGTTTTC 16480

ACTACAGTGTCTACACTGAGTTGAAATGAATCCTCCATACTCGATAATAACATTTAGTGTATTTGGTGGGTAATCAAAAG

MfeI

16481 CATTGTGTGCTAATGGACAAGTTTTTGGTTTATATAAAAATACATGTGTTGGTAGCGATAATGTTACTGACTTTAATGCA 16560

GTAACACACGATTACCTGTTCAAAAACCAAATATATTTTTATGTACACAACCATCGCTATTACAATGACTGAAATTACGT

HindIII

16561 ATTGCAACATGTGACTGGACAAATGCTGGTGATTACATTTTAGCTAACACCTGTACTGAAAGACTCAAGCTTTTTGCAGC 16640

TAACGTTGTACACTGACCTGTTTACGACCACTAATGTAAAATCGATTGTGGACATGACTTTCTGAGTTCGAAAAACGTCG

DraI

16641 AGAAACGCTCAAAGCTACTGAGGAGACATTTAAACTGTCTTATGGTATTGCTACTGTACGTGAAGTGCTGTCTGACAGAG 16720

TCTTTGCGAGTTTCGATGACTCCTCTGTAAATTTGACAGAATACCATAACGATGACATGCACTTCACGACAGACTGTCTC

16721 AATTACATCTTTCATGGGAAGTTGGTAAACCTAGACCACCACTTAACCGAAATTATGTCTTTACTGGTTATCGTGTAACT 16800

TTAATGTAGAAAGTACCCTTCAACCATTTGGATCTGGTGGTGAATTGGCTTTAATACAGAAATGACCAATAGCACATTGA

16801 AAAAACAGTAAAGTACAAATAGGAGAGTACACCTTTGAAAAAGGTGACTATGGTGATGCTGTTGTTTACCGAGGTACAAC 16880

TTTTTGTCATTTCATGTTTATCCTCTCATGTGGAAACTTTTTCCACTGATACCACTACGACAACAAATGGCTCCATGTTG

ApaLISpeI

16881 AACTTACAAATTAAATGTTGGTGATTATTTTGTGCTGACATCACATACAGTAATGCCATTAAGTGCACCTACACTAGTGC 16960

TTGAATGTTTAATTTACAACCACTAATAAAACACGACTGTAGTGTATGTCATTACGGTAATTCACGTGGATGTGATCACG

16961 CACAAGAGCACTATGTTAGAATTACTGGCTTATACCCAACACTCAATATCTCAGATGAGTTTTCTAGCAATGTTGCAAAT 17040

GTGTTCTCGTGATACAATCTTAATGACCGAATATGGGTTGTGAGTTATAGAGTCTACTCAAAAGATCGTTACAACGTTTA

17041 TATCAAAAGGTTGGTATGCAAAAGTATTCTACACTCCAGGGACCACCTGGTACTGGTAAGAGTCATTTTGCTATTGGCCT 17120

ATAGTTTTCCAACCATACGTTTTCATAAGATGTGAGGTCCCTGGTGGACCATGACCATTCTCAGTAAAACGATAACCGGA

17121 AGCTCTCTACTACCCTTCTGCTCGCATAGTGTATACAGCTTGCTCTCATGCCGCTGTTGATGCACTATGTGAGAAGGCAT 17200

TCGAGAGATGATGGGAAGACGAGCGTATCACATATGTCGAACGAGAGTACGGCGACAACTACGTGATACACTCTTCCGTA

SspIPmlIEcoRI

17201 TAAAATATTTGCCTATAGATAAATGTAGTAGAATTATACCTGCACGTGCTCGTGTAGAGTGTTTTGATAAATTCAAAGTG 17280

ATTTTATAAACGGATATCTATTTACATCATCTTAATATGGACGTGCACGAGCACATCTCACAAAACTATTTAAGTTTCAC

NsiI

17281 AATTCAACATTAGAACAGTATGTCTTTTGTACTGTAAATGCATTGCCTGAGACGACAGCAGATATAGTTGTCTTTGATGA 17360

TTAAGTTGTAATCTTGTCATACAGAAAACATGACATTTACGTAACGGACTCTGCTGTCGTCTATATCAACAGAAACTACT

MscIBsrGI

17361 AATTTCAATGGCCACAAATTATGATTTGAGTGTTGTCAATGCCAGATTACGTGCTAAGCACTATGTGTACATTGGCGACC 17440

TTAAAGTTACCGGTGTTTAATACTAAACTCACAACAGTTACGGTCTAATGCACGATTCGTGATACACATGTAACCGCTGG

SspI

17441 CTGCTCAATTACCTGCACCACGCACATTGCTAACTAAGGGCACACTAGAACCAGAATATTTCAATTCAGTGTGTAGACTT 17520

GACGAGTTAATGGACGTGGTGCGTGTAACGATTGATTCCCGTGTGATCTTGGTCTTATAAAGTTAAGTCACACATCTGAA

17521 ATGAAAACTATAGGTCCAGACATGTTCCTCGGAACTTGTCGGCGTTGTCCTGCTGAAATTGTTGACACTGTGAGTGCTTT 17600

TACTTTTGATATCCAGGTCTGTACAAGGAGCCTTGAACAGCCGCAACAGGACGACTTTAACAACTGTGACACTCACGAAA

HindIIIDraIPsiI

17601 GGTTTATGATAATAAGCTTAAAGCACATAAAGACAAATCAGCTCAATGCTTTAAAATGTTTTATAAGGGTGTTATCACGC 17680

CCAAATACTATTATTCGAATTTCGTGTATTTCTGTTTAGTCGAGTTACGAAATTTTACAAAATATTCCCACAATAGTGCG

EcoRI

17681 ATGATGTTTCATCTGCAATTAACAGGCCACAAATAGGCGTGGTAAGAGAATTCCTTACACGTAACCCTGCTTGGAGAAAA 17760

TACTACAAAGTAGACGTTAATTGTCCGGTGTTTATCCGCACCATTCTCTTAAGGAATGTGCATTGGGACGAACCTCTTTT

PsiI

17761 GCTGTCTTTATTTCACCTTATAATTCACAGAATGCTGTAGCCTCAAAGATTTTGGGACTACCAACTCAAACTGTTGATTC 17840

CGACAGAAATAAAGTGGAATATTAAGTGTCTTACGACATCGGAGTTTCTAAAACCCTGATGGTTGAGTTTGACAACTAAG

17841 ATCACAGGGCTCAGAATATGACTATGTCATATTCACTCAAACCACTGAAACAGCTCACTCTTGTAATGTAAACAGATTTA 17920

TAGTGTCCCGAGTCTTATACTGATACAGTATAAGTGAGTTTGGTGACTTTGTCGAGTGAGAACATTACATTTGTCTAAAT

17921 ATGTTGCTATTACCAGAGCAAAAGTAGGCATACTTTGCATAATGTCTGATAGAGACCTTTATGACAAGTTGCAATTTACA 18000

TACAACGATAATGGTCTCGTTTTCATCCGTATGAAACGTATTACAGACTATCTCTGGAAATACTGTTCAACGTTAAATGT

DraI

18001 AGTCTTGAAATTCCACGTAGGAATGTGGCAACTTTACAAGCTGAAAATGTAACAGGACTCTTTAAAGATTGTAGTAAGGT 18080

TCAGAACTTTAAGGTGCATCCTTACACCGTTGAAATGTTCGACTTTTACATTGTCCTGAGAAATTTCTAACATCATTCCA

18081 AATCACTGGGTTACATCCTACACAGGCACCTACACACCTCAGTGTTGACACTAAATTCAAAACTGAAGGTTTATGTGTTG 18160

TTAGTGACCCAATGTAGGATGTGTCCGTGGATGTGTGGAGTCACAACTGTGATTTAAGTTTTGACTTCCAAATACACAAC

Bsu36IDraI

18161 ACATACCTGGCATACCTAAGGACATGACCTATAGAAGACTCATCTCTATGATGGGTTTTAAAATGAATTATCAAGTTAAT 18240

TGTATGGACCGTATGGATTCCTGTACTGGATATCTTCTGAGTAGAGATACTACCCAAAATTTTACTTAATAGTTCAATTA

18241 GGTTACCCTAACATGTTTATCACCCGCGAAGAAGCTATAAGACATGTACGTGCATGGATTGGCTTCGATGTCGAGGGGTG 18320

CCAATGGGATTGTACAAATAGTGGGCGCTTCTTCGATATTCTGTACATGCACGTACCTAACCGAAGCTACAGCTCCCCAC

KpnIHpaI

18321 TCATGCTACTAGAGAAGCTGTTGGTACCAATTTACCTTTACAGCTAGGTTTTTCTACAGGTGTTAACCTAGTTGCTGTAC 18400

AGTACGATGATCTCTTCGACAACCATGGTTAAATGGAAATGTCGATCCAAAAAGATGTCCACAATTGGATCAACGACATG

DraI

18401 CTACAGGTTATGTTGATACACCTAATAATACAGATTTTTCCAGAGTTAGTGCTAAACCACCGCCTGGAGATCAATTTAAA 18480

GATGTCCAATACAACTATGTGGATTATTATGTCTAAAAAGGTCTCAATCACGATTTGGTGGCGGACCTCTAGTTAAATTT

BsrGIBsrGI

18481 CACCTCATACCACTTATGTACAAAGGACTTCCTTGGAATGTAGTGCGTATAAAGATTGTACAAATGTTAAGTGACACACT 18560

GTGGAGTATGGTGAATACATGTTTCCTGAAGGAACCTTACATCACGCATATTTCTAACATGTTTACAATTCACTGTGTGA

18561 TAAAAATCTCTCTGACAGAGTCGTATTTGTCTTATGGGCACATGGCTTTGAGTTGACATCTATGAAGTATTTTGTGAAAA 18640

ATTTTTAGAGAGACTGTCTCAGCATAAACAGAATACCCGTGTACCGAAACTCAACTGTAGATACTTCATAAAACACTTTT

18641 TAGGACCTGAGCGCACCTGTTGTCTATGTGATAGACGTGCCACATGCTTTTCCACTGCTTCAGACACTTATGCCTGTTGG 18720

ATCCTGGACTCGCGTGGACAACAGATACACTATCTGCACGGTGTACGAAAAGGTGACGAAGTCTGTGAATACGGACAACC

18721 CATCATTCTATTGGATTTGATTACGTCTATAATCCGTTTATGATTGATGTTCAACAATGGGGTTTTACAGGTAACCTACA 18800

GTAGTAAGATAACCTAAACTAATGCAGATATTAGGCAAATACTAACTACAAGTTGTTACCCCAAAATGTCCATTGGATGT

NcoIBspHI

18801 AAGCAACCATGATCTGTATTGTCAAGTCCATGGTAATGCACATGTAGCTAGTTGTGATGCAATCATGACTAGGTGTCTAG 18880

TTCGTTGGTACTAGACATAACAGTTCAGGTACCATTACGTGTACATCGATCAACACTACGTTAGTACTGATCCACAGATC

AseI

18881 CTGTCCACGAGTGCTTTGTTAAGCGTGTTGACTGGACTATTGAATATCCTATAATTGGTGATGAACTGAAGATTAATGCG 18960

GACAGGTGCTCACGAAACAATTCGCACAACTGACCTGATAACTTATAGGATATTAACCACTACTTGACTTCTAATTACGC

18961 GCTTGTAGAAAGGTTCAACACATGGTTGTTAAAGCTGCATTATTAGCAGACAAATTCCCAGTTCTTCACGACATTGGTAA 19040

CGAACATCTTTCCAAGTTGTGTACCAACAATTTCGACGTAATAATCGTCTGTTTAAGGGTCAAGAAGTGCTGTAACCATT

HindIIIPsiI

19041 CCCTAAAGCTATTAAGTGTGTACCTCAAGCTGATGTAGAATGGAAGTTCTATGATGCACAGCCTTGTAGTGACAAAGCTT 19120

GGGATTTCGATAATTCACACATGGAGTTCGACTACATCTTACCTTCAAGATACTACGTGTCGGAACATCACTGTTTCGAA

19121 ATAAAATAGAAGAATTATTCTATTCTTATGCCACACATTCTGACAAATTCACAGATGGTGTATGCCTATTTTGGAATTGC 19200

TATTTTATCTTCTTAATAAGATAAGAATACGGTGTGTAAGACTGTTTAAGTGTCTACCACATACGGATAAAACCTTAACG

EcoRV

19201 AATGTCGATAGATATCCTGCTAATTCCATTGTTTGTAGATTTGACACTAGAGTGCTATCTAACCTTAACTTGCCTGGTTG 19280

TTACAGCTATCTATAGGACGATTAAGGTAACAAACATCTAAACTGTGATCTCACGATAGATTGGAATTGAACGGACCAAC

polyA signal

NsiIDraI

19281 TGATGGTGGCAGTTTGTATGTAAATAAACATGCATTCCACACACCAGCTTTTGATAAAAGTGCTTTTGTTAATTTAAAAC 19360

ACTACCACCGTCAAACATACATTTATTTGTACGTAAGGTGTGTGGTCGAAAACTATTTTCACGAAAACAATTAAATTTTG

19361 AATTACCATTTTTCTATTACTCTGACAGTCCATGTGAGTCTCATGGAAAACAAGTAGTGTCAGATATAGATTATGTACCA 19440

TTAATGGTAAAAAGATAATGAGACTGTCAGGTACACTCAGAGTACCTTTTGTTCATCACAGTCTATATCTAATACATGGT

19441 CTAAAGTCTGCTACGTGTATAACACGTTGCAATTTAGGTGGTGCTGTCTGTAGACATCATGCTAATGAGTACAGATTGTA 19520

GATTTCAGACGATGCACATATTGTGCAACGTTAAATCCACCACGACAGACATCTGTAGTACGATTACTCATGTCTAACAT

PsiIPvuIIPsiI

19521 TCTCGATGCTTATAACATGATGATCTCAGCTGGCTTTAGCTTGTGGGTTTACAAACAATTTGATACTTATAACCTCTGGA 19600

AGAGCTACGAATATTGTACTACTAGAGTCGACCGAAATCGAACACCCAAATGTTTGTTAAACTATGAATATTGGAGACCT

19601 ACACTTTTACAAGACTTCAGAGTTTAGAAAATGTGGCTTTTAATGTTGTAAATAAGGGACACTTTGATGGACAACAGGGT 19680

TGTGAAAATGTTCTGAAGTCTCAAATCTTTTACACCGAAAATTACAACATTTATTCCCTGTGAAACTACCTGTTGTCCCA

polyA signal

AseI

19681 GAAGTACCAGTTTCTATCATTAATAACACTGTTTACACAAAAGTTGATGGTGTTGATGTAGAATTGTTTGAAAATAAAAC 19760

CTTCATGGTCAAAGATAGTAATTATTGTGACAAATGTGTTTTCAACTACCACAACTACATCTTAACAAACTTTTATTTTG

19761 AACATTACCTGTTAATGTAGCATTTGAGCTTTGGGCTAAGCGCAACATTAAACCAGTACCAGAGGTGAAAATACTCAATA 19840

TTGTAATGGACAATTACATCGTAAACTCGAAACCCGATTCGCGTTGTAATTTGGTCATGGTCTCCACTTTTATGAGTTAT

19841 ATTTGGGTGTGGACATTGCTGCTAATACTGTGATCTGGGACTACAAAAGAGATGCTCCAGCACATATATCTACTATTGGT 19920

TAAACCCACACCTGTAACGACGATTATGACACTAGACCCTGATGTTTTCTCTACGAGGTCGTGTATATAGATGATAACCA

ApaLI

19921 GTTTGTTCTATGACTGACATAGCCAAGAAACCAACTGAAACGATTTGTGCACCACTCACTGTCTTTTTTGATGGTAGAGT 20000

CAAACAAGATACTGACTGTATCGGTTCTTTGGTTGACTTTGCTAAACACGTGGTGAGTGACAGAAAAAACTACCATCTCA

20001 TGATGGTCAAGTAGACTTATTTAGAAATGCCCGTAATGGTGTTCTTATTACAGAAGGTAGTGTTAAAGGTTTACAACCAT 20080

ACTACCAGTTCATCTGAATAAATCTTTACGGGCATTACCACAAGAATAATGTCTTCCATCACAATTTCCAAATGTTGGTA

AseIPsiI

20081 CTGTAGGTCCCAAACAAGCTAGTCTTAATGGAGTCACATTAATTGGAGAAGCCGTAAAAACACAGTTCAATTATTATAAG 20160

GACATCCAGGGTTTGTTCGATCAGAATTACCTCAGTGTAATTAACCTCTTCGGCATTTTTGTGTCAAGTTAATAATATTC

DraI

20161 AAAGTTGATGGTGTTGTCCAACAATTACCTGAAACTTACTTTACTCAGAGTAGAAATTTACAAGAATTTAAACCCAGGAG 20240

TTTCAACTACCACAACAGGTTGTTAATGGACTTTGAATGAAATGAGTCTCATCTTTAAATGTTCTTAAATTTGGGTCCTC

EcoRIBstBI

20241 TCAAATGGAAATTGATTTCTTAGAATTAGCTATGGATGAATTCATTGAACGGTATAAATTAGAAGGCTATGCCTTCGAAC 20320

AGTTTACCTTTAACTAAAGAATCTTAATCGATACCTACTTAAGTAACTTGCCATATTTAATCTTCCGATACGGAAGCTTG

AclI

20321 ATATCGTTTATGGAGATTTTAGTCATAGTCAGTTAGGTGGTTTACATCTACTGATTGGACTAGCTAAACGTTTTAAGGAA 20400

TATAGCAAATACCTCTAAAATCAGTATCAGTCAATCCACCAAATGTAGATGACTAACCTGATCGATTTGCAAAATTCCTT

FspI

20401 TCACCTTTTGAATTAGAAGATTTTATTCCTATGGACAGTACAGTTAAAAACTATTTCATAACAGATGCGCAAACAGGTTC 20480

AGTGGAAAACTTAATCTTCTAAAATAAGGATACCTGTCATGTCAATTTTTGATAAAGTATTGTCTACGCGTTTGTCCAAG

polyA signal

20481 ATCTAAGTGTGTGTGTTCTGTTATTGATTTATTACTTGATGATTTTGTTGAAATAATAAAATCCCAAGATTTATCTGTAG 20560

TAGATTCACACACACAAGACAATAACTAAATAATGAACTACTAAAACAACTTTATTATTTTAGGGTTCTAAATAGACATC

MscI

20561 TTTCTAAGGTTGTCAAAGTGACTATTGACTATACAGAAATTTCATTTATGCTTTGGTGTAAAGATGGCCATGTAGAAACA 20640

AAAGATTCCAACAGTTTCACTGATAACTGATATGTCTTTAAAGTAAATACGAAACCACATTTCTACCGGTACATCTTTGT

20641 TTTTACCCAAAATTACAATCTAGTCAAGCGTGGCAACCGGGTGTTGCTATGCCTAATCTTTACAAAATGCAAAGAATGCT 20720

AAAATGGGTTTTAATGTTAGATCAGTTCGCACCGTTGGCCCACAACGATACGGATTAGAAATGTTTTACGTTTCTTACGA

20721 ATTAGAAAAGTGTGACCTTCAAAATTATGGTGATAGTGCAACATTACCTAAAGGCATAATGATGAATGTCGCAAAATATA 20800

TAATCTTTTCACACTGGAAGTTTTAATACCACTATCACGTTGTAATGGATTTCCGTATTACTACTTACAGCGTTTTATAT

SspIDraI

20801 CTCAACTGTGTCAATATTTAAACACATTAACATTAGCTGTACCCTATAATATGAGAGTTATACATTTTGGTGCTGGTTCT 20880

GAGTTGACACAGTTATAAATTTGTGTAATTGTAATCGACATGGGATATTATACTCTCAATATGTAAAACCACGACCAAGA

PvuIIBglII

20881 GATAAAGGAGTTGCACCAGGTACAGCTGTTTTAAGACAGTGGTTGCCTACGGGTACGCTGCTTGTCGATTCAGATCTTAA 20960

CTATTTCCTCAACGTGGTCCATGTCGACAAAATTCTGTCACCAACGGATGCCCATGCGACGAACAGCTAAGTCTAGAATT

polyA signal

BsrGI

20961 TGACTTTGTCTCTGATGCAGATTCAACTTTGATTGGTGATTGTGCAACTGTACATACAGCTAATAAATGGGATCTCATTA 21040

ACTGAAACAGAGACTACGTCTAAGTTGAAACTAACCACTAACACGTTGACATGTATGTCGATTATTTACCCTAGAGTAAT

21041 TTAGTGATATGTACGACCCTAAGACTAAAAATGTTACAAAAGAAAATGACTCTAAAGAGGGTTTTTTCACTTACATTTGT 21120

AATCACTATACATGCTGGGATTCTGATTTTTACAATGTTTTCTTTTACTGAGATTTCTCCCAAAAAAGTGAATGTAAACA

NheIPsiI

21121 GGGTTTATACAACAAAAGCTAGCTCTTGGAGGTTCCGTGGCTATAAAGATAACAGAACATTCTTGGAATGCTGATCTTTA 21200

CCCAAATATGTTGTTTTCGATCGAGAACCTCCAAGGCACCGATATTTCTATTGTCTTGTAAGAACCTTACGACTAGAAAT

21201 TAAGCTCATGGGACACTTCGCATGGTGGACAGCCTTTGTTACTAATGTGAATGCGTCATCATCTGAAGCATTTTTAATTG 21280

ATTCGAGTACCCTGTGAAGCGTACCACCTGTCGGAAACAATGATTACACTTACGCAGTAGTAGACTTCGTAAAAATTAAC

NsiI/SphI

21281 GATGTAATTATCTTGGCAAACCACGCGAACAAATAGATGGTTATGTCATGCATGCAAATTACATATTTTGGAGGAATACA 21360

CTACATTAATAGAACCGTTTGGTGCGCTTGTTTATCTACCAATACAGTACGTACGTTTAATGTATAAAACCTCCTTATGT

21361 AATCCAATTCAGTTGTCTTCCTATTCTTTATTTGACATGAGTAAATTTCCCCTTAAATTAAGGGGTACTGCTGTTATGTC 21440

TTAGGTTAAGTCAACAGAAGGATAAGAAATAAACTGTACTCATTTAAAGGGGAATTTAATTCCCCATGACGACAATACAG

DraIPsiI

21441 TTTAAAAGAAGGTCAAATCAATGATATGATTTTATCTCTTCTTAGTAAAGGTAGACTTATAATTAGAGAAAACAACAGAG 21520

AAATTTTCTTCCAGTTTAGTTACTATACTAAAATAGAGAAGAATCATTTCCATCTGAATATTAATCTCTTTTGTTGTCTC

HpaISpeI

21521 TTGTTATTTCTAGTGATGTTCTTGTTAACAACTAAACGAACAATGTTTGTTTTTCTTGTTTTATTGCCACTAGTCTCTAG 21600

AACAATAAAGATCACTACAAGAACAATTGTTGATTTGCTTGTTACAAACAAAAAGAACAAAATAACGGTGATCAGAGATC

PmlI

21601 TCAGTGTGTTAATCTTACAACCAGAACTCAATTACCCCCTGCATACACTAATTCTTTCACACGTGGTGTTTATTACCCTG 21680

AGTCACACAATTAGAATGTTGGTCTTGAGTTAATGGGGGACGTATGTGATTAAGAAAGTGTGCACCACAAATAATGGGAC

21681 ACAAAGTTTTCAGATCCTCAGTTTTACATTCAACTCAGGACTTGTTCTTACCTTTCTTTTCCAATGTTACTTGGTTCCAT 21760

TGTTTCAAAAGTCTAGGAGTCAAAATGTAAGTTGAGTCCTGAACAAGAATGGAAAGAAAAGGTTACAATGAACCAAGGTA

21761 GCTATACATGTCTCTGGGACCAATGGTACTAAGAGGTTTGATAACCCTGTCCTACCATTTAATGATGGTGTTTATTTTGC 21840

CGATATGTACAGAGACCCTGGTTACCATGATTCTCCAAACTATTGGGACAGGATGGTAAATTACTACCACAAATAAAACG

BstBI

21841 TTCCACTGAGAAGTCTAACATAATAAGAGGCTGGATTTTTGGTACTACTTTAGATTCGAAGACCCAGTCCCTACTTATTG 21920

AAGGTGACTCTTCAGATTGTATTATTCTCCGACCTAAAAACCATGATGAAATCTAAGCTTCTGGGTCAGGGATGAATAAC

21921 TTAATAACGCTACTAATGTTGTTATTAAAGTCTGTGAATTTCAATTTTGTAATGATCCATTTTTGGGTGTTTATTACCAC 22000

AATTATTGCGATGATTACAACAATAATTTCAGACACTTAAAGTTAAAACATTACTAGGTAAAAACCCACAAATAATGGTG

22001 AAAAACAACAAAAGTTGGATGGAAAGTGAGTTCAGAGTTTATTCTAGTGCGAATAATTGCACTTTTGAATATGTCTCTCA 22080

TTTTTGTTGTTTTCAACCTACCTTTCACTCAAGTCTCAAATAAGATCACGCTTATTAACGTGAAAACTTATACAGAGAGT

SspI

22081 GCCTTTTCTTATGGACCTTGAAGGAAAACAGGGTAATTTCAAAAATCTTAGGGAATTTGTGTTTAAGAATATTGATGGTT 22160

CGGAAAAGAATACCTGGAACTTCCTTTTGTCCCATTAAAGTTTTTAGAATCCCTTAAACACAAATTCTTATAACTACCAA

DraIAseIBsu36I

22161 ATTTTAAAATATATTCTAAGCACACGCCTATTAATTTAGTGCGTGATCTCCCTCAGGGTTTTTCGGCTTTAGAACCATTG 22240

TAAAATTTTATATAAGATTCGTGTGCGGATAATTAAATCACGCACTAGAGGGAGTCCCAAAAAGCCGAAATCTTGGTAAC

22241 GTAGATTTGCCAATAGGTATTAACATCACTAGGTTTCAAACTTTACTTGCTTTACATAGAAGTTATTTGACTCCTGGTGA 22320

CATCTAAACGGTTATCCATAATTGTAGTGATCCAAAGTTTGAAATGAACGAAATGTATCTTCAATAAACTGAGGACCACT

PvuIIPstIAvrII

22321 TTCTTCTTCAGGTTGGACAGCTGGTGCTGCAGCTTATTATGTGGGTTATCTTCAACCTAGGACTTTTCTATTAAAATATA 22400

AAGAAGAAGTCCAACCTGTCGACCACGACGTCGAATAATACACCCAATAGAAGTTGGATCCTGAAAAGATAATTTTATAT

ApaLI

22401 ATGAAAATGGAACCATTACAGATGCTGTAGACTGTGCACTTGACCCTCTCTCAGAAACAAAGTGTACGTTGAAATCCTTC 22480

TACTTTTACCTTGGTAATGTCTACGACATCTGACACGTGAACTGGGAGAGAGTCTTTGTTTCACATGCAACTTTAGGAAG

SspI

22481 ACTGTAGAAAAAGGAATCTATCAAACTTCTAACTTTAGAGTCCAACCAACAGAATCTATTGTTAGATTTCCTAATATTAC 22560

TGACATCTTTTTCCTTAGATAGTTTGAAGATTGAAATCTCAGGTTGGTTGTCTTAGATAACAATCTAAAGGATTATAATG

22561 AAACTTGTGCCCTTTTGGTGAAGTTTTTAACGCCACCAGATTTGCATCTGTTTATGCTTGGAACAGGAAGAGAATCAGCA 22640

TTTGAACACGGGAAAACCACTTCAAAAATTGCGGTGGTCTAAACGTAGACAAATACGAACCTTGTCCTTCTCTTAGTCGT

22641 ACTGTGTTGCTGATTATTCTGTCCTATATAATTCCGCATCATTTTCCACTTTTAAGTGTTATGGAGTGTCTCCTACTAAA 22720

TGACACAACGACTAATAAGACAGGATATATTAAGGCGTAGTAAAAGGTGAAAATTCACAATACCTCACAGAGGATGATTT

22721 TTAAATGATCTCTGCTTTACTAATGTCTATGCAGATTCATTTGTAATTAGAGGTGATGAAGTCAGACAAATCGCTCCAGG 22800

AATTTACTAGAGACGAAATGATTACAGATACGTCTAAGTAAACATTAATCTCCACTACTTCAGTCTGTTTAGCGAGGTCC

PsiIPsiIEcoRI

22801 GCAAACTGGAAAGATTGCTGATTATAATTATAAATTACCAGATGATTTTACAGGCTGCGTTATAGCTTGGAATTCTAACA 22880

CGTTTGACCTTTCTAACGACTAATATTAATATTTAATGGTCTACTAAAATGTCCGACGCAATATCGAACCTTAAGATTGT

PsiI

22881 ATCTTGATTCTAAGGTTGGTGGTAATTATAATTACCTGTATAGATTGTTTAGGAAGTCTAATCTCAAACCTTTTGAGAGA 22960

TAGAACTAAGATTCCAACCACCATTAATATTAATGGACATATCTAACAAATCCTTCAGATTAGAGTTTGGAAAACTCTCT

22961 GATATTTCAACTGAAATCTATCAGGCCGGTAGCACACCTTGTAATGGTGTTGAAGGTTTTAATTGTTACTTTCCTTTACA 23040

CTATAAAGTTGACTTTAGATAGTCCGGCCATCGTGTGGAACATTACCACAACTTCCAAAATTAACAATGAAAGGAAATGT

NdeIScaI

23041 ATCATATGGTTTCCAACCCACTAATGGTGTTGGTTACCAACCATACAGAGTAGTAGTACTTTCTTTTGAACTTCTACATG 23120

TAGTATACCAAAGGTTGGGTGATTACCACAACCAATGGTTGGTATGTCTCATCATCATGAAAGAAAACTTGAAGATGTAC

23121 CACCAGCAACTGTTTGTGGACCTAAAAAGTCTACTAATTTGGTTAAAAACAAATGTGTCAATTTCAACTTCAATGGTTTA 23200

GTGGTCGTTGACAAACACCTGGATTTTTCAGATGATTAAACCAATTTTTGTTTACACAGTTAAAGTTGAAGTTACCAAAT

23201 ACAGGCACAGGTGTTCTTACTGAGTCTAACAAAAAGTTTCTGCCTTTCCAACAATTTGGCAGAGACATTGCTGACACTAC 23280

TGTCCGTGTCCACAAGAATGACTCAGATTGTTTTTCAAAGACGGAAAGGTTGTTAAACCGTCTCTGTAACGACTGTGATG

PsiI

23281 TGATGCTGTCCGTGATCCACAGACACTTGAGATTCTTGACATTACACCATGTTCTTTTGGTGGTGTCAGTGTTATAACAC 23360

ACTACGACAGGCACTAGGTGTCTGTGAACTCTAAGAACTGTAATGTGGTACAAGAAAACCACCACAGTCACAATATTGTG

HpaI

23361 CAGGAACAAATACTTCTAACCAGGTTGCTGTTCTTTATCAGGATGTTAACTGCACAGAAGTCCCTGTTGCTATTCATGCA 23440

GTCCTTGTTTATGAAGATTGGTCCAACGACAAGAAATAGTCCTACAATTGACGTGTCTTCAGGGACAACGATAAGTACGT

PmlI

23441 GATCAACTTACTCCTACTTGGCGTGTTTATTCTACAGGTTCTAATGTTTTTCAAACACGTGCAGGCTGTTTAATAGGGGC 23520

CTAGTTGAATGAGGATGAACCGCACAAATAAGATGTCCAAGATTACAAAAAGTTTGTGCACGTCCGACAAATTATCCCCG

NdeI

23521 TGAACATGTCAACAACTCATATGAGTGTGACATACCCATTGGTGCAGGTATATGCGCTAGTTATCAGACTCAGACTAATT 23600

ACTTGTACAGTTGTTGAGTATACTCACACTGTATGGGTAACCACGTCCATATACGCGATCAATAGTCTGAGTCTGATTAA

23601 CTCCTCGGCGGGCACGTAGTGTAGCTAGTCAATCCATCATTGCCTACACTATGTCACTTGGTGCAGAAAATTCAGTTGCT 23680

GAGGAGCCGCCCGTGCATCACATCGATCAGTTAGGTAGTAACGGATGTGATACAGTGAACCACGTCTTTTAAGTCAACGA

23681 TACTCTAATAACTCTATTGCCATACCCACAAATTTTACTATTAGTGTTACCACAGAAATTCTACCAGTGTCTATGACCAA 23760

ATGAGATTATTGAGATAACGGTATGGGTGTTTAAAATGATAATCACAATGGTGTCTTTAAGATGGTCACAGATACTGGTT

BsrGIBsrGIBsrGI

23761 GACATCAGTAGATTGTACAATGTACATTTGTGGTGATTCAACTGAATGCAGCAATCTTTTGTTGCAATATGGCAGTTTTT 23840

CTGTAGTCATCTAACATGTTACATGTAAACACCACTAAGTTGACTTACGTCGTTAGAAAACAACGTTATACCGTCAAAAA

23841 GTACACAATTAAACCGTGCTTTAACTGGAATAGCTGTTGAACAAGACAAAAACACCCAAGAAGTTTTTGCACAAGTCAAA 23920

CATGTGTTAATTTGGCACGAAATTGACCTTATCGACAACTTGTTCTGTTTTTGTGGGTTCTTCAAAAACGTGTTCAGTTT

SspI

23921 CAAATTTACAAAACACCACCAATTAAAGATTTTGGTGGTTTTAATTTTTCACAAATATTACCAGATCCATCAAAACCAAG 24000

GTTTAAATGTTTTGTGGTGGTTAATTTCTAAAACCACCAAAATTAAAAAGTGTTTATAATGGTCTAGGTAGTTTTGGTTC

BglII

24001 CAAGAGGTCATTTATTGAAGATCTACTTTTCAACAAAGTGACACTTGCAGATGCTGGCTTCATCAAACAATATGGTGATT 24080

GTTCTCCAGTAAATAACTTCTAGATGAAAAGTTGTTTCACTGTGAACGTCTACGACCGAAGTAGTTTGTTATACCACTAA

ApaLI

24081 GCCTTGGTGATATTGCTGCTAGAGACCTCATTTGTGCACAAAAGTTTAACGGCCTTACTGTTTTGCCACCTTTGCTCACA 24160

CGGAACCACTATAACGACGATCTCTGGAGTAAACACGTGTTTTCAAATTGCCGGAATGACAAAACGGTGGAAACGAGTGT

24161 GATGAAATGATTGCTCAATACACTTCTGCACTGTTAGCGGGTACAATCACTTCTGGTTGGACCTTTGGTGCAGGTGCTGC 24240

CTACTTTACTAACGAGTTATGTGAAGACGTGACAATCGCCCATGTTAGTGAAGACCAACCTGGAAACCACGTCCACGACG

24241 ATTACAAATACCATTTGCTATGCAAATGGCTTATAGGTTTAATGGTATTGGAGTTACACAGAATGTTCTCTATGAGAACC 24320

TAATGTTTATGGTAAACGATACGTTTACCGAATATCCAAATTACCATAACCTCAATGTGTCTTACAAGAGATACTCTTGG

ApaLI

24321 AAAAATTGATTGCCAACCAATTTAATAGTGCTATTGGCAAAATTCAAGACTCACTTTCTTCCACAGCAAGTGCACTTGGA 24400

TTTTTAACTAACGGTTGGTTAAATTATCACGATAACCGTTTTAAGTTCTGAGTGAAAGAAGGTGTCGTTCACGTGAACCT

HindIIIDraI

24401 AAACTTCAAGATGTGGTCAACCAAAATGCACAAGCTTTAAACACGCTTGTTAAACAACTTAGCTCCAATTTTGGTGCAAT 24480

TTTGAAGTTCTACACCAGTTGGTTTTACGTGTTCGAAATTTGTGCGAACAATTTGTTGAATCGAGGTTAAAACCACGTTA

DraIEcoRV

24481 TTCAAGTGTTTTAAATGATATCCTTTCACGTCTTGACAAAGTTGAGGCTGAAGTGCAAATTGATAGGTTGATCACAGGCA 24560

AAGTTCACAAAATTTACTATAGGAAAGTGCAGAACTGTTTCAACTCCGACTTCACGTTTAACTATCCAACTAGTGTCCGT

NdeIAseIPstI

24561 GACTTCAAAGTTTGCAGACATATGTGACTCAACAATTAATTAGAGCTGCAGAAATCAGAGCTTCTGCTAATCTTGCTGCT 24640

CTGAAGTTTCAAACGTCTGTATACACTGAGTTGTTAATTAATCTCGACGTCTTTAGTCTCGAAGACGATTAGAACGACGA

24641 ACTAAAATGTCAGAGTGTGTACTTGGACAATCAAAAAGAGTTGATTTTTGTGGAAAGGGCTATCATCTTATGTCCTTCCC 24720

TGATTTTACAGTCTCACACATGAACCTGTTAGTTTTTCTCAACTAAAAACACCTTTCCCGATAGTAGAATACAGGAAGGG

24721 TCAGTCAGCACCTCATGGTGTAGTCTTCTTGCATGTGACTTATGTCCCTGCACAAGAAAAGAACTTCACAACTGCTCCTG 24800

AGTCAGTCGTGGAGTACCACATCAGAAGAACGTACACTGAATACAGGGACGTGTTCTTTTCTTGAAGTGTTGACGAGGAC

BspHI

24801 CCATTTGTCATGATGGAAAAGCACACTTTCCTCGTGAAGGTGTCTTTGTTTCAAATGGCACACACTGGTTTGTAACACAA 24880

GGTAAACAGTACTACCTTTTCGTGTGAAAGGAGCACTTCCACAGAAACAAAGTTTACCGTGTGTGACCAAACATTGTGTT

24881 AGGAATTTTTATGAACCACAAATCATTACTACAGACAACACATTTGTGTCTGGTAACTGTGATGTTGTAATAGGAATTGT 24960

TCCTTAAAAATACTTGGTGTTTAGTAATGATGTCTGTTGTGTAAACACAGACCATTGACACTACAACATTATCCTTAACA

SspI

24961 CAACAACACAGTTTATGATCCTTTGCAACCTGAATTAGACTCATTCAAGGAGGAGTTAGATAAATATTTTAAGAATCATA 25040

GTTGTTGTGTCAAATACTAGGAAACGTTGGACTTAATCTGAGTAAGTTCCTCCTCAATCTATTTATAAAATTCTTAGTAT

AseI

25041 CATCACCAGATGTTGATTTAGGTGACATCTCTGGCATTAATGCTTCAGTTGTAAACATTCAAAAAGAAATTGACCGCCTC 25120

GTAGTGGTCTACAACTAAATCCACTGTAGAGACCGTAATTACGAAGTCAACATTTGTAAGTTTTTCTTTAACTGGCGGAG

DraIClaI*MscINcoI

25121 AATGAGGTTGCCAAGAATTTAAATGAATCTCTCATCGATCTCCAAGAACTTGGAAAGTATGAGCAGTATATAAAATGGCC 25200

TTACTCCAACGGTTCTTAAATTTACTTAGAGAGTAGCTAGAGGTTCTTGAACCTTTCATACTCGTCATATATTTTACCGG

25201 ATGGTACATTTGGCTAGGTTTTATAGCTGGCTTGATTGCCATAGTAATGGTGACAATTATGCTTTGCTGTATGACCAGTT 25280

TACCATGTAAACCGATCCAAAATATCGACCGAACTAACGGTATCATTACCACTGTTAATACGAAACGACATACTGGTCAA

BamHI

25281 GCTGTAGTTGTCTCAAGGGCTGTTGTTCTTGTGGATCCTGCTGCAAATTTGATGAAGACGACTCTGAGCCAGTGCTCAAA 25360

CGACATCAACAGAGTTCCCGACAACAAGAACACCTAGGACGACGTTTAAACTACTTCTGCTGAGACTCGGTCACGAGTTT

MfeI

25361 GGAGTCAAATTACATTACACATAAACGAACTTATGGATTTGTTTATGAGAATCTTCACAATTGGAACTGTAACTTTGAAG 25440

CCTCAGTTTAATGTAATGTGTATTTGCTTGAATACCTAAACAAATACTCTTAGAAGTGTTAACCTTGACATTGAAACTTC

25441 CAAGGTGAAATCAAGGATGCTACTCCTTCAGATTTTGTTCGCGCTACTGCAACGATACCGATACAAGCCTCACTCCCTTT 25520

GTTCCACTTTAGTTCCTACGATGAGGAAGTCTAAAACAAGCGCGATGACGTTGCTATGGCTATGTTCGGAGTGAGGGAAA

AfeI

25521 CGGATGGCTTATTGTTGGCGTTGCACTTCTTGCTGTTTTTCAGAGCGCTTCCAAAATCATAACCCTCAAAAAGAGATGGC 25600

GCCTACCGAATAACAACCGCAACGTGAAGAACGACAAAAAGTCTCGCGAAGGTTTTAGTATTGGGAGTTTTTCTCTACCG

25601 AACTAGCACTCTCCAAGGGTGTTCACTTTGTTTGCAACTTGCTGTTGTTGTTTGTAACAGTTTACTCACACCTTTTGCTC 25680

TTGATCGTGAGAGGTTCCCACAAGTGAAACAAACGTTGAACGACAACAACAAACATTGTCAAATGAGTGTGGAAAACGAG

25681 GTTGCTGCTGGCCTTGAAGCCCCTTTTCTCTATCTTTATGCTTTAGTCTACTTCTTGCAGAGTATAAACTTTGTAAGAAT 25760

CAACGACGACCGGAACTTCGGGGAAAAGAGATAGAAATACGAAATCAGATGAAGAACGTCTCATATTTGAAACATTCTTA

25761 AATAATGAGGCTTTGGCTTTGCTGGAAATGCCGTTCCAAAAACCCATTACTTTATGATGCCAACTATTTTCTTTGCTGGC 25840

TTATTACTCCGAAACCGAAACGACCTTTACGGCAAGGTTTTTGGGTAATGAAATACTACGGTTGATAAAAGAAACGACCG

MfeI

25841 ATACTAATTGTTACGACTATTGTATACCTTACAATAGTGTAACTTCTTCAATTGTCATTACTTCAGGTGATGGCACAACA 25920

TATGATTAACAATGCTGATAACATATGGAATGTTATCACATTGAAGAAGTTAACAGTAATGAAGTCCACTACCGTGTTGT

25921 AGTCCTATTTCTGAACATGACTACCAGATTGGTGGTTATACTGAAAAATGGGAATCTGGAGTAAAAGACTGTGTTGTATT 26000

TCAGGATAAAGACTTGTACTGATGGTCTAACCACCAATATGACTTTTTACCCTTAGACCTCATTTTCTGACACAACATAA

PvuIIMfeI

26001 ACACAGTTACTTCACTTCAGACTATTACCAGCTGTACTCAACTCAATTGAGTACAGACACTGGTGTTGAACATGTTACCT 26080

TGTGTCAATGAAGTGAAGTCTGATAATGGTCGACATGAGTTGAGTTAACTCATGTCTGTGACCACAACTTGTACAATGGA

polyA signal

BspEI

26081 TCTTCATCTACAATAAAATTGTTGATGAGCCTGAAGAACATGTCCAAATTCACACAATCGACGGTTCATCCGGAGTTGTT 26160

AGAAGTAGATGTTATTTTAACAACTACTCGGACTTCTTGTACAGGTTTAAGTGTGTTAGCTGCCAAGTAGGCCTCAACAA

26161 AATCCAGTAATGGAACCAATTTATGATGAACCGACGACGACTACTAGCGTGCCTTTGTAAGCACAAGCTGATGAGTACGA 26240

TTAGGTCATTACCTTGGTTAAATACTACTTGGCTGCTGCTGATGATCGCACGGAAACATTCGTGTTCGACTACTCATGCT

26241 ACTTATGTACTCATTCGTTTCGGAAGAGACAGGTACGTTAATAGTTAATAGCGTACTTCTTTTTCTTGCTTTCGTGGTAT 26320

TGAATACATGAGTAAGCAAAGCCTTCTCTGTCCATGCAATTATCAATTATCGCATGAAGAAAAAGAACGAAAGCACCATA

SspIHpaI

26321 TCTTGCTAGTTACACTAGCCATCCTTACTGCGCTTCGATTGTGTGCGTACTGCTGCAATATTGTTAACGTGAGTCTTGTA 26400

AGAACGATCAATGTGATCGGTAGGAATGACGCGAAGCTAACACACGCATGACGACGTTATAACAATTGCACTCAGAACAT

EcoRIXbaI

26401 AAACCTTCTTTTTACGTTTACTCTCGTGTTAAAAATCTGAATTCTTCTAGAGTTCCTGATCTTCTGGTCTAAACGAACTA 26480

TTTGGAAGAAAAATGCAAATGAGAGCACAATTTTTAGACTTAAGAAGATCTCAAGGACTAGAAGACCAGATTTGCTTGAT

SspINcoI

26481 AATATTATATTAGTTTTTCTGTTTGGAACTTTAATTTTAGCCATGGCAGATTCCAACGGTACTATTACCGTTGAAGAGCT 26560

TTATAATATAATCAAAAAGACAAACCTTGAAATTAAAATCGGTACCGTCTAAGGTTGCCATGATAATGGCAACTTCTCGA

26561 TAAAAAGCTCCTTGAACAATGGAACCTAGTAATAGGTTTCCTATTCCTTACATGGATTTGTCTTCTACAATTTGCCTATG 26640

ATTTTTCGAGGAACTTGTTACCTTGGATCATTATCCAAAGGATAAGGAATGTACCTAAACAGAAGATGTTAAACGGATAC

MscI

26641 CCAACAGGAATAGGTTTTTGTATATAATTAAGTTAATTTTCCTCTGGCTGTTATGGCCAGTAACTTTAGCTTGTTTTGTG 26720

GGTTGTCCTTATCCAAAAACATATATTAATTCAATTAAAAGGAGACCGACAATACCGGTCATTGAAATCGAACAAAACAC

polyA signal

AgeI

26721 CTTGCTGCTGTTTACAGAATAAATTGGATCACCGGTGGAATTGCTATCGCAATGGCTTGTCTTGTAGGCTTGATGTGGCT 26800

GAACGACGACAAATGTCTTATTTAACCTAGTGGCCACCTTAACGATAGCGTTACCGAACAGAACATCCGAACTACACCGA

BsiWI/MluI

26801 CAGCTACTTCATTGCTTCTTTCAGACTGTTTGCGCGTACGCGTTCCATGTGGTCATTCAATCCAGAAACTAACATTCTTC 26880

GTCGATGAAGTAACGAAGAAAGTCTGACAAACGCGCATGCGCAAGGTACACCAGTAAGTTAGGTCTTTGATTGTAAGAAG

NcoIXbaI

26881 TCAACGTGCCACTCCATGGCACTATTCTGACCAGACCGCTTCTAGAAAGTGAACTCGTAATCGGAGCTGTGATCCTTCGT 26960

AGTTGCACGGTGAGGTACCGTGATAAGACTGGTCTGGCGAAGATCTTTCACTTGAGCATTAGCCTCGACACTAGGAAGCA

26961 GGACATCTTCGTATTGCTGGACACCATCTAGGACGCTGTGACATCAAGGACCTGCCTAAAGAAATCACTGTTGCTACATC 27040

CCTGTAGAAGCATAACGACCTGTGGTAGATCCTGCGACACTGTAGTTCCTGGACGGATTTCTTTAGTGACAACGATGTAG

27041 ACGAACGCTTTCTTATTACAAATTGGGAGCTTCGCAGCGTGTAGCAGGTGACTCAGGTTTTGCTGCATACAGTCGCTACA 27120

TGCTTGCGAAAGAATAATGTTTAACCCTCGAAGCGTCGCACATCGTCCACTGAGTCCAAAACGACGTATGTCAGCGATGT

SspIBsrGI

27121 GGATTGGCAACTATAAATTAAACACAGACCATTCCAGTAGCAGTGACAATATTGCTTTGCTTGTACAGTAAGTGACAACA 27200

CCTAACCGTTGATATTTAATTTGTGTCTGGTAAGGTCATCGTCACTGTTATAACGAAACGAACATGTCATTCACTGTTGT

DraI

27201 GATGTTTCATCTCGTTGACTTTCAGGTTACTATAGCAGAGATATTACTAATTATTATGAGGACTTTTAAAGTTTCCATTT 27280

CTACAAAGTAGAGCAACTGAAAGTCCAATGATATCGTCTCTATAATGATTAATAATACTCCTGAAAATTTCAAAGGTAAA

polyA signal

SspI

27281 GGAATCTTGATTACATCATAAACCTCATAATTAAAAATTTATCTAAGTCACTAACTGAGAATAAATATTCTCAATTAGAT 27360

CCTTAGAACTAATGTAGTATTTGGAGTATTAATTTTTAAATAGATTCAGTGATTGACTCTTATTTATAAGAGTTAATCTA

27361 GAAGAGCAACCAATGGAGATTGATTAAACGAACATGAAAATTATTCTTTTCTTGGCACTGATAACACTCGCTACTTGTGA 27440

CTTCTCGTTGGTTACCTCTAACTAATTTGCTTGTACTTTTAATAAGAAAAGAACCGTGACTATTGTGAGCGATGAACACT

ScaIDraI

27441 GCTTTATCACTACCAAGAGTGTGTTAGAGGTACAACAGTACTTTTAAAAGAACCTTGCTCTTCTGGAACATACGAGGGCA 27520

CGAAATAGTGATGGTTCTCACACAATCTCCATGTTGTCATGAAAATTTTCTTGGAACGAGAAGACCTTGTATGCTCCCGT

27521 ATTCACCATTTCATCCTCTAGCTGATAACAAATTTGCACTGACTTGCTTTAGCACTCAATTTGCTTTTGCTTGTCCTGAC 27600

TAAGTGGTAAAGTAGGAGATCGACTATTGTTTAAACGTGACTGAACGAAATCGTGAGTTAAACGAAAACGAACAGGACTG

27601 GGCGTAAAACACGTCTATCAGTTACGTGCCAGATCAGTTTCACCTAAACTGTTCATCAGACAAGAGGAAGTTCAAGAACT 27680

CCGCATTTTGTGCAGATAGTCAATGCACGGTCTAGTCAAAGTGGATTTGACAAGTAGTCTGTTCTCCTTCAAGTTCTTGA

PsiI

27681 TTACTCTCCAATTTTTCTTATTGTTGCGGCAATAGTGTTTATAACACTTTGCTTCACACTCAAAAGAAAGACAGAATGAT 27760

AATGAGAGGTTAAAAAGAATAACAACGCCGTTATCACAAATATTGTGAAACGAAGTGTGAGTTTTCTTTCTGTCTTACTA

AseI

27761 TGAACTTTCATTAATTGACTTCTATTTGTGCTTTTTAGCCTTTCTGCTATTCCTTGTTTTAATTATGCTTATTATCTTTT 27840

ACTTGAAAGTAATTAACTGAAGATAAACACGAAAAATCGGAAAGACGATAAGGAACAAAATTAATACGAATAATAGAAAA

27841 GGTTCTCACTTGAACTGCAAGATCATAATGAAACTTGTCACGCCTAAACGAACATGAAATTTCTTGTTTTCTTAGGAATC 27920

CCAAGAGTGAACTTGACGTTCTAGTATTACTTTGAACAGTGCGGATTTGCTTGTACTTTAAAGAACAAAAGAATCCTTAG

NdeI

27921 ATCACAACTGTAGCTGCATTTCACCAAGAATGTAGTTTACAGTCATGTACTCAACATCAACCATATGTAGTTGATGACCC 28000

TAGTGTTGACATCGACGTAAAGTGGTTCTTACATCAAATGTCAGTACATGAGTTGTAGTTGGTATACATCAACTACTGGG

28001 GTGTCCTATTCACTTCTATTCTAAATGGTATATTAGAGTAGGAGCTAGAAAATCAGCACCTTTAATTGAATTGTGCGTGG 28080

CACAGGATAAGTGAAGATAAGATTTACCATATAATCTCATCCTCGATCTTTTAGTCGTGGAAATTAACTTAACACGCACC

ClaIEcoRVAseI

28081 ATGAGGCTGGTTCTAAATCACCCATTCAGTACATCGATATCGGTAATTATACAGTTTCCTGTTTACCTTTTACAATTAAT 28160

TACTCCGACCAAGATTTAGTGGGTAAGTCATGTAGCTATAGCCATTAATATGTCAAAGGACAAATGGAAAATGTTAATTA

BspHI

28161 TGCCAGGAACCTAAATTGGGTAGTCTTGTAGTGCGTTGTTCGTTCTATGAAGACTTTTTAGAGTATCATGACGTTCGTGT 28240

ACGGTCCTTGGATTTAACCCATCAGAACATCACGCAACAAGCAAGATACTTCTGAAAAATCTCATAGTACTGCAAGCACA

28241 TGTTTTAGATTTCATCTAAACGAACAAACTAAAATGTCTGATAATGGACCCCAAAATCAGCGAAATGCACCCCGCATTAC 28320

ACAAAATCTAAAGTAGATTTGCTTGTTTGATTTTACAGACTATTACCTGGGGTTTTAGTCGCTTTACGTGGGGCGTAATG

28321 GTTTGGTGGACCCTCAGATTCAACTGGCAGTAACCAGAATGGAGAACGCAGTGGGGCGCGATCAAAACAACGTCGGCCCC 28400

CAAACCACCTGGGAGTCTAAGTTGACCGTCATTGGTCTTACCTCTTGCGTCACCCCGCGCTAGTTTTGTTGCAGCCGGGG

XhoI

28401 AAGGTTTACCCAATAATACTGCGTCTTGGTTCACCGCTCTCACTCAACATGGCAAGGAAGACCTTAAATTCCCTCGAGGA 28480

TTCCAAATGGGTTATTATGACGCAGAACCAAGTGGCGAGAGTGAGTTGTACCGTTCCTTCTGGAATTTAAGGGAGCTCCT

EcoRI

28481 CAAGGCGTTCCAATTAACACCAATAGCAGTCCAGATGACCAAATTGGCTACTACCGAAGAGCTACCAGACGAATTCGTGG 28560

GTTCCGCAAGGTTAATTGTGGTTATCGTCAGGTCTACTGGTTTAACCGATGATGGCTTCTCGATGGTCTGCTTAAGCACC

BglIIAvrII

28561 TGGTGACGGTAAAATGAAAGATCTCAGTCCAAGATGGTATTTCTACTACCTAGGAACTGGGCCAGAAGCTGGACTTCCCT 28640

ACCACTGCCATTTTACTTTCTAGAGTCAGGTTCTACCATAAAGATGATGGATCCTTGACCCGGTCTTCGACCTGAAGGGA

NdeI

28641 ATGGTGCTAACAAAGACGGCATCATATGGGTTGCAACTGAGGGAGCCTTGAATACACCAAAAGATCACATTGGCACCCGC 28720

TACCACGATTGTTTCTGCCGTAGTATACCCAACGTTGACTCCCTCGGAACTTATGTGGTTTTCTAGTGTAACCGTGGGCG

28721 AATCCTGCTAACAATGCTGCAATCGTGCTACAACTTCCTCAAGGAACAACATTGCCAAAAGGCTTCTACGCAGAAGGGAG 28800

TTAGGACGATTGTTACGACGTTAGCACGATGTTGAAGGAGTTCCTTGTTGTAACGGTTTTCCGAAGATGCGTCTTCCCTC

28801 CAGAGGCGGCAGTCAAGCCTCTTCTCGTTCCTCATCACGTAGTCGCAACAGTTCAAGAAATTCAACTCCAGGCAGCAGTA 28880

GTCTCCGCCGTCAGTTCGGAGAAGAGCAAGGAGTAGTGCATCAGCGTTGTCAAGTTCTTTAAGTTGAGGTCCGTCGTCAT

28881 GGGGAACTTCTCCTGCTAGAATGGCTGGCAATGGCGGTGATGCTGCTCTTGCTTTGCTGCTGCTTGACAGATTGAACCAG 28960

CCCCTTGAAGAGGACGATCTTACCGACCGTTACCGCCACTACGACGAGAACGAAACGACGACGAACTGTCTAACTTGGTC

28961 CTTGAGAGCAAAATGTCTGGTAAAGGCCAACAACAACAAGGCCAAACTGTCACTAAGAAATCTGCTGCTGAGGCTTCTAA 29040

GAACTCTCGTTTTACAGACCATTTCCGGTTGTTGTTGTTCCGGTTTGACAGTGATTCTTTAGACGACGACTCCGAAGATT

HindIII

29041 GAAGCCTCGGCAAAAACGTACTGCCACTAAAGCATACAATGTAACACAAGCTTTCGGCAGACGTGGTCCAGAACAAACCC 29120

CTTCGGAGCCGTTTTTGCATGACGGTGATTTCGTATGTTACATTGTGTTCGAAAGCCGTCTGCACCAGGTCTTGTTTGGG

29121 AAGGAAATTTTGGGGACCAGGAACTAATCAGACAAGGAACTGATTACAAACATTGGCCGCAAATTGCACAATTTGCCCCC 29200

TTCCTTTAAAACCCCTGGTCCTTGATTAGTCTGTTCCTTGACTAATGTTTGTAACCGGCGTTTAACGTGTTAAACGGGGG

AfeI

29201 AGCGCTTCAGCGTTCTTCGGAATGTCGCGCATTGGCATGGAAGTCACACCTTCGGGAACGTGGTTGACCTACACAGGTGC 29280

TCGCGAAGTCGCAAGAAGCCTTACAGCGCGTAACCGTACCTTCAGTGTGGAAGCCCTTGCACCAACTGGATGTGTCCACG

29281 CATCAAATTGGATGACAAAGATCCAAATTTCAAAGATCAAGTCATTTTGCTGAATAAGCATATTGACGCATACAAAACAT 29360

GTAGTTTAACCTACTGTTTCTAGGTTTAAAGTTTCTAGTTCAGTAAAACGACTTATTCGTATAACTGCGTATGTTTTGTA

29361 TCCCACCAACAGAGCCTAAAAAGGACAAAAAGAAGAAGGCTGATGAAACTCAAGCCTTACCGCAGAGACAGAAGAAACAG 29440

AGGGTGGTTGTCTCGGATTTTTCCTGTTTTTCTTCTTCCGACTACTTTGAGTTCGGAATGGCGTCTCTGTCTTCTTTGTC

PstIMfeI

29441 CAAACTGTGACTCTTCTTCCTGCTGCAGATTTGGATGATTTCTCCAAACAATTGCAACAATCCATGAGCAGTGCTGACTC 29520

GTTTGACACTGAGAAGAAGGACGACGTCTAAACCTACTAAAGAGGTTTGTTAACGTTGTTAGGTACTCGTCACGACTGAG

StuIAclI

29521 AACTCAGGCCTAAACTCATGCAGACCACACAAGGCAGATGGGCTATATAAACGTTTTCGCTTTTCCGTTTACGATATATA 29600

TTGAGTCCGGATTTGAGTACGTCTGGTGTGTTCCGTCTACCCGATATATTTGCAAAAGCGAAAAGGCAAATGCTATATAT

EcoRIHpaI

29601 GTCTACTCTTGTGCAGAATGAATTCTCGTAACTACATAGCACAAGTAGATGTAGTTAACTTTAATCTCACATAGCAATCT 29680

CAGATGAGAACACGTCTTACTTAAGAGCATTGATGTATCGTGTTCATCTACATCAATTGAAATTAGAGTGTATCGTTAGA

PvuIBsrGI

29681 TTAATCAGTGTGTAACATTAGGGAGGACTTGAAAGAGCCACCACATTTTCACCGAGGCCACGCGGAGTACGATCGAGTGT 29760

AATTAGTCACACATTGTAATCCCTCCTGAACTTTCTCGGTGGTGTAAAAGTGGCTCCGGTGCGCCTCATGCTAGCTCACA

AseI

29761 ACAGTGAACAATGCTAGGGAGAGCTGCCTATATGGAAGAGCCCTAATGTGTAAAATTAATTTTAGTAGTGCTATCCCCAT 29840

TGTCACTTGTTACGATCCCTCTCGACGGATATACCTTCTCGGGATTACACATTTTAATTAAAATCATCACGATAGGGGTA

29841 GTGATTTTAATAGCTTCTTAGGAGAATGACAAAAAAAAAAAAAAAAAAAAAAAAAAAAAAAAA 29903

CACTAAAATTATCGAAGAATCCTCTTACTGTTTTTTTTTTTTTTTTTTTTTTTTTTTTTTTTT
