# Supplementary material for: Whole Genome Analysis and Targeted Drug Discovery Using Computational Methods and High Throughput Screening Tools for Emerged Novel Coronavirus (2019-nCoV)
Source: J Pharm Drug Res. Author manuscript; Available in PMC 2020 Jul 2. (PMC7331973)
Supplement: supplement8SARS COV2 ALIGHNED WHOLE GENOME TO TRANSALTED ALIGHTMENT [file NIHMS1582187-supplement-supplement8SARS_COV2_ALIGHNED_WHOLE_GENOME_TO_TRANSALTED_ALIGHTMENT.docx]

IKGLYLPR*QTNQLSISCRSVL*TNFK

BglIIDraI

1 ATTAAAGGTTTATACCTTCCCAGGTAACAAACCAACCAACTTTCGATCTCTTGTAGATCTGTTCTCTAAACGAACTTTAA 80

TAATTTCCAAATATGGAAGGGTCCATTGTTTGGTTGGTTGAAAGCTAGAGAACATCTAGACAAGAGATTTGCTTGAAATT

ICVAVTRLHA*CTHAV*LITNYCR*QD

SphIApaLIAseI

81 AATCTGTGTGGCTGTCACTCGGCTGCATGCTTAGTGCACTCACGCAGTATAATTAATAACTAATTACTGTCGTTGACAGG 160

TTAGACACACCGACAGTGAGCCGACGTACGAATCACGTGAGTGCGTCATATTAATTATTGATTAATGACAGCAACTGTCC

TSNSSIFCRLLTVSSVLQPIISTSRF

PstI

161 ACACGAGTAACTCGTCTATCTTCTGCAGGCTGCTTACGGTTTCGTCCGTGTTGCAGCCGATCATCAGCACATCTAGGTTT 240

TGTGCTCATTGAGCAGATAGAAGACGTCCGACGAATGCCAAAGCAGGCACAACGTCGGCTAGTAGTCGTGTAGATCCAAA

RPGVTER*DGEPCPWFQRENTRPTQFA

241 CGTCCGGGTGTGACCGAAAGGTAAGATGGAGAGCCTTGTCCCTGGTTTCAACGAGAAAACACACGTCCAACTCAGTTTGC 320

GCAGGCCCACACTGGCTTTCCATTCTACCTCTCGGAACAGGGACCAAAGTTGCTCTTTTGTGTGCAGGTTGAGTCAAACG

CFTGSRRARTWLWRLRGGGLIRGTSTS

NruIBsiWI

321 CTGTTTTACAGGTTCGCGACGTGCTCGTACGTGGCTTTGGAGACTCCGTGGAGGAGGTCTTATCAGAGGCACGTCAACAT 400

GACAAAATGTCCAAGCGCTGCACGAGCATGCACCGAAACCTCTGAGGCACCTCCTCCAGAATAGTCTCCGTGCAGTTGTA

*RWHLWLSRS*KRRFAST*TALCVHQ

AclI

401 CTTAAAGATGGCACTTGTGGCTTAGTAGAAGTTGAAAAAGGCGTTTTGCCTCAACTTGAACAGCCCTATGTGTTCATCAA 480

GAATTTCTACCGTGAACACCGAATCATCTTCAACTTTTTCCGCAAAACGGAGTTGAACTTGTCGGGATACACAAGTAGTT

TFGCSNCTSWSCYG*AGSRTRRHSVRS

481 ACGTTCGGATGCTCGAACTGCACCTCATGGTCATGTTATGGTTGAGCTGGTAGCAGAACTCGAAGGCATTCAGTACGGTC 560

TGCAAGCCTACGAGCTTGACGTGGAGTACCAGTACAATACCAACTCGACCATCGTCTTGAGCTTCCGTAAGTCATGCCAG

*W*DTWCPCPSCGRNTSGLPQGSSS*E

561 GTAGTGGTGAGACACTTGGTGTCCTTGTCCCTCATGTGGGCGAAATACCAGTGGCTTACCGCAAGGTTCTTCTTCGTAAG 640

CATCACCACTCTGTGAACCACAGGAACAGGGAGTACACCCGCTTTATGGTCACCGAATGGCGTTCCAAGAAGAAGCATTC

R**RSWWP*LRRRSKVI*LRRRAWH*

MscINarI

641 AACGGTAATAAAGGAGCTGGTGGCCATAGTTACGGCGCCGATCTAAAGTCATTTGACTTAGGCGACGAGCTTGGCACTGA 720

TTGCCATTATTTCCTCGACCACCGGTATCAATGCCGCGGCTAGATTTCAGTAAACTGAATCCGCTGCTCGAACCGTGACT

SL*RFSRKLEH*T*QWCYP*THA*A*R

721 TCCTTATGAAGATTTTCAAGAAAACTGGAACACTAAACATAGCAGTGGTGTTACCCGTGAACTCATGCGTGAGCTTAACG 800

AGGAATACTTCTAAAAGTTCTTTTGACCTTGTGATTTGTATCGTCACCACAATGGGCACTTGAGTACGCACTCGAATTGC

RGIHSLCR*QLLWP*WLPS*VH*RPSS

801 GAGGGGCATACACTCGCTATGTCGATAACAACTTCTGTGGCCCTGATGGCTACCCTCTTGAGTGCATTAAAGACCTTCTA 880

CTCCCCGTATGTGAGCGATACAGCTATTGTTGAAGACACCGGGACTACCGATGGGAGAACTCACGTAATTTCTGGAAGAT

TCW*SFMHFVRTTGLY*H*EGCILLP

PmlIHindIII

881 GCACGTGCTGGTAAAGCTTCATGCACTTTGTCCGAACAACTGGACTTTATTGACACTAAGAGGGGTGTATACTGCTGCCG 960

CGTGCACGACCATTTCGAAGTACGTGAAACAGGCTTGTTGACCTGAAATAACTGTGATTCTCCCCACATATGACGACGGC

*T*A*NCLVHGTF*KEL*IADTF*N*I

AclI

961 TGAACATGAGCATGAAATTGCTTGGTACACGGAACGTTCTGAAAAGAGCTATGAATTGCAGACACCTTTTGAAATTAAAT 1040

ACTTGTACTCGTACTTTAACGAACCATGTGCCTTGCAAGACTTTTCTCGATACTTAACGTCTGTGGAAAACTTTAATTTA

GKEI*HLQWGMSKFCISLKFHNQDYST

1041 TGGCAAAGAAATTTGACACCTTCAATGGGGAATGTCCAAATTTTGTATTTCCCTTAAATTCCATAATCAAGACTATTCAA 1120

ACCGTTTCTTTAAACTGTGGAAGTTACCCCTTACAGGTTTAAAACATAAAGGGAATTTAAGGTATTAGTTCTGATAAGTT

KG*KEKA*WLYG*NSICLSSCVTK*M

HindIIIEcoRI

1121 CCAAGGGTTGAAAAGAAAAAGCTTGATGGCTTTATGGGTAGAATTCGATCTGTCTATCCAGTTGCGTCACCAAATGAATG 1200

GGTTCCCAACTTTTCTTTTTCGAACTACCGAAATACCCATCTTAAGCTAGACAGATAGGTCAACGCAGTGGTTTACTTAC

QPNVPFNSHEV*SLW*NFMADGRFC*S

BspHI

1201 CAACCAAATGTGCCTTTCAACTCTCATGAAGTGTGATCATTGTGGTGAAACTTCATGGCAGACGGGCGATTTTGTTAAAG 1280

GTTGGTTTACACGGAAAGTTGAGAGTACTTCACACTAGTAACACCACTTTGAAGTACCGTCTGCCCGCTAAAACAATTTC

HLRILWH*EFD*RRCHYLWLLTPKCCC

1281 CCACTTGCGAATTTTGTGGCACTGAGAATTTGACTAAAGAAGGTGCCACTACTTGTGGTTACTTACCCCAAAATGCTGTT 1360

GGTGAACGCTTAAAACACCGTGACTCTTAAACTGATTTCTTCCACGGTGATGAACACCAATGAATGGGGTTTTACGACAA

*NLLSSMSQFRSRT*A*SCRIP**IW

1361 GTTAAAATTTATTGTCCAGCATGTCACAATTCAGAAGTAGGACCTGAGCATAGTCTTGCCGAATACCATAATGAATCTGG 1440

CAATTTTAAATAACAGGTCGTACAGTGTTAAGTCTTCATCCTGGACTCGTATCAGAACGGCTTATGGTATTACTTAGACC

LENHSS*GWSHYCLWRLCVLLCWLP*Q

1441 CTTGAAAACCATTCTTCGTAAGGGTGGTCGCACTATTGCCTTTGGAGGCTGTGTGTTCTCTTATGTTGGTTGCCATAACA 1520

GAACTTTTGGTAAGAAGCATTCCCACCAGCGTGATAACGGAAACCTCCGACACACAAGAGAATACAACCAACGGTATTGT

VCLLGSTC*R*HRL*PYRCCWRRFRRS

PmlINheIAfeI

1521 AGTGTGCCTATTGGGTTCCACGTGCTAGCGCTAACATAGGTTGTAACCATACAGGTGTTGTTGGAGAAGGTTCCGAAGGT 1600

TCACACGGATAACCCAAGGTGCACGATCGCGATTGTATCCAACATTGGTATGTCCACAACAACCTCTTCCAAGGCTTCCA

**QPS*NTPKRESQHQYCW*L*T**R

SspIDraI

1601 CTTAATGACAACCTTCTTGAAATACTCCAAAAAGAGAAAGTCAACATCAATATTGTTGGTGACTTTAAACTTAATGAAGA 1680

GAATTACTGTTGGAAGAACTTTATGAGGTTTTTCTCTTTCAGTTGTAGTTATAACAACCACTGAAATTTGAATTACTTCT

DRHYFGIFFCFHKCFCGNCERFGL*SI

PsiI

1681 GATCGCCATTATTTTGGCATCTTTTTCTGCTTCCACAAGTGCTTTTGTGGAAACTGTGAAAGGTTTGGATTATAAAGCAT 1760

CTAGCGGTAATAAAACCGTAGAAAAAGACGAAGGTGTTCACGAAAACACCTTTGACACTTTCCAAACCTAATATTTCGTA

QTNC*ILW*F*SYKRKS*KRCLEYW*T

DraISspI

1761 TCAAACAAATTGTTGAATCCTGTGGTAATTTTAAAGTTACAAAAGGAAAAGCTAAAAAAGGTGCCTGGAATATTGGTGAA 1840

AGTTTGTTTAACAACTTAGGACACCATTAAAATTTCAATGTTTTCCTTTTCGATTTTTTCCACGGACCTTATAACCACTT

EINTESSLCICIRGCSCCTINFLPHS

NsiI

1841 CAGAAATCAATACTGAGTCCTCTTTATGCATTTGCATCAGAGGCTGCTCGTGTTGTACGATCAATTTTCTCCCGCACTCT 1920

GTCTTTAGTTATGACTCAGGAGAAATACGTAAACGTAGTCTCCGACGAGCACAACATGCTAGTTAAAAGAGGGCGTGAGA

*NCSKFCACFTEGRYNNTRWNFTVFTE

1921 TGAAACTGCTCAAAATTCTGTGCGTGTTTTACAGAAGGCCGCTATAACAATACTAGATGGAATTTCACAGTATTCACTGA 2000

ACTTTGACGAGTTTTAAGACACGCACAAAATGTCTTCCGGCGATATTGTTATGATCTACCTTAAAGTGTCATAAGTGACT

TH*CYDVHI*FGY*QSSCNGLHYRWCC

2001 GACTCATTGATGCTATGATGTTCACATCTGATTTGGCTACTAACAATCTAGTTGTAATGGCCTACATTACAGGTGGTGTT 2080

CTGAGTAACTACGATACTACAAGTGTAGACTAAACCGATGATTGTTAGATCAACATTACCGGATGTAATGTCCACCACAA

SVDFAVAN*HLWHCL*KTQTRP*LA*

2081 GTTCAGTTGACTTCGCAGTGGCTAACTAACATCTTTGGCACTGTTTATGAAAAACTCAAACCCGTCCTTGATTGGCTTGA 2160

CAAGTCAACTGAAGCGTCACCGATTGATTGTAGAAACCGTGACAAATACTTTTTGAGTTTGGGCAGGAACTAACCGAACT

REV*GRCRVS*RRLGNC*IYLNLCL*N

2161 AGAGAAGTTTAAGGAAGGTGTAGAGTTTCTTAGAGACGGTTGGGAAATTGTTAAATTTATCTCAACCTGTGCTTGTGAAA 2240

TCTCTTCAAATTCCTTCCACATCTCAAAGAATCTCTGCCAACCCTTTAACAATTTAAATAGAGTTGGACACGAACACTTT

CRWTNCHLCKGN*GECSDIL*ACK*IF

HindIII

2241 TTGTCGGTGGACAAATTGTCACCTGTGCAAAGGAAATTAAGGAGAGTGTTCAGACATTCTTTAAGCTTGTAAATAAATTT 2320

AACAGCCACCTGTTTAACAGTGGACACGTTTCCTTTAATTCCTCTCACAAGTCTGTAAGAAATTCGAACATTTATTTAAA

GFVC*LYHYWWS*T*SLEFR*NICHA

2321 TTGGCTTTGTGTGCTGACTCTATCATTATTGGTGGAGCTAAACTTAAAGCCTTGAATTTAGGTGAAACATTTGTCACGCA 2400

AACCGAAACACACGACTGAGATAGTAATAACCACCTCGATTTGAATTTCGGAACTTAAATCCACTTTGTAAACAGTGCGT

LKGIVQKVC*IQRRNWPTHASKSPKRN

BsrGI

2401 CTCAAAGGGATTGTACAGAAAGTGTGTTAAATCCAGAGAAGAAACTGGCCTACTCATGCCTCTAAAAGCCCCAAAAGAAA 2480

GAGTTTCCCTAACATGTCTTTCACACAATTTAGGTCTCTTCTTTGACCGGATGAGTACGGAGATTTTCGGGGTTTTCTTT

YLLRGRNTSHRSVNRGSCLENW*FTTI

HpaI

2481 TTATCTTCTTAGAGGGAGAAACACTTCCCACAGAAGTGTTAACAGAGGAAGTTGTCTTGAAAACTGGTGATTTACAACCA 2560

AATAGAAGAATCTCCCTCTTTGTGAAGGGTGTCTTCACAATTGTCTCCTTCAACAGAACTTTTGACCACTAAATGTTGGT

RTTY**SC*SSIGWYTSLY*RAYVAR

SpeI

2561 TTAGAACAACCTACTAGTGAAGCTGTTGAAGCTCCATTGGTTGGTACACCAGTTTGTATTAACGGGCTTATGTTGCTCGA 2640

AATCTTGTTGGATGATCACTTCGACAACTTCGAGGTAACCAACCATGTGGTCAAACATAATTGCCCGAATACAACGAGCT

NQRHRKVLCPCT*YDGNKQYLHTQRRC

ScaIApaLI

2641 AATCAAAGACACAGAAAAGTACTGTGCCCTTGCACCTAATATGATGGTAACAAACAATACCTTCACACTCAAAGGCGGTG 2720

TTAGTTTCTGTGTCTTTTCATGACACGGGAACGTGGATTATACTACCATTGTTTGTTATGGAAGTGTGAGTTTCCGCCAC

TNKGYFW**HCDRSARLQECEYHF*T*

2721 CACCAACAAAGGTTACTTTTGGTGATGACACTGTGATAGAAGTGCAAGGTTACAAGAGTGTGAATATCACTTTTGAACTT 2800

GTGGTTGTTTCCAATGAAAACCACTACTGTGACACTATCTTCACGTTCCAATGTTCTCACACTTATAGTGAAAACTTGAA

*KD**ST**EVLCLYS*TRYRSK*VR

ScaI

2801 GATGAAAGGATTGATAAAGTACTTAATGAGAAGTGCTCTGCCTATACAGTTGAACTCGGTACAGAAGTAAATGAGTTCGC 2880

CTACTTTCCTAACTATTTCATGAATTACTCTTCACGAGACGGATATGTCAACTTGAGCCATGTCTTCATTTACTCAAGCG

LCCGRCCHKNFATSI*ITYTTGH*FR*

2881 CTGTGTTGTGGCAGATGCTGTCATAAAAACTTTGCAACCAGTATCTGAATTACTTACACCACTGGGCATTGATTTAGATG 2960

GACACAACACCGTCTACGACAGTATTTTTGAAACGTTGGTCATAGACTTAATGAATGTGGTGACCCGTAACTAAATCTAC

VEYGYILLI**VW*V*IGFTYVLFFLP

DraINdeI

2961 AGTGGAGTATGGCTACATACTACTTATTTGATGAGTCTGGTGAGTTTAAATTGGCTTCACATATGTATTGTTCTTTCTAC 3040

TCACCTCATACCGATGTATGATGAATAAACTACTCAGACCACTCAAATTTAACCGAAGTGTATACATAACAAGAAAGATG

SR*G*RRR*L*RRRV*AINSI*VWY*

3041 CCTCCAGATGAGGATGAAGAAGAAGGTGATTGTGAAGAAGAAGAGTTTGAGCCATCAACTCAATATGAGTATGGTACTGA 3120

GGAGGTCTACTCCTACTTCTTCTTCCACTAACACTTCTTCTTCTCAAACTCGGTAGTTGAGTTATACTCATACCATGACT

R*LPR*TFGIWCHFCCSST*RRARRRL

3121 AGATGATTACCAAGGTAAACCTTTGGAATTTGGTGCCACTTCTGCTGCTCTTCAACCTGAAGAAGAGCAAGAAGAAGATT 3200

TCTACTAATGGTTCCATTTGGAAACCTTAAACCACGGTGAAGACGACGAGAAGTTGGACTTCTTCTCGTTCTTCTTCTAA

VR***STNCWSTRRQ*GQSDNYYSNNC

MfeI

3201 GGTTAGATGATGATAGTCAACAAACTGTTGGTCAACAAGACGGCAGTGAGGACAATCAGACAACTACTATTCAAACAATT 3280

CCAATCTACTACTATCAGTTGTTTGACAACCAGTTGTTCTGCCGTCACTCCTGTTAGTCTGTTGATGATAAGTTTGTTAA

*GSTSIRDGTYTSCSDY*SE*F*WLF

DraI

3281 GTTGAGGTTCAACCTCAATTAGAGATGGAACTTACACCAGTTGTTCAGACTATTGAAGTGAATAGTTTTAGTGGTTATTT 3360

CAACTCCAAGTTGGAGTTAATCTCTACCTTGAATGTGGTCAACAAGTCTGATAACTTCACTTATCAAAATCACCAATAAA

KTY*QCIH*KCRHCGRS*KGKTNSGC*

3361 AAAACTTACTGACAATGTATACATTAAAAATGCAGACATTGTGGAAGAAGCTAAAAAGGTAAAACCAACAGTGGTTGTTA 3440

TTTTGAATGACTGTTACATATGTAATTTTTACGTCTGTAACACCTTCTTCGATTTTTCCATTTTGGTTGTCACCAACAAT

CSQCLP*TWRRCCRSLK*GY*QCHAS*

3441 ATGCAGCCAATGTTTACCTTAAACATGGAGGAGGTGTTGCAGGAGCCTTAAATAAGGCTACTAACAATGCCATGCAAGTT 3520

TACGTCGGTTACAAATGGAATTTGTACCTCCTCCACAACGTCCTCGGAATTTATTCCGATGATTGTTACGGTACGTTCAA

I**LHSY*WTT*SGW*LCFKRTQSC*

3521 GAATCTGATGATTACATAGCTACTAATGGACCACTTAAAGTGGGTGGTAGTTGTGTTTTAAGCGGACACAATCTTGCTAA 3600

CTTAGACTACTAATGTATCGATGATTACCTGGTGAATTTCACCCACCATCAACACAAAATTCGCCTGTGTTAGAACGATT

TLSSCCRPKC*QR*RHSTS*ECL*KF*

HpaIAflII

3601 ACACTGTCTTCATGTTGTCGGCCCAAATGTTAACAAAGGTGAAGACATTCAACTTCTTAAGAGTGCTTATGAAAATTTTA 3680

TGTGACAGAAGTACAACAGCCGGGTTTACAATTGTTTCCACTTCTGTAAGTTGAAGAATTCTCACGAATACTTTTAAAAT

SARSSTCTIIISWYFWC*PYTFFKSLC

PvuII

3681 ATCAGCACGAAGTTCTACTTGCACCATTATTATCAGCTGGTATTTTTGGTGCTGACCCTATACATTCTTTAAGAGTTTGT 3760

TAGTCGTGCTTCAAGATGAACGTGGTAATAATAGTCGACCATAAAAACCACGACTGGGATATGTAAGAAATTCTCAAACA

RYCSHKCLLSCL**KSL*QTCFKLFG

HindIII

3761 GTAGATACTGTTCGCACAAATGTCTACTTAGCTGTCTTTGATAAAAATCTCTATGACAAACTTGTTTCAAGCTTTTTGGA 3840

CATCTATGACAAGCGTGTTTACAGATGAATCGACAGAAACTATTTTTAGAGATACTGTTTGAACAAAGTTCGAAAAACCT

NEE*KAS*TKDR*DS*RGS*AIYN*K*

PsiI

3841 AATGAAGAGTGAAAAGCAAGTTGAACAAAAGATCGCTGAGATTCCTAAAGAGGAAGTTAAGCCATTTATAACTGAAAGTA 3920

TTACTTCTCACTTTTCGTTCAACTTGTTTTCTAGCGACTCTAAGGATTTCTCCTTCAATTCGGTAAATATTGACTTTCAT

TFS*TEKTR**ENQSLC*RSYNNSGRN

HindIII

3921 AACCTTCAGTTGAACAGAGAAAACAAGATGATAAGAAAATCAAAGCTTGTGTTGAAGAAGTTACAACAACTCTGGAAGAA 4000

TTGGAAGTCAACTTGTCTCTTTTGTTCTACTATTCTTTTAGTTTCGAACACAACTTCTTCAATGTTGTTGAGACCTTCTT

*VPHRKLVTLY*H*WQSSSRFCHSC*

AseI

4001 ACTAAGTTCCTCACAGAAAACTTGTTACTTTATATTGACATTAATGGCAATCTTCATCCAGATTCTGCCACTCTTGTTAG 4080

TGATTCAAGGAGTGTCTTTTGAACAATGAAATATAACTGTAATTACCGTTAGAAGTAGGTCTAAGACGGTGAGAACAATC

*H*HHFLKERCSIYSG*CCSRGCFNCC

4081 TGACATTGACATCACTTTCTTAAAGAAAGATGCTCCATATATAGTGGGTGATGTTGTTCAAGAGGGTGTTTTAACTGCTG 4160

ACTGTAACTGTAGTGAAAGAATTTCTTTCTACGAGGTATATATCACCCACTACAACAAGTTCTCCCACAAAATTGACGAC

GYTY*KGWWHY*NASESFEKSANRQLY

NheIHindIII

4161 TGGTTATACCTACTAAAAAGGCTGGTGGCACTACTGAAATGCTAGCGAAAGCTTTGAGAAAAGTGCCAACAGACAATTAT 4240

ACCAATATGGATGATTTTTCCGACCACCGTGATGACTTTACGATCGCTTTCGAAACTCTTTTCACGGTTGTCTGTTAATA

NHLPGSGFKWLHCRGGKDSA*KV*KC

SmaIDraI

4241 ATAACCACTTACCCGGGTCAGGGTTTAAATGGTTACACTGTAGAGGAGGCAAAGACAGTGCTTAAAAAGTGTAAAAGTGC 4320

TATTGGTGAATGGGCCCAGTCCCAAATTTACCAATGTGACATCTCCTCCGTTTCTGTCACGAATTTTTCACATTTTCACG

LLHSTIYYL**EARNSWNCFLEFARNA

4321 CTTTTACATTCTACCATCTATTATCTCTAATGAGAAGCAAGAAATTCTTGGAACTGTTTCTTGGAATTTGCGAGAAATGC 4400

GAAAATGTAAGATGGTAGATAATAGAGATTACTCTTCGTTCTTTAAGAACCTTGACAAAGAACCTTAAACGCTCTTTACG

CTCRRNTQINACLCGN*SHSFNYTA*I

AseI

4401 TTGCACATGCAGAAGAAACACGCAAATTAATGCCTGTCTGTGTGGAAACTAAAGCCATAGTTTCAACTATACAGCGTAAA 4480

AACGTGTACGTCTTCTTTGTGCGTTTAATTACGGACAGACACACCTTTGATTTCGGTATCAAAGTTGATATGTCGCATTT

*GY*NTRGCG*LWC*ILLLHQ*NNCS

4481 TATAAGGGTATTAAAATACAAGAGGGTGTGGTTGATTATGGTGCTAGATTTTACTTTTACACCAGTAAAACAACTGTAGC 4560

ATATTCCCATAATTTTATGTTCTCCCACACCAACTAATACCACGATCTAAAATGAAAATGTGGTCATTTTGTTGACATCG

VTYQHT*RSK*NSCYNATWLCNTWLKF

4561 GTCACTTATCAACACACTTAACGATCTAAATGAAACTCTTGTTACAATGCCACTTGGCTATGTAACACATGGCTTAAATT 4640

CAGTGAATAGTTGTGTGAATTGCTAGATTTACTTTGAGAACAATGTTACGGTGAACCGATACATTGTGTACCGAATTTAA

GRSCSVYEISQSASYSFCFFT*CCYSV

BglII

4641 TGGAAGAAGCTGCTCGGTATATGAGATCTCTCAAAGTGCCAGCTACAGTTTCTGTTTCTTCACCTGATGCTGTTACAGCG 4720

ACCTTCTTCGACGAGCCATATACTCTAGAGAGTTTCACGGTCGATGTCAAAGACAAAGAAGTGGACTACGACAATGTCGC

*WLSYFFF*NT*RTFY*NHLTCWFL*

4721 TATAATGGTTATCTTACTTCTTCTTCTAAAACACCTGAAGAACATTTTATTGAAACCATCTCACTTGCTGGTTCCTATAA 4800

ATATTACCAATAGAATGAAGAAGAAGATTTTGTGGACTTCTTGTAAAATAACTTTGGTAGAGTGAACGACCAAGGATATT

RLVLFWTIYTTRYRIS*ER**KCILH*

AflIISpeI

4801 AGATTGGTCCTATTCTGGACAATCTACACAACTAGGTATAGAATTTCTTAAGAGAGGTGATAAAAGTGTATATTACACTA 4880

TCTAACCAGGATAAGACCTGTTAGATGTGTTGATCCATATCTTAAAGAATTCTCTCCACTATTTTCACATATAATGTGAT

*SYHIPPRW*SYHL*QS*DTSFFERSE

AflII

4881 GTAATCCTACCACATTCCACCTAGATGGTGAAGTTATCACCTTTGACAATCTTAAGACACTTCTTTCTTTGAGAGAAGTG 4960

CATTAGGATGGTGTAAGGTGGATCTACCACTTCAATAGTGGAAACTGTTAGAATTCTGTGAAGAAAGAAACTCTCTTCAC

DY*GVYNSRQH*PPHASCGHVNDIWT

NdeI

4961 AGGACTATTAAGGTGTTTACAACAGTAGACAACATTAACCTCCACACGCAAGTTGTGGACATGTCAATGACATATGGACA 5040

TCCTGATAATTCCACAAATGTTGTCATCTGTTGTAATTGGAGGTGTGCGTTCAACACCTGTACAGTTACTGTATACCTGT

TVWSNLFGWS*CY*NKTS*FT*R*NIL

5041 ACAGTTTGGTCCAACTTATTTGGATGGAGCTGATGTTACTAAAATAAAACCTCATAATTCACATGAAGGTAAAACATTTT 5120

TGTCAAACCAGGTTGAATAAACCTACCTCGACTACAATGATTTTATTTTGGAGTATTAAGTGTACTTCCATTTTGTAAAA

CFT***HSTC*GF*VLPHN*S*FSG*V

ScaI

5121 ATGTTTTACCTAATGATGACACTCTACGTGTTGAGGCTTTTGAGTACTACCACACAACTGATCCTAGTTTTCTGGGTAGG 5200

TACAAAATGGATTACTACTGTGAGATGCACAACTCCGAAAACTCATGATGGTGTGTTGACTAGGATCAAAAGACCCATCC

HVSIKSH*KVEIPTS*WFNFY*MGR*

5201 TACATGTCAGCATTAAATCACACTAAAAAGTGGAAATACCCACAAGTTAATGGTTTAACTTCTATTAAATGGGCAGATAA 5280

ATGTACAGTCGTAATTTAGTGTGATTTTTCACCTTTATGGGTGTTCAATTACCAAATTGAAGATAATTTACCCGTCTATT

QLLSCHCIVNTPTNRVEV*STCSTRCL

HpaI

5281 CAACTGTTATCTTGCCACTGCATTGTTAACACTCCAACAAATAGAGTTGAAGTTTAATCCACCTGCTCTACAAGATGCTT 5360

GTTGACAATAGAACGGTGACGTAACAATTGTGAGGTTGTTTATCTCAACTTCAAATTAGGTGGACGAGATGTTCTACGAA

LQSKGW*SC*LLCTYLSLL**DSR*VR

ApaLI

5361 ATTACAGAGCAAGGGCTGGTGAAGCTGCTAACTTTTGTGCACTTATCTTAGCCTACTGTAATAAGACAGTAGGTGAGTTA 5440

TAATGTCTCGTTCCCGACCACTTCGACGATTGAAAACACGTGAATAGAATCGGATGACATTATTCTGTCATCCACTCAAT

*C*RNNELLVSTCQFRFLQKSLERGV

5441 GGTGATGTTAGAGAAACAATGAGTTACTTGTTTCAACATGCCAATTTAGATTCTTGCAAAAGAGTCTTGAACGTGGTGTG 5520

CCACTACAATCTCTTTGTTACTCAATGAACAAAGTTGTACGGTTAAATCTAAGAACGTTTTCTCAGAACTTGCACCACAC

*NLWTTADNP*GCRSCYVHGHTFL*TI

AflIIBsrGI

5521 TAAAACTTGTGGACAACAGCAGACAACCCTTAAGGGTGTAGAAGCTGTTATGTACATGGGCACACTTTCTTATGAACAAT 5600

ATTTTGAACACCTGTTGTCGTCTGTTGGGAATTCCCACATCTTCGACAATACATGTACCCGTGTGAAAGAATACTTGTTA

*ERCSDTLYVW*TSYKISSTTGVTFCY

5601 TTAAGAAAGGTGTTCAGATACCTTGTACGTGTGGTAAACAAGCTACAAAATATCTAGTACAACAGGAGTCACCTTTTGTT 5680

AATTCTTTCCACAAGTCTATGGAACATGCACACCATTTGTTCGATGTTTTATAGATCATGTTGTCCTCAGTGGAAAACAA

DVSTTCSV*T*AWYIYLC**VHW*LP

AflII

5681 ATGATGTCAGCACCACCTGCTCAGTATGAACTTAAGCATGGTACATTTACTTGTGCTAGTGAGTACACTGGTAATTACCA 5760

TACTACAGTCGTGGTGGACGAGTCATACTTGAATTCGTACCATGTAAATGAACACGATCACTCATGTGACCATTAATGGT

VWSL*TYNF*RNFVLHRRCFTYKVLRI

5761 GTGTGGTCACTATAAACATATAACTTCTAAAGAAACTTTGTATTGCATAGACGGTGCTTTACTTACAAAGTCCTCAGAAT 5840

CACACCAGTGATATTTGTATATTGAAGATTTCTTTGAAACATAACGTATCTGCCACGAAATGAATGTTTCAGGAGTCTTA

QRSYYGCFLQRKQLHNNHKTSYL*IGW

PsiI

5841 ACAAAGGTCCTATTACGGATGTTTTCTACAAAGAAAACAGTTACACAACAACCATAAAACCAGTTACTTATAAATTGGAT 5920

TGTTTCCAGGATAATGCCTACAAAAGATGTTTCTTTTGTCAATGTGTTGTTGGTATTTTGGTCAATGAATATTTAACCTA

CCLYRN*P*VGQLL*ERQFLFHRATN

BsrGIPsiIMfeI

5921 GGTGTTGTTTGTACAGAAATTGACCCTAAGTTGGACAATTATTATAAGAAAGACAATTCTTATTTCACAGAGCAACCAAT 6000

CCACAACAAACATGTCTTTAACTGGGATTCAACCTGTTAATAATATTCTTTCTGTTAAGAATAAAGTGTCTCGTTGGTTA

*SCTKPTISKRKLR*F*VCM**YQIC*

HindIII

6001 TGATCTTGTACCAAACCAACCATATCCAAACGCAAGCTTCGATAATTTTAAGTTTGTATGTGATAATATCAAATTTGCTG 6080

ACTAGAACATGGTTTGGTTGGTATAGGTTTGCGTTCGAAGCTATTAAAATTCAAACATACACTATTATAGTTTAAACGAC

*FKPVNWL*ETCFKRA*SYIFP*LKW*

DraIHpaIPsiI

6081 ATGATTTAAACCAGTTAACTGGTTATAAGAAACCTGCTTCAAGAGAGCTTAAAGTTACATTTTTCCCTGACTTAAATGGT 6160

TACTAAATTTGGTCAATTGACCAATATTCTTTGGACGAAGTTCTCTCGAATTTCAATGTAAAAAGGGACTGAATTTACCA

CGGY*L*TLHTLF*ERS*IVT*TYCL

PsiI

6161 GATGTGGTGGCTATTGATTATAAACACTACACACCCTCTTTTAAGAAAGGAGCTAAATTGTTACATAAACCTATTGTTTG 6240

CTACACCACCGATAACTAATATTTGTGATGTGTGGGAGAAAATTCTTTCCTCGATTTAACAATGTATTTGGATAACAAAC

AC*QCN**SHV*TKYLVYTLSLEHKTS

HpaI

6241 GCATGTTAACAATGCAACTAATAAAGCCACGTATAAACCAAATACCTGGTGTATACGTTGTCTTTGGAGCACAAAACCAG 6320

CGTACAATTGTTACGTTGATTATTTCGGTGCATATTTGGTTTATGGACCACATATGCAACAGAAACCTCGTGTTTTGGTC

*NIKFV*CTEVRGRAGNG*SCLRRSKT

BglII

6321 TTGAAACATCAAATTCGTTTGATGTACTGAAGTCAGAGGACGCGCAGGGAATGGATAATCTTGCCTGCGAAGATCTAAAA 6400

AACTTTGTAGTTTAAGCAAACTACATGACTTCAGTCTCCTGCGCGTCCCTTACCTATTAGAACGGACGCTTCTAGATTTT

SL*RSSGKSYHTERRS*V*CENYRSC

6401 CCAGTCTCTGAAGAAGTAGTGGAAAATCCTACCATACAGAAAGACGTTCTTGAGTGTAATGTGAAAACTACCGAAGTTGT 6480

GGTCAGAGACTTCTTCATCACCTTTTAGGATGGTATGTCTTTCTGCAAGAACTCACATTACACTTTTGATGGCTTCAACA

RRHYT*TSK**FKNYRRGWPHRSNGCL

DraIMscIBglII

6481 AGGAGACATTATACTTAAACCAGCAAATAATAGTTTAAAAATTACAGAAGAGGTTGGCCACACAGATCTAATGGCTGCTT 6560

TCCTCTGTAATATGAATTTGGTCGTTTATTATCAAATTTTTAATGTCTTCTCCAACCGGTGTGTCTAGATTACCGACGAA

CRQF*SYY*ET**II*SIRFENPCYSW

XbaI

6561 ATGTAGACAATTCTAGTCTTACTATTAAGAAACCTAATGAATTATCTAGAGTATTAGGTTTGAAAACCCTTGCTACTCAT 6640

TACATCTGTTAAGATCAGAATGATAATTCTTTGGATTACTTAATAGATCTCATAATCCAAACTTTTGGGAACGATGAGTA

FSCC**CPLGYYS*LC*AFS*QSC*Y

6641 GGTTTAGCTGCTGTTAATAGTGTCCCTTGGGATACTATAGCTAATTATGCTAAGCCTTTTCTTAACAAAGTTGTTAGTAC 6720

CCAAATCGACGACAATTATCACAGGGAACCCTATGATATCGATTAATACGATTCGGAAAAGAATTGTTTCAACAATCATG

NY*HSYTVFKPCLY*LYALFLYFIATI

PmeI/DraIMfeI

6721 AACTACTAACATAGTTACACGGTGTTTAAACCGTGTTTGTACTAATTATATGCCTTATTTCTTTACTTTATTGCTACAAT 6800

TTGATGATTGTATCAATGTGCCACAAATTTGGCACAAACATGATTAATATACGGAATAAAGAAATGAAATAACGATGTTA

VYFY*KYKF*N*SIYADYYSKEYC*EC

XbaI

6801 TGTGTACTTTTACTAGAAGTACAAATTCTAGAATTAAAGCATCTATGCCGACTACTATAGCAAAGAATACTGTTAAGAGT 6880

ACACATGAAAATGATCTTCATGTTTAAGATCTTAATTTCGTAGATACGGCTGATGATATCGTTTCTTATGACAATTCTCA

R*ILSRGFI*LFEVT*FF*TDKYYNL

XbaISspIPsiI

6881 GTCGGTAAATTTTGTCTAGAGGCTTCATTTAATTATTTGAAGTCACCTAATTTTTCTAAACTGATAAATATTATAATTTG 6960

CAGCCATTTAAAACAGATCTCCGAAGTAAATTAATAAACTTCAGTGGATTAAAAAGATTTGACTATTTATAATATTAAAC

VFTIKCLPRFFNLLNRCFRCFNV*FRH

AvrIISphI

6961 GTTTTTACTATTAAGTGTTTGCCTAGGTTCTTTAATCTACTCAACCGCTGCTTTAGGTGTTTTAATGTCTAATTTAGGCA 7040

CAAAAATGATAATTCACAAACGGATCCAAGAAATTAGATGAGTTGGCGACGAAATCCACAAAATTACAGATTAAATCCGT

AFLLYWLQRRLFELY*CHYCNLLYWFY

7041 TGCCTTCTTACTGTACTGGTTACAGAGAAGGCTATTTGAACTCTACTAATGTCACTATTGCAACCTACTGTACTGGTTCT 7120

ACGGAAGAATGACATGACCAATGTCTCTTCCGATAAACTTGAGATGATTACAGTGATAACGTTGGATGACATGACCAAGA

TL*CLS*WFRFFRHLSFFRNYTNYHF

7121 ATACCTTGTAGTGTTTGTCTTAGTGGTTTAGATTCTTTAGACACCTATCCTTCTTTAGAAACTATACAAATTACCATTTC 7200

TATGGAACATCACAAACAGAATCACCAAATCTAAGAAATCTGTGGATAGGAAGAAATCTTTGATATGTTTAATGGTAAAG

IF*MGFNCFWLSCRVVFGIYSFH*VFL

DraI

7201 ATCTTTTAAATGGGATTTAACTGCTTTTGGCTTAGTTGCAGAGTGGTTTTTGGCATATATTCTTTTCACTAGGTTTTTCT 7280

TAGAAAATTTACCCTAAATTGACGAAAACCGAATCAACGTCTCACCAAAAACCGTATATAAGAAAAGTGATCCAAAAAGA

CTWIGCNHAIVFQLFCSTFY**FLAYV

MfeI

7281 ATGTACTTGGATTGGCTGCAATCATGCAATTGTTTTTCAGCTATTTTGCAGTACATTTTATTAGTAATTCTTGGCTTATG 7360

TACATGAACCTAACCGACGTTAGTACGTTAACAAAAAGTCGATAAAACGTCATGTAAAATAATCATTAAGAACCGAATAC

VNN*SCTNGPDFSYG*NVHLLCIILL

AseIBsrGIBsrGI

7361 TGGTTAATAATTAATCTTGTACAAATGGCCCCGATTTCAGCTATGGTTAGAATGTACATCTTCTTTGCATCATTTTATTA 7440

ACCAATTATTAATTAGAACATGTTTACCGGGGCTAAAGTCGATACCAATCTTACATGTAGAAGAAACGTAGTAAAATAAT

CMEKLCACCRRL*FINLYDVLQT**SN

7441 TGTATGGAAAAGTTATGTGCATGTTGTAGACGGTTGTAATTCATCAACTTGTATGATGTGTTACAAACGTAATAGAGCAA 7520

ACATACCTTTTCAATACACGTACAACATCTGCCAACATTAAGTAGTTGAACATACTACACAATGTTTGCATTATCTCGTT

KSRMYNYC*WC*KVLLCLC*WR*RLLQ

BsrGI

7521 CAAGAGTCGAATGTACAACTATTGTTAATGGTGTTAGAAGGTCCTTTTATGTCTATGCTAATGGAGGTAAAGGCTTTTGC 7600

GTTCTCAGCTTACATGTTGATAACAATTACCACAATCTTCCAGGAAAATACAGATACGATTACCTCCATTTCCGAAAACG

TTQLELC*L*YILCW*YIY***SCER

MfeI

7601 AAACTACACAATTGGAATTGTGTTAATTGTGATACATTCTGTGCTGGTAGTACATTTATTAGTGATGAAGTTGCGAGAGA 7680

TTTGATGTGTTAACCTTAACACAATTAACACTATGTAAGACACGACCATCATGTAAATAATCACTACTTCAACGCTCTCT

LVTTV*KTNKSY*PVFLHR**CYSEEW

DraI

7681 CTTGTCACTACAGTTTAAAAGACCAATAAATCCTACTGACCAGTCTTCTTACATCGTTGATAGTGTTACAGTGAAGAATG 7760

GAACAGTGATGTCAAATTTTCTGGTTATTTAGGATGACTGGTCAGAAGAATGTAGCAACTATCACAATGTCACTTCTTAC

FHPSLL**SWSKDL*KTFSLSFC*LRQ

HpaI

7761 GTTCCATCCATCTTTACTTTGATAAAGCTGGTCAAAAGACTTATGAAAGACATTCTCTCTCTCATTTTGTTAACTTAGAC 7840

CAAGGTAGGTAGAAATGAAACTATTTCGACCAGTTTTCTGAATACTTTCTGTAAGAGAGAGAGTAAAACAATTGAATCTG

PES**H*RFIAY*CYSF*W*IKM*RI

AseI

7841 AACCTGAGAGCTAATAACACTAAAGGTTCATTGCCTATTAATGTTATAGTTTTTGATGGTAAATCAAAATGTGAAGAATC 7920

TTGGACTCTCGATTATTGTGATTTCCAAGTAACGGATAATTACAATATCAAAAACTACCATTTAGTTTTACACTTCTTAG

ICKISVCLLQSAYVSTYTVTRSGISV*

7921 ATCTGCAAAATCAGCGTCTGTTTACTACAGTCAGCTTATGTGTCAACCTATACTGTTACTAGATCAGGCATTAGTGTCTG 8000

TAGACGTTTTAGTCGCAGACAAATGATGTCAGTCGAATACACAGTTGGATATGACAATGATCTAGTCCGTAATCACAGAC

CW**CGSCS*NV*CLR*YVFINF*RTN

8001 ATGTTGGTGATAGTGCGGAAGTTGCAGTTAAAATGTTTGATGCTTACGTTAATACGTTTTCATCAACTTTTAACGTACCA 8080

TACAACCACTATCACGCCTTCAACGTCAATTTTACAAACTACGAATGCAATTATGCAAAAGTAGTTGAAAATTGCATGGT

GKTQNTSCNCRS*TCKECVLRQCLIY

SpeIPstI

8081 ATGGAAAAACTCAAAACACTAGTTGCAACTGCAGAAGCTGAACTTGCAAAGAATGTGTCCTTAGACAATGTCTTATCTAC 8160

TACCTTTTTGAGTTTTGTGATCAACGTTGACGTCTTCGACTTGAACGTTTCTTACACAGGAATCTGTTACAGAATAGATG

FYFSSSARVC*FRCRN*RCC*MS*IVT

8161 TTTTATTTCAGCAGCTCGGCAAGGGTTTGTTGATTCAGATGTAGAAACTAAAGATGTTGTTGAATGTCTTAAATTGTCAC 8240

AAAATAAAGTCGTCGAGCCGTTCCCAAACAACTAAGTCTACATCTTTGATTTCTACAACAACTTACAGAATTTAACAGTG

SI*HRSYWR*L**LYAHL*QS*KHDTP

8241 ATCAATCTGACATAGAAGTTACTGGCGATAGTTGTAATAACTATATGCTCACCTATAACAAAGTTGAAAACATGACACCC 8320

TAGTTAGACTGTATCTTCAATGACCGCTATCAACATTATTGATATACGAGTGGATATTGTTTCAACTTTTGTACTGTGGG

*PWCLY*L*CASY*CAGSKKSQHCFD

AseIFspI

8321 CGTGACCTTGGTGCTTGTATTGACTGTAGTGCGCGTCATATTAATGCGCAGGTAGCAAAAAGTCACAACATTGCTTTGAT 8400

GCACTGGAACCACGAACATAACTGACATCACGCGCAGTATAATTACGCGTCCATCGTTTTTCAGTGTTGTAACGAAACTA

MER*RFHVIV*TTTKTNT*CC*KE*LT

AclISnaBI

8401 ATGGAACGTTAAAGATTTCATGTCATTGTCTGAACAACTACGAAAACAAATACGTAGTGCTGCTAAAAAGAATAACTTAC 8480

TACCTTGCAATTTCTAAAGTACAGTAACAGACTTGTTGATGCTTTTGTTTATGCATCACGACGATTTTTCTTATTGAATG

F*VDMCNY*TSC*CCNNKDST*GW*NC

AflII

8481 CTTTTAAGTTGACATGTGCAACTACTAGACAAGTTGTTAATGTTGTAACAACAAAGATAGCACTTAAGGGTGGTAAAATT 8560

GAAAATTCAACTGTACACGTTGATGATCTGTTCAACAATTACAACATTGTTGTTTCTATCGTGAATTCCCACCATTTTAA

**LVEAVN*SYTCVPFCCCYFLFNNT

PacI

8561 GTTAATAATTGGTTGAAGCAGTTAATTAAAGTTACACTTGTGTTCCTTTTTGTTGCTGCTATTTTCTATTTAATAACACC 8640

CAATTATTAACCAACTTCGTCAATTAATTTCAATGTGAACACAAGGAAAAACAACGACGATAAAAGATAAATTATTGTGG

CSCHV*TY*LFK*NHRIQGY*WWCHS*

8641 TGTTCATGTCATGTCTAAACATACTGACTTTTCAAGTGAAATCATAGGATACAAGGCTATTGATGGTGGTGTCACTCGTG 8720

ACAAGTACAGTACAGATTTGTATGACTGAAAAGTTCACTTTAGTATCCTATGTTCCGATAACTACCACCACAGTGAGCAC

HSIYRYLFC*QTC*F*HMV*PAWW*LY

8721 ACATAGCATCTACAGATACTTGTTTTGCTAACAAACATGCTGATTTTGACACATGGTTTAGCCAGCGTGGTGGTAGTTAT 8800

TGTATCGTAGATGTCTATGAACAAAACGATTGTTTGTACGACTAAAACTGTGTACCAAATCGGTCGCACCACCATCAATA

**QSLPIDCCSHNKRSGFCRAWFAWH

HindIIIPstI

8801 ACTAATGACAAAGCTTGCCCATTGATTGCTGCAGTCATAACAAGAGAAGTGGGTTTTGTCGTGCCTGGTTTGCCTGGCAC 8880

TGATTACTGTTTCGAACGGGTAACTAACGACGTCAGTATTGTTCTCTTCACCCAAAACAGCACGGACCAAACGGACCGTG

DITHN*W*LFAFLT*SF*CSW*HLLHT

8881 GATATTACGCACAACTAATGGTGACTTTTTGCATTTCTTACCTAGAGTTTTTAGTGCAGTTGGTAACATCTGTTACACAC 8960

CTATAATGCGTGTTGATTACCACTGAAAAACGTAAAGAATGGATCTCAAAAATCACGTCAACCATTGTAGACAATGTGTG

IKTYRVH*LCNISLCFGC*MYNF*RCF

BsrGIDraI

8961 CATCAAAACTTATAGAGTACACTGACTTTGCAACATCAGCTTGTGTTTTGGCTGCTGAATGTACAATTTTTAAAGATGCT 9040

GTAGTTTTGAATATCTCATGTGACTGAAACGTTGTAGTCGAACACAAAACCGACGACTTACATGTTAAAAATTTCTACGA

W*ASTILL*YQCTRRFCCL*KFTP*H

9041 TCTGGTAAGCCAGTACCATATTGTTATGATACCAATGTACTAGAAGGTTCTGTTGCTTATGAAAGTTTACGCCCTGACAC 9120

AGACCATTCGGTCATGGTATAACAATACTATGGTTACATGATCTTCCAAGACAACGAATACTTTCAAATGCGGGACTGTG

TLCAHGWLYYSIS*HLP*RFC*SGNNF

9121 ACGTTATGTGCTCATGGATGGCTCTATTATTCAATTTCCTAACACCTACCTTGAAGGTTCTGTTAGAGTGGTAACAACTT 9200

TGCAATACACGAGTACCTACCGAGATAATAAGTTAAAGGATTGTGGATGGAACTTCCAAGACAATCTCACCATTGTTGAA

*F*VL*ARHL*KIRSWCLCIY*W*MGT

ScaISpeI

9201 TTGATTCTGAGTACTGTAGGCACGGCACTTGTGAAAGATCAGAAGCTGGTGTTTGTGTATCTACTAGTGGTAGATGGGTA 9280

AACTAAGACTCATGACATCCGTGCCGTGAACACTTTCTAGTCTTCGACCACAAACACATAGATGATCACCATCTACCCAT

*Q*LLQIFTRSFLWCRCCKFTY*YVY

BglII

9281 CTTAACAATGATTATTACAGATCTTTACCAGGAGTTTTCTGTGGTGTAGATGCTGTAAATTTACTTACTAATATGTTTAC 9360

GAATTGTTACTAATAATGTCTAGAAATGGTCCTCAAAAGACACCACATCTACGACATTTAAATGAATGATTATACAAATG

TTNSTYWCFGHISIYSSWWYCSYRSNM

9361 ACCACTAATTCAACCTATTGGTGCTTTGGACATATCAGCATCTATAGTAGCTGGTGGTATTGTAGCTATCGTAGTAACAT 9440

TGGTGATTAAGTTGGATAACCACGAAACCTGTATAGTCGTAGATATCATCGACCACCATAACATCGATAGCATCATTGTA

PCLLFYEV*KSFW*IQSCSCL*YFTIP

9441 GCCTTGCCTACTATTTTATGAGGTTTAGAAGAGCTTTTGGTGAATACAGTCATGTAGTTGCCTTTAATACTTTACTATTC 9520

CGGAACGGATGATAAAATACTCCAAATCTTCTCGAAAACCACTTATGTCAGTACATCAACGGAAATTATGAAATGATAAG

YVIHCTLFNTSLLILTWCLFCYLLVL

9521 CTTATGTCATTCACTGTACTCTGTTTAACACCAGTTTACTCATTCTTACCTGGTGTTTATTCTGTTATTTACTTGTACTT 9600

GAATACAGTAAGTGACATGAGACAAATTGTGGTCAAATGAGTAAGAATGGACCACAAATAAGACAATAAATGAACATGAA

DILSY**CFFFSTYSVDGYVHTFSTFL

9601 GACATTTTATCTTACTAATGATGTTTCTTTTTTAGCACATATTCAGTGGATGGTTATGTTCACACCTTTAGTACCTTTCT 9680

CTGTAAAATAGAATGATTACTACAAAGAAAAAATCGTGTATAAGTCACCTACCAATACAAGTGTGGAAATCATGGAAAGA

DNNCLYHLYFHKAFLLVL**LPKETCS

MfeI

9681 GGATAACAATTGCTTATATCATTTGTATTTCCACAAAGCATTTCTATTGGTTCTTTAGTAATTACCTAAAGAGACGTGTA 9760

CCTATTGTTAACGAATATAGTAAACATAAAGGTGTTTCGTAAAGATAACCAAGAAATCATTAATGGATTTCTCTGCACAT

L*WCFL*YF*RSCAVHLFVK*RNVSK

ScaIApaLI

9761 GTCTTTAATGGTGTTTCCTTTAGTACTTTTGAAGAAGCTGCGCTGTGCACCTTTTTGTTAAATAAAGAAATGTATCTAAA 9840

CAGAAATTACCACAAAGGAAATCATGAAAACTTCTTCGACGCGACACGTGGAAAAACAATTTATTTCTTTACATAGATTT

VA**CAITSYAI**ILSSL**VQVF*W

PsiI

9841 GTTGCGTAGTGATGTGCTATTACCTCTTACGCAATATAATAGATACTTAGCTCTTTATAATAAGTACAAGTATTTTAGTG 9920

CAACGCATCACTACACGATAATGGAGAATGCGTTATATTATCTATGAATCGAGAAATATTATTCATGTTCATAAAATCAC

SNGYN*LQRSCLLSSRKGSQ*LQ*LRF

9921 GAGCAATGGATACAACTAGCTACAGAGAAGCTGCTTGTTGTCATCTCGCAAAGGCTCTCAATGACTTCAGTAACTCAGGT 10000

CTCGTTACCTATGTTGATCGATGTCTCTTCGACGAACAACAGTAGAGCGTTTCCGAGAGTTACTGAAGTCATTGAGTCCA

*CSLPTTTNLYHLSCFAEWF*KNGIP

PvuII

10001 TCTGATGTTCTTTACCAACCACCACAAACCTCTATCACCTCAGCTGTTTTGCAGAGTGGTTTTAGAAAAATGGCATTCCC 10080

AGACTACAAGAAATGGTTGGTGGTGTTTGGAGATAGTGGAGTCGACAAAACGTCTCACCAAAATCTTTTTACCGTAAGGG

IW*S*GLYGTSNLWYNYT*RSLA**RS

10081 ATCTGGTAAAGTTGAGGGTTGTATGGTACAAGTAACTTGTGGTACAACTACACTTAACGGTCTTTGGCTTGATGACGTAG 10160

TAGACCATTTCAACTCCCAACATACCATGTTCATTGAACACCATGTTGATGTGAATTGCCAGAAACCGAACTACTGCATC

LLSKTCDLHL*RHA*P*L*RFTHS*V*

10161 TTTACTGTCCAAGACATGTGATCTGCACCTCTGAAGACATGCTTAACCCTAATTATGAAGATTTACTCATTCGTAAGTCT 10240

AAATGACAGGTTCTGTACACTAGACGTGGAGACTTCTGTACGAATTGGGATTAATACTTCTAAATGAGTAAGCATTCAGA

S*FLGTGW*CSTQGYWTFYAKLCT*A

AflIIHindIII/AflII

10241 AATCATAATTTCTTGGTACAGGCTGGTAATGTTCAACTCAGGGTTATTGGACATTCTATGCAAAATTGTGTACTTAAGCT 10320

TTAGTATTAAAGAACCATGTCCGACCATTACAAGTTGAGTCCCAATAACCTGTAAGATACGTTTTAACACATGAATTCGA

*G*YSQS*DT*V*VCSHSTRTDFFSVS

10321 TAAGGTTGATACAGCCAATCCTAAGACACCTAAGTATAAGTTTGTTCGCATTCAACCAGGACAGACTTTTTCAGTGTTAG 10400

ATTCCAACTATGTCGGTTAGGATTCTGTGGATTCATATTCAAACAAGCGTAAGTTGGTCCTGTCTGAAAAAGTCACAATC

LLQWFTIWCLPMCYEAQFHY*GFIP*W

10401 CTTGTTACAATGGTTCACCATCTGGTGTTTACCAATGTGCTATGAGGCCCAATTTCACTATTAAGGGTTCATTCCTTAAT 10480

GAACAATGTTACCAAGTGGTAGACCACAAATGGTTACACGATACTCCGGGTTAAAGTGATAATTCCCAAGTAAGGAATTA

FMW*CWF*HRL*LCLFLLHAPYGITN

NdeI

10481 GGTTCATGTGGTAGTGTTGGTTTTAACATAGATTATGACTGTGTCTCTTTTTGTTACATGCACCATATGGAATTACCAAC 10560

CCAAGTACACCATCACAACCAAAATTGTATCTAATACTGACACAGAGAAAAACAATGTACGTGGTATACCTTAATGGTTG

WSSCWHRLRR*LLWTFC*QANSTSSWY

PvuII

10561 TGGAGTTCATGCTGGCACAGACTTAGAAGGTAACTTTTATGGACCTTTTGTTGACAGGCAAACAGCACAAGCAGCTGGTA 10640

ACCTCAAGTACGACCGTGTCTGAATCTTCCATTGAAAATACCTGGAAAACAACTGTCCGTTTGTCGTGTTCGTCGACCAT

GHNYYS*CFSLVVRCCYKWRQVVSQSI

PsiIClaI

10641 CGGACACAACTATTACAGTTAATGTTTTAGCTTGGTTGTACGCTGCTGTTATAAATGGAGACAGGTGGTTTCTCAATCGA 10720

GCCTGTGTTGATAATGTCAATTACAAAATCGAACCAACATGCGACGACAATATTTACCTCTGTCCACCAAAGAGTTAGCT

YHNS**L*PCGYEVQL*TSNTRPC*H

10721 TTTACCACAACTCTTAATGACTTTAACCTTGTGGCTATGAAGTACAATTATGAACCTCTAACACAAGACCATGTTGACAT 10800

AAATGGTGTTGAGAATTACTGAAATTGGAACACCGATACTTCATGTTAATACTTGGAGATTGTGTTCTGGTACAACTGTA

TRTSFCSNWNCRFRYVCFIKRITAKWY

10801 ACTAGGACCTCTTTCTGCTCAAACTGGAATTGCCGTTTTAGATATGTGTGCTTCATTAAAAGAATTACTGCAAAATGGTA 10880

TGATCCTGGAGAAAGACGAGTTTGACCTTAACGGCAAAATCTATACACACGAAGTAATTTTCTTAATGACGTTTTACCAT

EWTYHIG*CFIRR*IYTF*CC*TMLRC

10881 TGAATGGACGTACCATATTGGGTAGTGCTTTATTAGAAGATGAATTTACACCTTTTGATGTTGTTAGACAATGCTCAGGT 10960

ACTTACCTGCATGGTATAACCCATCACGAAATAATCTTCTACTTAAATGTGGAAAACTACAACAATCTGTTACGAGTCCA

YFPKCSEKNNQGYTPLVVTHNFDFTF

10961 GTTACTTTCCAAAGTGCAGTGAAAAGAACAATCAAGGGTACACACCACTGGTTGTTACTCACAATTTTGACTTCACTTTT 11040

CAATGAAAGGTTTCACGTCACTTTTCTTGTTAGTTCCCATGTGTGGTGACCAACAATGAGTGTTAAAACTGAAGTGAAAA

SFSPEYSMVFVLFFV*KCLFTFCYGYY

ScaI

11041 AGTTTTAGTCCAGAGTACTCAATGGTCTTTGTTCTTTTTTTTGTATGAAAATGCCTTTTTACCTTTTGCTATGGGTATTA 11120

TCAAAATCAGGTCTCATGAGTTACCAGAAACAAGAAAAAAAACATACTTTTACGGAAAAATGGAAAACGATACCCATAAT

CYVCFCNDVCQT*ACISLFVFVTFSCH

SphI/NsiI

11121 TTGCTATGTCTGCTTTTGCAATGATGTTTGTCAAACATAAGCATGCATTTCTCTGTTTGTTTTTGTTACCTTCTCTTGCC 11200

AACGATACAGACGAAAACGTTACTACAAACAGTTTGTATTCGTACGTAAAGAGACAAACAAAAACAATGGAAGAGAACGG

CSLF*YGLYAC*LGDAYYDMVGYG*Y

SpeI

11201 ACTGTAGCTTATTTTAATATGGTCTATATGCCTGCTAGTTGGGTGATGCGTATTATGACATGGTTGGATATGGTTGATAC 11280

TGACATCGAATAAAATTATACCAGATATACGGACGATCAACCCACTACGCATAATACTGTACCAACCTATACCAACTATG

*FVWF*AKRLCYVCISCSVTNPYDSKN

NsiIPvuII

11281 TAGTTTGTCTGGTTTTAAGCTAAAAGACTGTGTTATGTATGCATCAGCTGTAGTGTTACTAATCCTTATGACAGCAAGAA 11360

ATCAAACAGACCAAAATTCGATTTTCTGACACAATACATACGTAGTCGACATCACAATGATTAGGAATACTGTCGTTCTT

CV**WC*ESVDTYECLDTRL*SLLW*C

PsiI

11361 CTGTGTATGATGATGGTGCTAGGAGAGTGTGGACACTTATGAATGTCTTGACACTCGTTTATAAAGTTTATTATGGTAAT 11440

GACACATACTACTACCACGATCCTCTCACACCTGTGAATACTTACAGAACTGTGAGCAAATATTTCAAATAATACCATTA

FRSSHFHVGSYNLCYF*LLRCSYNCH

PsiI

11441 GCTTTAGATCAAGCCATTTCCATGTGGGCTCTTATAATCTCTGTTACTTCTAACTACTCAGGTGTAGTTACAACTGTCAT 11520

CGAAATCTAGTTCGGTAAAGGTACACCCGAGAATATTAGAGACAATGAAGATTGATGAGTCCACATCAATGTTGACAGTA

VFGQRYCFYVC*VLPYFLHNW*YTSVY

MscI

11521 GTTTTTGGCCAGAGGTATTGTTTTTATGTGTGTTGAGTATTGCCCTATTTTCTTCATAACTGGTAATACACTTCAGTGTA 11600

CAAAAACCGGTCTCCATAACAAAAATACACACAACTCATAACGGGATAAAAGAAGTATTGACCATTATGTGAAGTCACAT

NASLLFLRLFLYLLLWPLLFTQPLL*T

11601 TAATGCTAGTTTATTGTTTCTTAGGCTATTTTTGTACTTGTTACTTTGGCCTCTTTTGTTTACTCAACCGCTACTTTAGA 11680

ATTACGATCAAATAACAAAGAATCCGATAAAAACATGAACAATGAAACCGGAGAAAACAAATGAGTTGGCGATGAAATCT

DSWCL*LLSFYTGV*IYEFTGTTPTQ

EcoRI

11681 CTGACTCTTGGTGTTTATGATTACTTAGTTTCTACACAGGAGTTTAGATATATGAATTCACAGGGACTACTCCCACCCAA 11760

GACTGAGAACCACAAATACTAATGAATCAAAGATGTGTCCTCAAATCTATATACTTAAGTGTCCCTGATGAGGGTGGGTT

E*HRCLQTQH*IVGCWWQTLYQSSHCT

BsrGI

11761 GAATAGCATAGATGCCTTCAAACTCAACATTAAATTGTTGGGTGTTGGTGGCAAACCTTGTATCAAAGTAGCCACTGTAC 11840

CTTATCGTATCTACGGAAGTTTGAGTTGTAATTTAACAACCCACAACCACCGTTTGGAACATAGTTTCATCGGTGACATG

V*NVRCKVHISSLTLSFATTQSRIII*

ApaLI

11841 AGTCTAAAATGTCAGATGTAAAGTGCACATCAGTAGTCTTACTCTCAGTTTTGCAACAACTCAGAGTAGAATCATCATCT 11920

TCAGATTTTACAGTCTACATTTCACGTGTAGTCATCAGAATGAGAGTCAAAACGTTGTTGAGTCTCATCTTAGTAGTAGA

IVGSMCPVTQ*HSLS*RYY*SL*KNG

11921 AAATTGTGGGCTCAATGTGTCCAGTTACACAATGACATTCTCTTAGCTAAAGATACTACTGAAGCCTTTGAAAAAATGGT 12000

TTTAACACCCGAGTTACACAGGTCAATGTGTTACTGTAAGAGAATCGATTTCTATGATGACTTCGGAAACTTTTTTACCA

FTTFCFAFHAGCCRHKQAL*RNAGQQG

HindIII

12001 TTCACTACTTTCTGTTTTGCTTTCCATGCAGGGTGCTGTAGACATAAACAAGCTTTGTGAAGAAATGCTGGACAACAGGG 12080

AAGTGATGAAAGACAAAACGAAAGGTACGTCCCACGACATCTGTATTTGTTCGAAACACTTCTTTACGACCTGTTGTCCC

NLTSYSLRV*FPSIICSFCYCSRSL*A

NdeIHindIII

12081 CAACCTTACAAGCTATAGCCTCAGAGTTTAGTTCCCTTCCATCATATGCAGCTTTTGCTACTGCTCAAGAAGCTTATGAG 12160

GTTGGAATGTTCGATATCGGAGTCTCAAATCAAGGGAAGGTAGTATACGTCGAAAACGATGACGAGTTCTTCGAATACTC

GCC*W*F*SCS*KVEEVFECG*I*I*

12161 CAGGCTGTTGCTAATGGTGATTCTGAAGTTGTTCTTAAAAAGTTGAAGAAGTCTTTGAATGTGGCTAAATCTGAATTTGA 12240

GTCCGACAACGATTACCACTAAGACTTCAACAAGAATTTTTCAACTTCTTCAGAAACTTACACCGATTTAGACTTAAACT

P*CSHAT*VGKDG*SSYDPNV*TG*I*

BglII

12241 CCGTGATGCAGCCATGCAACGTAAGTTGGAAAAGATGGCTGATCAAGCTATGACCCAAATGTATAAACAGGCTAGATCTG 12320

GGCACTACGTCGGTACGTTGCATTCAACCTTTTCTACCGACTAGTTCGATACTGGGTTTACATATTTGTCCGATCTAGAC

GQEGKSY*CYADNAFHYA*KVG**CTQ

SpeI

12321 AGGACAAGAGGGCAAAAGTTACTAGTGCTATGCAGACAATGCTTTTCACTATGCTTAGAAAGTTGGATAATGATGCACTC 12400

TCCTGTTCTCCCGTTTTCAATGATCACGATACGTCTGTTACGAAAAGTGATACGAATCTTTCAACCTATTACTACGTGAG

QHYQQCKRWLCSLEHNTSYNSSQTNG

12401 AACAACATTATCAACAATGCAAGAGATGGTTGTGTTCCCTTGAACATAATACCTCTTACAACAGCAGCCAAACTAATGGT 12480

TTGTTGTAATAGTTGTTACGTTCTCTACCAACACAAGGGAACTTGTATTATGGAGAATGTTGTCGTCGGTTTGATTACCA

CHTRL*HI*KYV*WYNIYLCISIVGNP

NsiI

12481 TGTCATACCAGACTATAACACATATAAAAATACGTGTGATGGTACAACATTTACTTATGCATCAGCATTGTGGGAAATCC 12560

ACAGTATGGTCTGATATTGTGTATATTTTTATGCACACTACCATGTTGTAAATGAATACGTAGTCGTAACACCCTTTAGG

TGCRCR**NCST**N*YGQFT*FSMAS

12561 AACAGGTTGTAGATGCAGATAGTAAAATTGTTCAACTTAGTGAAATTAGTATGGACAATTCACCTAATTTAGCATGGCCT 12640

TTGTCCAACATCTACGTCTATCATTTTAACAAGTTGAATCACTTTAATCATACCTGTTAAGTGGATTAAATCGTACCGGA

YCNSFKGQFCCQITE**A*SCCTTTD

12641 CTTATTGTAACAGCTTTAAGGGCCAATTCTGCTGTCAAATTACAGAATAATGAGCTTAGTCCTGTTGCACTACGACAGAT 12720

GAATAACATTGTCGAAATTCCCGGTTAAGACGACAGTTTAATGTCTTATTACTCGAATCAGGACAACGTGATGCTGTCTA

VLCCRYYTNCLH**QCVSLLQHNKGR*

12721 GTCTTGTGCTGCCGGTACTACACAAACTGCTTGCACTGATGACAATGCGTTAGCTTACTACAACACAACAAAGGGAGGTA 12800

CAGAACACGACGGCCATGATGTGTTTGACGAACGTGACTACTGTTACGCAATCGAATGATGTTGTGTTGTTTCCCTCCAT

VCTCTVIRFTGFEMG*IP*E*WNWYYL

12801 GGTTTGTACTTGCACTGTTATCCGATTTACAGGATTTGAAATGGGCTAGATTCCCTAAGAGTGATGGAACTGGTACTATC 12880

CCAAACATGAACGTGACAATAGGCTAAATGTCCTAAACTTTACCCGATCTAAGGGATTCTCACTACCTTGACCATGATAG

YRTGTTL*VCYRHT*RS*SEVFILY*

12881 TATACAGAACTGGAACCACCTTGTAGGTTTGTTACAGACACACCTAAAGGTCCTAAAGTGAAGTATTTATACTTTATTAA 12960

ATATGTCTTGACCTTGGTGGAACATCCAAACAATGTCTGTGTGGATTTCCAGGATTTCACTTCATAAATATGAAATAATT

RIKQPK*RYGTW*FSCHSTSTSW*CNR

12961 AGGATTAAACAACCTAAATAGAGGTATGGTACTTGGTAGTTTAGCTGCCACAGTACGTCTACAAGCTGGTAATGCAACAG 13040

TCCTAATTTGTTGGATTTATCTCCATACCATGAACCATCAAATCGACGGTGTCATGCAGATGTTCGACCATTACGTTGTC

SACQFNCIIFLCFCCRCC*SLQRLSS*

HindIII

13041 AAGTGCCTGCCAATTCAACTGTATTATCTTTCTGTGCTTTTGCTGTAGATGCTGCTAAAGCTTACAAAGATTATCTAGCT 13120

TTCACGGACGGTTAAGTTGACATAATAGAAAGACACGAAAACGACATCTACGACGATTTCGAATGTTTCTAATAGATCGA

WGTTNH*LC*DVVYTHWYWSGNNSYT

BsrGI

13121 AGTGGGGGACAACCAATCACTAATTGTGTTAAGATGTTGTGTACACACACTGGTACTGGTCAGGCAATAACAGTTACACC 13200

TCACCCCCTGTTGGTTAGTGATTAACACAATTCTACAACACATGTGTGTGACCATGACCAGTCCGTTATTGTCAATGTGG

GSQYGSRILWWCIVLSVLPLPHRSSKS

13201 GGAAGCCAATATGGATCAAGAATCCTTTGGTGGTGCATCGTGTTGTCTGTACTGCCGTTGCCACATAGATCATCCAAATC 13280

CCTTCGGTTATACCTAGTTCTTAGGAAACCACCACGTAGCACAACAGACATGACGGCAACGGTGTATCTAGTAGGTTTAG

*RIL*LKR*VCTNTYNLC**PCGFYT*

BsrGI

13281 CTAAAGGATTTTGTGACTTAAAAGGTAAGTATGTACAAATACCTACAACTTGTGCTAATGACCCTGTGGGTTTTACACTT 13360

GATTTCCTAAAACACTGAATTTTCCATTCATACATGTTTATGGATGTTGAACACGATTACTGGGACACCCAAAATGTGAA

KHSLYRLRYVERLWL*L*STPRTHAS

13361 AAAAACACAGTCTGTACCGTCTGCGGTATGTGGAAAGGTTATGGCTGTAGTTGTGATCAACTCCGCGAACCCATGCTTCA 13440

TTTTTGTGTCAGACATGGCAGACGCCATACACCTTTCCAATACCGACATCAACACTAGTTGAGGCGCTTGGGTACGAAGT

VS*CTIVFKRVCGVSAARLTPCGTGTS

PvuIIDraISpeIScaI

13441 GTCAGCTGATGCACAATCGTTTTTAAACGGGTTTGCGGTGTAAGTGCAGCCCGTCTTACACCGTGCGGCACAGGCACTAG 13520

CAGTCGACTACGTGTTAGCAAAAATTTGCCCAAACGCCACATTCACGTCGGGCAGAATGTGGCACGCCGTGTCCGTGATC

TDVVYRAFDIYNDKVAGFAKFLKTNCC

13521 TACTGATGTCGTATACAGGGCTTTTGACATCTACAATGATAAAGTAGCTGGTTTTGCTAAATTCCTAAAAACTAATTGTT 13600

ATGACTACAGCATATGTCCCGAAAACTGTAGATGTTACTATTTCATCGACCAAAACGATTTAAGGATTTTTGATTAACAA

RFQEKDEDDNLIDSYFVVKRHTFSNY

13601 GTCGCTTCCAAGAAAAGGACGAAGATGACAATTTAATTGATTCTTACTTTGTAGTTAAGAGACACACTTTCTCTAACTAC 13680

CAGCGAAGGTTCTTTTCCTGCTTCTACTGTTAAATTAACTAAGAATGAAACATCAATTCTCTGTGTGAAAGAGATTGATG

QHEETIYNLLKDCPAVAKHDFFKFRID

PsiIAflIIPvuII

13681 CAACATGAAGAAACAATTTATAATTTACTTAAGGATTGTCCAGCTGTTGCTAAACATGACTTCTTTAAGTTTAGAATAGA 13760

GTTGTACTTCTTTGTTAAATATTAAATGAATTCCTAACAGGTCGACAACGATTTGTACTGAAGAAATTCAAATCTTATCT

GDMVPHISRQRLTKYTMADLVYALRHF

KpnI

13761 CGGTGACATGGTACCACATATATCACGTCAACGTCTTACTAAATACACAATGGCAGACCTCGTCTATGCTTTAAGGCATT 13840

GCCACTGTACCATGGTGTATATAGTGCAGTTGCAGAATGATTTATGTGTTACCGTCTGGAGCAGATACGAAATTCCGTAA

DEGNCDTLKEILVTYNCCDDDYFNKK

MfeI

13841 TTGATGAAGGTAATTGTGACACATTAAAAGAAATACTTGTCACATACAATTGTTGTGATGATGATTATTTCAATAAAAAG 13920

AACTACTTCCATTAACACTGTGTAATTTTCTTTATGAACAGTGTATGTTAACAACACTACTACTAATAAAGTTATTTTTC

DWYDFVENPDILRVYANLGERVRQALL

MluIHindIII

13921 GACTGGTATGATTTTGTAGAAAACCCAGATATATTACGCGTATACGCCAACTTAGGTGAACGTGTACGCCAAGCTTTGTT 14000

CTGACCATACTAAAACATCTTTTGGGTCTATATAATGCGCATATGCGGTTGAATCCACTTGCACATGCGGTTCGAAACAA

KTVQFCDAMRNAGIVGVLTLDNQDLNG

BglII

14001 AAAAACAGTACAATTCTGTGATGCCATGCGAAATGCTGGTATTGTTGGTGTACTGACATTAGATAATCAAGATCTCAATG 14080

TTTTTGTCATGTTAAGACACTACGGTACGCTTTACGACCATAACAACCACATGACTGTAATCTATTAGTTCTAGAGTTAC

NWYDFGDFIQTTPGSGVPVVDSYYSL

14081 GTAACTGGTATGATTTCGGTGATTTCATACAAACCACGCCAGGTAGTGGAGTTCCTGTTGTAGATTCTTATTATTCATTG 14160

CATTGACCATACTAAAGCCACTAAAGTATGTTTGGTGCGGTCCATCACCTCAAGGACAACATCTAAGAATAATAAGTAAC

LMPILTLTRALTAESHVDTDLTKPYIK

PstI

14161 TTAATGCCTATATTAACCTTGACCAGGGCTTTAACTGCAGAGTCACATGTTGACACTGACTTAACAAAGCCTTACATTAA 14240

AATTACGGATATAATTGGAACTGGTCCCGAAATTGACGTCTCAGTGTACAACTGTGACTGAATTGTTTCGGAATGTAATT

WDLLKYDFTEERLKLFDRYFKYWDQTY

DraISspI

14241 GTGGGATTTGTTAAAATATGACTTCACGGAAGAGAGGTTAAAACTCTTTGACCGTTATTTTAAATATTGGGATCAGACAT 14320

CACCCTAAACAATTTTATACTGAAGTGCCTTCTCTCCAATTTTGAGAAACTGGCAATAAAATTTATAACCCTAGTCTGTA

HPNCVNCLDDRCILHCANFNVLFSTV

HpaINsiI

14321 ACCACCCAAATTGTGTTAACTGTTTGGATGACAGATGCATTCTGCATTGTGCAAACTTTAATGTTTTATTCTCTACAGTG 14400

TGGTGGGTTTAACACAATTGACAAACCTACTGTCTACGTAAGACGTAACACGTTTGAAATTACAAAATAAGAGATGTCAC

FPPTSFGPLVRKIFVDGVPFVVSTGYH

SpeISspI

14401 TTCCCACCTACAAGTTTTGGACCACTAGTGAGAAAAATATTTGTTGATGGTGTTCCATTTGTAGTTTCAACTGGATACCA 14480

AAGGGTGGATGTTCAAAACCTGGTGATCACTCTTTTTATAAACAACTACCACAAGGTAAACATCAAAGTTGACCTATGGT

FRELGVVHNQDVNLHSSRLSFKELLVY

BsrGIXbaI

14481 CTTCAGAGAGCTAGGTGTTGTACATAATCAGGATGTAAACTTACATAGCTCTAGACTTAGTTTTAAGGAATTACTTGTGT 14560

GAAGTCTCTCGATCCACAACATGTATTAGTCCTACATTTGAATGTATCGAGATCTGAATCAAAATTCCTTAATGAACACA

AADPAMHAASGNLLLDKRTTCFSVAA

14561 ATGCTGCTGACCCTGCTATGCACGCTGCTTCTGGTAATCTATTACTAGATAAACGCACTACGTGCTTTTCAGTAGCTGCA 14640

TACGACGACTGGGACGATACGTGCGACGAAGACCATTAGATAATGATCTATTTGCGTGATGCACGAAAAGTCATCGACGT

LTNNVAFQTVKPGNFNKDFYDFAVSKG

14641 CTTACTAACAATGTTGCTTTTCAAACTGTCAAACCCGGTAATTTTAACAAAGACTTCTATGACTTTGCTGTGTCTAAGGG 14720

GAATGATTGTTACAACGAAAAGTTTGACAGTTTGGGCCATTAAAATTGTTTCTGAAGATACTGAAACGACACAGATTCCC

FFKEGSSVELKHFFFAQDGNAAISDYD

14721 TTTCTTTAAGGAAGGAAGTTCTGTTGAATTAAAACACTTCTTCTTTGCTCAGGATGGTAATGCTGCTATCAGCGATTATG 14800

AAAGAAATTCCTTCCTTCAAGACAACTTAATTTTGTGAAGAAGAAACGAGTCCTACCATTACGACGATAGTCGCTAATAC

YYRYNLPTMCDIRQLLFVVEVVDKYF

PsiIEcoRVScaI

14801 ACTACTATCGTTATAATCTACCAACAATGTGTGATATCAGACAACTACTATTTGTAGTTGAAGTTGTTGATAAGTACTTT 14880

TGATGATAGCAATATTAGATGGTTGTTACACACTATAGTCTGTTGATGATAAACATCAACTTCAACAACTATTCATGAAA

DCYDGGCINANQVIVNNLDKSAGFPFN

AseIPvuII

14881 GATTGTTACGATGGTGGCTGTATTAATGCTAACCAAGTCATCGTCAACAACCTAGACAAATCAGCTGGTTTTCCATTTAA 14960

CTAACAATGCTACCACCGACATAATTACGATTGGTTCAGTAGCAGTTGTTGGATCTGTTTAGTCGACCAAAAGGTAAATT

KWGKARLYYDSMSYEDQDALFAYTKRN

14961 TAAATGGGGTAAGGCTAGACTTTATTATGATTCAATGAGTTATGAGGATCAAGATGCACTTTTCGCATATACAAAACGTA 15040

ATTTACCCCATTCCGATCTGAAATAATACTAAGTTACTCAATACTCCTAGTTCTACGTGAAAAGCGTATATGTTTTGCAT

VIPTITQMNLKYAISAKNRARTVAGV

AflIISacI

15041 ATGTCATCCCTACTATAACTCAAATGAATCTTAAGTATGCCATTAGTGCAAAGAATAGAGCTCGCACCGTAGCTGGTGTC 15120

TACAGTAGGGATGATATTGAGTTTACTTAGAATTCATACGGTAATCACGTTTCTTATCTCGAGCGTGGCATCGACCACAG

SICSTMTNRQFHQKLLKSIAATRGATV

ScaI

15121 TCTATCTGTAGTACTATGACCAATAGACAGTTTCATCAAAAATTATTGAAATCAATAGCCGCCACTAGAGGAGCTACTGT 15200

AGATAGACATCATGATACTGGTTATCTGTCAAAGTAGTTTTTAATAACTTTAGTTATCGGCGGTGATCTCCTCGATGACA

VIGTSKFYGGWHNMLKTVYSDVENPHL

15201 AGTAATTGGAACAAGCAAATTCTATGGTGGTTGGCACAACATGTTAAAAACTGTTTATAGTGATGTAGAAAACCCTCACC 15280

TCATTAACCTTGTTCGTTTAAGATACCACCAACCGTGTTGTACAATTTTTGACAAATATCACTACATCTTTTGGGAGTGG

MGWDYPKCDRAMPNMLRIMASLVLAR

15281 TTATGGGTTGGGATTATCCTAAATGTGATAGAGCCATGCCTAACATGCTTAGAATTATGGCCTCACTTGTTCTTGCTCGC 15360

AATACCCAACCCTAATAGGATTTACACTATCTCGGTACGGATTGTACGAATCTTAATACCGGAGTGAACAAGAACGAGCG

KHTTCCSLSHRFYRLANECAQVLSEMV

15361 AAACATACAACGTGTTGTAGCTTGTCACACCGTTTCTATAGATTAGCTAATGAGTGTGCTCAAGTATTGAGTGAAATGGT 15440

TTTGTATGTTGCACAACATCGAACAGTGTGGCAAAGATATCTAATCGATTACTCACACGAGTTCATAACTCACTTTACCA

MCGGSLYVKPGGTSSGDATTAYANSVF

15441 CATGTGTGGCGGTTCACTATATGTTAAACCAGGTGGAACCTCATCAGGAGATGCCACAACTGCTTATGCTAATAGTGTTT 15520

GTACACACCGCCAAGTGATATACAATTTGGTCCACCTTGGAGTAGTCCTCTACGGTGTTGACGAATACGATTATCACAAA

NICQAVTANVNALLSTDGNKIADKYV

15521 TTAACATTTGTCAAGCTGTCACGGCCAATGTTAATGCACTTTTATCTACTGATGGTAACAAAATTGCCGATAAGTATGTC 15600

AATTGTAAACAGTTCGACAGTGCCGGTTACAATTACGTGAAAATAGATGACTACCATTGTTTTAACGGCTATTCATACAG

RNLQHRLYECLYRNRDVDTDFVNEFYA

15601 CGCAATTTACAACACAGACTTTATGAGTGTCTCTATAGAAATAGAGATGTTGACACAGACTTTGTGAATGAGTTTTACGC 15680

GCGTTAAATGTTGTGTCTGAAATACTCACAGAGATATCTTTATCTCTACAACTGTGTCTGAAACACTTACTCAAAATGCG

YLRKHFSMMILSDDAVVCFNSTYASQG

NsiI

15681 ATATTTGCGTAAACATTTCTCAATGATGATACTCTCTGACGATGCTGTTGTGTGTTTCAATAGCACTTATGCATCTCAAG 15760

TATAAACGCATTTGTAAAGAGTTACTACTATGAGAGACTGCTACGACAACACACAAAGTTATCGTGAATACGTAGAGTTC

LVASIKNFKSVLYYQNNVFMSEAKCW

NheI

15761 GTCTAGTGGCTAGCATAAAGAACTTTAAGTCAGTTCTTTATTATCAAAACAATGTTTTTATGTCTGAAGCAAAATGTTGG 15840

CAGATCACCGATCGTATTTCTTGAAATTCAGTCAAGAAATAATAGTTTTGTTACAAAAATACAGACTTCGTTTTACAACC

TETDLTKGPHEFCSQHTMLVKQGDDYV

BspHI

15841 ACTGAGACTGACCTTACTAAAGGACCTCATGAATTTTGCTCTCAACATACAATGCTAGTTAAACAGGGTGATGATTATGT 15920

TGACTCTGACTGGAATGATTTCCTGGAGTACTTAAAACGAGAGTTGTATGTTACGATCAATTTGTCCCACTACTAATACA

YLPYPDPSRILGAGCFVDDIVKTDGTL

AvrIINaeIEcoRV

15921 GTACCTTCCTTACCCAGATCCATCAAGAATCCTAGGGGCCGGCTGTTTTGTAGATGATATCGTAAAAACAGATGGTACAC 16000

CATGGAAGGAATGGGTCTAGGTAGTTCTTAGGATCCCCGGCCGACAAAACATCTACTATAGCATTTTTGTCTACCATGTG

MIERFVSLAIDAYPLTKHPNQEYADV

16001 TTATGATTGAACGGTTCGTGTCTTTAGCTATAGATGCTTACCCACTTACTAAACATCCTAATCAGGAGTATGCTGATGTC 16080

AATACTAACTTGCCAAGCACAGAAATCGATATCTACGAATGGGTGAATGATTTGTAGGATTAGTCCTCATACGACTACAG

FHLYLQYIRKLHDELTGHMLDMYSVML

HpaI

16081 TTTCATTTGTACTTACAATACATAAGAAAGCTACATGATGAGTTAACAGGACACATGTTAGACATGTATTCTGTTATGCT 16160

AAAGTAAACATGAATGTTATGTATTCTTTCGATGTACTACTCAATTGTCCTGTGTACAATCTGTACATAAGACAATACGA

TNDNTSRYWEPEFYEAMYTPHTVLQAV

BsrGI

16161 TACTAATGATAACACTTCAAGGTATTGGGAACCTGAGTTTTATGAGGCTATGTACACACCGCATACAGTCTTACAGGCTG 16240

ATGATTACTATTGTGAAGTTCCATAACCCTTGGACTCAAAATACTCCGATACATGTGTGGCGTATGTCAGAATGTCCGAC

GACVLCNSQTSLRCGACIRRPFLCCK

SnaBI

16241 TTGGGGCTTGTGTTCTTTGCAATTCACAGACTTCATTAAGATGTGGTGCTTGCATACGTAGACCATTCTTATGTTGTAAA 16320

AACCCCGAACACAAGAAACGTTAAGTGTCTGAAGTAATTCTACACCACGAACGTATGCATCTGGTAAGAATACAACATTT

CCYDHVISTSHKLVLSVNPYVCNAPGC

16321 TGCTGTTACGACCATGTCATATCAACATCACATAAATTAGTCTTGTCTGTTAATCCGTATGTTTGCAATGCTCCAGGTTG 16400

ACGACAATGCTGGTACAGTATAGTTGTAGTGTATTTAATCAGAACAGACAATTAGGCATACAAACGTTACGAGGTCCAAC

DVTDVTQLYLGGMSYYCKSHKPPISFP

16401 TGATGTCACAGATGTGACTCAACTTTACTTAGGAGGTATGAGCTATTATTGTAAATCACATAAACCACCCATTAGTTTTC 16480

ACTACAGTGTCTACACTGAGTTGAAATGAATCCTCCATACTCGATAATAACATTTAGTGTATTTGGTGGGTAATCAAAAG

LCANGQVFGLYKNTCVGSDNVTDFNA

MfeI

16481 CATTGTGTGCTAATGGACAAGTTTTTGGTTTATATAAAAATACATGTGTTGGTAGCGATAATGTTACTGACTTTAATGCA 16560

GTAACACACGATTACCTGTTCAAAAACCAAATATATTTTTATGTACACAACCATCGCTATTACAATGACTGAAATTACGT

IATCDWTNAGDYILANTCTERLKLFAA

HindIII

16561 ATTGCAACATGTGACTGGACAAATGCTGGTGATTACATTTTAGCTAACACCTGTACTGAAAGACTCAAGCTTTTTGCAGC 16640

TAACGTTGTACACTGACCTGTTTACGACCACTAATGTAAAATCGATTGTGGACATGACTTTCTGAGTTCGAAAAACGTCG

ETLKATEETFKLSYGIATVREVLSDRE

DraI

16641 AGAAACGCTCAAAGCTACTGAGGAGACATTTAAACTGTCTTATGGTATTGCTACTGTACGTGAAGTGCTGTCTGACAGAG 16720

TCTTTGCGAGTTTCGATGACTCCTCTGTAAATTTGACAGAATACCATAACGATGACATGCACTTCACGACAGACTGTCTC

LHLSWEVGKPRPPLNRNYVFTGYRVT

16721 AATTACATCTTTCATGGGAAGTTGGTAAACCTAGACCACCACTTAACCGAAATTATGTCTTTACTGGTTATCGTGTAACT 16800

TTAATGTAGAAAGTACCCTTCAACCATTTGGATCTGGTGGTGAATTGGCTTTAATACAGAAATGACCAATAGCACATTGA

KNSKVQIGEYTFEKGDYGDAVVYRGTT

16801 AAAAACAGTAAAGTACAAATAGGAGAGTACACCTTTGAAAAAGGTGACTATGGTGATGCTGTTGTTTACCGAGGTACAAC 16880

TTTTTGTCATTTCATGTTTATCCTCTCATGTGGAAACTTTTTCCACTGATACCACTACGACAACAAATGGCTCCATGTTG

TYKLNVGDYFVLTSHTVMPLSAPTLVP

ApaLISpeI

16881 AACTTACAAATTAAATGTTGGTGATTATTTTGTGCTGACATCACATACAGTAATGCCATTAAGTGCACCTACACTAGTGC 16960

TTGAATGTTTAATTTACAACCACTAATAAAACACGACTGTAGTGTATGTCATTACGGTAATTCACGTGGATGTGATCACG

QEHYVRITGLYPTLNISDEFSSNVAN

16961 CACAAGAGCACTATGTTAGAATTACTGGCTTATACCCAACACTCAATATCTCAGATGAGTTTTCTAGCAATGTTGCAAAT 17040

GTGTTCTCGTGATACAATCTTAATGACCGAATATGGGTTGTGAGTTATAGAGTCTACTCAAAAGATCGTTACAACGTTTA

YQKVGMQKYSTLQGPPGTGKSHFAIGL

17041 TATCAAAAGGTTGGTATGCAAAAGTATTCTACACTCCAGGGACCACCTGGTACTGGTAAGAGTCATTTTGCTATTGGCCT 17120

ATAGTTTTCCAACCATACGTTTTCATAAGATGTGAGGTCCCTGGTGGACCATGACCATTCTCAGTAAAACGATAACCGGA

ALYYPSARIVYTACSHAAVDALCEKAL

17121 AGCTCTCTACTACCCTTCTGCTCGCATAGTGTATACAGCTTGCTCTCATGCCGCTGTTGATGCACTATGTGAGAAGGCAT 17200

TCGAGAGATGATGGGAAGACGAGCGTATCACATATGTCGAACGAGAGTACGGCGACAACTACGTGATACACTCTTCCGTA

KYLPIDKCSRIIPARARVECFDKFKV

SspIPmlIEcoRI

17201 TAAAATATTTGCCTATAGATAAATGTAGTAGAATTATACCTGCACGTGCTCGTGTAGAGTGTTTTGATAAATTCAAAGTG 17280

ATTTTATAAACGGATATCTATTTACATCATCTTAATATGGACGTGCACGAGCACATCTCACAAAACTATTTAAGTTTCAC

NSTLEQYVFCTVNALPETTADIVVFDE

NsiI

17281 AATTCAACATTAGAACAGTATGTCTTTTGTACTGTAAATGCATTGCCTGAGACGACAGCAGATATAGTTGTCTTTGATGA 17360

TTAAGTTGTAATCTTGTCATACAGAAAACATGACATTTACGTAACGGACTCTGCTGTCGTCTATATCAACAGAAACTACT

ISMATNYDLSVVNARLRAKHYVYIGDP

MscIBsrGI

17361 AATTTCAATGGCCACAAATTATGATTTGAGTGTTGTCAATGCCAGATTACGTGCTAAGCACTATGTGTACATTGGCGACC 17440

TTAAAGTTACCGGTGTTTAATACTAAACTCACAACAGTTACGGTCTAATGCACGATTCGTGATACACATGTAACCGCTGG

AQLPAPRTLLTKGTLEPEYFNSVCRL

SspI

17441 CTGCTCAATTACCTGCACCACGCACATTGCTAACTAAGGGCACACTAGAACCAGAATATTTCAATTCAGTGTGTAGACTT 17520

GACGAGTTAATGGACGTGGTGCGTGTAACGATTGATTCCCGTGTGATCTTGGTCTTATAAAGTTAAGTCACACATCTGAA

MKTIGPDMFLGTCRRCPAEIVDTVSAL

17521 ATGAAAACTATAGGTCCAGACATGTTCCTCGGAACTTGTCGGCGTTGTCCTGCTGAAATTGTTGACACTGTGAGTGCTTT 17600

TACTTTTGATATCCAGGTCTGTACAAGGAGCCTTGAACAGCCGCAACAGGACGACTTTAACAACTGTGACACTCACGAAA

VYDNKLKAHKDKSAQCFKMFYKGVITH

HindIIIDraIPsiI

17601 GGTTTATGATAATAAGCTTAAAGCACATAAAGACAAATCAGCTCAATGCTTTAAAATGTTTTATAAGGGTGTTATCACGC 17680

CCAAATACTATTATTCGAATTTCGTGTATTTCTGTTTAGTCGAGTTACGAAATTTTACAAAATATTCCCACAATAGTGCG

DVSSAINRPQIGVVREFLTRNPAWRK

EcoRI

17681 ATGATGTTTCATCTGCAATTAACAGGCCACAAATAGGCGTGGTAAGAGAATTCCTTACACGTAACCCTGCTTGGAGAAAA 17760

TACTACAAAGTAGACGTTAATTGTCCGGTGTTTATCCGCACCATTCTCTTAAGGAATGTGCATTGGGACGAACCTCTTTT

AVFISPYNSQNAVASKILGLPTQTVDS

PsiI

17761 GCTGTCTTTATTTCACCTTATAATTCACAGAATGCTGTAGCCTCAAAGATTTTGGGACTACCAACTCAAACTGTTGATTC 17840

CGACAGAAATAAAGTGGAATATTAAGTGTCTTACGACATCGGAGTTTCTAAAACCCTGATGGTTGAGTTTGACAACTAAG

SQGSEYDYVIFTQTTETAHSCNVNRFN

17841 ATCACAGGGCTCAGAATATGACTATGTCATATTCACTCAAACCACTGAAACAGCTCACTCTTGTAATGTAAACAGATTTA 17920

TAGTGTCCCGAGTCTTATACTGATACAGTATAAGTGAGTTTGGTGACTTTGTCGAGTGAGAACATTACATTTGTCTAAAT

VAITRAKVGILCIMSDRDLYDKLQFT

17921 ATGTTGCTATTACCAGAGCAAAAGTAGGCATACTTTGCATAATGTCTGATAGAGACCTTTATGACAAGTTGCAATTTACA 18000

TACAACGATAATGGTCTCGTTTTCATCCGTATGAAACGTATTACAGACTATCTCTGGAAATACTGTTCAACGTTAAATGT

SLEIPRRNVATLQAENVTGLFKDCSKV

DraI

18001 AGTCTTGAAATTCCACGTAGGAATGTGGCAACTTTACAAGCTGAAAATGTAACAGGACTCTTTAAAGATTGTAGTAAGGT 18080

TCAGAACTTTAAGGTGCATCCTTACACCGTTGAAATGTTCGACTTTTACATTGTCCTGAGAAATTTCTAACATCATTCCA

ITGLHPTQAPTHLSVDTKFKTEGLCVD

18081 AATCACTGGGTTACATCCTACACAGGCACCTACACACCTCAGTGTTGACACTAAATTCAAAACTGAAGGTTTATGTGTTG 18160

TTAGTGACCCAATGTAGGATGTGTCCGTGGATGTGTGGAGTCACAACTGTGATTTAAGTTTTGACTTCCAAATACACAAC

IPGIPKDMTYRRLISMMGFKMNYQVN

Bsu36IDraI

18161 ACATACCTGGCATACCTAAGGACATGACCTATAGAAGACTCATCTCTATGATGGGTTTTAAAATGAATTATCAAGTTAAT 18240

TGTATGGACCGTATGGATTCCTGTACTGGATATCTTCTGAGTAGAGATACTACCCAAAATTTTACTTAATAGTTCAATTA

GYPNMFITREEAIRHVRAWIGFDVEGC

18241 GGTTACCCTAACATGTTTATCACCCGCGAAGAAGCTATAAGACATGTACGTGCATGGATTGGCTTCGATGTCGAGGGGTG 18320

CCAATGGGATTGTACAAATAGTGGGCGCTTCTTCGATATTCTGTACATGCACGTACCTAACCGAAGCTACAGCTCCCCAC

HATREAVGTNLPLQLGFSTGVNLVAVP

KpnIHpaI

18321 TCATGCTACTAGAGAAGCTGTTGGTACCAATTTACCTTTACAGCTAGGTTTTTCTACAGGTGTTAACCTAGTTGCTGTAC 18400

AGTACGATGATCTCTTCGACAACCATGGTTAAATGGAAATGTCGATCCAAAAAGATGTCCACAATTGGATCAACGACATG

TGYVDTPNNTDFSRVSAKPPPGDQFK

DraI

18401 CTACAGGTTATGTTGATACACCTAATAATACAGATTTTTCCAGAGTTAGTGCTAAACCACCGCCTGGAGATCAATTTAAA 18480

GATGTCCAATACAACTATGTGGATTATTATGTCTAAAAAGGTCTCAATCACGATTTGGTGGCGGACCTCTAGTTAAATTT

HLIPLMYKGLPWNVVRIKIVQMLSDTL

BsrGIBsrGI

18481 CACCTCATACCACTTATGTACAAAGGACTTCCTTGGAATGTAGTGCGTATAAAGATTGTACAAATGTTAAGTGACACACT 18560

GTGGAGTATGGTGAATACATGTTTCCTGAAGGAACCTTACATCACGCATATTTCTAACATGTTTACAATTCACTGTGTGA

KNLSDRVVFVLWAHGFELTSMKYFVKI

18561 TAAAAATCTCTCTGACAGAGTCGTATTTGTCTTATGGGCACATGGCTTTGAGTTGACATCTATGAAGTATTTTGTGAAAA 18640

ATTTTTAGAGAGACTGTCTCAGCATAAACAGAATACCCGTGTACCGAAACTCAACTGTAGATACTTCATAAAACACTTTT

GPERTCCLCDRRATCFSTASDTYACW

18641 TAGGACCTGAGCGCACCTGTTGTCTATGTGATAGACGTGCCACATGCTTTTCCACTGCTTCAGACACTTATGCCTGTTGG 18720

ATCCTGGACTCGCGTGGACAACAGATACACTATCTGCACGGTGTACGAAAAGGTGACGAAGTCTGTGAATACGGACAACC

HHSIGFDYVYNPFMIDVQQWGFTGNLQ

18721 CATCATTCTATTGGATTTGATTACGTCTATAATCCGTTTATGATTGATGTTCAACAATGGGGTTTTACAGGTAACCTACA 18800

GTAGTAAGATAACCTAAACTAATGCAGATATTAGGCAAATACTAACTACAAGTTGTTACCCCAAAATGTCCATTGGATGT

SNHDLYCQVHGNAHVASCDAIMTRCLA

NcoIBspHI

18801 AAGCAACCATGATCTGTATTGTCAAGTCCATGGTAATGCACATGTAGCTAGTTGTGATGCAATCATGACTAGGTGTCTAG 18880

TTCGTTGGTACTAGACATAACAGTTCAGGTACCATTACGTGTACATCGATCAACACTACGTTAGTACTGATCCACAGATC

VHECFVKRVDWTIEYPIIGDELKINA

AseI

18881 CTGTCCACGAGTGCTTTGTTAAGCGTGTTGACTGGACTATTGAATATCCTATAATTGGTGATGAACTGAAGATTAATGCG 18960

GACAGGTGCTCACGAAACAATTCGCACAACTGACCTGATAACTTATAGGATATTAACCACTACTTGACTTCTAATTACGC

ACRKVQHMVVKAALLADKFPVLHDIGN

18961 GCTTGTAGAAAGGTTCAACACATGGTTGTTAAAGCTGCATTATTAGCAGACAAATTCCCAGTTCTTCACGACATTGGTAA 19040

CGAACATCTTTCCAAGTTGTGTACCAACAATTTCGACGTAATAATCGTCTGTTTAAGGGTCAAGAAGTGCTGTAACCATT

PKAIKCVPQADVEWKFYDAQPCSDKAY

HindIIIPsiI

19041 CCCTAAAGCTATTAAGTGTGTACCTCAAGCTGATGTAGAATGGAAGTTCTATGATGCACAGCCTTGTAGTGACAAAGCTT 19120

GGGATTTCGATAATTCACACATGGAGTTCGACTACATCTTACCTTCAAGATACTACGTGTCGGAACATCACTGTTTCGAA

KIEELFYSYATHSDKFTDGVCLFWNC

19121 ATAAAATAGAAGAATTATTCTATTCTTATGCCACACATTCTGACAAATTCACAGATGGTGTATGCCTATTTTGGAATTGC 19200

TATTTTATCTTCTTAATAAGATAAGAATACGGTGTGTAAGACTGTTTAAGTGTCTACCACATACGGATAAAACCTTAACG

NVDRYPANSIVCRFDTRVLSNLNLPGC

EcoRV

19201 AATGTCGATAGATATCCTGCTAATTCCATTGTTTGTAGATTTGACACTAGAGTGCTATCTAACCTTAACTTGCCTGGTTG 19280

TTACAGCTATCTATAGGACGATTAAGGTAACAAACATCTAAACTGTGATCTCACGATAGATTGGAATTGAACGGACCAAC

DGGSLYVNKHAFHTPAFDKSAFVNLKQ

NsiIDraI

19281 TGATGGTGGCAGTTTGTATGTAAATAAACATGCATTCCACACACCAGCTTTTGATAAAAGTGCTTTTGTTAATTTAAAAC 19360

ACTACCACCGTCAAACATACATTTATTTGTACGTAAGGTGTGTGGTCGAAAACTATTTTCACGAAAACAATTAAATTTTG

LPFFYYSDSPCESHGKQVVSDIDYVP

19361 AATTACCATTTTTCTATTACTCTGACAGTCCATGTGAGTCTCATGGAAAACAAGTAGTGTCAGATATAGATTATGTACCA 19440

TTAATGGTAAAAAGATAATGAGACTGTCAGGTACACTCAGAGTACCTTTTGTTCATCACAGTCTATATCTAATACATGGT

LKSATCITRCNLGGAVCRHHANEYRLY

19441 CTAAAGTCTGCTACGTGTATAACACGTTGCAATTTAGGTGGTGCTGTCTGTAGACATCATGCTAATGAGTACAGATTGTA 19520

GATTTCAGACGATGCACATATTGTGCAACGTTAAATCCACCACGACAGACATCTGTAGTACGATTACTCATGTCTAACAT

LDAYNMMISAGFSLWVYKQFDTYNLWN

PsiIPvuIIPsiI

19521 TCTCGATGCTTATAACATGATGATCTCAGCTGGCTTTAGCTTGTGGGTTTACAAACAATTTGATACTTATAACCTCTGGA 19600

AGAGCTACGAATATTGTACTACTAGAGTCGACCGAAATCGAACACCCAAATGTTTGTTAAACTATGAATATTGGAGACCT

TFTRLQSLENVAFNVVNKGHFDGQQG

19601 ACACTTTTACAAGACTTCAGAGTTTAGAAAATGTGGCTTTTAATGTTGTAAATAAGGGACACTTTGATGGACAACAGGGT 19680

TGTGAAAATGTTCTGAAGTCTCAAATCTTTTACACCGAAAATTACAACATTTATTCCCTGTGAAACTACCTGTTGTCCCA

EVPVSIINNTVYTKVDGVDVELFENKT

AseI

19681 GAAGTACCAGTTTCTATCATTAATAACACTGTTTACACAAAAGTTGATGGTGTTGATGTAGAATTGTTTGAAAATAAAAC 19760

CTTCATGGTCAAAGATAGTAATTATTGTGACAAATGTGTTTTCAACTACCACAACTACATCTTAACAAACTTTTATTTTG

TLPVNVAFELWAKRNIKPVPEVKILNN

19761 AACATTACCTGTTAATGTAGCATTTGAGCTTTGGGCTAAGCGCAACATTAAACCAGTACCAGAGGTGAAAATACTCAATA 19840

TTGTAATGGACAATTACATCGTAAACTCGAAACCCGATTCGCGTTGTAATTTGGTCATGGTCTCCACTTTTATGAGTTAT

LGVDIAANTVIWDYKRDAPAHISTIG

19841 ATTTGGGTGTGGACATTGCTGCTAATACTGTGATCTGGGACTACAAAAGAGATGCTCCAGCACATATATCTACTATTGGT 19920

TAAACCCACACCTGTAACGACGATTATGACACTAGACCCTGATGTTTTCTCTACGAGGTCGTGTATATAGATGATAACCA

VCSMTDIAKKPTETICAPLTVFFDGRV

ApaLI

19921 GTTTGTTCTATGACTGACATAGCCAAGAAACCAACTGAAACGATTTGTGCACCACTCACTGTCTTTTTTGATGGTAGAGT 20000

CAAACAAGATACTGACTGTATCGGTTCTTTGGTTGACTTTGCTAAACACGTGGTGAGTGACAGAAAAAACTACCATCTCA

DGQVDLFRNARNGVLITEGSVKGLQPS

20001 TGATGGTCAAGTAGACTTATTTAGAAATGCCCGTAATGGTGTTCTTATTACAGAAGGTAGTGTTAAAGGTTTACAACCAT 20080

ACTACCAGTTCATCTGAATAAATCTTTACGGGCATTACCACAAGAATAATGTCTTCCATCACAATTTCCAAATGTTGGTA

VGPKQASLNGVTLIGEAVKTQFNYYK

AseIPsiI

20081 CTGTAGGTCCCAAACAAGCTAGTCTTAATGGAGTCACATTAATTGGAGAAGCCGTAAAAACACAGTTCAATTATTATAAG 20160

GACATCCAGGGTTTGTTCGATCAGAATTACCTCAGTGTAATTAACCTCTTCGGCATTTTTGTGTCAAGTTAATAATATTC

KVDGVVQQLPETYFTQSRNLQEFKPRS

DraI

20161 AAAGTTGATGGTGTTGTCCAACAATTACCTGAAACTTACTTTACTCAGAGTAGAAATTTACAAGAATTTAAACCCAGGAG 20240

TTTCAACTACCACAACAGGTTGTTAATGGACTTTGAATGAAATGAGTCTCATCTTTAAATGTTCTTAAATTTGGGTCCTC

QMEIDFLELAMDEFIERYKLEGYAFEH

EcoRIBstBI

20241 TCAAATGGAAATTGATTTCTTAGAATTAGCTATGGATGAATTCATTGAACGGTATAAATTAGAAGGCTATGCCTTCGAAC 20320

AGTTTACCTTTAACTAAAGAATCTTAATCGATACCTACTTAAGTAACTTGCCATATTTAATCTTCCGATACGGAAGCTTG

IVYGDFSHSQLGGLHLLIGLAKRFKE

AclI

20321 ATATCGTTTATGGAGATTTTAGTCATAGTCAGTTAGGTGGTTTACATCTACTGATTGGACTAGCTAAACGTTTTAAGGAA 20400

TATAGCAAATACCTCTAAAATCAGTATCAGTCAATCCACCAAATGTAGATGACTAACCTGATCGATTTGCAAAATTCCTT

SPFELEDFIPMDSTVKNYFITDAQTGS

FspI

20401 TCACCTTTTGAATTAGAAGATTTTATTCCTATGGACAGTACAGTTAAAAACTATTTCATAACAGATGCGCAAACAGGTTC 20480

AGTGGAAAACTTAATCTTCTAAAATAAGGATACCTGTCATGTCAATTTTTGATAAAGTATTGTCTACGCGTTTGTCCAAG

SKCVCSVIDLLLDDFVEIIKSQDLSVV

20481 ATCTAAGTGTGTGTGTTCTGTTATTGATTTATTACTTGATGATTTTGTTGAAATAATAAAATCCCAAGATTTATCTGTAG 20560

TAGATTCACACACACAAGACAATAACTAAATAATGAACTACTAAAACAACTTTATTATTTTAGGGTTCTAAATAGACATC

SKVVKVTIDYTEISFMLWCKDGHVET

MscI

20561 TTTCTAAGGTTGTCAAAGTGACTATTGACTATACAGAAATTTCATTTATGCTTTGGTGTAAAGATGGCCATGTAGAAACA 20640

AAAGATTCCAACAGTTTCACTGATAACTGATATGTCTTTAAAGTAAATACGAAACCACATTTCTACCGGTACATCTTTGT

FYPKLQSSQAWQPGVAMPNLYKMQRML

20641 TTTTACCCAAAATTACAATCTAGTCAAGCGTGGCAACCGGGTGTTGCTATGCCTAATCTTTACAAAATGCAAAGAATGCT 20720

AAAATGGGTTTTAATGTTAGATCAGTTCGCACCGTTGGCCCACAACGATACGGATTAGAAATGTTTTACGTTTCTTACGA

LEKCDLQNYGDSATLPKGIMMNVAKYT

20721 ATTAGAAAAGTGTGACCTTCAAAATTATGGTGATAGTGCAACATTACCTAAAGGCATAATGATGAATGTCGCAAAATATA 20800

TAATCTTTTCACACTGGAAGTTTTAATACCACTATCACGTTGTAATGGATTTCCGTATTACTACTTACAGCGTTTTATAT

QLCQYLNTLTLAVPYNMRVIHFGAGS

SspIDraI

20801 CTCAACTGTGTCAATATTTAAACACATTAACATTAGCTGTACCCTATAATATGAGAGTTATACATTTTGGTGCTGGTTCT 20880

GAGTTGACACAGTTATAAATTTGTGTAATTGTAATCGACATGGGATATTATACTCTCAATATGTAAAACCACGACCAAGA

DKGVAPGTAVLRQWLPTGTLLVDSDLN

PvuIIBglII

20881 GATAAAGGAGTTGCACCAGGTACAGCTGTTTTAAGACAGTGGTTGCCTACGGGTACGCTGCTTGTCGATTCAGATCTTAA 20960

CTATTTCCTCAACGTGGTCCATGTCGACAAAATTCTGTCACCAACGGATGCCCATGCGACGAACAGCTAAGTCTAGAATT

DFVSDADSTLIGDCATVHTANKWDLII

BsrGI

20961 TGACTTTGTCTCTGATGCAGATTCAACTTTGATTGGTGATTGTGCAACTGTACATACAGCTAATAAATGGGATCTCATTA 21040

ACTGAAACAGAGACTACGTCTAAGTTGAAACTAACCACTAACACGTTGACATGTATGTCGATTATTTACCCTAGAGTAAT

SDMYDPKTKNVTKENDSKEGFFTYIC

21041 TTAGTGATATGTACGACCCTAAGACTAAAAATGTTACAAAAGAAAATGACTCTAAAGAGGGTTTTTTCACTTACATTTGT 21120

AATCACTATACATGCTGGGATTCTGATTTTTACAATGTTTTCTTTTACTGAGATTTCTCCCAAAAAAGTGAATGTAAACA

GFIQQKLALGGSVAIKITEHSWNADLY

NheIPsiI

21121 GGGTTTATACAACAAAAGCTAGCTCTTGGAGGTTCCGTGGCTATAAAGATAACAGAACATTCTTGGAATGCTGATCTTTA 21200

CCCAAATATGTTGTTTTCGATCGAGAACCTCCAAGGCACCGATATTTCTATTGTCTTGTAAGAACCTTACGACTAGAAAT

KLMGHFAWWTAFVTNVNASSSEAFLIG

21201 TAAGCTCATGGGACACTTCGCATGGTGGACAGCCTTTGTTACTAATGTGAATGCGTCATCATCTGAAGCATTTTTAATTG 21280

ATTCGAGTACCCTGTGAAGCGTACCACCTGTCGGAAACAATGATTACACTTACGCAGTAGTAGACTTCGTAAAAATTAAC

CNYLGKPREQIDGYVMHANYIFWRNT

NsiI/SphI

21281 GATGTAATTATCTTGGCAAACCACGCGAACAAATAGATGGTTATGTCATGCATGCAAATTACATATTTTGGAGGAATACA 21360

CTACATTAATAGAACCGTTTGGTGCGCTTGTTTATCTACCAATACAGTACGTACGTTTAATGTATAAAACCTCCTTATGT

NPIQLSSYSLFDMSKFPLKLRGTAVMS

21361 AATCCAATTCAGTTGTCTTCCTATTCTTTATTTGACATGAGTAAATTTCCCCTTAAATTAAGGGGTACTGCTGTTATGTC 21440

TTAGGTTAAGTCAACAGAAGGATAAGAAATAAACTGTACTCATTTAAAGGGGAATTTAATTCCCCATGACGACAATACAG

LKEGQINDMILSLLSKGRLIIRENNRV

DraIPsiI

21441 TTTAAAAGAAGGTCAAATCAATGATATGATTTTATCTCTTCTTAGTAAAGGTAGACTTATAATTAGAGAAAACAACAGAG 21520

AAATTTTCTTCCAGTTTAGTTACTATACTAAAATAGAGAAGAATCATTTCCATCTGAATATTAATCTCTTTTGTTGTCTC

VISSDVLVNN*TNNVCFSCFIATSL*

HpaISpeI

21521 TTGTTATTTCTAGTGATGTTCTTGTTAACAACTAAACGAACAATGTTTGTTTTTCTTGTTTTATTGCCACTAGTCTCTAG 21600

AACAATAAAGATCACTACAAGAACAATTGTTGATTTGCTTGTTACAAACAAAAAGAACAAAATAACGGTGATCAGAGATC

SVC*SYNQNSITPCIH*FFHTWCLLP*

PmlI

21601 TCAGTGTGTTAATCTTACAACCAGAACTCAATTACCCCCTGCATACACTAATTCTTTCACACGTGGTGTTTATTACCCTG 21680

AGTCACACAATTAGAATGTTGGTCTTGAGTTAATGGGGGACGTATGTGATTAAGAAAGTGTGCACCACAAATAATGGGAC

QSFQILSFTFNSGLVLTFLFQCYLVPC

21681 ACAAAGTTTTCAGATCCTCAGTTTTACATTCAACTCAGGACTTGTTCTTACCTTTCTTTTCCAATGTTACTTGGTTCCAT 21760

TGTTTCAAAAGTCTAGGAGTCAAAATGTAAGTTGAGTCCTGAACAAGAATGGAAAGAAAAGGTTACAATGAACCAAGGTA

YTCLWDQWY*EV**PCPTI**WCLFC

21761 GCTATACATGTCTCTGGGACCAATGGTACTAAGAGGTTTGATAACCCTGTCCTACCATTTAATGATGGTGTTTATTTTGC 21840

CGATATGTACAGAGACCCTGGTTACCATGATTCTCCAAACTATTGGGACAGGATGGTAAATTACTACCACAAATAAAACG

FH*EV*HNKRLDFWYYFRFEDPVPTYC

BstBI

21841 TTCCACTGAGAAGTCTAACATAATAAGAGGCTGGATTTTTGGTACTACTTTAGATTCGAAGACCCAGTCCCTACTTATTG 21920

AAGGTGACTCTTCAGATTGTATTATTCTCCGACCTAAAAACCATGATGAAATCTAAGCTTCTGGGTCAGGGATGAATAAC

**RY*CCY*SL*ISIL**SIFGCLLPQ

21921 TTAATAACGCTACTAATGTTGTTATTAAAGTCTGTGAATTTCAATTTTGTAATGATCCATTTTTGGGTGTTTATTACCAC 22000

AATTATTGCGATGATTACAACAATAATTTCAGACACTTAAAGTTAAAACATTACTAGGTAAAAACCCACAAATAATGGTG

KQQKLDGK*VQSLF*CE*LHF*ICLS

22001 AAAAACAACAAAAGTTGGATGGAAAGTGAGTTCAGAGTTTATTCTAGTGCGAATAATTGCACTTTTGAATATGTCTCTCA 22080

TTTTTGTTGTTTTCAACCTACCTTTCACTCAAGTCTCAAATAAGATCACGCTTATTAACGTGAAAACTTATACAGAGAGT

AFSYGP*RKTG*FQKS*GICV*EY*WL

SspI

22081 GCCTTTTCTTATGGACCTTGAAGGAAAACAGGGTAATTTCAAAAATCTTAGGGAATTTGTGTTTAAGAATATTGATGGTT 22160

CGGAAAAGAATACCTGGAACTTCCTTTTGTCCCATTAAAGTTTTTAGAATCCCTTAAACACAAATTCTTATAACTACCAA

F*NIF*AHAY*FSA*SPSGFFGFRTIG

DraIAseIBsu36I

22161 ATTTTAAAATATATTCTAAGCACACGCCTATTAATTTAGTGCGTGATCTCCCTCAGGGTTTTTCGGCTTTAGAACCATTG 22240

TAAAATTTTATATAAGATTCGTGTGCGGATAATTAAATCACGCACTAGAGGGAGTCCCAAAAAGCCGAAATCTTGGTAAC

RFANRY*HH*VSNFTCFT*KLFDSW*

22241 GTAGATTTGCCAATAGGTATTAACATCACTAGGTTTCAAACTTTACTTGCTTTACATAGAAGTTATTTGACTCCTGGTGA 22320

CATCTAAACGGTTATCCATAATTGTAGTGATCCAAAGTTTGAAATGAACGAAATGTATCTTCAATAAACTGAGGACCACT

FFFRLDSWCCSLLCGLSST*DFSIKI*

PvuIIPstIAvrII

22321 TTCTTCTTCAGGTTGGACAGCTGGTGCTGCAGCTTATTATGTGGGTTATCTTCAACCTAGGACTTTTCTATTAAAATATA 22400

AAGAAGAAGTCCAACCTGTCGACCACGACGTCGAATAATACACCCAATAGAAGTTGGATCCTGAAAAGATAATTTTATAT

*KWNHYRCCRLCT*PSLRNKVYVEILH

ApaLI

22401 ATGAAAATGGAACCATTACAGATGCTGTAGACTGTGCACTTGACCCTCTCTCAGAAACAAAGTGTACGTTGAAATCCTTC 22480

TACTTTTACCTTGGTAATGTCTACGACATCTGACACGTGAACTGGGAGAGAGTCTTTGTTTCACATGCAACTTTAGGAAG

CRKRNLSNF*L*SPTNRIYC*IS*YY

SspI

22481 ACTGTAGAAAAAGGAATCTATCAAACTTCTAACTTTAGAGTCCAACCAACAGAATCTATTGTTAGATTTCCTAATATTAC 22560

TGACATCTTTTTCCTTAGATAGTTTGAAGATTGAAATCTCAGGTTGGTTGTCTTAGATAACAATCTAAAGGATTATAATG

KLVPFW*SF*RHQICICLCLEQEENQQ

22561 AAACTTGTGCCCTTTTGGTGAAGTTTTTAACGCCACCAGATTTGCATCTGTTTATGCTTGGAACAGGAAGAGAATCAGCA 22640

TTTGAACACGGGAAAACCACTTCAAAAATTGCGGTGGTCTAAACGTAGACAAATACGAACCTTGTCCTTCTCTTAGTCGT

LCC*LFCPI*FRIIFHF*VLWSVSY*I

22641 ACTGTGTTGCTGATTATTCTGTCCTATATAATTCCGCATCATTTTCCACTTTTAAGTGTTATGGAGTGTCTCCTACTAAA 22720

TGACACAACGACTAATAAGACAGGATATATTAAGGCGTAGTAAAAGGTGAAAATTCACAATACCTCACAGAGGATGATTT

K*SLLY*CLCRFICN*R**SQTNRSR

22721 TTAAATGATCTCTGCTTTACTAATGTCTATGCAGATTCATTTGTAATTAGAGGTGATGAAGTCAGACAAATCGCTCCAGG 22800

AATTTACTAGAGACGAAATGATTACAGATACGTCTAAGTAAACATTAATCTCCACTACTTCAGTCTGTTTAGCGAGGTCC

ANWKDC*L*L*ITR*FYRLRYSLEF*Q

PsiIPsiIEcoRI

22801 GCAAACTGGAAAGATTGCTGATTATAATTATAAATTACCAGATGATTTTACAGGCTGCGTTATAGCTTGGAATTCTAACA 22880

CGTTTGACCTTTCTAACGACTAATATTAATATTTAATGGTCTACTAAAATGTCCGACGCAATATCGAACCTTAAGATTGT

S*F*GWW*L*LPV*IV*EV*SQTF*ER

PsiI

22881 ATCTTGATTCTAAGGTTGGTGGTAATTATAATTACCTGTATAGATTGTTTAGGAAGTCTAATCTCAAACCTTTTGAGAGA 22960

TAGAACTAAGATTCCAACCACCATTAATATTAATGGACATATCTAACAAATCCTTCAGATTAGAGTTTGGAAAACTCTCT

YFN*NLSGR*HTL*WC*RF*LLLSFT

22961 GATATTTCAACTGAAATCTATCAGGCCGGTAGCACACCTTGTAATGGTGTTGAAGGTTTTAATTGTTACTTTCCTTTACA 23040

CTATAAAGTTGACTTTAGATAGTCCGGCCATCGTGTGGAACATTACCACAACTTCCAAAATTAACAATGAAAGGAAATGT

IIWFPTH*WCWLPTIQSSSTFF*TSTC

NdeIScaI

23041 ATCATATGGTTTCCAACCCACTAATGGTGTTGGTTACCAACCATACAGAGTAGTAGTACTTTCTTTTGAACTTCTACATG 23120

TAGTATACCAAAGGTTGGGTGATTACCACAACCAATGGTTGGTATGTCTCATCATCATGAAAGAAAACTTGAAGATGTAC

TSNCLWT*KVY*FG*KQMCQFQLQWFN

23121 CACCAGCAACTGTTTGTGGACCTAAAAAGTCTACTAATTTGGTTAAAAACAAATGTGTCAATTTCAACTTCAATGGTTTA 23200

GTGGTCGTTGACAAACACCTGGATTTTTCAGATGATTAAACCAATTTTTGTTTACACAGTTAAAGTTGAAGTTACCAAAT

RHRCSY*V*QKVSAFPTIWQRHC*HY

23201 ACAGGCACAGGTGTTCTTACTGAGTCTAACAAAAAGTTTCTGCCTTTCCAACAATTTGGCAGAGACATTGCTGACACTAC 23280

TGTCCGTGTCCACAAGAATGACTCAGATTGTTTTTCAAAGACGGAAAGGTTGTTAAACCGTCTCTGTAACGACTGTGATG

*CCP*STDT*DS*HYTMFFWWCQCYNT

PsiI

23281 TGATGCTGTCCGTGATCCACAGACACTTGAGATTCTTGACATTACACCATGTTCTTTTGGTGGTGTCAGTGTTATAACAC 23360

ACTACGACAGGCACTAGGTGTCTGTGAACTCTAAGAACTGTAATGTGGTACAAGAAAACCACCACAGTCACAATATTGTG

RNKYF*PGCCSLSGC*LHRSPCCYSCR

HpaI

23361 CAGGAACAAATACTTCTAACCAGGTTGCTGTTCTTTATCAGGATGTTAACTGCACAGAAGTCCCTGTTGCTATTCATGCA 23440

GTCCTTGTTTATGAAGATTGGTCCAACGACAAGAAATAGTCCTACAATTGACGTGTCTTCAGGGACAACGATAAGTACGT

STYSYLACLFYRF*CFSNTCRLFNRG

PmlI

23441 GATCAACTTACTCCTACTTGGCGTGTTTATTCTACAGGTTCTAATGTTTTTCAAACACGTGCAGGCTGTTTAATAGGGGC 23520

CTAGTTGAATGAGGATGAACCGCACAAATAAGATGTCCAAGATTACAAAAAGTTTGTGCACGTCCGACAAATTATCCCCG

*TCQQLI*V*HTHWCRYMR*LSDSD*F

NdeI

23521 TGAACATGTCAACAACTCATATGAGTGTGACATACCCATTGGTGCAGGTATATGCGCTAGTTATCAGACTCAGACTAATT 23600

ACTTGTACAGTTGTTGAGTATACTCACACTGTATGGGTAACCACGTCCATATACGCGATCAATAGTCTGAGTCTGATTAA

SSAGT*CS*SIHHCLHYVTWCRKFSCL

23601 CTCCTCGGCGGGCACGTAGTGTAGCTAGTCAATCCATCATTGCCTACACTATGTCACTTGGTGCAGAAAATTCAGTTGCT 23680

GAGGAGCCGCCCGTGCATCACATCGATCAGTTAGGTAGTAACGGATGTGATACAGTGAACCACGTCTTTTAAGTCAACGA

L**LYCHTHKFYY*CYHRNSTSVYDQ

23681 TACTCTAATAACTCTATTGCCATACCCACAAATTTTACTATTAGTGTTACCACAGAAATTCTACCAGTGTCTATGACCAA 23760

ATGAGATTATTGAGATAACGGTATGGGTGTTTAAAATGATAATCACAATGGTGTCTTTAAGATGGTCACAGATACTGGTT

DISRLYNVHLW*FN*MQQSFVAIWQFL

BsrGIBsrGIBsrGI

23761 GACATCAGTAGATTGTACAATGTACATTTGTGGTGATTCAACTGAATGCAGCAATCTTTTGTTGCAATATGGCAGTTTTT 23840

CTGTAGTCATCTAACATGTTACATGTAAACACCACTAAGTTGACTTACGTCGTTAGAAAACAACGTTATACCGTCAAAAA

YTIKPCFNWNSC*TRQKHPRSFCTSQT

23841 GTACACAATTAAACCGTGCTTTAACTGGAATAGCTGTTGAACAAGACAAAAACACCCAAGAAGTTTTTGCACAAGTCAAA 23920

CATGTGTTAATTTGGCACGAAATTGACCTTATCGACAACTTGTTCTGTTTTTGTGGGTTCTTCAAAAACGTGTTCAGTTT

NLQNTTN*RFWWF*FFTNITRSIKTK

SspI

23921 CAAATTTACAAAACACCACCAATTAAAGATTTTGGTGGTTTTAATTTTTCACAAATATTACCAGATCCATCAAAACCAAG 24000

GTTTAAATGTTTTGTGGTGGTTAATTTCTAAAACCACCAAAATTAAAAAGTGTTTATAATGGTCTAGGTAGTTTTGGTTC

QEVIY*RSTFQQSDTCRCWLHQTIW*L

BglII

24001 CAAGAGGTCATTTATTGAAGATCTACTTTTCAACAAAGTGACACTTGCAGATGCTGGCTTCATCAAACAATATGGTGATT 24080

GTTCTCCAGTAAATAACTTCTAGATGAAAAGTTGTTTCACTGTGAACGTCTACGACCGAAGTAGTTTGTTATACCACTAA

PW*YCC*RPHLCTKV*RPYCFATFAHR

ApaLI

24081 GCCTTGGTGATATTGCTGCTAGAGACCTCATTTGTGCACAAAAGTTTAACGGCCTTACTGTTTTGCCACCTTTGCTCACA 24160

CGGAACCACTATAACGACGATCTCTGGAGTAAACACGTGTTTTCAAATTGCCGGAATGACAAAACGGTGGAAACGAGTGT

*NDCSIHFCTVSGYNHFWLDLWCRCC

24161 GATGAAATGATTGCTCAATACACTTCTGCACTGTTAGCGGGTACAATCACTTCTGGTTGGACCTTTGGTGCAGGTGCTGC 24240

CTACTTTACTAACGAGTTATGTGAAGACGTGACAATCGCCCATGTTAGTGAAGACCAACCTGGAAACCACGTCCACGACG

ITNTICYANGL*V*WYWSYTECSL*EP

24241 ATTACAAATACCATTTGCTATGCAAATGGCTTATAGGTTTAATGGTATTGGAGTTACACAGAATGTTCTCTATGAGAACC 24320

TAATGTTTATGGTAAACGATACGTTTACCGAATATCCAAATTACCATAACCTCAATGTGTCTTACAAGAGATACTCTTGG

KIDCQPI**CYWQNSRLTFFHSKCTWK

ApaLI

24321 AAAAATTGATTGCCAACCAATTTAATAGTGCTATTGGCAAAATTCAAGACTCACTTTCTTCCACAGCAAGTGCACTTGGA 24400

TTTTTAACTAACGGTTGGTTAAATTATCACGATAACCGTTTTAAGTTCTGAGTGAAAGAAGGTGTCGTTCACGTGAACCT

TSRCGQPKCTSFKHAC*TT*LQFWCN

HindIIIDraI

24401 AAACTTCAAGATGTGGTCAACCAAAATGCACAAGCTTTAAACACGCTTGTTAAACAACTTAGCTCCAATTTTGGTGCAAT 24480

TTTGAAGTTCTACACCAGTTGGTTTTACGTGTTCGAAATTTGTGCGAACAATTTGTTGAATCGAGGTTAAAACCACGTTA

FKCFK*YPFTS*QS*G*SAN**VDHRQ

DraIEcoRV

24481 TTCAAGTGTTTTAAATGATATCCTTTCACGTCTTGACAAAGTTGAGGCTGAAGTGCAAATTGATAGGTTGATCACAGGCA 24560

AAGTTCACAAAATTTACTATAGGAAAGTGCAGAACTGTTTCAACTCCGACTTCACGTTTAACTATCCAACTAGTGTCCGT

TSKFADICDSTIN*SCRNQSFC*SCCY

NdeIAseIPstI

24561 GACTTCAAAGTTTGCAGACATATGTGACTCAACAATTAATTAGAGCTGCAGAAATCAGAGCTTCTGCTAATCTTGCTGCT 24640

CTGAAGTTTCAAACGTCTGTATACACTGAGTTGTTAATTAATCTCGACGTCTTTAGTCTCGAAGACGATTAGAACGACGA

*NVRVCTWTIKKS*FLWKGLSSYVLP

24641 ACTAAAATGTCAGAGTGTGTACTTGGACAATCAAAAAGAGTTGATTTTTGTGGAAAGGGCTATCATCTTATGTCCTTCCC 24720

TGATTTTACAGTCTCACACATGAACCTGTTAGTTTTTCTCAACTAAAAACACCTTTCCCGATAGTAGAATACAGGAAGGG

SVSTSWCSLLACDLCPCTRKELHNCSC

24721 TCAGTCAGCACCTCATGGTGTAGTCTTCTTGCATGTGACTTATGTCCCTGCACAAGAAAAGAACTTCACAACTGCTCCTG 24800

AGTCAGTCGTGGAGTACCACATCAGAAGAACGTACACTGAATACAGGGACGTGTTCTTTTCTTGAAGTGTTGACGAGGAC

HLS*WKSTLSS*RCLCFKWHTLVCNTK

BspHI

24801 CCATTTGTCATGATGGAAAAGCACACTTTCCTCGTGAAGGTGTCTTTGTTTCAAATGGCACACACTGGTTTGTAACACAA 24880

GGTAAACAGTACTACCTTTTCGTGTGAAAGGAGCACTTCCACAGAAACAAAGTTTACCGTGTGTGACCAAACATTGTGTT

EFL*TTNHYYRQHICVW*L*CCNRNC

24881 AGGAATTTTTATGAACCACAAATCATTACTACAGACAACACATTTGTGTCTGGTAACTGTGATGTTGTAATAGGAATTGT 24960

TCCTTAAAAATACTTGGTGTTTAGTAATGATGTCTGTTGTGTAAACACAGACCATTGACACTACAACATTATCCTTAACA

QQHSL*SFAT*IRLIQGGVR*IF*ESY

SspI

24961 CAACAACACAGTTTATGATCCTTTGCAACCTGAATTAGACTCATTCAAGGAGGAGTTAGATAAATATTTTAAGAATCATA 25040

GTTGTTGTGTCAAATACTAGGAAACGTTGGACTTAATCTGAGTAAGTTCCTCCTCAATCTATTTATAAAATTCTTAGTAT

ITRC*FR*HLWH*CFSCKHSKRN*PPQ

AseI

25041 CATCACCAGATGTTGATTTAGGTGACATCTCTGGCATTAATGCTTCAGTTGTAAACATTCAAAAAGAAATTGACCGCCTC 25120

GTAGTGGTCTACAACTAAATCCACTGTAGAGACCGTAATTACGAAGTCAACATTTGTAAGTTTTTCTTTAACTGGCGGAG

*GCQEFK*ISHRSPRTWKV*AVYKMA

DraIClaI*MscINcoI

25121 AATGAGGTTGCCAAGAATTTAAATGAATCTCTCATCGATCTCCAAGAACTTGGAAAGTATGAGCAGTATATAAAATGGCC 25200

TTACTCCAACGGTTCTTAAATTTACTTAGAGAGTAGCTAGAGGTTCTTGAACCTTTCATACTCGTCATATATTTTACCGG

MVHLARFYSWLDCHSNGDNYALLYDQL

25201 ATGGTACATTTGGCTAGGTTTTATAGCTGGCTTGATTGCCATAGTAATGGTGACAATTATGCTTTGCTGTATGACCAGTT 25280

TACCATGTAAACCGATCCAAAATATCGACCGAACTAACGGTATCATTACCACTGTTAATACGAAACGACATACTGGTCAA

L*LSQGLLFLWILLQI**RRL*ASAQR

BamHI

25281 GCTGTAGTTGTCTCAAGGGCTGTTGTTCTTGTGGATCCTGCTGCAAATTTGATGAAGACGACTCTGAGCCAGTGCTCAAA 25360

CGACATCAACAGAGTTCCCGACAACAAGAACACCTAGGACGACGTTTAAACTACTTCTGCTGAGACTCGGTCACGAGTTT

SQITLHINELMDLFMRIFTIGTVTLK

MfeI

25361 GGAGTCAAATTACATTACACATAAACGAACTTATGGATTTGTTTATGAGAATCTTCACAATTGGAACTGTAACTTTGAAG 25440

CCTCAGTTTAATGTAATGTGTATTTGCTTGAATACCTAAACAAATACTCTTAGAAGTGTTAACCTTGACATTGAAACTTC

QGEIKDATPSDFVRATATIPIQASLPF

25441 CAAGGTGAAATCAAGGATGCTACTCCTTCAGATTTTGTTCGCGCTACTGCAACGATACCGATACAAGCCTCACTCCCTTT 25520

GTTCCACTTTAGTTCCTACGATGAGGAAGTCTAAAACAAGCGCGATGACGTTGCTATGGCTATGTTCGGAGTGAGGGAAA

GWLIVGVALLAVFQSASKIITLKKRWQ

AfeI

25521 CGGATGGCTTATTGTTGGCGTTGCACTTCTTGCTGTTTTTCAGAGCGCTTCCAAAATCATAACCCTCAAAAAGAGATGGC 25600

GCCTACCGAATAACAACCGCAACGTGAAGAACGACAAAAAGTCTCGCGAAGGTTTTAGTATTGGGAGTTTTTCTCTACCG

LALSKGVHFVCNLLLLFVTVYSHLLL

25601 AACTAGCACTCTCCAAGGGTGTTCACTTTGTTTGCAACTTGCTGTTGTTGTTTGTAACAGTTTACTCACACCTTTTGCTC 25680

TTGATCGTGAGAGGTTCCCACAAGTGAAACAAACGTTGAACGACAACAACAAACATTGTCAAATGAGTGTGGAAAACGAG

VAAGLEAPFLYLYALVYFLQSINFVRI

25681 GTTGCTGCTGGCCTTGAAGCCCCTTTTCTCTATCTTTATGCTTTAGTCTACTTCTTGCAGAGTATAAACTTTGTAAGAAT 25760

CAACGACGACCGGAACTTCGGGGAAAAGAGATAGAAATACGAAATCAGATGAAGAACGTCTCATATTTGAAACATTCTTA

IMRLWLCWKCRSKNPLLYDANYFLCWH

25761 AATAATGAGGCTTTGGCTTTGCTGGAAATGCCGTTCCAAAAACCCATTACTTTATGATGCCAACTATTTTCTTTGCTGGC 25840

TTATTACTCCGAAACCGAAACGACCTTTACGGCAAGGTTTTTGGGTAATGAAATACTACGGTTGATAAAAGAAACGACCG

TNCYDYCIPYNSVTSSIVITSGDGTT

MfeI

25841 ATACTAATTGTTACGACTATTGTATACCTTACAATAGTGTAACTTCTTCAATTGTCATTACTTCAGGTGATGGCACAACA 25920

TATGATTAACAATGCTGATAACATATGGAATGTTATCACATTGAAGAAGTTAACAGTAATGAAGTCCACTACCGTGTTGT

SPISEHDYQIGGYTEKWESGVKDCVVL

25921 AGTCCTATTTCTGAACATGACTACCAGATTGGTGGTTATACTGAAAAATGGGAATCTGGAGTAAAAGACTGTGTTGTATT 26000

TCAGGATAAAGACTTGTACTGATGGTCTAACCACCAATATGACTTTTTACCCTTAGACCTCATTTTCTGACACAACATAA

HSYFTSDYYQLYSTQLSTDTGVEHVTF

PvuIIMfeI

26001 ACACAGTTACTTCACTTCAGACTATTACCAGCTGTACTCAACTCAATTGAGTACAGACACTGGTGTTGAACATGTTACCT 26080

TGTGTCAATGAAGTGAAGTCTGATAATGGTCGACATGAGTTGAGTTAACTCATGTCTGTGACCACAACTTGTACAATGGA

FIYNKIVDEPEEHVQIHTIDGSSGVV

BspEI

26081 TCTTCATCTACAATAAAATTGTTGATGAGCCTGAAGAACATGTCCAAATTCACACAATCGACGGTTCATCCGGAGTTGTT 26160

AGAAGTAGATGTTATTTTAACAACTACTCGGACTTCTTGTACAGGTTTAAGTGTGTTAGCTGCCAAGTAGGCCTCAACAA

NPVMEPIYDEPTTTTSVPL*AQADEYE

26161 AATCCAGTAATGGAACCAATTTATGATGAACCGACGACGACTACTAGCGTGCCTTTGTAAGCACAAGCTGATGAGTACGA 26240

TTAGGTCATTACCTTGGTTAAATACTACTTGGCTGCTGCTGATGATCGCACGGAAACATTCGTGTTCGACTACTCATGCT

LMYSFVSEETGTLIVNSVLLFLAFVVF

26241 ACTTATGTACTCATTCGTTTCGGAAGAGACAGGTACGTTAATAGTTAATAGCGTACTTCTTTTTCTTGCTTTCGTGGTAT 26320

TGAATACATGAGTAAGCAAAGCCTTCTCTGTCCATGCAATTATCAATTATCGCATGAAGAAAAAGAACGAAAGCACCATA

LLVTLAILTALRLCAYCCNIVNVSLV

SspIHpaI

26321 TCTTGCTAGTTACACTAGCCATCCTTACTGCGCTTCGATTGTGTGCGTACTGCTGCAATATTGTTAACGTGAGTCTTGTA 26400

AGAACGATCAATGTGATCGGTAGGAATGACGCGAAGCTAACACACGCATGACGACGTTATAACAATTGCACTCAGAACAT

KPSFYVYSRVKNLNSSRVPDLLV*TN*

EcoRIXbaI

26401 AAACCTTCTTTTTACGTTTACTCTCGTGTTAAAAATCTGAATTCTTCTAGAGTTCCTGATCTTCTGGTCTAAACGAACTA 26480

TTTGGAAGAAAAATGCAAATGAGAGCACAATTTTTAGACTTAAGAAGATCTCAAGGACTAGAAGACCAGATTTGCTTGAT

ILY*FFCLEL*F*PWQIPTVLLPLKSL

SspINcoI

26481 AATATTATATTAGTTTTTCTGTTTGGAACTTTAATTTTAGCCATGGCAGATTCCAACGGTACTATTACCGTTGAAGAGCT 26560

TTATAATATAATCAAAAAGACAAACCTTGAAATTAAAATCGGTACCGTCTAAGGTTGCCATGATAATGGCAACTTCTCGA

KSSLNNGT***VSYSLHGFVFYNLPM

26561 TAAAAAGCTCCTTGAACAATGGAACCTAGTAATAGGTTTCCTATTCCTTACATGGATTTGTCTTCTACAATTTGCCTATG 26640

ATTTTTCGAGGAACTTGTTACCTTGGATCATTATCCAAAGGATAAGGAATGTACCTAAACAGAAGATGTTAAACGGATAC

PTGIGFCI*LS*FSSGCYGQ*L*LVLC

MscI

26641 CCAACAGGAATAGGTTTTTGTATATAATTAAGTTAATTTTCCTCTGGCTGTTATGGCCAGTAACTTTAGCTTGTTTTGTG 26720

GGTTGTCCTTATCCAAAAACATATATTAATTCAATTAAAAGGAGACCGACAATACCGGTCATTGAAATCGAACAAAACAC

LLLFTE*IGSPVELLSQWLVL*A*CGS

AgeI

26721 CTTGCTGCTGTTTACAGAATAAATTGGATCACCGGTGGAATTGCTATCGCAATGGCTTGTCTTGTAGGCTTGATGTGGCT 26800

GAACGACGACAAATGTCTTATTTAACCTAGTGGCCACCTTAACGATAGCGTTACCGAACAGAACATCCGAACTACACCGA

ATSLLLSDCLRVRVPCGHSIQKLTFF

BsiWI/MluI

26801 CAGCTACTTCATTGCTTCTTTCAGACTGTTTGCGCGTACGCGTTCCATGTGGTCATTCAATCCAGAAACTAACATTCTTC 26880

GTCGATGAAGTAACGAAGAAAGTCTGACAAACGCGCATGCGCAAGGTACACCAGTAAGTTAGGTCTTTGATTGTAAGAAG

STCHSMALF*PDRF*KVNS*SEL*SFV

NcoIXbaI

26881 TCAACGTGCCACTCCATGGCACTATTCTGACCAGACCGCTTCTAGAAAGTGAACTCGTAATCGGAGCTGTGATCCTTCGT 26960

AGTTGCACGGTGAGGTACCGTGATAAGACTGGTCTGGCGAAGATCTTTCACTTGAGCATTAGCCTCGACACTAGGAAGCA

DIFVLLDTI*DAVTSRTCLKKSLLLHH

26961 GGACATCTTCGTATTGCTGGACACCATCTAGGACGCTGTGACATCAAGGACCTGCCTAAAGAAATCACTGTTGCTACATC 27040

CCTGTAGAAGCATAACGACCTGTGGTAGATCCTGCGACACTGTAGTTCCTGGACGGATTTCTTTAGTGACAACGATGTAG

ERFLITNWELRSV*QVTQVLLHTVAT

27041 ACGAACGCTTTCTTATTACAAATTGGGAGCTTCGCAGCGTGTAGCAGGTGACTCAGGTTTTGCTGCATACAGTCGCTACA 27120

TGCTTGCGAAAGAATAATGTTTAACCCTCGAAGCGTCGCACATCGTCCACTGAGTCCAAAACGACGTATGTCAGCGATGT

GLATIN*TQTIPVAVTILLCLYSK*QQ

SspIBsrGI

27121 GGATTGGCAACTATAAATTAAACACAGACCATTCCAGTAGCAGTGACAATATTGCTTTGCTTGTACAGTAAGTGACAACA 27200

CCTAACCGTTGATATTTAATTTGTGTCTGGTAAGGTCATCGTCACTGTTATAACGAAACGAACATGTCATTCACTGTTGT

MFHLVDFQVTIAEILLIIMRTFKVSIW

DraI

27201 GATGTTTCATCTCGTTGACTTTCAGGTTACTATAGCAGAGATATTACTAATTATTATGAGGACTTTTAAAGTTTCCATTT 27280

CTACAAAGTAGAGCAACTGAAAGTCCAATGATATCGTCTCTATAATGATTAATAATACTCCTGAAAATTTCAAAGGTAAA

NLDYIINLIIKNLSKSLTENKYSQLD

SspI

27281 GGAATCTTGATTACATCATAAACCTCATAATTAAAAATTTATCTAAGTCACTAACTGAGAATAAATATTCTCAATTAGAT 27360

CCTTAGAACTAATGTAGTATTTGGAGTATTAATTTTTAAATAGATTCAGTGATTGACTCTTATTTATAAGAGTTAATCTA

EEQPMEID*TNMKIILFLALITLATCE

27361 GAAGAGCAACCAATGGAGATTGATTAAACGAACATGAAAATTATTCTTTTCTTGGCACTGATAACACTCGCTACTTGTGA 27440

CTTCTCGTTGGTTACCTCTAACTAATTTGCTTGTACTTTTAATAAGAAAAGAACCGTGACTATTGTGAGCGATGAACACT

LYHYQECVRGTTVLLKEPCSSGTYEGN

ScaIDraI

27441 GCTTTATCACTACCAAGAGTGTGTTAGAGGTACAACAGTACTTTTAAAAGAACCTTGCTCTTCTGGAACATACGAGGGCA 27520

CGAAATAGTGATGGTTCTCACACAATCTCCATGTTGTCATGAAAATTTTCTTGGAACGAGAAGACCTTGTATGCTCCCGT

SPFHPLADNKFALTCFSTQFAFACPD

27521 ATTCACCATTTCATCCTCTAGCTGATAACAAATTTGCACTGACTTGCTTTAGCACTCAATTTGCTTTTGCTTGTCCTGAC 27600

TAAGTGGTAAAGTAGGAGATCGACTATTGTTTAAACGTGACTGAACGAAATCGTGAGTTAAACGAAAACGAACAGGACTG

GVKHVYQLRARSVSPKLFIRQEEVQEL

27601 GGCGTAAAACACGTCTATCAGTTACGTGCCAGATCAGTTTCACCTAAACTGTTCATCAGACAAGAGGAAGTTCAAGAACT 27680

CCGCATTTTGTGCAGATAGTCAATGCACGGTCTAGTCAAAGTGGATTTGACAAGTAGTCTGTTCTCCTTCAAGTTCTTGA

YSPIFLIVAAIVFITLCFTLKRKTE*L

PsiI

27681 TTACTCTCCAATTTTTCTTATTGTTGCGGCAATAGTGTTTATAACACTTTGCTTCACACTCAAAAGAAAGACAGAATGAT 27760

AATGAGAGGTTAAAAAGAATAACAACGCCGTTATCACAAATATTGTGAAACGAAGTGTGAGTTTTCTTTCTGTCTTACTA

NFH*LTSICAF*PFCYSLF*LCLLSF

AseI

27761 TGAACTTTCATTAATTGACTTCTATTTGTGCTTTTTAGCCTTTCTGCTATTCCTTGTTTTAATTATGCTTATTATCTTTT 27840

ACTTGAAAGTAATTAACTGAAGATAAACACGAAAAATCGGAAAGACGATAAGGAACAAAATTAATACGAATAATAGAAAA

GSHLNCKIIMKLVTPKRT*NFLFS*ES

27841 GGTTCTCACTTGAACTGCAAGATCATAATGAAACTTGTCACGCCTAAACGAACATGAAATTTCTTGTTTTCTTAGGAATC 27920

CCAAGAGTGAACTTGACGTTCTAGTATTACTTTGAACAGTGCGGATTTGCTTGTACTTTAAAGAACAAAAGAATCCTTAG

SQL*LHFTKNVVYSHVLNINHM*LMTR

NdeI

27921 ATCACAACTGTAGCTGCATTTCACCAAGAATGTAGTTTACAGTCATGTACTCAACATCAACCATATGTAGTTGATGACCC 28000

TAGTGTTGACATCGACGTAAAGTGGTTCTTACATCAAATGTCAGTACATGAGTTGTAGTTGGTATACATCAACTACTGGG

VLFTSILNGILE*ELENQHL*LNCAW

28001 GTGTCCTATTCACTTCTATTCTAAATGGTATATTAGAGTAGGAGCTAGAAAATCAGCACCTTTAATTGAATTGTGCGTGG 28080

CACAGGATAAGTGAAGATAAGATTTACCATATAATCTCATCCTCGATCTTTTAGTCGTGGAAATTAACTTAACACGCACC

MRLVLNHPFSTSISVIIQFPVYLLQLI

ClaIEcoRVAseI

28081 ATGAGGCTGGTTCTAAATCACCCATTCAGTACATCGATATCGGTAATTATACAGTTTCCTGTTTACCTTTTACAATTAAT 28160

TACTCCGACCAAGATTTAGTGGGTAAGTCATGTAGCTATAGCCATTAATATGTCAAAGGACAAATGGAAAATGTTAATTA

ARNLNWVVL*CVVRSMKTF*SIMTFVL

BspHI

28161 TGCCAGGAACCTAAATTGGGTAGTCTTGTAGTGCGTTGTTCGTTCTATGAAGACTTTTTAGAGTATCATGACGTTCGTGT 28240

ACGGTCCTTGGATTTAACCCATCAGAACATCACGCAACAAGCAAGATACTTCTGAAAAATCTCATAGTACTGCAAGCACA

F*ISSKRTN*NV**WTPKSAKCTPHY

28241 TGTTTTAGATTTCATCTAAACGAACAAACTAAAATGTCTGATAATGGACCCCAAAATCAGCGAAATGCACCCCGCATTAC 28320

ACAAAATCTAAAGTAGATTTGCTTGTTTGATTTTACAGACTATTACCTGGGGTTTTAGTCGCTTTACGTGGGGCGTAATG

VWWTLRFNWQ*PEWRTQWGAIKTTSAP

28321 GTTTGGTGGACCCTCAGATTCAACTGGCAGTAACCAGAATGGAGAACGCAGTGGGGCGCGATCAAAACAACGTCGGCCCC 28400

CAAACCACCTGGGAGTCTAAGTTGACCGTCATTGGTCTTACCTCTTGCGTCACCCCGCGCTAGTTTTGTTGCAGCCGGGG

RFTQ*YCVLVHRSHSTWQGRP*IPSRT

XhoI

28401 AAGGTTTACCCAATAATACTGCGTCTTGGTTCACCGCTCTCACTCAACATGGCAAGGAAGACCTTAAATTCCCTCGAGGA 28480

TTCCAAATGGGTTATTATGACGCAGAACCAAGTGGCGAGAGTGAGTTGTACCGTTCCTTCTGGAATTTAAGGGAGCTCCT

RRSN*HQ*QSR*PNWLLPKSYQTNSW

EcoRI

28481 CAAGGCGTTCCAATTAACACCAATAGCAGTCCAGATGACCAAATTGGCTACTACCGAAGAGCTACCAGACGAATTCGTGG 28560

GTTCCGCAAGGTTAATTGTGGTTATCGTCAGGTCTACTGGTTTAACCGATGATGGCTTCTCGATGGTCTGCTTAAGCACC

W*R*NERSQSKMVFLLPRNWARSWTSL

BglIIAvrII

28561 TGGTGACGGTAAAATGAAAGATCTCAGTCCAAGATGGTATTTCTACTACCTAGGAACTGGGCCAGAAGCTGGACTTCCCT 28640

ACCACTGCCATTTTACTTTCTAGAGTCAGGTTCTACCATAAAGATGATGGATCCTTGACCCGGTCTTCGACCTGAAGGGA

WC*QRRHHMGCN*GSLEYTKRSHWHPQ

NdeI

28641 ATGGTGCTAACAAAGACGGCATCATATGGGTTGCAACTGAGGGAGCCTTGAATACACCAAAAGATCACATTGGCACCCGC 28720

TACCACGATTGTTTCTGCCGTAGTATACCCAACGTTGACTCCCTCGGAACTTATGTGGTTTTCTAGTGTAACCGTGGGCG

SC*QCCNRATTSSRNNIAKRLLRRRE

28721 AATCCTGCTAACAATGCTGCAATCGTGCTACAACTTCCTCAAGGAACAACATTGCCAAAAGGCTTCTACGCAGAAGGGAG 28800

TTAGGACGATTGTTACGACGTTAGCACGATGTTGAAGGAGTTCCTTGTTGTAACGGTTTTCCGAAGATGCGTCTTCCCTC

QRRQSSLFSFLIT*SQQFKKFNSRQQ*

28801 CAGAGGCGGCAGTCAAGCCTCTTCTCGTTCCTCATCACGTAGTCGCAACAGTTCAAGAAATTCAACTCCAGGCAGCAGTA 28880

GTCTCCGCCGTCAGTTCGGAGAAGAGCAAGGAGTAGTGCATCAGCGTTGTCAAGTTCTTTAAGTTGAGGTCCGTCGTCAT

GNFSC*NGWQWR*CCSCFAAA*QIEPA

28881 GGGGAACTTCTCCTGCTAGAATGGCTGGCAATGGCGGTGATGCTGCTCTTGCTTTGCTGCTGCTTGACAGATTGAACCAG 28960

CCCCTTGAAGAGGACGATCTTACCGACCGTTACCGCCACTACGACGAGAACGAAACGACGACGAACTGTCTAACTTGGTC

*EQNVW*RPTTTRPNCH*EICC*GF*

28961 CTTGAGAGCAAAATGTCTGGTAAAGGCCAACAACAACAAGGCCAAACTGTCACTAAGAAATCTGCTGCTGAGGCTTCTAA 29040

GAACTCTCGTTTTACAGACCATTTCCGGTTGTTGTTGTTCCGGTTTGACAGTGATTCTTTAGACGACGACTCCGAAGATT

EASAKTYCH*SIQCNTSFRQTWSRTNP

HindIII

29041 GAAGCCTCGGCAAAAACGTACTGCCACTAAAGCATACAATGTAACACAAGCTTTCGGCAGACGTGGTCCAGAACAAACCC 29120

CTTCGGAGCCGTTTTTGCATGACGGTGATTTCGTATGTTACATTGTGTTCGAAAGCCGTCTGCACCAGGTCTTGTTTGGG

RKFWGPGTNQTRN*LQTLAANCTICPQ

29121 AAGGAAATTTTGGGGACCAGGAACTAATCAGACAAGGAACTGATTACAAACATTGGCCGCAAATTGCACAATTTGCCCCC 29200

TTCCTTTAAAACCCCTGGTCCTTGATTAGTCTGTTCCTTGACTAATGTTTGTAACCGGCGTTTAACGTGTTAAACGGGGG

RFSVLRNVAHWHGSHTFGNVVDLHRC

AfeI

29201 AGCGCTTCAGCGTTCTTCGGAATGTCGCGCATTGGCATGGAAGTCACACCTTCGGGAACGTGGTTGACCTACACAGGTGC 29280

TCGCGAAGTCGCAAGAAGCCTTACAGCGCGTAACCGTACCTTCAGTGTGGAAGCCCTTGCACCAACTGGATGTGTCCACG

HQIG*QRSKFQRSSHFAE*AY*RIQNI

29281 CATCAAATTGGATGACAAAGATCCAAATTTCAAAGATCAAGTCATTTTGCTGAATAAGCATATTGACGCATACAAAACAT 29360

GTAGTTTAACCTACTGTTTCTAGGTTTAAAGTTTCTAGTTCAGTAAAACGACTTATTCGTATAACTGCGTATGTTTTGTA

PTNRA*KGQKEEG**NSSLTAETEETA

29361 TCCCACCAACAGAGCCTAAAAAGGACAAAAAGAAGAAGGCTGATGAAACTCAAGCCTTACCGCAGAGACAGAAGAAACAG 29440

AGGGTGGTTGTCTCGGATTTTTCCTGTTTTTCTTCTTCCGACTACTTTGAGTTCGGAATGGCGTCTCTGTCTTCTTTGTC

NCDSSSCCRFG*FLQTIATIHEQC*L

PstIMfeI

29441 CAAACTGTGACTCTTCTTCCTGCTGCAGATTTGGATGATTTCTCCAAACAATTGCAACAATCCATGAGCAGTGCTGACTC 29520

GTTTGACACTGAGAAGAAGGACGACGTCTAAACCTACTAAAGAGGTTTGTTAACGTTGTTAGGTACTCGTCACGACTGAG

NSGLNSCRPHKADGLYKRFRFSVYDI*

StuIAclI

29521 AACTCAGGCCTAAACTCATGCAGACCACACAAGGCAGATGGGCTATATAAACGTTTTCGCTTTTCCGTTTACGATATATA 29600

TTGAGTCCGGATTTGAGTACGTCTGGTGTGTTCCGTCTACCCGATATATTTGCAAAAGCGAAAAGGCAAATGCTATATAT

STLVQNEFS*LHSTSRCS*L*SHIAIF

EcoRIHpaI

29601 GTCTACTCTTGTGCAGAATGAATTCTCGTAACTACATAGCACAAGTAGATGTAGTTAACTTTAATCTCACATAGCAATCT 29680

CAGATGAGAACACGTCTTACTTAAGAGCATTGATGTATCGTGTTCATCTACATCAATTGAAATTAGAGTGTATCGTTAGA

NQCVTLGRT*KSHHIFTEATRSTIEC

PvuIBsrGI

29681 TTAATCAGTGTGTAACATTAGGGAGGACTTGAAAGAGCCACCACATTTTCACCGAGGCCACGCGGAGTACGATCGAGTGT 29760

AATTAGTCACACATTGTAATCCCTCCTGAACTTTCTCGGTGGTGTAAAAGTGGCTCCGGTGCGCCTCATGCTAGCTCACA

TVNNARESCLYGRALMCKINFSSAIPM

AseI

29761 ACAGTGAACAATGCTAGGGAGAGCTGCCTATATGGAAGAGCCCTAATGTGTAAAATTAATTTTAGTAGTGCTATCCCCAT 29840

TGTCACTTGTTACGATCCCTCTCGACGGATATACCTTCTCGGGATTACACATTTTAATTAAAATCATCACGATAGGGGTA

*F**LLRRMTKKKKKKKKKK

29841 GTGATTTTAATAGCTTCTTAGGAGAATGACAAAAAAAAAAAAAAAAAAAAAAAAAAAAAAAAA 29903

CACTAAAATTATCGAAGAATCCTCTTACTGTTTTTTTTTTTTTTTTTTTTTTTTTTTTTTTTT
