# Supplementary material for: Whole Genome Analysis and Targeted Drug Discovery Using Computational Methods and High Throughput Screening Tools for Emerged Novel Coronavirus (2019-nCoV)
Source: J Pharm Drug Res. Author manuscript; Available in PMC 2020 Jul 2. (PMC7331973)
Supplement: supplement9Taxonomy [file NIHMS1582187-supplement-supplement9Taxonomy.docx]

| Taxonomy | Number of hits | Number of Organisms | Description |
| --- | --- | --- | --- |
| [root](https://www.ncbi.nlm.nih.gov/Taxonomy/Browser/wwwtax.cgi?id=1) | [106](https://blast.ncbi.nlm.nih.gov/Blast.cgi) | 42 |  |
| **.** [Coronaviridae](https://www.ncbi.nlm.nih.gov/Taxonomy/Browser/wwwtax.cgi?id=11118) | [105](https://blast.ncbi.nlm.nih.gov/Blast.cgi) | 41 |  |
| **..** [Betacoronavirus](https://www.ncbi.nlm.nih.gov/Taxonomy/Browser/wwwtax.cgi?id=694002) | [101](https://blast.ncbi.nlm.nih.gov/Blast.cgi) | 39 |  |
| **...** [Sarbecovirus](https://www.ncbi.nlm.nih.gov/Taxonomy/Browser/wwwtax.cgi?id=2509511" \o "Show taxonomy info for Sarbecovirus (taxid 2509511)" \t "lnktx6T8E0KJ7016) | [97](https://blast.ncbi.nlm.nih.gov/Blast.cgi) | 35 |  |
| **....** [Severe acute respiratory syndrome-related coronavirus](https://www.ncbi.nlm.nih.gov/Taxonomy/Browser/wwwtax.cgi?id=694009) | [3](https://blast.ncbi.nlm.nih.gov/Blast.cgi) | 35 | [Severe acute respiratory syndrome-related coronavirus hits](https://blast.ncbi.nlm.nih.gov/Blast.cgi) |
| **.....** [Severe acute respiratory syndrome coronavirus 2](https://www.ncbi.nlm.nih.gov/Taxonomy/Browser/wwwtax.cgi?id=2697049) | [55](https://blast.ncbi.nlm.nih.gov/Blast.cgi) | 1 | [Severe acute respiratory syndrome coronavirus 2 hits](https://blast.ncbi.nlm.nih.gov/Blast.cgi) |
| **.....** [Bat SARS-like coronavirus](https://www.ncbi.nlm.nih.gov/Taxonomy/Browser/wwwtax.cgi?id=1508227) | [7](https://blast.ncbi.nlm.nih.gov/Blast.cgi) | 1 | [Bat SARS-like coronavirus hits](https://blast.ncbi.nlm.nih.gov/Blast.cgi) |
| **.....** [SARS coronavirus ZS-C](https://www.ncbi.nlm.nih.gov/Taxonomy/Browser/wwwtax.cgi?id=249088) | [1](https://blast.ncbi.nlm.nih.gov/Blast.cgi) | 1 | [SARS coronavirus ZS-C hits](https://blast.ncbi.nlm.nih.gov/Blast.cgi) |
| **.....** [SARS coronavirus ZS-B](https://www.ncbi.nlm.nih.gov/Taxonomy/Browser/wwwtax.cgi?id=249081) | [1](https://blast.ncbi.nlm.nih.gov/Blast.cgi) | 1 | [SARS coronavirus ZS-B hits](https://blast.ncbi.nlm.nih.gov/Blast.cgi) |
| **.....** [SARS coronavirus ZS-A](https://www.ncbi.nlm.nih.gov/Taxonomy/Browser/wwwtax.cgi?id=249082) | [1](https://blast.ncbi.nlm.nih.gov/Blast.cgi) | 1 | [SARS coronavirus ZS-A hits](https://blast.ncbi.nlm.nih.gov/Blast.cgi) |
| **.....** [Civet SARS CoV SZ16/2003](https://www.ncbi.nlm.nih.gov/Taxonomy/Browser/wwwtax.cgi?id=231515) | [1](https://blast.ncbi.nlm.nih.gov/Blast.cgi) | 1 | [Civet SARS CoV SZ16/2003 hits](https://blast.ncbi.nlm.nih.gov/Blast.cgi) |
| **.....** [Civet SARS CoV SZ3/2003](https://www.ncbi.nlm.nih.gov/Taxonomy/Browser/wwwtax.cgi?id=231513) | [1](https://blast.ncbi.nlm.nih.gov/Blast.cgi) | 1 | [Civet SARS CoV SZ3/2003 hits](https://blast.ncbi.nlm.nih.gov/Blast.cgi) |
| **.....** [SARS coronavirus BJ182-12](https://www.ncbi.nlm.nih.gov/Taxonomy/Browser/wwwtax.cgi?id=511429) | [1](https://blast.ncbi.nlm.nih.gov/Blast.cgi) | 1 | [SARS coronavirus BJ182-12 hits](https://blast.ncbi.nlm.nih.gov/Blast.cgi) |
| **.....** [SARS coronavirus BJ182-8](https://www.ncbi.nlm.nih.gov/Taxonomy/Browser/wwwtax.cgi?id=511431) | [1](https://blast.ncbi.nlm.nih.gov/Blast.cgi) | 1 | [SARS coronavirus BJ182-8 hits](https://blast.ncbi.nlm.nih.gov/Blast.cgi) |
| **.....** [SARS coronavirus BJ182b](https://www.ncbi.nlm.nih.gov/Taxonomy/Browser/wwwtax.cgi?id=511433) | [1](https://blast.ncbi.nlm.nih.gov/Blast.cgi) | 1 | [SARS coronavirus BJ182b hits](https://blast.ncbi.nlm.nih.gov/Blast.cgi) |
| **.....** [SARS coronavirus BJ182a](https://www.ncbi.nlm.nih.gov/Taxonomy/Browser/wwwtax.cgi?id=511432) | [1](https://blast.ncbi.nlm.nih.gov/Blast.cgi) | 1 | [SARS coronavirus BJ182a hits](https://blast.ncbi.nlm.nih.gov/Blast.cgi) |
| **.....** [BtRs-BetaCoV/YN2013](https://www.ncbi.nlm.nih.gov/Taxonomy/Browser/wwwtax.cgi?id=1503303" \o "Show taxonomy info for BtRs-BetaCoV/YN2013 (taxid 1503303)" \t "lnktx6T8E0KJ7016) | [1](https://blast.ncbi.nlm.nih.gov/Blast.cgi) | 1 | [BtRs-BetaCoV/YN2013 hits](https://blast.ncbi.nlm.nih.gov/Blast.cgi) |
| **.....** [Bat coronavirus Cp/Yunnan2011](https://www.ncbi.nlm.nih.gov/Taxonomy/Browser/wwwtax.cgi?id=1283333) | [1](https://blast.ncbi.nlm.nih.gov/Blast.cgi) | 1 | [Bat coronavirus Cp/Yunnan2011 hits](https://blast.ncbi.nlm.nih.gov/Blast.cgi) |
| **.....** [BtRs-BetaCoV/HuB2013](https://www.ncbi.nlm.nih.gov/Taxonomy/Browser/wwwtax.cgi?id=1503302" \o "Show taxonomy info for BtRs-BetaCoV/HuB2013 (taxid 1503302)" \t "lnktx6T8E0KJ7016) | [1](https://blast.ncbi.nlm.nih.gov/Blast.cgi) | 1 | [BtRs-BetaCoV/HuB2013 hits](https://blast.ncbi.nlm.nih.gov/Blast.cgi) |
| **.....** [Bat CoV 279/2005](https://www.ncbi.nlm.nih.gov/Taxonomy/Browser/wwwtax.cgi?id=389167) | [1](https://blast.ncbi.nlm.nih.gov/Blast.cgi) | 1 | [Bat CoV 279/2005 hits](https://blast.ncbi.nlm.nih.gov/Blast.cgi) |
| **.....** [Bat coronavirus Rp/Shaanxi2011](https://www.ncbi.nlm.nih.gov/Taxonomy/Browser/wwwtax.cgi?id=1283332) | [1](https://blast.ncbi.nlm.nih.gov/Blast.cgi) | 1 | [Bat coronavirus Rp/Shaanxi2011 hits](https://blast.ncbi.nlm.nih.gov/Blast.cgi) |
| **.....** [BtRf-BetaCoV/SX2013](https://www.ncbi.nlm.nih.gov/Taxonomy/Browser/wwwtax.cgi?id=1503300" \o "Show taxonomy info for BtRf-BetaCoV/SX2013 (taxid 1503300)" \t "lnktx6T8E0KJ7016) | [1](https://blast.ncbi.nlm.nih.gov/Blast.cgi) | 1 | [BtRf-BetaCoV/SX2013 hits](https://blast.ncbi.nlm.nih.gov/Blast.cgi) |
| **.....** [BtRf-BetaCoV/HeB2013](https://www.ncbi.nlm.nih.gov/Taxonomy/Browser/wwwtax.cgi?id=1503296" \o "Show taxonomy info for BtRf-BetaCoV/HeB2013 (taxid 1503296)" \t "lnktx6T8E0KJ7016) | [1](https://blast.ncbi.nlm.nih.gov/Blast.cgi) | 1 | [BtRf-BetaCoV/HeB2013 hits](https://blast.ncbi.nlm.nih.gov/Blast.cgi) |
| **.....** [BtRf-BetaCoV/JL2012](https://www.ncbi.nlm.nih.gov/Taxonomy/Browser/wwwtax.cgi?id=1503299" \o "Show taxonomy info for BtRf-BetaCoV/JL2012 (taxid 1503299)" \t "lnktx6T8E0KJ7016) | [1](https://blast.ncbi.nlm.nih.gov/Blast.cgi) | 1 | [BtRf-BetaCoV/JL2012 hits](https://blast.ncbi.nlm.nih.gov/Blast.cgi) |
| **.....** [Bat SARS coronavirus HKU3](https://www.ncbi.nlm.nih.gov/Taxonomy/Browser/wwwtax.cgi?id=442736) | [13](https://blast.ncbi.nlm.nih.gov/Blast.cgi) | 13 |  |
| **......** [Bat SARS coronavirus HKU3-7](https://www.ncbi.nlm.nih.gov/Taxonomy/Browser/wwwtax.cgi?id=742004) | [1](https://blast.ncbi.nlm.nih.gov/Blast.cgi) | 1 | [Bat SARS coronavirus HKU3-7 hits](https://blast.ncbi.nlm.nih.gov/Blast.cgi) |
| **......** [Bat SARS coronavirus HKU3-8](https://www.ncbi.nlm.nih.gov/Taxonomy/Browser/wwwtax.cgi?id=742005) | [1](https://blast.ncbi.nlm.nih.gov/Blast.cgi) | 1 | [Bat SARS coronavirus HKU3-8 hits](https://blast.ncbi.nlm.nih.gov/Blast.cgi) |
| **......** [Bat SARS coronavirus HKU3-12](https://www.ncbi.nlm.nih.gov/Taxonomy/Browser/wwwtax.cgi?id=741999) | [1](https://blast.ncbi.nlm.nih.gov/Blast.cgi) | 1 | [Bat SARS coronavirus HKU3-12 hits](https://blast.ncbi.nlm.nih.gov/Blast.cgi) |
| **......** [Bat SARS coronavirus HKU3-2](https://www.ncbi.nlm.nih.gov/Taxonomy/Browser/wwwtax.cgi?id=338605) | [1](https://blast.ncbi.nlm.nih.gov/Blast.cgi) | 1 | [Bat SARS coronavirus HKU3-2 hits](https://blast.ncbi.nlm.nih.gov/Blast.cgi) |
| **......** [Bat SARS coronavirus HKU3-5](https://www.ncbi.nlm.nih.gov/Taxonomy/Browser/wwwtax.cgi?id=742002) | [1](https://blast.ncbi.nlm.nih.gov/Blast.cgi) | 1 | [Bat SARS coronavirus HKU3-5 hits](https://blast.ncbi.nlm.nih.gov/Blast.cgi) |
| **......** [Bat SARS coronavirus HKU3-4](https://www.ncbi.nlm.nih.gov/Taxonomy/Browser/wwwtax.cgi?id=742001) | [1](https://blast.ncbi.nlm.nih.gov/Blast.cgi) | 1 | [Bat SARS coronavirus HKU3-4 hits](https://blast.ncbi.nlm.nih.gov/Blast.cgi) |
| **......** [Bat SARS coronavirus HKU3-11](https://www.ncbi.nlm.nih.gov/Taxonomy/Browser/wwwtax.cgi?id=741998) | [1](https://blast.ncbi.nlm.nih.gov/Blast.cgi) | 1 | [Bat SARS coronavirus HKU3-11 hits](https://blast.ncbi.nlm.nih.gov/Blast.cgi) |
| **......** [Bat SARS coronavirus HKU3-1](https://www.ncbi.nlm.nih.gov/Taxonomy/Browser/wwwtax.cgi?id=333387) | [1](https://blast.ncbi.nlm.nih.gov/Blast.cgi) | 1 | [Bat SARS coronavirus HKU3-1 hits](https://blast.ncbi.nlm.nih.gov/Blast.cgi) |
| **......** [Bat SARS coronavirus HKU3-3](https://www.ncbi.nlm.nih.gov/Taxonomy/Browser/wwwtax.cgi?id=338606) | [1](https://blast.ncbi.nlm.nih.gov/Blast.cgi) | 1 | [Bat SARS coronavirus HKU3-3 hits](https://blast.ncbi.nlm.nih.gov/Blast.cgi) |
| **......** [Bat SARS coronavirus HKU3-13](https://www.ncbi.nlm.nih.gov/Taxonomy/Browser/wwwtax.cgi?id=742000) | [1](https://blast.ncbi.nlm.nih.gov/Blast.cgi) | 1 | [Bat SARS coronavirus HKU3-13 hits](https://blast.ncbi.nlm.nih.gov/Blast.cgi) |
| **......** [Bat SARS coronavirus HKU3-6](https://www.ncbi.nlm.nih.gov/Taxonomy/Browser/wwwtax.cgi?id=742003) | [1](https://blast.ncbi.nlm.nih.gov/Blast.cgi) | 1 | [Bat SARS coronavirus HKU3-6 hits](https://blast.ncbi.nlm.nih.gov/Blast.cgi) |
| **......** [Bat SARS coronavirus HKU3-10](https://www.ncbi.nlm.nih.gov/Taxonomy/Browser/wwwtax.cgi?id=741997) | [1](https://blast.ncbi.nlm.nih.gov/Blast.cgi) | 1 | [Bat SARS coronavirus HKU3-10 hits](https://blast.ncbi.nlm.nih.gov/Blast.cgi) |
| **......** [Bat SARS coronavirus HKU3-9](https://www.ncbi.nlm.nih.gov/Taxonomy/Browser/wwwtax.cgi?id=742006) | [1](https://blast.ncbi.nlm.nih.gov/Blast.cgi) | 1 | [Bat SARS coronavirus HKU3-9 hits](https://blast.ncbi.nlm.nih.gov/Blast.cgi) |
| **.....** [Bat SARS CoV Rf1/2004](https://www.ncbi.nlm.nih.gov/Taxonomy/Browser/wwwtax.cgi?id=347537) | [1](https://blast.ncbi.nlm.nih.gov/Blast.cgi) | 2 | [Bat SARS CoV Rf1/2004 hits](https://blast.ncbi.nlm.nih.gov/Blast.cgi) |
| **......** [Bat CoV 273/2005](https://www.ncbi.nlm.nih.gov/Taxonomy/Browser/wwwtax.cgi?id=389166) | [1](https://blast.ncbi.nlm.nih.gov/Blast.cgi) | 1 | [Bat CoV 273/2005 hits](https://blast.ncbi.nlm.nih.gov/Blast.cgi) |
| **...** [unclassified Betacoronavirus](https://www.ncbi.nlm.nih.gov/Taxonomy/Browser/wwwtax.cgi?id=696098) | [4](https://blast.ncbi.nlm.nih.gov/Blast.cgi) | 4 |  |
| **....** [Coronavirus BtRs-BetaCoV/YN2018B](https://www.ncbi.nlm.nih.gov/Taxonomy/Browser/wwwtax.cgi?id=2591235) | [1](https://blast.ncbi.nlm.nih.gov/Blast.cgi) | 1 | [Coronavirus BtRs-BetaCoV/YN2018B hits](https://blast.ncbi.nlm.nih.gov/Blast.cgi) |
| **....** [Coronavirus BtRs-BetaCoV/YN2018C](https://www.ncbi.nlm.nih.gov/Taxonomy/Browser/wwwtax.cgi?id=2591236) | [1](https://blast.ncbi.nlm.nih.gov/Blast.cgi) | 1 | [Coronavirus BtRs-BetaCoV/YN2018C hits](https://blast.ncbi.nlm.nih.gov/Blast.cgi) |
| **....** [Coronavirus BtRs-BetaCoV/YN2018A](https://www.ncbi.nlm.nih.gov/Taxonomy/Browser/wwwtax.cgi?id=2591234) | [1](https://blast.ncbi.nlm.nih.gov/Blast.cgi) | 1 | [Coronavirus BtRs-BetaCoV/YN2018A hits](https://blast.ncbi.nlm.nih.gov/Blast.cgi) |
| **....** [Coronavirus BtRl-BetaCoV/SC2018](https://www.ncbi.nlm.nih.gov/Taxonomy/Browser/wwwtax.cgi?id=2591233) | [1](https://blast.ncbi.nlm.nih.gov/Blast.cgi) | 1 | [Coronavirus BtRl-BetaCoV/SC2018 hits](https://blast.ncbi.nlm.nih.gov/Blast.cgi) |
| **..** [Bat coronavirus RaTG13](https://www.ncbi.nlm.nih.gov/Taxonomy/Browser/wwwtax.cgi?id=2709072) | [1](https://blast.ncbi.nlm.nih.gov/Blast.cgi) | 1 | [Bat coronavirus RaTG13 hits](https://blast.ncbi.nlm.nih.gov/Blast.cgi) |
| **..** [Bat coronavirus](https://www.ncbi.nlm.nih.gov/Taxonomy/Browser/wwwtax.cgi?id=1508220) | [3](https://blast.ncbi.nlm.nih.gov/Blast.cgi) | 1 | [Bat coronavirus hits](https://blast.ncbi.nlm.nih.gov/Blast.cgi) |
| **.** [recombinant coronavirus](https://www.ncbi.nlm.nih.gov/Taxonomy/Browser/wwwtax.cgi?id=575864) | [1](https://blast.ncbi.nlm.nih.gov/Blast.cgi) | 1 | [recombinant coronavirus hits](https://blast.ncbi.nlm.nih.gov/Blast.cgi) |
