# Supplementary material for: Whole Genome Analysis and Targeted Drug Discovery Using Computational Methods and High Throughput Screening Tools for Emerged Novel Coronavirus (2019-nCoV)
Source: J Pharm Drug Res. Author manuscript; Available in PMC 2020 Jul 2. (PMC7331973)
Supplement: supplement2Factsheet_SmartBLAST [file NIHMS1582187-supplement-supplement2Factsheet_SmartBLAST.pdf]

SmartBLAST: A Tool For Rapid Protein Identification

https://blast.ncbi.nlm.nih.gov/smartblast/

Quickly identifying close relatives of a protein query and evaluating their phylogenetic relationship

National Center for Biotechnology Information • National Library of Medicine • National Institutes of Health • Department of Health and Human Services

Introduction

SmartBLAST is a service that quickly finds closest relatives to a protein query and evaluates the phylogentic relationship among the query and matched sequences. It does so by finding up to three best matches from the non-redundant protein database (nr) along with the two best matches from the complete proteomes of well-studied reference species (landmark dataset). If possible, the two matches from reference species will be from different organisms. In addition, SmartBLAST presents Conserved Domain Database matches to the query. A multiple sequence alignment based phylogenetic tree for the matched sequences is also part of the results presentation. Note that SmartBLAST is under active development and may change with little notice.

Workflow Behind the Service

SmartBLAST uses a combination of BLAST and a multiple sequence alignment algorithm. SmartBLAST searches the query against the nr protein database with an optimized version of BLAST targeted to find closely related sequences. It also simultaneously searches the landmark dataset (http://bit.ly/2aOVhPO, see “Search sets”) with BLASTP. Finally, it performs a multiple sequence alignment on the six sequences (the query plus up to five matched subject sequences) using the COBALT multiple sequence alignment tool. The multiple sequence alignment and BLASTP serve different but complementary roles: BLASTP identifies database sequences similar to the query and calculates only pairwise similarities between the query and individual database sequences. The COBALT’s multiple alignment compares all sequences to each other and produces an optimal alignment between all sequences, which is ideal for the presentation of a phylogenetic tree and shows the relationship among a set of sequences. The optimization of the procedure combined with the reduced landmark dataset make the SmartBLAST search extremely fast and ideal for initial screening of unknown protein sequences.

Access to the SmartBLAST Search Page

The BLAST homepage provides a convenient link to SmartBLAST (**A**) in the “Specialized searches” section. The actual search page has a very simple interface (**B**). Paste in a query protein sequence in a supported format, such as FASTA (**C**), in the search box and press “Submit” to initiate a search. Note that SmartBLAST accepts only a single protein sequence as input and will reject input that consists of multiple protein sequences or nucleotide sequence (**D**).

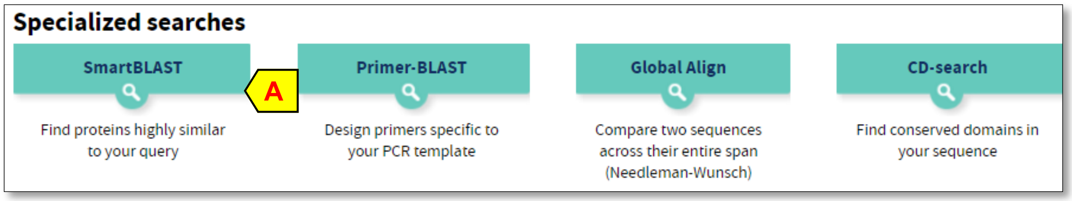

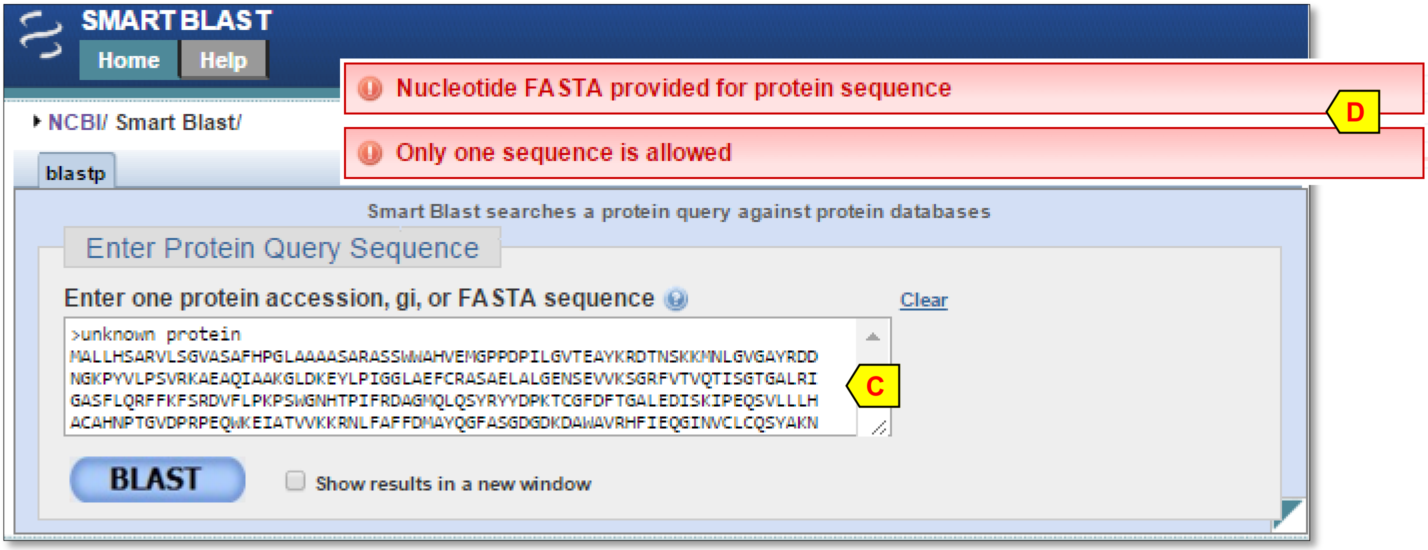

 Contact: blast-help@ncbi.nlm.nih.gov

The Results Page

SmartBLAST results contain three sections: Summary, Descriptions, and Alignments. The example below (**A**) has the Descriptions and Alignments sections closed (**B**) to emphasize the graphically enriched Summary.

Summary

The Summary presents only the top five matches from different organisms. This succinct set of best hits from a representative group of organisms allows SmartBLAST to show the relationships among the sequence, in the form of a phylogenetic tree (**C**), and the alignment coverage in schematic diagram form (**D**). This section also displays the sequence titles (**E**) and conserved domain(s) identified (**F**). For input in FASTA format, SmartBLAST will map it to existing proteins in the database. A successful mapping enables SmartBLAST to get a descriptive title from the RefSeq entry and assign taxonomic identification (**G**, as in this example). This section is highly interactive and it contains embedded popups in the titles, a schematic diagram depiction of alignments and CDD matches, as well as a link to the Legend.

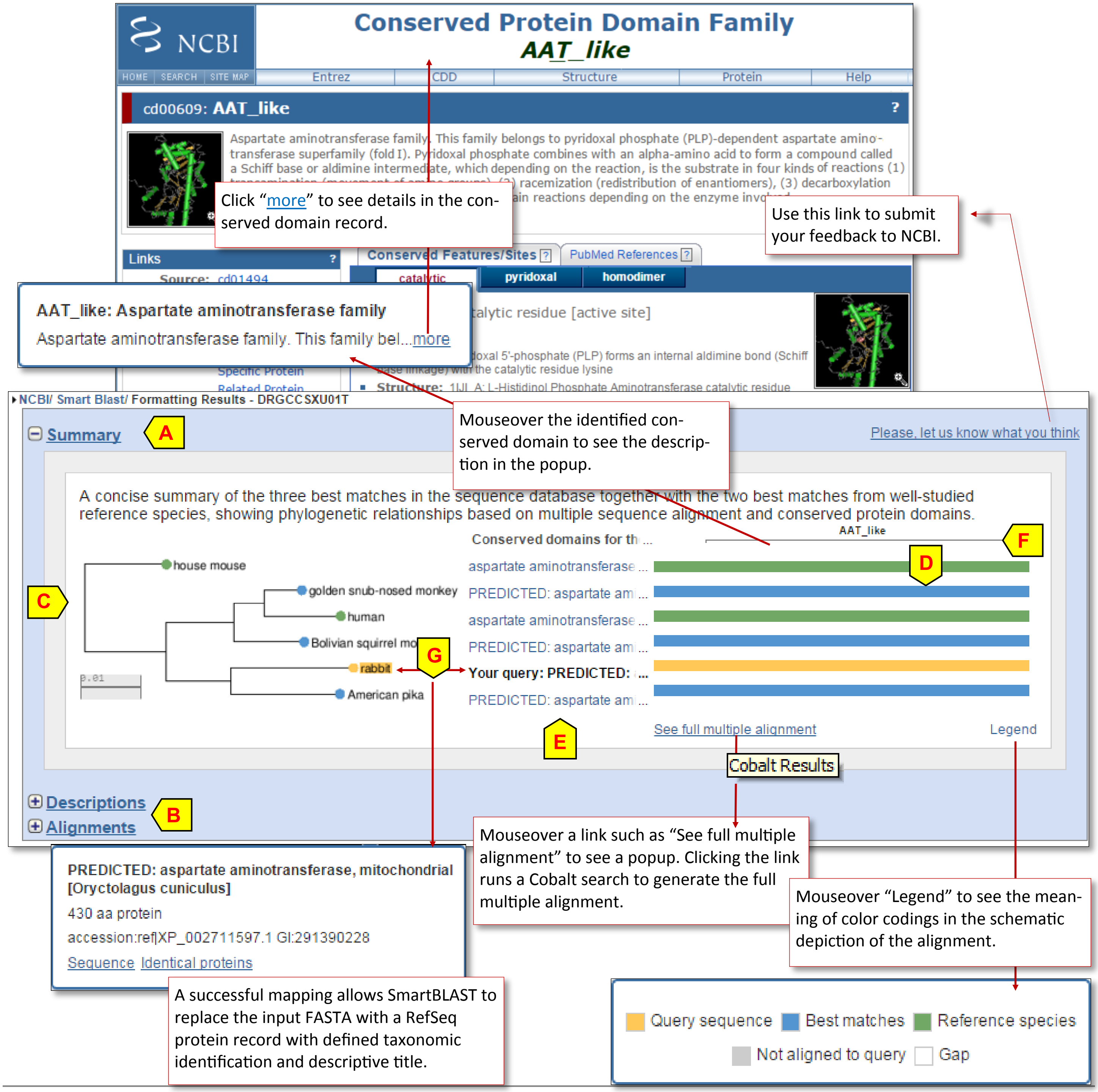

The Result Page (cont.)

Descriptions

This section lists the matches found by SmartBLAST in two separate tables. The “Best hits” table (**A**) contains only the five database matches displayed in the Summary section. The checkbox color (**B**) corresponds to the alignment cartoon to indicate the source of the hit (from the landmark dataset or NR). The “Additional BLAST Hits” table (**C**, shown in part) lists additional hits found in NR and the reference dataset. It supplements the “Best hits” set by expanding the taxonomic coverage and showing additional hits from the same organism that could represent isoforms, variants, or paralogs.

The tables are interactive with configurable columns and have links to specific sections of the Alignments as well as links to actual protein records.

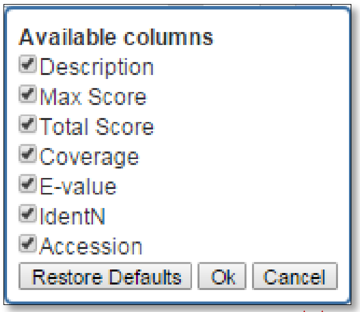

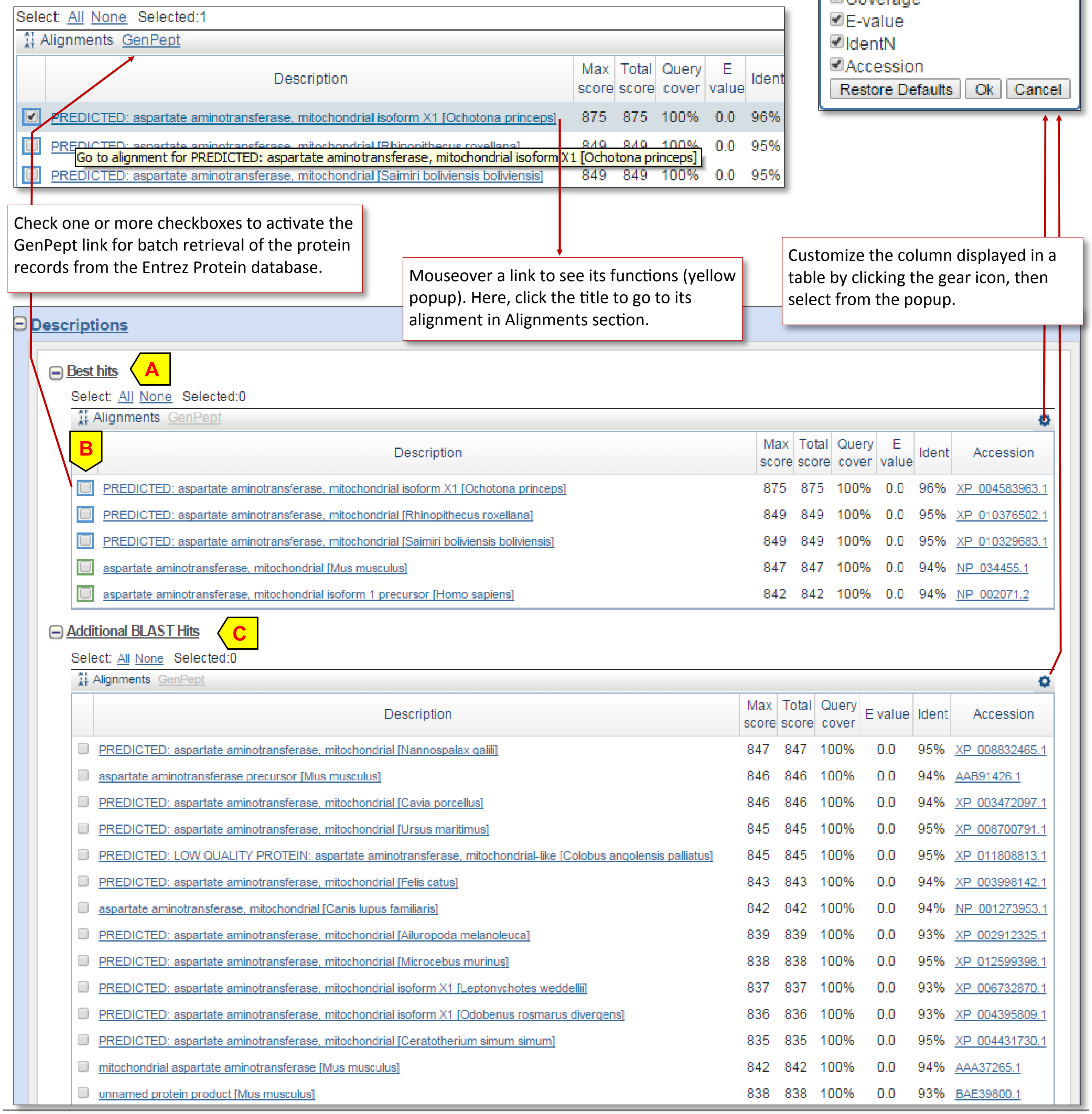

The Results Page (cont.)

Alignments

This section (**A**) provides the detailed alignments between the query and matched database sequences. For simplicity, we only display two, one is the top match (**B**) and the second is an alignment to a less closely related sequence (**C**). The horizontal lines (**D**) separate alignments from different database sequences and arrows (**E**) facilitate the navigation among different sections/subsections. The table (**F**) just above the alignment sums up the statistics for that alignment. Links under Related Information (**G**) provide additional information available for the matched database sequence from other NCBI resources.

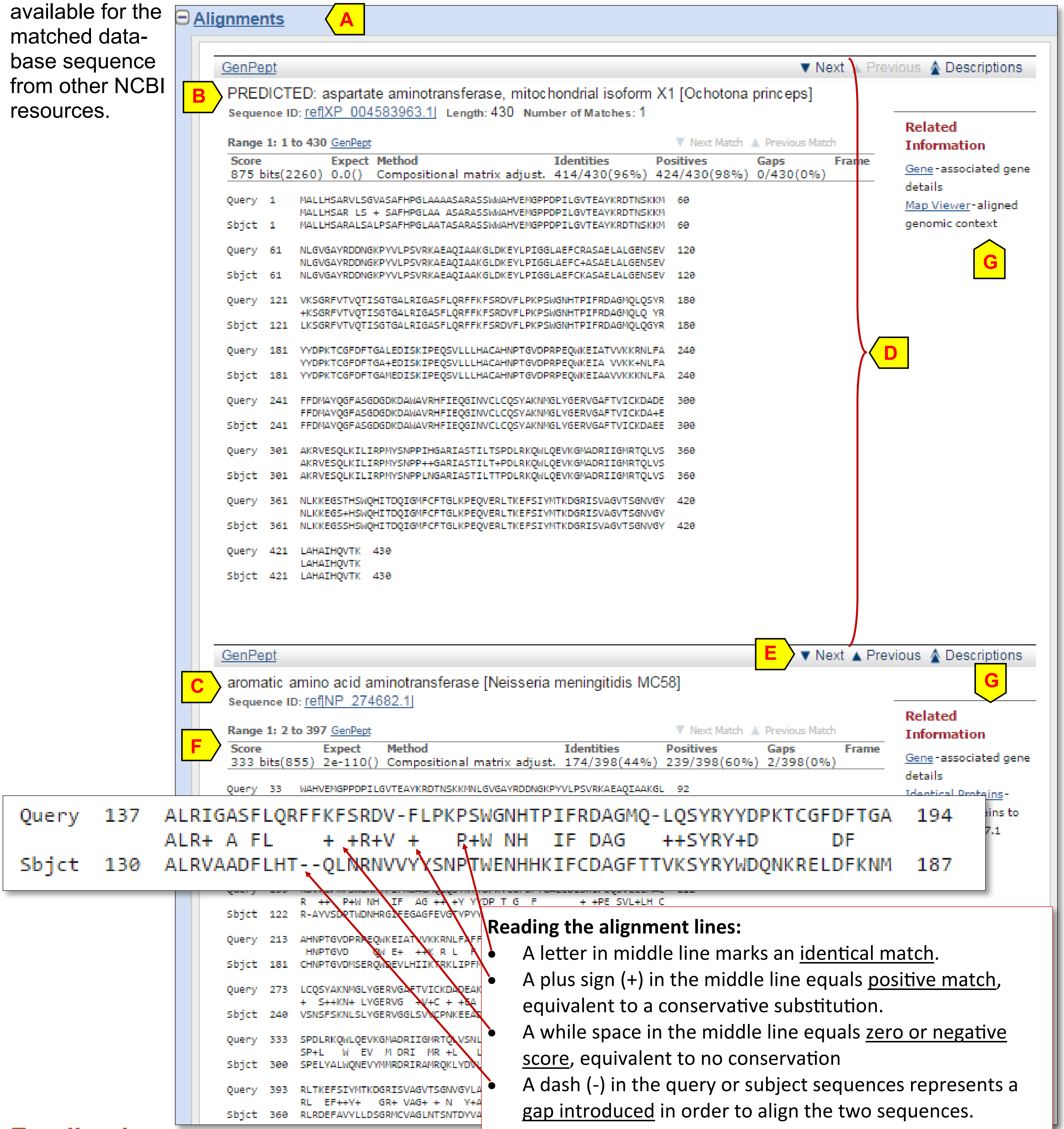

Reading the alignment lines:

- A letter in middle line marks an identical match.
- A plus sign (+) in the middle line equals positive match, equivalent to a conservative substitution.
- A while space in the middle line equals zero or negative score, equivalent to no conservation
- A dash (-) in the query or subject sequences represents a gap introduced in order to align the two sequences.

Feedback

Please submit your comments and feedback to blast-help@ncbi.nlm.nih.gov
