# Supplementary material for: Whole Genome Analysis and Targeted Drug Discovery Using Computational Methods and High Throughput Screening Tools for Emerged Novel Coronavirus (2019-nCoV)
Source: J Pharm Drug Res. Author manuscript; Available in PMC 2020 Jul 2. (PMC7331973)
Supplement: supplement6Smart BLAST orf whole genome [file NIHMS1582187-supplement-supplement6Smart_BLAST_orf_whole_genome.pdf]

- NCBI Home
- Sign in to NCBI
- Skip to Main Content
- Skip to Navigation
- About NCBI Accesskeys

U.S. National Library of Medicine

NCBI National Center for Biotechnology Information

-
- My NCBI
- Sign in to NCBI
- Register
- Sign Out

SMARTBLAST » Formatting Results - 6V3TBM71011

- Home
- Help

-

Report description

Summary [?]

Query: unnamed protein product Identical to: ORF16:266:13483 Query length: 4405 aa
DOMAINS: Papain like viral protease and 12 other(s)
Peptidase_C30
Viral_protease
nsp8

SUD-
M
NSP10
Nsp1
nsp9
Corona_NSP4_C
nsp7
Nsp3_PL2pro
Macro
DUF3655
NAR

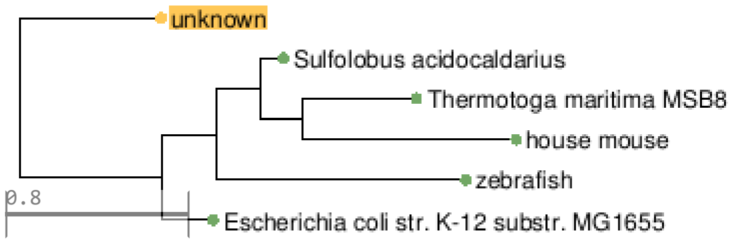

Your query: unnamed protein product
ADP-ribose-binding protein
recombination factor protein RarA
protein mono-ADP-ribosyltransferase PARP9 isoform X1
poly ymerase 14 isoform X3 [Danio rerio]
2'-O-acetyl-ADP-ribose deacetylase, regulator of RNase III activity
DOMAINS: and other(s) domains ...

unnamed protein product
4405 aa protein
ID:ORF16:266:13483

About the database
See full multiple alignment Legend

Descriptions

, Reading indexes 1-2, displaying indexes 1-2, Reading indexes 3-7, displaying indexes 3-7
Load next setPrevious Match

Best hits

Show all columns of the table presenting best hits producing significant alignments Select:AllNone Selected:0
Alignments Download GenPept Graphics Distance tree of results Multiple alignment Show/hide columns of the table presenting best hits

Best hits producing significant alignments

Select for downloading or viewing reports	Description	Max score	Total score	Query cover	E value	Ident	Accession
1 Select seq ref|WP_011278100.1|	ADP-ribose-binding protein [Sulfolobus acidocaldarius]	73.2	73.2	2%	1e-12	36.29%	WP_011278100.1
2 Select seq ref|NP_228318.1|	recombination factor protein RarA [Thermotoga maritima MSB8]	76.3	76.3	2%	6e-12	39.84%	NP_228318.1
3 Select seq ref|XP_021327929.1|	poly [ADP-ribose] polymerase 14 isoform X3 [Danio rerio]	63.5	63.5	3%	7e-08	32.26%	XP_021327929.1
4 Select seq ref|XP_006522818.1|	protein mono-ADP-ribosyltransferase PARP9 isoform X1 [Mus musculus]	62.4	62.4	2%	1e-07	33.80%	XP_006522818.1

Select for downloading or viewing reports	Description	Max score	Total score	Query cover	E value	Ident	Accession
5 Select seq ref|NP_415563.1|	2'-O-acetyl-ADP-ribose deacetylase, regulator of RNase III activity [Escherichia coli str. K-12 substr. MG1655]	55.8	55.8	3%	9e-07	29.07%	NP_415563.1

Additional BLAST Hits

Show all columns of the table presenting additional hits Select:AllNone Selected:0
Alignments GenPept Graphics Distance tree of results Multiple alignment Show/hide columns of the table presenting sequences with E-value WORSE than threshold

Additional Hits

Select for downloading or viewing reports	Description	Max score	Total score	Query cover	E value	Ident	Accession
6 Select seq gb|QIA20043.1|	orf1ab polyprotein [Severe acute respiratory syndrome coronavirus 2]	9173	9173	100%	0.0	99.98%	QIA20043.1
7 Select seq gb|QHU79172.1|	orf1a polyprotein [Severe acute respiratory syndrome coronavirus 2]	9141	9141	100%	0.0	99.73%	QHU79172.1
8 Select seq gb|QHQ71972.1|	orf1ab polyprotein [Severe acute respiratory syndrome coronavirus 2]	9137	9137	99%	0.0	100.00%	QHQ71972.1

Select for downloading or viewing reports	Description	Max score	Total score	Query cover	E value	Ident	Accession
9 Select seq gb|QHR63249.1|	orf1ab polyprotein [Severe acute respiratory syndrome coronavirus 2]	9137	9137	99%	0.0	100.00%	QHR63249.1
10 Select seq gb|QHZ00388.1|	orf1ab polyprotein [Severe acute respiratory syndrome coronavirus 2]	9137	9137	99%	0.0	100.00%	QHZ00388.1
11 Select seq gb|QID21067.1|	orf1ab polyprotein [Severe acute respiratory syndrome coronavirus 2]	9137	9137	99%	0.0	100.00%	QID21067.1
12 Select seq ref|YP_009724389.1|	orf1ab polyprotein [Severe acute respiratory syndrome coronavirus 2]	9137	9137	99%	0.0	100.00%	YP_009724389.1
13 Select seq gb|QHQ71962.1|	orf1ab polyprotein [Severe acute respiratory syndrome coronavirus 2]	9135	9135	99%	0.0	99.98%	QHQ71962.1
14 Select seq gb|QHQ82463.1|	orf1ab polyprotein [Severe acute respiratory syndrome coronavirus 2]	9135	9135	99%	0.0	99.98%	QHQ82463.1
15 Select seq gb|QHW06058.1|	orf1ab polyprotein [Severe acute respiratory syndrome coronavirus 2]	9135	9135	99%	0.0	99.98%	QHW06058.1

Select for downloading or viewing reports	Description	Max score	Total score	Query cover	E value	Ident	Accession
16 Select seq gb|QHZ00378.1|	orf1ab polyprotein [Severe acute respiratory syndrome coronavirus 2]	9135	9135	99%	0.0	99.98%	QHZ00378.1
17 Select seq gb|QIA98605.1|	orf1ab polyprotein [Severe acute respiratory syndrome coronavirus 2]	9135	9135	99%	0.0	99.98%	QIA98605.1
18 Select seq gb|QID98793.1|	orf1ab polyprotein [Severe acute respiratory syndrome coronavirus 2]	9135	9135	99%	0.0	99.98%	QID98793.1
19 Select seq gb|QIE07470.1|	orf1ab polyprotein [Severe acute respiratory syndrome-related coronavirus]	9135	9135	99%	0.0	99.98%	QIE07470.1
20 Select seq gb|QHU36843.1|	orf1ab polyprotein [Severe acute respiratory syndrome coronavirus 2]	9134	9134	99%	0.0	99.98%	QHU36843.1
21 Select seq gb|QHW06038.1|	orf1ab polyprotein [Severe acute respiratory syndrome coronavirus 2]	9134	9134	99%	0.0	99.95%	QHW06038.1
22 Select seq gb|QHN73809.1|	orf1ab polyprotein [Severe acute respiratory syndrome coronavirus 2]	9134	9134	99%	0.0	99.98%	QHN73809.1

Select for downloading or viewing reports	Description	Max score	Total score	Query cover	E value	Ident	Accession
23 Select seq gb|QIE07460.1|	orf1ab polyprotein [Severe acute respiratory syndrome coronavirus 2]	9134	9134	99%	0.0	99.98%	QIE07460.1
24 Select seq gb|QHU36863.1|	orf1ab polyprotein [Severe acute respiratory syndrome coronavirus 2]	9133	9133	99%	0.0	99.98%	QHU36863.1
25 Select seq gb|QHR63269.1|	orf1ab polyprotein [Severe acute respiratory syndrome coronavirus 2]	9133	9133	99%	0.0	99.98%	QHR63269.1
26 Select seq gb|QHU36823.1|	orf1ab polyprotein [Severe acute respiratory syndrome coronavirus 2]	9132	9132	99%	0.0	99.95%	QHU36823.1
27 Select seq gb|QIC53203.1|	orf1ab polyprotein [Severe acute respiratory syndrome coronavirus 2]	9132	9132	99%	0.0	99.95%	QIC53203.1
28 Select seq gb|QHR63289.1|	orf1ab polyprotein [Severe acute respiratory syndrome coronavirus 2]	9131	9131	99%	0.0	99.95%	QHR63289.1
29 Select seq gb|QHZ87581.1|	orf1ab polyprotein [Severe acute respiratory syndrome coronavirus 2]	9131	9131	99%	0.0	99.95%	QHZ87581.1

Select for downloading or viewing reports	Description	Max score	Total score	Query cover	E value	Ident	Accession
30 Select seq gb|QHO62876.1|	orf1ab polyprotein [Severe acute respiratory syndrome coronavirus 2]	9130	9130	99%	0.0	99.95%	QHO62876.1
31 Select seq gb|QHZ00398.1|	orf1ab polyprotein [Severe acute respiratory syndrome coronavirus 2]	9130	9130	99%	0.0	99.95%	QHZ00398.1
32 Select seq gb|QIE07480.1|	orf1ab polyprotein [Severe acute respiratory syndrome-related coronavirus]	9129	9129	99%	0.0	99.93%	QIE07480.1
33 Select seq gb|QHZ87591.1|	orf1ab polyprotein [Severe acute respiratory syndrome coronavirus 2]	9109	9109	99%	0.0	99.82%	QHZ87591.1
34 Select seq gb|QHU79171.1|	orf1ab polyprotein [Severe acute respiratory syndrome coronavirus 2]	9102	9102	99%	0.0	99.73%	QHU79171.1
35 Select seq gb|QHR63299.1|	orf1ab polyprotein [Bat coronavirus RaTG13]	8968	8968	99%	0.0	98.05%	QHR63299.1
36 Select seq gb|AVP78030.1|	non-structural polyprotein 1ab [Bat SARS-like coronavirus]	8683	8683	99%	0.0	95.39%	AVP78030.1

Select for downloading or viewing reports	Description	Max score	Total score	Query cover	E value	Ident	Accession
37 Select seq gb|AVP78041.1|	non-structural polyprotein 1ab [Bat SARS-like coronavirus]	8646	8646	99%	0.0	94.94%	AVP78041.1
38 Select seq gb|AID16715.1|	orf1ab polyprotein [Bat SARS-like coronavirus]	7598	7598	99%	0.0	82.32%	AID16715.1
39 Select seq gb|ATO98131.1|	non-structural polyprotein 1a [Bat SARS-like coronavirus]	7527	7527	100%	0.0	80.48%	ATO98131.1
40 Select seq gb|AHX37556.1|	protein 1a [Rhinolophus affinis coronavirus]	7525	7525	100%	0.0	80.26%	AHX37556.1
41 Select seq gb|ATO98192.1|	non-structural polyprotein 1a [Bat SARS-like coronavirus]	7518	7518	100%	0.0	80.41%	ATO98192.1
42 Select seq gb|ATO98156.1|	non-structural polyprotein 1a [Bat SARS-like coronavirus]	7517	7517	100%	0.0	80.54%	ATO98156.1
43 Select seq gb|AGZ48804.1|	non-structural polyprotein 1a [Bat SARS-like coronavirus RsSHC014]	7515	7515	100%	0.0	80.48%	AGZ48804.1

Select for downloading or viewing reports	Description	Max score	Total score	Query cover	E value	Ident	Accession
44 Select seq gb|ATO98107.1|	non-structural polyprotein 1a [Bat SARS-like coronavirus]	7513	7513	100%	0.0	80.48%	ATO98107.1
45 Select seq gb|AAP13575.1|	orf1a polyprotein [SARS coronavirus CUHK-W1]	7511	7511	100%	0.0	80.35%	AAP13575.1
46 Select seq gb|AAY60778.1|	orf1a polyprotein [SARS coronavirus BJ162]	7511	7511	100%	0.0	80.35%	AAY60778.1
47 Select seq gb|AAP33695.1|	polyprotein 1a [SARS coronavirus Frankfurt 1]	7509	7509	100%	0.0	80.33%	AAP33695.1
48 Select seq gb|AAR87599.1|	orf1a polyprotein [SARS coronavirus TW9]	7509	7509	100%	0.0	80.33%	AAR87599.1
49 Select seq ref|NP_828850.1|	orf1a polyprotein (pp1a) [Severe acute respiratory syndrome-related coronavirus]	7509	7509	100%	0.0	80.33%	NP_828850.1
50 Select seq gb|AAR23244.1|	orf1a polyprotein [SARS coronavirus Sino1-11]	7508	7508	100%	0.0	80.30%	AAR23244.1

Select for downloading or viewing reports	Description	Max score	Total score	Query cover	E value	Ident	Accession
51 Select seq gb|AAP30029.1|	orf1a polyprotein [SARS coronavirus BJ01]	7508	7508	100%	0.0	80.30%	AAP30029.1
52 Select seq gb|AAP13439.1|	nonstructural polyprotein pp1a [SARS coronavirus Urbani]	7507	7507	100%	0.0	80.30%	AAP13439.1
53 Select seq gb|AAR87533.1|	orf1a polyprotein [SARS coronavirus TW3]	7507	7507	100%	0.0	80.30%	AAR87533.1
54 Select seq gb|AAR87544.1|	orf1a polyprotein [SARS coronavirus TW4]	7507	7507	100%	0.0	80.30%	AAR87544.1
55 Select seq gb|ADC35509.1|	orf1a polyprotein [SARS coronavirus HKU-39849]	7507	7507	100%	0.0	80.30%	ADC35509.1
56 Select seq gb|AFR58684.1|	polyprotein orf1a [Severe acute respiratory syndrome-related coronavirus]	7507	7507	100%	0.0	80.30%	AFR58684.1
57 Select seq gb|AFR58698.1|	polyprotein orf1a [Severe acute respiratory syndrome-related coronavirus]	7507	7507	100%	0.0	80.30%	AFR58698.1

Select for downloading or viewing reports	Description	Max score	Total score	Query cover	E value	Ident	Accession
58 Select seq gb|AAR23252.1|	orf1a polyprotein [SARS coronavirus Sino3-11]	7506	7506	100%	0.0	80.30%	AAR23252.1
59 Select seq gb|ACV88184.1|	replicase 1AB polyprotein [Severe acute respiratory syndrome-related coronavirus]	7506	7506	100%	0.0	80.28%	ACV88184.1
60 Select seq gb|ACZ72208.1|	orf1a polyprotein [SARS coronavirus MA15]	7506	7506	100%	0.0	80.26%	ACZ72208.1
61 Select seq gb|AEA10531.1|	polyprotein orf1a [SARS coronavirus MA15 ExoN1]	7506	7506	100%	0.0	80.24%	AEA10531.1
62 Select seq gb|AEA10726.1|	polyprotein orf1a [SARS coronavirus MA15 ExoN1]	7506	7506	100%	0.0	80.26%	AEA10726.1
63 Select seq gb|AAT76148.1|	Orf1a polyprotein [SARS coronavirus TJF]	7505	7505	100%	0.0	80.28%	AAT76148.1
64 Select seq gb|AAR16181.1|	orf1a polyprotein [SARS coronavirus ZJ01]	7505	7505	100%	0.0	80.28%	AAR16181.1

Select for downloading or viewing reports	Description	Max score	Total score	Query cover	E value	Ident	Accession
65 Select seq gb|ACZ71929.1|	orf1a polyprotein [SARS coronavirus wtic-MB]	7505	7505	100%	0.0	80.28%	ACZ71929.1
66 Select seq gb|AAR87511.1|	orf1a polyprotein [SARS coronavirus TW11]	7504	7504	100%	0.0	80.28%	AAR87511.1
67 Select seq gb|ACZ71765.1|	orf1a polyprotein [SARS coronavirus wtic-MB]	7504	7504	100%	0.0	80.28%	ACZ71765.1
68 Select seq gb|AEA10756.1|	polyprotein orf1a [SARS coronavirus MA15 ExoN1]	7504	7504	100%	0.0	80.26%	AEA10756.1
69 Select seq gb|AFD56972.1|	orf1a polyprotein [SARS coronavirus HKU-39849]	7504	7504	100%	0.0	80.30%	AFD56972.1
70 Select seq gb|ACZ71780.1|	orf1a polyprotein [SARS coronavirus ExoN1]	7503	7503	100%	0.0	80.26%	ACZ71780.1
71 Select seq gb|ACZ72076.1|	orf1a polyprotein [SARS coronavirus MA15]	7503	7503	100%	0.0	80.24%	ACZ72076.1

Select for downloading or viewing reports	Description	Max score	Total score	Query cover	E value	Ident	Accession
72 Select seq gb|ACZ71914.1|	orf1a polyprotein [SARS coronavirus wtic-MB]	7502	7502	100%	0.0	80.26%	ACZ71914.1
73 Select seq gb|ATO98204.1|	non-structural polyprotein 1a [Bat SARS-like coronavirus]	7500	7500	100%	0.0	80.59%	ATO98204.1
74 Select seq gb|ATO98119.1|	non-structural polyprotein 1a [Bat SARS-like coronavirus]	7498	7498	100%	0.0	80.54%	ATO98119.1
75 Select seq gb|ALK02469.1|	non-structural polyprotein 1a [SARS-like coronavirus WIV16]	7495	7495	100%	0.0	80.57%	ALK02469.1
76 Select seq gb|QDF43834.1|	ORF1ab [Coronavirus BtRs-BetaCoV/YN2018D]	7494	7494	99%	0.0	80.66%	QDF43834.1
77 Select seq gb|ATO98180.1|	non-structural polyprotein 1a [Bat SARS-like coronavirus]	7492	7492	100%	0.0	80.48%	ATO98180.1
78 Select seq gb|ATO98130.1|	non-structural polyprotein 1ab [Bat SARS-like coronavirus]	7491	7491	99%	0.0	80.46%	ATO98130.1

Select for downloading or viewing reports	Description	Max score	Total score	Query cover	E value	Ident	Accession
79 ☐ Select seq gb|ATO98168.1|	non-structural polyprotein 1a [Bat SARS-like coronavirus]	7490	7490	100%	0.0	80.36%	ATO98168.1
80 ☐ Select seq gb|QDF43829.1|	ORF1ab [Coronavirus BtRs-BetaCoV/YN2018C]	7489	7489	99%	0.0	80.57%	QDF43829.1
81 ☐ Select seq gb|ATO98155.1|	non-structural polyprotein 1ab [Bat SARS-like coronavirus]	7484	7484	99%	0.0	80.53%	ATO98155.1
82 ☐ Select seq gb|ATO98229.1|	non-structural polyprotein 1ab [Bat SARS-like coronavirus]	7484	7484	99%	0.0	80.48%	ATO98229.1
83 ☐ Select seq gb|QDF43819.1|	ORF1ab [Coronavirus BtRs-BetaCoV/YN2018A]	7483	7483	99%	0.0	80.44%	QDF43819.1
84 ☐ Select seq gb|AAR14802.1|	putative orf1ab polyprotein [SARS coronavirus PUMC01]	7480	7480	100%	0.0	80.33%	AAR14802.1
85 ☐ Select seq gb|AAS00002.1|	nonstructural polyprotein [SARS coronavirus GZ02]	7479	7479	99%	0.0	80.38%	AAS00002.1

Select for downloading or viewing reports	Description	Max score	Total score	Query cover	E value	Ident	Accession
86<br>Select seq gb|AAY60791.1|	orf1ab polyprotein [SARS coronavirus BJ202]	7477	7477	99%	0.0	80.33%	AAY60791.1
87<br>Select seq gb|AAP94757.1|	putative orf1ab polyprotein [SARS coronavirus CUHK-AG03]	7475	7475	99%	0.0	80.31%	AAP94757.1
88<br>Select seq gb|AAR87587.1|	putative polyprotein [SARS coronavirus TW8]	7475	7475	99%	0.0	80.31%	AAR87587.1
89<br>Select seq ref|NP_828849.2|	orf1ab polyprotein (pp1ab) [Severe acute respiratory syndrome-related coronavirus]	7475	7475	99%	0.0	80.31%	NP_828849.2
90<br>Select seq gb|AAP33696.1|	polyprotein 1ab [SARS coronavirus Frankfurt 1]	7475	7475	99%	0.0	80.31%	AAP33696.1
91<br>Select seq gb|AAY60779.1|	orf1ab polyprotein [SARS coronavirus BJ162]	7474	7474	99%	0.0	80.33%	AAY60779.1
92<br>Select seq gb|ATO98203.1|	non-structural polyprotein 1ab [Bat SARS-like coronavirus]	7467	7467	99%	0.0	80.53%	ATO98203.1

Select for downloading or viewing reports	Description	Max score	Total score	Query cover	E value	Ident	Accession
93 Select seq gb|ATO98118.1|	non-structural polyprotein 1ab [Bat SARS-like coronavirus]	7467	7467	99%	0.0	80.53%	ATO98118.1
94 Select seq gb|ATO98179.1|	non-structural polyprotein 1ab [Bat SARS-like coronavirus]	7461	7461	99%	0.0	80.46%	ATO98179.1
95 Select seq ref|WP_011278100.1|	ADP-ribose-binding protein [Sulfolobus acidocaldarius]	73.2	73.2	2%	1e-12	36.29%	WP_011278100.1
96 Select seq ref|NP_228318.1|	recombination factor protein RarA [Thermotoga maritima MSB8]	76.3	76.3	2%	6e-12	39.84%	NP_228318.1
97 Select seq ref|XP_021327929.1|	poly [ADP-ribose] polymerase 14 isoform X3 [Danio rerio]	63.5	63.5	3%	7e-08	32.26%	XP_021327929.1
98 Select seq ref|XP_021327930.1|	poly [ADP-ribose] polymerase 14-like [Danio rerio]	63.2	63.2	3%	9e-08	31.61%	XP_021327930.1
99 Select seq ref|XP_021327928.1|	poly [ADP-ribose] polymerase 14 isoform X2 [Danio rerio]	62.8	62.8	3%	1e-07	32.26%	XP_021327928.1

Select for downloading or viewing reports	Description	Max score	Total score	Query cover	E value	Ident	Accession
100 Select seq ref|XP_006522818.1|	protein mono-ADP-ribosyltransferase PARP9 isoform X1 [Mus musculus]	62.4	62.4	2%	1e-07	33.80%	XP_006522818.1
101 Select seq ref|NP_084529.1|	protein mono-ADP-ribosyltransferase PARP9 [Mus musculus]	62.4	62.4	2%	1e-07	33.80%	NP_084529.1
102 Select seq ref|XP_017213522.2|	poly [ADP-ribose] polymerase 14 isoform X1 [Danio rerio]	60.5	60.5	3%	5e-07	31.61%	XP_017213522.2
103 Select seq ref|NP_415563.1|	2'-O-acetyl-ADP-ribose deacetylase, regulator of RNase III activity [Escherichia coli str. K-12 substr. MG1655]	55.8	55.8	3%	9e-07	29.07%	NP_415563.1
104 Select seq ref|XP_017213521.2|	poly [ADP-ribose] polymerase 14 isoform X1 [Danio rerio]	59.3	59.3	3%	1e-06	31.41%	XP_017213521.2
105 Select seq ref|XP_011511230.1|	protein mono-ADP-ribosyltransferase PARP14 isoform X1 [Homo sapiens]	57.0	57.0	2%	6e-06	32.26%	XP_011511230.1
106 Select seq ref|XP_011511231.1|	protein mono-ADP-ribosyltransferase PARP14 isoform X2 [Homo sapiens]	57.0	57.0	2%	6e-06	32.26%	XP_011511231.1

Select for downloading or viewing reports	Description	Max score	Total score	Query cover	E value	Ident	Accession
107 Select seq ref|NP_060024.2|	protein mono-ADP-ribosyltransferase PARP14 [Homo sapiens]	56.2	56.2	2%	1e-05	32.26%	NP_060024.2
108 Select seq ref|XP_001340167.2|	poly [ADP-ribose] polymerase 9 [Danio rerio]	55.8	55.8	2%	1e-05	33.08%	XP_001340167.2
109 Select seq ref|XP_021327926.1|	poly [ADP-ribose] polymerase 14 isoform X2 [Danio rerio]	55.8	55.8	2%	1e-05	30.88%	XP_021327926.1
110 Select seq ref|XP_021336679.1|	O-acetyl-ADP-ribose deacetylase MACROD1 isoform X1 [Danio rerio]	50.4	50.4	3%	1e-04	28.57%	XP_021336679.1
111 Select seq ref|NP_001004573.2|	O-acetyl-ADP-ribose deacetylase MACROD1 [Danio rerio]	51.2	51.2	3%	1e-04	28.57%	NP_001004573.2
112 Select seq ref|NP_113646.2|	protein mono-ADP-ribosyltransferase PARP9 isoform a [Homo sapiens]	50.1	50.1	2%	7e-04	30.99%	NP_113646.2
113 Select seq ref|XP_011511520.1|	protein mono-ADP-ribosyltransferase PARP9 isoform X3 [Homo sapiens]	50.1	50.1	2%	7e-04	30.99%	XP_011511520.1

Select for downloading or viewing reports	Description	Max score	Total score	Query cover	E value	Ident	Accession
114 Select seq ref|NP_001139575.1|	protein mono-ADP-ribosyltransferase PARP9 isoform b [Homo sapiens]	49.7	49.7	2%	8e-04	30.99%	NP_001139575.1

Alignments

Loading alignment... for sequences ref|WP_011278100.1|,ref|NP_228318.1| Reading indexes 1-2
Download
FASTA (complete sequence)
FASTA (aligned sequences)
GenBank (complete sequence)
Continue Cancel
GenPeptGraphics Next Previous Descriptions
ADP-ribose-binding protein [Sulfolobus acidocaldarius]
Sequence ID: WP_011278100.1Length: 181Number of Matches: 1
Related Information
Gene-associated gene details
Identical Proteins-Identical proteins to WP_011278100.1
Range 1: 13 to 132GenPeptGraphics Next Match Previous Match First Match

Alignment statistics for match #1

Score	Expect	Method	Identities	Positives	Gaps	Frame
73.2 bits(178)	1e-12()	Composition-based stats.	45/124(36%)	71/124(57%)	10/124(8%)	

Features:

```
Query  1038  VYIKNADIVEEAKKVKPTVVVNAANVYLKHGGGVAGALNKATNNAMQVESDDYIATNGPL  1097
             V ++N DI     KV+    +VNAAN YL HGGGVA A+ ++     +Q ESD+Y+   NGP+
Sbjct  13    VILENGDIT----KVEADAIVNAANSYLSHGGGVALAIVRSGGYIIQEESDEYVRRNGPV  68

Query  1098  KVGGSCVLSGHNL-AKHCLHVVGPNVNKGEDIQL---LKSAYENFNQHEV--LLAPLLSA  1151
              VG    V +    L A++ +H VGP      D +L    ++ + E   ++ ++    +   P +S
```

```
Sbjct  69    PVGEVAVTTAGKLKARYVIHAVGPRYGIEGDDKLESAIRRSLEKADELKLSSIALPAIST  128

Query  1152  GIFG  1155
             GI+G
Sbjct  129   GIYG  132
```

Download

- (•) FASTA (complete sequence)
- ( ) FASTA (aligned sequences)
- ( ) GenBank (complete sequence)

Continue Cancel

GenPeptGraphics Next Previous Descriptions

AAA family ATPase [Thermotoga maritima]

Sequence ID: WP_004081432.1Length: 599Number of Matches: 1

Related Information

Gene-associated gene details

Identical Proteins-Identical proteins to WP_004081432.1

Range 1: 428 to 551GenPeptGraphics Next Match Previous Match First Match

Alignment statistics for match #1

Score	Expect	Method	Identities	Positives	Gaps	Frame
76.3 bits(186)	6e-12()	Compositional matrix adjust.	51/128(40%)	67/128(52%)	13/128(10%)	

Features:

```
Query  1037  NVYIKNADIVEEAKKVKPTVVVNAANVYLKHGGGVAGALNKATNNAMQVESDDYIATNGP  1096
               + I   DI  E    +   +VNAAN YLKHGGGVAGA+ +A  + +Q ESD  +   G
Sbjct  428   KIRIVKGDITRE----EVDAIVNAANEYLKHGGGVAGAIVRAGGSVIQEESDRIVQERGR  483

Query  1097  LKVGGSCVLSGHNL-AKHCLHVVGP---NVNKGEDIQLLKSAYEN-FNQHEVLLA----P  1147
             +   G + V S    L AK+ +H VGP      + GED  L K+ Y      HE+ L      P
Sbjct  484   VPTGEAVVTSAGKLKAKYVIHTVGPVWRGGSHGEDELLYKAVYNALLRAHELKLKSISMP  543

Query  1148  LLSAGIFG  1155
               +S GIFG
Sbjct  544   AISTGIFG  551
```

Loading alignment... for sequences ref|XP_021327929.1|,ref|XP_006522818.1|,ref|NP_415563.1|,gb|QIA20043.1|,gb|QHU79172.1| Reading indexes 3-7

Download

- FASTA (complete sequence)
- FASTA (aligned sequences)
- GenBank (complete sequence)

Continue Cancel

GenPeptGraphics Next Previous Descriptions

poly [ADP-ribose] polymerase 14 isoform X3 [Danio rerio]

Sequence ID: XP_021327929.1Length: 1708Number of Matches: 1

Related Information

Gene-associated gene details

NewGenome Data Viewer-aligned genomic context

Range 1: 786 to 932GenPeptGraphics Next Match Previous Match First Match

Alignment statistics for match #1

Score	Expect	Method	Identities	Positives	Gaps	Frame
63.5 bits(153)	7e-08()	Compositional matrix adjust.	50/155(32%)	76/155(49%)	19/155(12%)	

Features:

```
Query  1038  VYIKNADIVEEAKKVKPTVVVNAANVYLKHGGGVAGALNKATNNAMQVESDDYIATNGPL  1097
             V ++ ADI      +     VVNAAN   LKH GGVA AL +A     +Q    D +I   NGPL
Sbjct  786   VSVRKADIC----TLSVDAVVNAANEDLKHTGGVAYALLQAAGRCLQEYCDLHIKVNGPL  841

Query  1098  KVGGSCVLSGHNLA-KHCLHVVGPN---VNKGEDIQ--LLKSAYENFNQH-----EVLLA  1146
               G  + +      L   K+ +H VGP     +N+    +Q  L ++   E+ NQ         +
Sbjct  842   TPGDAIITDAGRLPCKYVVHAVGPRFSALNQRTGVQQCLRRAVRESLNQASSKKCSSIAI  901

Query  1147  PLLSAGIFGADPIHSLRVCVDTVRTNVYLAVFDKN  1181
             P++S+GIFG      L +C +++    V    + + N
Sbjct  902   PVISSGIFGC----PLDLCTESIAKEVRQYIENHN  932
```

Download

- FASTA (complete sequence)
- FASTA (aligned sequences)
- GenBank (complete sequence)

Continue Cancel

GenPeptGraphics Next Previous Descriptions

protein mono-ADP-ribosyltransferase PARP9 isoform X1 [Mus musculus]

Sequence ID: XP_006522818.1Length: 866Number of Matches: 1

Related Information

Gene-associated gene details

NewGenome Data Viewer-aligned genomic context

Identical Proteins-Identical proteins to XP_006522818.1

Range 1: 137 to 274GenPeptGraphics Next Match Previous Match First Match

Alignment statistics for match #1

Score	Expect	Method	Identities	Positives	Gaps	Frame
62.4 bits(150)	1e-07()	Compositional matrix adjust.	48/142(34%)	64/142(45%)	16/142(11%)	

Features:

```
Query  1057  VVNAANVYLKHGGGVAGALNKATNNAMQVESDDYIATNGPLKVGGSCVLSGHNLAKH-CL  1115
             VVNAAN  L HG G+AG+L K     +Q ES   IA  G + VGG  +     L  H  +
Sbjct  137   VVNAANENLLHGSGLAGSLVKTGGFEIQEESKRIIANVGKISVGGIAITGAGRLPCHLII  196

Query  1116  HVVGPN---VNKGEDIQLLKSAYENFNQH--------EVLLAPLLSAGIFGADPIHSLRV  1164
             H VGP     N    I+LLK A  N    +        + +  P LS+GIF       L +
Sbjct  197   HAVGPRWTVTNSQTAIELLKFAIRNILDYVTKYDLRIKTVAIPALSSGIFQ----FPLDL  252

Query  1165  CVDTVRTNVYLAVFDKNLYDKL  1186
             C    +    + L    DK ++  L
Sbjct  253   CTSIILETIRLYFQDKQMFGNL  274
```

Download

- FASTA (complete sequence)
- FASTA (aligned sequences)
- GenBank (complete sequence)

Continue Cancel

GenPeptGraphics Next Previous Descriptions

MULTISPECIES: O-acetyl-ADP-ribose deacetylase [Proteobacteria]

Sequence ID: WP_000857405.1Length: 177Number of Matches: 1

Related Information

Gene-associated gene details
Identical Proteins-Identical proteins to WP_000857405.1
Range 1: 5 to 172GenPeptGraphics Next Match Previous Match First Match

Alignment statistics for match #1

Score	Expect	Method	Identities	Positives	Gaps	Frame
55.8 bits(133)	9e-07()	Composition-based stats.	50/172(29%)	79/172(45%)	25/172(14%)	

Features:

```
Query  1038  VYIKNADIVEEAKKVKPTVVVNAANVYLKHGGGVAGALNKATNNAMQVESDDYIATNGPL  1097
             +++    DI      K+    V+VNAAN  L   GGGV GA+++A    A+            G
Sbjct  5     IHVVQGDIT----KLAVDVIVNAANPSLMGGGGVDGAIHRAAGPALLDACLKVRQQQGDC  60

Query  1098  KVGGSCV-LSGHNLAKHCLHVVGPNVNKGE--DIQLLKSAYEN------FNQHEVLLAPL  1148
               G + + L+G    AK   +H VGP     GE   + QLL+ AY N        N +  +  P
Sbjct  61    PTGHAVITLAGDLPAKAVVHTVGPVWRGGEQNEDQLLQDAYLNSLRLVAANSYTSVAFPA  120

Query  1149  LSAGIFGADPIHSLRVCVDTVR---------TNVYLAVFDK---NLYDKLVS  1188
             +S G++G      +   + V TV             VY    +D+    +LY++L++
Sbjct  121   ISTGVYGYPRAAAAEIAVKTVSEFITRHALPEQVYFVCYDEENAHLYERLLT  172
```

Download

- (•) FASTA (complete sequence)
- ( ) FASTA (aligned sequences)
- ( ) GenBank (complete sequence)

Continue Cancel

GenPeptGraphics Next Previous Descriptions

orf1ab polyprotein [Severe acute respiratory syndrome coronavirus 2]
Sequence ID: QIA20043.1Length: 4405Number of Matches: 1
Related Information
Range 1: 1 to 4405GenPeptGraphics Next Match Previous Match First Match

Alignment statistics for match #1

Score	Expect	Method	Identities	Positives	Gaps	Frame
9173 bits(23804)	0.0()	Compositional matrix adjust.	4404/4405(99%)	4404/4405(99%)	0/4405(0%)	

Features:

```
Query  1    MESLVPGFNEKTHVQLSLPVLQVRDVLVRGFGDSVEEVLSEARQHLKDGTCGLVEVEKGV  60
            MESLVPGFNEKTHVQLSLPVLQVRDVLVRGFGDSVEEVLSEARQHLKDGTCGLVEVEKGV
Sbjct  1    MESLVPGFNEKTHVQLSLPVLQVRDVLVRGFGDSVEEVLSEARQHLKDGTCGLVEVEKGV  60

Query  61   LPQLEQPYVFIKRSDARTAPHGHVMVELVAELEGIQYGRSGETLGVLVPHVGEIPVAYRK  120
            LPQLEQPYVFIKRSDARTAPHGHVMVELVAELEGIQYGRSGETLGVLVPHVGEIPVAYRK
Sbjct  61   LPQLEQPYVFIKRSDARTAPHGHVMVELVAELEGIQYGRSGETLGVLVPHVGEIPVAYRK  120

Query  121  VLLRKNGNKGAGGHSYGADLKSFDLGDELGTDPYEDFQENWNTKHSSGVTRELMRELNGG  180
            VLLRKNGNKGAGGHSYGADLKSFDLGDELGTDPYEDFQENWNTKHSSGVTRELMRELNGG
Sbjct  121  VLLRKNGNKGAGGHSYGADLKSFDLGDELGTDPYEDFQENWNTKHSSGVTRELMRELNGG  180

Query  181  AYTRYVDNNFCGPDGYPLECIKDLLARAGKASCTLSEQLDFIDTKRGVYCCREHEHEIAW  240
            AYTRYVDNNFCGPDGYPLECIKDLLARAGKASCTLSEQLDFIDTKRGVYCCREHEHEIAW
Sbjct  181  AYTRYVDNNFCGPDGYPLECIKDLLARAGKASCTLSEQLDFIDTKRGVYCCREHEHEIAW  240

Query  241  YTERSEKSYELQTPFEIKLAKKFDTFNGECPNFVFPLNSIIKTIQPRVEKKKLDGFMGRI  300
            YTERSEKSYELQTPFEIKLAKKFDTFNGECPNFVFPLNSIIKTIQPRVEKKKLDGFMGRI
Sbjct  241  YTERSEKSYELQTPFEIKLAKKFDTFNGECPNFVFPLNSIIKTIQPRVEKKKLDGFMGRI  300

Query  301  RSVYPVASPNECNQMCLSTLMKCDHCGETSWQTGDFVKATCEFCGTENLTKEGATTCGYL  360
            RSVYPVASPNECNQMCLSTLMKCDHCGETSWQTGDFVKATCEFCGTENLTKEGATTCGYL
Sbjct  301  RSVYPVASPNECNQMCLSTLMKCDHCGETSWQTGDFVKATCEFCGTENLTKEGATTCGYL  360

Query  361  PQNAVVKIYCPACHNSEVGPEHSLAEYHNESGLKTILRKGGRTIAFGGCVFSYVGCHNKC  420
            PQNAVVKIYCPACHNSEVGPEHSLAEYHNESGLKTILRKGGRTIAFGGCVFSYVGCHNKC
Sbjct  361  PQNAVVKIYCPACHNSEVGPEHSLAEYHNESGLKTILRKGGRTIAFGGCVFSYVGCHNKC  420

Query  421  AYWVPRASANIGCNHTGVVGEGSEGLNDNLLEILQKEKVNINIVGDFKLNEEIAIILASF  480
            AYWVPRASANIGCNHTGVVGEGSEGLNDNLLEILQKEKVNINIVGDFKLNEEIAIILASF
Sbjct  421  AYWVPRASANIGCNHTGVVGEGSEGLNDNLLEILQKEKVNINIVGDFKLNEEIAIILASF  480

Query  481  SASTSAFVETVKGLDYKAFKQIVESCGNFKVTKGKAKKGAWNIGEQKSILSPLYAFASEA  540
            SASTSAFVETVKGLDYKAFKQIVESCGNFKVTKGKAKKGAWNIGEQKSILSPLYAFASEA
Sbjct  481  SASTSAFVETVKGLDYKAFKQIVESCGNFKVTKGKAKKGAWNIGEQKSILSPLYAFASEA  540

Query  541  ARVVRSIFSRTLETAQNSVRVLQKAAITILDGISQYSLRLIDAMMFTSDLATNNLVVMAY  600
            ARVVRSIFSRTLETAQNSVRVLQKAAITILDGISQYSLRLIDAMMFTSDLATNNLVVMAY
Sbjct  541  ARVVRSIFSRTLETAQNSVRVLQKAAITILDGISQYSLRLIDAMMFTSDLATNNLVVMAY  600

Query  601  ITGGVVQLTSQWLTNIFGTVYEKLKPVLDWLEEKFKEGVEFLRDGWEIVKFISTCACEIV  660
            ITGGVVQLTSQWLTNIFGTVYEKLKPVLDWLEEKFKEGVEFLRDGWEIVKFISTCACEIV
Sbjct  601  ITGGVVQLTSQWLTNIFGTVYEKLKPVLDWLEEKFKEGVEFLRDGWEIVKFISTCACEIV  660
```

```
Query  661   GGQIVTCAKEIKESVQTFFKLVNKFLALCADSIIIGGAKLKALNLGETFVTHSKGLYRKC  720
             GGQIVTCAKEIKESVQTFFKLVNKFLALCADSIIIGGAKLKALNLGETFVTHSKGLYRKC
Sbjct  661   GGQIVTCAKEIKESVQTFFKLVNKFLALCADSIIIGGAKLKALNLGETFVTHSKGLYRKC  720

Query  721   VKSREETGLLMPLKAPKEIIFLEGETLPTEVLTEEVVLKTGDLQPLEQPTSEAVEAPLVG  780
             VKSREETGLLMPLKAPKEIIFLEGETLPTEVLTEEVVLKTGDLQPLEQPTSEAVEAPLVG
Sbjct  721   VKSREETGLLMPLKAPKEIIFLEGETLPTEVLTEEVVLKTGDLQPLEQPTSEAVEAPLVG  780

Query  781   TPVCINGLMLLEIKDTEKYCALAPNMMVTNNTFTLKGGAPTKVTFGDDTVIEVQGYKSVN  840
             TPVCINGLMLLEIKDTEKYCALAPNMMVTNNTFTLKGGAPTKVTFGDDTVIEVQGYKSVN
Sbjct  781   TPVCINGLMLLEIKDTEKYCALAPNMMVTNNTFTLKGGAPTKVTFGDDTVIEVQGYKSVN  840

Query  841   ITFELDERIDKVLNEKCSAYTVELGTEVNEFACVVADAVIKTLQPVSELLTPLGIDLDEW  900
             ITFELDERIDKVLNEKCSAYTVELGTEVNEFACVVADAVIKTLQPVSELLTPLGIDLDEW
Sbjct  841   ITFELDERIDKVLNEKCSAYTVELGTEVNEFACVVADAVIKTLQPVSELLTPLGIDLDEW  900

Query  901   SMATYYLFDESGEFKLASHMYCSFYPPDEDEEEGDCEEEEFEPSTQYEYGTEDDYQGKPL  960
             SMATYYLFDESGEFKLASHMYCSFYPPDEDEEEGDCEEEEFEPSTQYEYGTEDDYQGKPL
Sbjct  901   SMATYYLFDESGEFKLASHMYCSFYPPDEDEEEGDCEEEEFEPSTQYEYGTEDDYQGKPL  960

Query  961   EFGATSAALQPEEEQEEDWLDDDSQQTVGQQDGSEDNQTTTIQTIVEVQPQLEMELTPVV  1020
             EFGATSAALQPEEEQEEDWLDDDSQQTVGQQDGSEDNQTTTIQTIVEVQPQLEMELTPVV
Sbjct  961   EFGATSAALQPEEEQEEDWLDDDSQQTVGQQDGSEDNQTTTIQTIVEVQPQLEMELTPVV  1020

Query  1021  QTIEVNSFSGYLKLTDNVYIKNADIVEEAKKVKPTVVVNAANVYLKHGGGVAGALNKATN  1080
             QTIEVNSFSGYLKLTDNVYIKNADIVEEAKKVKPTVVVNAANVYLKHGGGVAGALNKATN
Sbjct  1021  QTIEVNSFSGYLKLTDNVYIKNADIVEEAKKVKPTVVVNAANVYLKHGGGVAGALNKATN  1080

Query  1081  NAMQVESDDYIATNGPLKVGGSCVLSGHNLAKHCLHVVGPNVNKGEDIQLLKSAYENFNQ  1140
             NAMQVESDDYIATNGPLKVGGSCVLSGHNLAKHCLHVVGPNVNKGEDIQLLKSAYENFNQ
Sbjct  1081  NAMQVESDDYIATNGPLKVGGSCVLSGHNLAKHCLHVVGPNVNKGEDIQLLKSAYENFNQ  1140

Query  1141  HEVLLAPLLSAGIFGADPIHSLRVCVDTVRTNVYLAVFDKNLYDKLVSSFLEMKSEKQVE  1200
             HEVLLAPLLSAGIFGADPIHSLRVCVDTVRTNVYLAVFDKNLYDKLVSSFLEMKSEKQVE
Sbjct  1141  HEVLLAPLLSAGIFGADPIHSLRVCVDTVRTNVYLAVFDKNLYDKLVSSFLEMKSEKQVE  1200

Query  1201  QKIAEIPKEEVKPFITESKPSVEQRKQDDKKIKACVEEVTTTLEETKFLTENLLLYIDIN  1260
             QKIAEIPKEEVKPFITESKPSVEQRKQDDKKIKACVEEVTTTLEETKFLTENLLLYIDIN
Sbjct  1201  QKIAEIPKEEVKPFITESKPSVEQRKQDDKKIKACVEEVTTTLEETKFLTENLLLYIDIN  1260

Query  1261  GNLHPDSATLVSDIDITFLKKDAPYIVGDVVQEGVLTAVVIPTKKAGGTTEMLAKALRKV  1320
             GNLHPDSATLVSDIDITFLKKDAPYIVGDVVQEGVLTAVVIPTKKAGGTTEMLAKALRKV
Sbjct  1261  GNLHPDSATLVSDIDITFLKKDAPYIVGDVVQEGVLTAVVIPTKKAGGTTEMLAKALRKV  1320
```

```
Query  1321  PTDNYITTYPGQGLNGYTVEEAKTVLKKCKSAFYILPSIISNEKQEILGTVSWNLREMLA  1380
             PTDNYITTYPGQGLNGYTVEEAKTVLKKCKSAFYILPSIISNEKQEILGTVSWNLREMLA
Sbjct  1321  PTDNYITTYPGQGLNGYTVEEAKTVLKKCKSAFYILPSIISNEKQEILGTVSWNLREMLA  1380

Query  1381  HAEETRKLMPVCVETKAIVSTIQRKYKGIKIQEGVVDYGARFYFYTSKTTVASLINTLND  1440
             HAEETRKLMPVCVETKAIVSTIQRKYKGIKIQEGVVDYGARFYFYTSKTTVASLINTLND
Sbjct  1381  HAEETRKLMPVCVETKAIVSTIQRKYKGIKIQEGVVDYGARFYFYTSKTTVASLINTLND  1440

Query  1441  LNETLVTMPLGYVTHGLNLEEAARYMRSLKVPATVSVSSPDAVTAYNGYLTSSSKTPEEH  1500
             LNETLVTMPLGYVTHGLNLEEAARYMRSLKVPATVSVSSPDAVTAYNGYLTSSSKTPEEH
Sbjct  1441  LNETLVTMPLGYVTHGLNLEEAARYMRSLKVPATVSVSSPDAVTAYNGYLTSSSKTPEEH  1500

Query  1501  FIETISLAGSYKDWSYSGQSTQLGIEFLKRGDKSVYYTSNPTTFHLDGEVITFDNLKTLL  1560
             FIETISLAGSYKDWSYSGQSTQLGIEFLKRGDKSVYYTSNPTTFHLDGEVITFDNLKTLL
Sbjct  1501  FIETISLAGSYKDWSYSGQSTQLGIEFLKRGDKSVYYTSNPTTFHLDGEVITFDNLKTLL  1560

Query  1561  SLREVRTIKVFTTVDNINLHTQVVDMSMTYGQQFGPTYLDGADVTKIKPHNSHEGKTFYV  1620
             SLREVRTIKVFTTVDNINLHTQVVDMSMTYGQQFGPTYLDGADVTKIKPHNSHEGKTFYV
Sbjct  1561  SLREVRTIKVFTTVDNINLHTQVVDMSMTYGQQFGPTYLDGADVTKIKPHNSHEGKTFYV  1620

Query  1621  LPNDDTLRVEAFEYYHTTDPSFLGRYMSALNHTKKWKYPQVNGLTSIKWADNNCYLATAL  1680
             LPNDDTLRVEAFEYYHTTDPSFLGRYMSALNHTKKWKYPQVNGLTSIKWADNNCYLATAL
Sbjct  1621  LPNDDTLRVEAFEYYHTTDPSFLGRYMSALNHTKKWKYPQVNGLTSIKWADNNCYLATAL  1680

Query  1681  LTLQQIELKFNPPALQDAYYRARAGEAANFCALILAYCNKTVGELGDVRETMSYLFQHAN  1740
             LTLQQIELKFNPPALQDAYYRARAGEAANFCALILAYCNKTVGELGDVRETMSYLFQHAN
Sbjct  1681  LTLQQIELKFNPPALQDAYYRARAGEAANFCALILAYCNKTVGELGDVRETMSYLFQHAN  1740

Query  1741  LDSCKRVLNVVCKTCGQQQTTLKGVEAVMYMGTLSYEQFKKGVQIPCTCGKQATKYLVQQ  1800
             LDSCKRVLNVVCKTCGQQQTTLKGVEAVMYMGTLSYEQFKKGVQIPCTCGKQATKYLVQQ
Sbjct  1741  LDSCKRVLNVVCKTCGQQQTTLKGVEAVMYMGTLSYEQFKKGVQIPCTCGKQATKYLVQQ  1800

Query  1801  ESPFVMMSAPPAQYELKHGTFTCASEYTGNYQCGHYKHITSKETLYCIDGALLTKSSEYK  1860
             ESPFVMMSAPPAQYELKHGTFTCASEYTGNYQCGHYKHITSKETLYCIDGALLTKSSEYK
Sbjct  1801  ESPFVMMSAPPAQYELKHGTFTCASEYTGNYQCGHYKHITSKETLYCIDGALLTKSSEYK  1860

Query  1861  GPITDVFYKENSYTTTIKPVTYKLDGVVCTEIDPKLDNYYKKDNSYFTEQPIDLVPNQPY  1920
             GPITDVFYKENSYTTTIKPVTYKLDGVVCTEIDPKLDNYYKKDNSYFTEQPIDLVPNQPY
Sbjct  1861  GPITDVFYKENSYTTTIKPVTYKLDGVVCTEIDPKLDNYYKKDNSYFTEQPIDLVPNQPY  1920

Query  1921  PNASFDNFKFVCDNIKFADDLNQLTGYKKPASRELKVTFFPDLNGDVVAIDYKHYTPSFK  1980
             PNASFDNFKFVCDNIKFADDLNQLTGYKKPASRELKVTFFPDLNGDVVAIDYKHYTPSFK
Sbjct  1921  PNASFDNFKFVCDNIKFADDLNQLTGYKKPASRELKVTFFPDLNGDVVAIDYKHYTPSFK  1980
```

```
Query  1981  KGAKLLHKPIVWHVNNATNKATYKPNTWCIRCLWSTKPVETSNSFDVLKSEDAQGMDNLA  2040
             KGAKLLHKPIVWHVNNATNKATYKPNTWCIRCLWSTKPVETSNSFDVLKSEDAQGMDNLA
Sbjct  1981  KGAKLLHKPIVWHVNNATNKATYKPNTWCIRCLWSTKPVETSNSFDVLKSEDAQGMDNLA  2040

Query  2041  CEDLKPVSEEVVENPTIQKDVLECNVKTTEVVGDIILKPANNSLKITEEVGHTDLMAAYV  2100
             CEDLKPVSEEVVENPTIQKDVLECNVKTTEVVGDIILKPANNSLKITEEVGHTDLMAAYV
Sbjct  2041  CEDLKPVSEEVVENPTIQKDVLECNVKTTEVVGDIILKPANNSLKITEEVGHTDLMAAYV  2100

Query  2101  DNSSLTIKKPNELSRVLGLKTLATHGLAAVNSVPWDTIANYAKPFLNKVVSTTTNIVTRC  2160
             DNSSLTIKKPNELSRVLGLKTLATHGLAAVNSVPWDTIANYAKPFLNKVVSTTTNIVTRC
Sbjct  2101  DNSSLTIKKPNELSRVLGLKTLATHGLAAVNSVPWDTIANYAKPFLNKVVSTTTNIVTRC  2160

Query  2161  LNRVCTNYMPYFFTLLLQLCTFTRSTNSRIKASMPTTIAKNTVKSVGKFCLEASFNYLKS  2220
             LNRVCTNYMPYFFTLLLQLCTFTRSTNSRIKASMPTTIAKNTVKSVGKFCLEASFNYLKS
Sbjct  2161  LNRVCTNYMPYFFTLLLQLCTFTRSTNSRIKASMPTTIAKNTVKSVGKFCLEASFNYLKS  2220

Query  2221  PNFSKLINIIIWFLLLSVCLGSLIYSTAALGVLMSNLGMPSYCTGYREGYLNSTNVTIAT  2280
             PNFSKLINIIIWFLLLSVCLGSLIYSTAALGVLMSNLGMPSYCTGYREGYLNSTNVTIAT
Sbjct  2221  PNFSKLINIIIWFLLLSVCLGSLIYSTAALGVLMSNLGMPSYCTGYREGYLNSTNVTIAT  2280

Query  2281  YCTGSIPCSVCLSGLDSLDTYPSLETIQITISSFKWDLTAFGLVAEWFLAYILFTRFFYV  2340
             YCTGSIPCSVCLSGLDSLDTYPSLETIQITISSFKWDLTAFGLVAEWFLAYILFTRFFYV
Sbjct  2281  YCTGSIPCSVCLSGLDSLDTYPSLETIQITISSFKWDLTAFGLVAEWFLAYILFTRFFYV  2340

Query  2341  LGLAAIMQLFFSYFAVHFISNSWLMWLIINLVQMAPISAMVRMYIFFASFYYVWKSYVHV  2400
             LGLAAIMQLFFSYFAVHFISNSWLMWLIINLVQMAPISAMVRMYIFFASFYYVWKSYVHV
Sbjct  2341  LGLAAIMQLFFSYFAVHFISNSWLMWLIINLVQMAPISAMVRMYIFFASFYYVWKSYVHV  2400

Query  2401  VDGCNSSTCMMCYKRNRATRVECTTIVNGVRRSFYVYANGGKGFCKLHNWNCVNCDTFCA  2460
             VDGCNSSTCMMCYKRNRATRVECTTIVNGVRRSFYVYANGGKGFCKLHNWNCVNCDTFCA
Sbjct  2401  VDGCNSSTCMMCYKRNRATRVECTTIVNGVRRSFYVYANGGKGFCKLHNWNCVNCDTFCA  2460

Query  2461  GSTFISDEVARDLSLQFKRPINPTDQSSYIVDSVTVKNGSIHLYFDKAGQKTYERHSLSH  2520
             GSTFISDEVARDLSLQFKRPINPTDQSSYIVDSVTVKNGSIHLYFDKAGQKTYERHSLSH
Sbjct  2461  GSTFISDEVARDLSLQFKRPINPTDQSSYIVDSVTVKNGSIHLYFDKAGQKTYERHSLSH  2520

Query  2521  FVNLDNLRANNTKGSLPINVIVFDGKSKCEESSAKSASVYYSQLMCQPILLLDQALVSDV  2580
             FVNLDNLRANNTKGSLPINVIVFDGKSKCEESSAKSASVYYSQLMCQPILLLDQALVSDV
Sbjct  2521  FVNLDNLRANNTKGSLPINVIVFDGKSKCEESSAKSASVYYSQLMCQPILLLDQALVSDV  2580

Query  2581  GDSAEVAVKMFDAYVNTFSSTFNVPMEKLKTLVATAEAELAKNVSLDNVLSTFISAARQG  2640
             GDSAEVAVKMFDAYVNTFSSTFNVPMEKLKTLVATAEAELAKNVSLDNVLSTFISAARQG
Sbjct  2581  GDSAEVAVKMFDAYVNTFSSTFNVPMEKLKTLVATAEAELAKNVSLDNVLSTFISAARQG  2640
```

```
Query  2641  FVDSDVETKDVVECLKLSHQSDIEVTGDSCNNYMLTYNKVENMTPRDLGACIDCSARHIN  2700
             FVDSDVETKDVVECLKLSHQSDIEVTGDSCNNYMLTYNKVENMTPRDLGACIDCSARHIN
Sbjct  2641  FVDSDVETKDVVECLKLSHQSDIEVTGDSCNNYMLTYNKVENMTPRDLGACIDCSARHIN  2700

Query  2701  AQVAKSHNIALIWNVKDFMSLSEQLRKQIRSAAKKNNLPFKLTCATTRQVVNVVTTKIAL  2760
             AQVAKSHNIALIWNVKDFMSLSEQLRKQIRSAAKKNNLPFKLTCATTRQVVNVVTTKIAL
Sbjct  2701  AQVAKSHNIALIWNVKDFMSLSEQLRKQIRSAAKKNNLPFKLTCATTRQVVNVVTTKIAL  2760

Query  2761  KGGKIVNNWLKQLIKVTLVFLFVAAIFYLITPVHVMSKHTDFSSEIIGYKAIDGGVTRDI  2820
             KGGKIVNNWLKQLIKVTLVFLFVAAIFYLITPVHVMSKHTDFSSEIIGYKAIDGGVTRDI
Sbjct  2761  KGGKIVNNWLKQLIKVTLVFLFVAAIFYLITPVHVMSKHTDFSSEIIGYKAIDGGVTRDI  2820

Query  2821  ASTDTCFANKHADFDTWFSQRGGSYTNDKACPLIAAVITREVGFVVPGLPGTILRTTNGD  2880
             ASTDTCFANKHADFDTWFSQRGGSYTNDKACPLIAAVITREVGFVVPGLPGTILRTTNGD
Sbjct  2821  ASTDTCFANKHADFDTWFSQRGGSYTNDKACPLIAAVITREVGFVVPGLPGTILRTTNGD  2880

Query  2881  FLHFLPRVFSAVGNICYTPSKLIEYTDFATSACVLAAECTIFKDASGKPVPYCYDTNVLE  2940
             FLHFLPRVFSAVGNICYTPSKLIEYTDFATSACVLAAECTIFKDASGKPVPYCYDTNVLE
Sbjct  2881  FLHFLPRVFSAVGNICYTPSKLIEYTDFATSACVLAAECTIFKDASGKPVPYCYDTNVLE  2940

Query  2941  GSVAYESLRPDTRYVLMDGSIIQFPNTYLEGSVRVVTTFDSEYCRHGTCERSEAGVCVST  3000
             GSVAYESLRPDTRYVLMDGSIIQFPNTYLEGSVRVVTTFDSEYCRHGTCERSEAGVCVST
Sbjct  2941  GSVAYESLRPDTRYVLMDGSIIQFPNTYLEGSVRVVTTFDSEYCRHGTCERSEAGVCVST  3000

Query  3001  SGRWVLNNDYYRSLPGVFCGVDAVNLLTNMFTPLIQPIGALDISASIVAGGIVAIVVTCL  3060
             SGRWVLNNDYYRSLPGVFCGVDAVNLLTNMFTPLIQPIGALDISASIVAGGIVAIVVTCL
Sbjct  3001  SGRWVLNNDYYRSLPGVFCGVDAVNLLTNMFTPLIQPIGALDISASIVAGGIVAIVVTCL  3060

Query  3061  AYYFMRFRRAFGEYSHVVAFNTLLFLMSFTVLCLTPVYSFLPGVYSVIYLYLTFYLTNDV  3120
             AYYFMRFRRAFGEYSHVVAFNTLLFLMSFTVLCLTPVYSFLPGVYSVIYLYLTFYLTNDV
Sbjct  3061  AYYFMRFRRAFGEYSHVVAFNTLLFLMSFTVLCLTPVYSFLPGVYSVIYLYLTFYLTNDV  3120

Query  3121  SFLAHIQWMVMFTPLVPFWITIAYIICISTKHFYWFFSNYLKRRVVFNGVSFSTFEEAAL  3180
             SFLAHIQWMVMFTPLVPFWITIAYIICISTKHFYWFFSNYLKRRVVFNGVSFSTFEEAAL
Sbjct  3121  SFLAHIQWMVMFTPLVPFWITIAYIICISTKHFYWFFSNYLKRRVVFNGVSFSTFEEAAL  3180

Query  3181  CTFLLNKEMYLKLRSDVLLPLTQYNRYLALYNKYKYFSGAMDTTSYREAACCHLAKALND  3240
             CTFLLNKEMYLKLRSDVLLPLTQYNRYLALYNKYKYFSGAMDTTSYREAACCHLAKALND
Sbjct  3181  CTFLLNKEMYLKLRSDVLLPLTQYNRYLALYNKYKYFSGAMDTTSYREAACCHLAKALND  3240

Query  3241  FSNSGSDVLYQPPQTSITSAVLQSGFRKMAFPSGKVEGCMVQVTCGTTTLNGLWLDDVVY  3300
             FSNSGSDVLYQPPQTSITSAVLQSGFRKMAFPSGKVEGCMVQVTCGTTTLNGLWLDDVVY
Sbjct  3241  FSNSGSDVLYQPPQTSITSAVLQSGFRKMAFPSGKVEGCMVQVTCGTTTLNGLWLDDVVY  3300
```

```
Query  3301  CPRHVICTSEDMLNPNYEDLLIRKSNHNFLVQAGNVQLRVIGHSMQNCVLKLKVDTANPK  3360
             CPRHVICTSEDMLNPNYEDLLIRKSNHNFLVQAGNVQLRVIGHSMQNCVLKLKVDTANPK
Sbjct  3301  CPRHVICTSEDMLNPNYEDLLIRKSNHNFLVQAGNVQLRVIGHSMQNCVLKLKVDTANPK  3360

Query  3361  TPKYKFVRIQPGQTFSVLACYNGSPSGVYQCAMRPNFTIKGSFLNGSCGSVGFNIDYDCV  3420
             TPKYKFVRIQPGQTFSVLACYNGSPSGVYQCAMRPNFTIKGSFLNGSCGSVGFNIDYDCV
Sbjct  3361  TPKYKFVRIQPGQTFSVLACYNGSPSGVYQCAMRPNFTIKGSFLNGSCGSVGFNIDYDCV  3420

Query  3421  SFCYMHHMELPTGVHAGTDLEGNFYGPFVDRQTAQAAGTDTTITVNVLAWLYAAVINGDR  3480
             SFCYMHHMELPTGVHAGTDLEGNFYGPFVDRQTAQAAGTDTTITVNVLAWLYAAVINGDR
Sbjct  3421  SFCYMHHMELPTGVHAGTDLEGNFYGPFVDRQTAQAAGTDTTITVNVLAWLYAAVINGDR  3480

Query  3481  WFLNRFTTTLNDFNLVAMKYNYEPLTQDHVDILGPLSAQTGIAVLDMCASLKELLQNGMN  3540
             WFLNRFTTTLNDFNLVAMKYNYEPLTQDHVDILGPLSAQTGIAVLDMCASLKELLQNGMN
Sbjct  3481  WFLNRFTTTLNDFNLVAMKYNYEPLTQDHVDILGPLSAQTGIAVLDMCASLKELLQNGMN  3540

Query  3541  GRTILGSALLEDEFTPFDVVRQCSGVTFQSAVKRTIKGTHHWLLLTILTSLLVLVQSTQW  3600
             GRTILGSALLEDEFTPFDVVRQCSGVTFQSAVKRTIKGTHHWLLLTILTSLLVLVQSTQW
Sbjct  3541  GRTILGSALLEDEFTPFDVVRQCSGVTFQSAVKRTIKGTHHWLLLTILTSLLVLVQSTQW  3600

Query  3601  SLFFFLYENAFLPFAMGIIAMSAFAMMFVKHKHAFLCLFLLPSLATVAYFNMVYMPASWV  3660
             SLFFF YENAFLPFAMGIIAMSAFAMMFVKHKHAFLCLFLLPSLATVAYFNMVYMPASWV
Sbjct  3601  SLFFFFYENAFLPFAMGIIAMSAFAMMFVKHKHAFLCLFLLPSLATVAYFNMVYMPASWV  3660

Query  3661  MRIMTWLDMVDTSLSGFKLKDCVMYASAVVLLILMTARTVYDDGARRVWTLMNVLTLVYK  3720
             MRIMTWLDMVDTSLSGFKLKDCVMYASAVVLLILMTARTVYDDGARRVWTLMNVLTLVYK
Sbjct  3661  MRIMTWLDMVDTSLSGFKLKDCVMYASAVVLLILMTARTVYDDGARRVWTLMNVLTLVYK  3720

Query  3721  VYYGNALDQAISMWALIISVTSNYSGVVTTVMFLARGIVFMCVEYCPIFFITGNTLQCIM  3780
             VYYGNALDQAISMWALIISVTSNYSGVVTTVMFLARGIVFMCVEYCPIFFITGNTLQCIM
Sbjct  3721  VYYGNALDQAISMWALIISVTSNYSGVVTTVMFLARGIVFMCVEYCPIFFITGNTLQCIM  3780

Query  3781  LVYCFLGYFCTCYFGLFCLLNRYFRLTLGVYDYLVSTQEFRYMNSQGLLPPKNSIDAFKL  3840
             LVYCFLGYFCTCYFGLFCLLNRYFRLTLGVYDYLVSTQEFRYMNSQGLLPPKNSIDAFKL
Sbjct  3781  LVYCFLGYFCTCYFGLFCLLNRYFRLTLGVYDYLVSTQEFRYMNSQGLLPPKNSIDAFKL  3840

Query  3841  NIKLLGVGGKPCIKVATVQSKMSDVKCTSVVLLSVLQQLRVESSSKLWAQCVQLHNDILL  3900
             NIKLLGVGGKPCIKVATVQSKMSDVKCTSVVLLSVLQQLRVESSSKLWAQCVQLHNDILL
Sbjct  3841  NIKLLGVGGKPCIKVATVQSKMSDVKCTSVVLLSVLQQLRVESSSKLWAQCVQLHNDILL  3900

Query  3901  AKDTTEAFEKMVSLLSVLLSMQGAVDINKLCEEMLDNRATLQAIASEFSSLPSYAAFATA  3960
             AKDTTEAFEKMVSLLSVLLSMQGAVDINKLCEEMLDNRATLQAIASEFSSLPSYAAFATA
Sbjct  3901  AKDTTEAFEKMVSLLSVLLSMQGAVDINKLCEEMLDNRATLQAIASEFSSLPSYAAFATA  3960
```

```
Query  3961  QEAYEQAVANGDSEVVLKKLKKSLNVAKSEFDRDAAMQRKLEKMADQAMTQMYKQARSED  4020
             QEAYEQAVANGDSEVVLKKLKKSLNVAKSEFDRDAAMQRKLEKMADQAMTQMYKQARSED
Sbjct  3961  QEAYEQAVANGDSEVVLKKLKKSLNVAKSEFDRDAAMQRKLEKMADQAMTQMYKQARSED  4020

Query  4021  KRAKVTSAMQTMLFTMLRKLDNDALNNIINNARDGCVPLNIIPLTTAAKLMVVIPDYNTY  4080
             KRAKVTSAMQTMLFTMLRKLDNDALNNIINNARDGCVPLNIIPLTTAAKLMVVIPDYNTY
Sbjct  4021  KRAKVTSAMQTMLFTMLRKLDNDALNNIINNARDGCVPLNIIPLTTAAKLMVVIPDYNTY  4080

Query  4081  KNTCDGTTFTYASALWEIQQVVDADSKIVQLSEISMDNSPNLAWPLIVTALRANSAVKLQ  4140
             KNTCDGTTFTYASALWEIQQVVDADSKIVQLSEISMDNSPNLAWPLIVTALRANSAVKLQ
Sbjct  4081  KNTCDGTTFTYASALWEIQQVVDADSKIVQLSEISMDNSPNLAWPLIVTALRANSAVKLQ  4140

Query  4141  NNELSPVALRQMSCAAGTTQTACTDDNALAYYNTTKGGRFVLALLSDLQDLKWARFPKSD  4200
             NNELSPVALRQMSCAAGTTQTACTDDNALAYYNTTKGGRFVLALLSDLQDLKWARFPKSD
Sbjct  4141  NNELSPVALRQMSCAAGTTQTACTDDNALAYYNTTKGGRFVLALLSDLQDLKWARFPKSD  4200

Query  4201  GTGTIYTELEPPCRFVTDTPKGPKVKYLYFIKGLNNLNRGMVLGSLAATVRLQAGNATEV  4260
             GTGTIYTELEPPCRFVTDTPKGPKVKYLYFIKGLNNLNRGMVLGSLAATVRLQAGNATEV
Sbjct  4201  GTGTIYTELEPPCRFVTDTPKGPKVKYLYFIKGLNNLNRGMVLGSLAATVRLQAGNATEV  4260

Query  4261  PANSTVLSFCAFAVDAAKAYKDYLASGGQPITNCVKMLCTHTGTGQAITVTPEANMDQES  4320
             PANSTVLSFCAFAVDAAKAYKDYLASGGQPITNCVKMLCTHTGTGQAITVTPEANMDQES
Sbjct  4261  PANSTVLSFCAFAVDAAKAYKDYLASGGQPITNCVKMLCTHTGTGQAITVTPEANMDQES  4320

Query  4321  FGGASCCLYCRCHIDHPNPKGFCDLKGKYVQIPTTCANDPVGFTLKNTVCTVCGMWKGYG  4380
             FGGASCCLYCRCHIDHPNPKGFCDLKGKYVQIPTTCANDPVGFTLKNTVCTVCGMWKGYG
Sbjct  4321  FGGASCCLYCRCHIDHPNPKGFCDLKGKYVQIPTTCANDPVGFTLKNTVCTVCGMWKGYG  4380

Query  4381  CSCDQLREPMLQSADAQSFLNGFAV  4405
             CSCDQLREPMLQSADAQSFLNGFAV
Sbjct  4381  CSCDQLREPMLQSADAQSFLNGFAV  4405
```

Download
- (•) FASTA (complete sequence)
- ( ) FASTA (aligned sequences)
- ( ) GenBank (complete sequence)

Continue Cancel

GenPeptGraphics Next Previous Descriptions

orf1a polyprotein [Severe acute respiratory syndrome coronavirus 2]

Sequence ID: QHU79172.1Length: 4405Number of Matches: 1

Related Information

Range 1: 1 to 4405GenPeptGraphics Next Match Previous Match First Match

Alignment statistics for match #1

Score	Expect	Method	Identities	Positives	Gaps	Frame
9141 bits(23719)	0.0()	Compositional matrix adjust.	4393/4405(99%)	4393/4405(99%)	0/4405(0%)	

Features:

```
Query  1    MESLVPGFNEKTHVQLSLPVLQVRDVLVRGFGDSVEEVLSEARQHLKDGTCGLVEVEKGV  60
            MESLVPGFNEKTHVQLSLPVLQVRDVLVRGFGDSVEEVLSEARQHLKDGTCGLVEVEKGV
Sbjct  1    MESLVPGFNEKTHVQLSLPVLQVRDVLVRGFGDSVEEVLSEARQHLKDGTCGLVEVEKGV  60

Query  61   LPQLEQPYVFIKRSDARTAPHGHVMVELVAELEGIQYGRSGETLGVLVPHVGEIPVAYRK  120
            LPQLEQPYVFIKRSDARTAPHGHVMVELVAELEGIQYGRSGETLGVLVPHVGEIPVAYRK
Sbjct  61   LPQLEQPYVFIKRSDARTAPHGHVMVELVAELEGIQYGRSGETLGVLVPHVGEIPVAYRK  120

Query  121  VLLRKNGNKGAGGHSYGADLKSFDLGDELGTDPYEDFQENWNTKHSSGVTRELMRELNGG  180
            VLLRKNGNKGAGGHSYGADLKSFDLGDELGTDPYEDFQENWNTKHSSGVTRELMRELNGG
Sbjct  121  VLLRKNGNKGAGGHSYGADLKSFDLGDELGTDPYEDFQENWNTKHSSGVTRELMRELNGG  180

Query  181  AYTRYVDNNFCGPDGYPLECIKDLLARAGKASCTLSEQLDFIDTKRGVYCCREHEHEIAW  240
            AYTRYVDNNFCGPDGYPLECIKDLLARAGKASCTLSEQLDFIDTKRGVYCCREHEHEIAW
Sbjct  181  AYTRYVDNNFCGPDGYPLECIKDLLARAGKASCTLSEQLDFIDTKRGVYCCREHEHEIAW  240

Query  241  YTERSEKSYELQTPFEIKLAKKFDTFNGECPNFVFPLNSIIKTIQPRVEKKKLDGFMGRI  300
            YTERSEKSYELQTPFEIKLAKKFDTFNGECPNFVFPLNSIIKTIQPRVEKKKLDGFMGRI
Sbjct  241  YTERSEKSYELQTPFEIKLAKKFDTFNGECPNFVFPLNSIIKTIQPRVEKKKLDGFMGRI  300

Query  301  RSVYPVASPNECNQMCLSTLMKCDHCGETSWQTGDFVKATCEFCGTENLTKEGATTCGYL  360
            RSVYPVASPNECNQMCLSTLMKCDHCGETSWQTGDFVKATCEFCGTENLTKEGATTCGYL
Sbjct  301  RSVYPVASPNECNQMCLSTLMKCDHCGETSWQTGDFVKATCEFCGTENLTKEGATTCGYL  360

Query  361  PQNAVVKIYCPACHNSEVGPEHSLAEYHNESGLKTILRKGGRTIAFGGCVFSYVGCHNKC  420
            PQNAVVKIYCPACHNSEVGPEHSLAEYHNESGLKTILRKGGRTIAFGGCVFSYVGCHNKC
Sbjct  361  PQNAVVKIYCPACHNSEVGPEHSLAEYHNESGLKTILRKGGRTIAFGGCVFSYVGCHNKC  420

Query  421  AYWVPRASANIGCNHTGVVGEGSEGLNDNLLEILQKEKVNINIVGDFKLNEEIAIILASF  480
            AYWVPRASANIGCNHTGVVGEGSEGLNDNLLEILQKEKVNINIVGDFKLNEEIAIILASF
Sbjct  421  AYWVPRASANIGCNHTGVVGEGSEGLNDNLLEILQKEKVNINIVGDFKLNEEIAIILASF  480

Query  481  SASTSAFVETVKGLDYKAFKQIVESCGNFKVTKGKAKKGAWNIGEQKSILSPLYAFASEA  540
            SASTSAFVETVKGLDYKAFKQIVESCGNFKVTKGKAKKGAWNIGEQKSILSPLYAFASEA
Sbjct  481  SASTSAFVETVKGLDYKAFKQIVESCGNFKVTKGKAKKGAWNIGEQKSILSPLYAFASEA  540
```

```
Query  541   ARVVRSIFSRTLETAQNSVRVLQKAAITILDGISQYSLRLIDAMMFTSDLATNNLVVMAY  600
             ARVVRSIFSRTLETAQNSVRVLQKAAITILDGISQYSLRLIDAMMFTSDLATNNLVVMAY
Sbjct  541   ARVVRSIFSRTLETAQNSVRVLQKAAITILDGISQYSLRLIDAMMFTSDLATNNLVVMAY  600

Query  601   ITGGVVQLTSQWLTNIFGTVYEKLKPVLDWLEEKFKEGVEFLRDGWEIVKFISTCACEIV  660
             ITGGVVQLTSQWLTNIFGTVYEKLKPVLDWLEEKFKEGVEFLRDGWEIVKFISTCACEIV
Sbjct  601   ITGGVVQLTSQWLTNIFGTVYEKLKPVLDWLEEKFKEGVEFLRDGWEIVKFISTCACEIV  660

Query  661   GGQIVTCAKEIKESVQTFFKLVNKFLALCADSIIIGGAKLKALNLGETFVTHSKGLYRKC  720
             GGQIVTCAKEIKESVQTFFKLVNKFLALCADSIIIGGAKLKALNLGETFVTHSKGLYRKC
Sbjct  661   GGQIVTCAKEIKESVQTFFKLVNKFLALCADSIIIGGAKLKALNLGETFVTHSKGLYRKC  720

Query  721   VKSREETGLLMPLKAPKEIIFLEGETLPTEVLTEEVVLKTGDLQPLEQPTSEAVEAPLVG  780
             VKSREETGLLMPLKAPKEIIFLEGETLPTEVLTEEVVLKTGDLQPLEQPTSEAVEAPLVG
Sbjct  721   VKSREETGLLMPLKAPKEIIFLEGETLPTEVLTEEVVLKTGDLQPLEQPTSEAVEAPLVG  780

Query  781   TPVCINGLMLLEIKDTEKYCALAPNMMVTNNTFTLKGGAPTKVTFGDDTVIEVQGYKSVN  840
             TPVCINGLMLLEIKDTEKYCALAPNMMVTNNTFTLKGGAPTKVTFGDDTVIEVQGYKSVN
Sbjct  781   TPVCINGLMLLEIKDTEKYCALAPNMMVTNNTFTLKGGAPTKVTFGDDTVIEVQGYKSVN  840

Query  841   ITFELDERIDKVLNEKCSAYTVELGTEVNEFACVVADAVIKTLQPVSELLTPLGIDLDEW  900
             ITFELDERIDKVLNEKCSAYTVELGTEVNEFACVVADAVIKTLQPVSELLTPLGIDLDEW
Sbjct  841   ITFELDERIDKVLNEKCSAYTVELGTEVNEFACVVADAVIKTLQPVSELLTPLGIDLDEW  900

Query  901   SMATYYLFDESGEFKLASHMYCSFYPPDEDEEEGDCEEEEFEPSTQYEYGTEDDYQGKPL  960
             SMATYYLFDESGEFKLASHMYCSFYPPDEDEEEGDCEEEEFEPSTQYEYGTEDDYQGKPL
Sbjct  901   SMATYYLFDESGEFKLASHMYCSFYPPDEDEEEGDCEEEEFEPSTQYEYGTEDDYQGKPL  960

Query  961   EFGATSAALQPEEEQEEDWLDDDSQQTVGQQDGSEDNQTTTIQTIVEVQPQLEMELTPVV  1020
             EFGATSAALQPEEEQEEDWLDDDSQQTVGQQDGSEDNQTTTIQTIVEVQPQLEMELTPVV
Sbjct  961   EFGATSAALQPEEEQEEDWLDDDSQQTVGQQDGSEDNQTTTIQTIVEVQPQLEMELTPVV  1020

Query  1021  QTIEVNSFSGYLKLTDNVYIKNADIVEEAKKVKPTVVVNAANVYLKHGGGVAGALNKATN  1080
             QTIEVNSFSGYLKLTDNVYIKNADIVEEAKKVKPTVVVNAANVYLKHGGGVAGALNKATN
Sbjct  1021  QTIEVNSFSGYLKLTDNVYIKNADIVEEAKKVKPTVVVNAANVYLKHGGGVAGALNKATN  1080

Query  1081  NAMQVESDDYIATNGPLKVGGSCVLSGHNLAKHCLHVVGPNVNKGEDIQLLKSAYENFNQ  1140
             NAMQVESDDYIATNGPLKVGGSCVLSGHNLAKHCLHVVGPNVNKGEDIQLLKSAYENFNQ
Sbjct  1081  NAMQVESDDYIATNGPLKVGGSCVLSGHNLAKHCLHVVGPNVNKGEDIQLLKSAYENFNQ  1140

Query  1141  HEVLLAPLLSAGIFGADPIHSLRVCVDTVRTNVYLAVFDKNLYDKLVSSFLEMKSEKQVE  1200
             HEVLLAPLLSAGIFGADPIHSLRVCVDTVRTNVYLAVFDKNLYDKLVSSFLEMKSEKQVE
Sbjct  1141  HEVLLAPLLSAGIFGADPIHSLRVCVDTVRTNVYLAVFDKNLYDKLVSSFLEMKSEKQVE  1200
```

```
Query  1201  QKIAEIPKEEVKPFITESKPSVEQRKQDDKKIKACVEEVTTTLEETKFLTENLLLYIDIN  1260
             QKIAEIPKEEVKPFITESKPSVEQRKQDDKKIKACVEEVTTTLEETKFLTENLLLYIDIN
Sbjct  1201  QKIAEIPKEEVKPFITESKPSVEQRKQDDKKIKACVEEVTTTLEETKFLTENLLLYIDIN  1260

Query  1261  GNLHPDSATLVSDIDITFLKKDAPYIVGDVVQEGVLTAVVIPTKKAGGTTEMLAKALRKV  1320
             GNLHPDSATLVSDIDITFLKKDAPYIVGDVVQEGVLTAVVIPTKKAGGTTEMLAKALRKV
Sbjct  1261  GNLHPDSATLVSDIDITFLKKDAPYIVGDVVQEGVLTAVVIPTKKAGGTTEMLAKALRKV  1320

Query  1321  PTDNYITTYPGQGLNGYTVEEAKTVLKKCKSAFYILPSIISNEKQEILGTVSWNLREMLA  1380
             PTDNYITTYPGQGLNGYTVEEAKTVLKKCKSAFYILPSIISNEKQEILGTVSWNLREMLA
Sbjct  1321  PTDNYITTYPGQGLNGYTVEEAKTVLKKCKSAFYILPSIISNEKQEILGTVSWNLREMLA  1380

Query  1381  HAEETRKLMPVCVETKAIVSTIQRKYKGIKIQEGVVDYGARFYFYTSKTTVASLINTLND  1440
             HAEETRKLMPVCVETKAIVSTIQRKYKGIKIQEGVVDYGARFYFYTSKTTVASLINTLND
Sbjct  1381  HAEETRKLMPVCVETKAIVSTIQRKYKGIKIQEGVVDYGARFYFYTSKTTVASLINTLND  1440

Query  1441  LNETLVTMPLGYVTHGLNLEEAARYMRSLKVPATVSVSSPDAVTAYNGYLTSSSKTPEEH  1500
             LNETLVTMPLGYVTHGLNLEEAARYMRSLKVPATVSVSSPDAVTAYNGYLTSSSKTPEEH
Sbjct  1441  LNETLVTMPLGYVTHGLNLEEAARYMRSLKVPATVSVSSPDAVTAYNGYLTSSSKTPEEH  1500

Query  1501  FIETISLAGSYKDWSYSGQSTQLGIEFLKRGDKSVYYTSNPTTFHLDGEVITFDNLKTLL  1560
             FIETISLAGSYKDWSYSGQSTQLGIEFLKRGDKSVYYTSNPTTFHLDGEVITFDNLKTLL
Sbjct  1501  FIETISLAGSYKDWSYSGQSTQLGIEFLKRGDKSVYYTSNPTTFHLDGEVITFDNLKTLL  1560

Query  1561  SLREVRTIKVFTTVDNINLHTQVVDMSMTYGQQFGPTYLDGADVTKIKPHNSHEGKTFYV  1620
             SLREVRTIKVFTTVDNINLHTQVVDMSMTYGQQFGPTYLDGADVTKIKPHNSHEGKTFYV
Sbjct  1561  SLREVRTIKVFTTVDNINLHTQVVDMSMTYGQQFGPTYLDGADVTKIKPHNSHEGKTFYV  1620

Query  1621  LPNDDTLRVEAFEYYHTTDPSFLGRYMSALNHTKKWKYPQVNGLTSIKWADNNCYLATAL  1680
             LPNDDTLRVEAFEYYHTTDPSFLGRYMSALNHTKKWKYPQVNGLTSIKWADNNCYLATAL
Sbjct  1621  LPNDDTLRVEAFEYYHTTDPSFLGRYMSALNHTKKWKYPQVNGLTSIKWADNNCYLATAL  1680

Query  1681  LTLQQIELKFNPPALQDAYYRARAGEAANFCALILAYCNKTVGELGDVRETMSYLFQHAN  1740
             LTLQQIELKFNPPALQDAYYRARAGEAANFCALILAYCNKTVGELGDVRETMSYLFQHAN
Sbjct  1681  LTLQQIELKFNPPALQDAYYRARAGEAANFCALILAYCNKTVGELGDVRETMSYLFQHAN  1740

Query  1741  LDSCKRVLNVVCKTCGQQQTTLKGVEAVMYMGTLSYEQFKKGVQIPCTCGKQATKYLVQQ  1800
             LDSCKRVLNVVCKTCGQQQTTLKGVEAVMYMGTLSYEQFKKGVQIPCTCGKQATKYLVQQ
Sbjct  1741  LDSCKRVLNVVCKTCGQQQTTLKGVEAVMYMGTLSYEQFKKGVQIPCTCGKQATKYLVQQ  1800

Query  1801  ESPFVMMSAPPAQYELKHGTFTCASEYTGNYQCGHYKHITSKETLYCIDGALLTKSSEYK  1860
             ESPFVMMSAPPAQYELKHGTFTCASEYTGNYQCGHYKHITSKETLYCIDGALLTKSSEYK
Sbjct  1801  ESPFVMMSAPPAQYELKHGTFTCASEYTGNYQCGHYKHITSKETLYCIDGALLTKSSEYK  1860
```

```
Query  1861  GPITDVFYKENSYTTTIKPVTYKLDGVVCTEIDPKLDNYYKKDNSYFTEQPIDLVPNQPY  1920
             GPITDVFYKENSYTTTIKPVTYKLDGVVCTEIDPKLDNYYKKDNSYFTEQPIDLVPNQPY
Sbjct  1861  GPITDVFYKENSYTTTIKPVTYKLDGVVCTEIDPKLDNYYKKDNSYFTEQPIDLVPNQPY  1920

Query  1921  PNASFDNFKFVCDNIKFADDLNQLTGYKKPASRELKVTFFPDLNGDVVAIDYKHYTPSFK  1980
             PNASFDNFKFVCDNIKFADDLNQLTGYKKPASRELKVTFFPDLNGDVVAIDYKHYTPSFK
Sbjct  1921  PNASFDNFKFVCDNIKFADDLNQLTGYKKPASRELKVTFFPDLNGDVVAIDYKHYTPSFK  1980

Query  1981  KGAKLLHKPIVWHVNNATNKATYKPNTWCIRCLWSTKPVETSNSFDVLKSEDAQGMDNLA  2040
             KGAKLLHKPIVWHVNNATNKATYKPNTWCIRCLWSTKPVETSNSFDVLKSEDAQGMDNLA
Sbjct  1981  KGAKLLHKPIVWHVNNATNKATYKPNTWCIRCLWSTKPVETSNSFDVLKSEDAQGMDNLA  2040

Query  2041  CEDLKPVSEEVVENPTIQKDVLECNVKTTEVVGDIILKPANNSLKITEEVGHTDLMAAYV  2100
             CEDLKPVSEEVVENPTIQKDVLECNVKTTEVVGDIILKPANNSLKITEEVGHTDLMAAYV
Sbjct  2041  CEDLKPVSEEVVENPTIQKDVLECNVKTTEVVGDIILKPANNSLKITEEVGHTDLMAAYV  2100

Query  2101  DNSSLTIKKPNELSRVLGLKTLATHGLAAVNSVPWDTIANYAKPFLNKVVSTTTNIVTRC  2160
             DNSSLTIKKPNELSRVLGLKTLATHGLAAVNSVPWDTIANYAKPFLNKVVSTTTNIVTRC
Sbjct  2101  DNSSLTIKKPNELSRVLGLKTLATHGLAAVNSVPWDTIANYAKPFLNKVVSTTTNIVTRC  2160

Query  2161  LNRVCTNYMPYFFTLLLQLCTFTRSTNSRIKASMPTTIAKNTVKSVGKFCLEASFNYLKS  2220
             LNRVC       FTLLLQLCTFTRSTNSRIKASMPTTIAKNTVKSVGKFCLEASFNYLKS
Sbjct  2161  LNRVCXXXXXXXFTLLLQLCTFTRSTNSRIKASMPTTIAKNTVKSVGKFCLEASFNYLKS  2220

Query  2221  PNFSKLINIIIWFLLLSVCLGSLIYSTAALGVLMSNLGMPSYCTGYREGYLNSTNVTIAT  2280
             PNFSKLINIIIWFLLLSVCLGSLIYSTAALGVLMSNLGMPSYCTGYREGYLNSTNVTIAT
Sbjct  2221  PNFSKLINIIIWFLLLSVCLGSLIYSTAALGVLMSNLGMPSYCTGYREGYLNSTNVTIAT  2280

Query  2281  YCTGSIPCSVCLSGLDSLDTYPSLETIQITISSFKWDLTAFGLVAEWFLAYILFTRFFYV  2340
             YCTGSIPCSVCLSGLDSLDTYPSLETIQITISSFKWDLTAFGLVAEWFLAYILFTRFFYV
Sbjct  2281  YCTGSIPCSVCLSGLDSLDTYPSLETIQITISSFKWDLTAFGLVAEWFLAYILFTRFFYV  2340

Query  2341  LGLAAIMQLFFSYFAVHFISNSWLMWLIINLVQMAPISAMVRMYIFFASFYYVWKSYVHV  2400
             LGLAAIMQLFFSYFAVHFISNSWLMWLI     MAPISAMVRMYIFFASFYYVWKSYVHV
Sbjct  2341  LGLAAIMQLFFSYFAVHFISNSWLMWLIXXXXXMAPISAMVRMYIFFASFYYVWKSYVHV  2400

Query  2401  VDGCNSSTCMMCYKRNRATRVECTTIVNGVRRSFYVYANGGKGFCKLHNWNCVNCDTFCA  2460
             VDGCNSSTCMMCYKRNRATRVECTTIVNGVRRSFYVYANGGKGFCKLHNWNCVNCDTFCA
Sbjct  2401  VDGCNSSTCMMCYKRNRATRVECTTIVNGVRRSFYVYANGGKGFCKLHNWNCVNCDTFCA  2460

Query  2461  GSTFISDEVARDLSLQFKRPINPTDQSSYIVDSVTVKNGSIHLYFDKAGQKTYERHSLSH  2520
             GSTFISDEVARDLSLQFKRPINPTDQSSYIVDSVTVKNGSIHLYFDKAGQKTYERHSLSH
Sbjct  2461  GSTFISDEVARDLSLQFKRPINPTDQSSYIVDSVTVKNGSIHLYFDKAGQKTYERHSLSH  2520
```

```
Query  2521  FVNLDNLRANNTKGSLPINVIVFDGKSKCEESSAKSASVYYSQLMCQPILLLDQALVSDV  2580
             FVNLDNLRANNTKGSLPINVIVFDGKSKCEESSAKSASVYYSQLMCQPILLLDQALVSDV
Sbjct  2521  FVNLDNLRANNTKGSLPINVIVFDGKSKCEESSAKSASVYYSQLMCQPILLLDQALVSDV  2580

Query  2581  GDSAEVAVKMFDAYVNTFSSTFNVPMEKLKTLVATAEAELAKNVSLDNVLSTFISAARQG  2640
             GDSAEVAVKMFDAYVNTFSSTFNVPMEKLKTLVATAEAELAKNVSLDNVLSTFISAARQG
Sbjct  2581  GDSAEVAVKMFDAYVNTFSSTFNVPMEKLKTLVATAEAELAKNVSLDNVLSTFISAARQG  2640

Query  2641  FVDSDVETKDVVECLKLSHQSDIEVTGDSCNNYMLTYNKVENMTPRDLGACIDCSARHIN  2700
             FVDSDVETKDVVECLKLSHQSDIEVTGDSCNNYMLTYNKVENMTPRDLGACIDCSARHIN
Sbjct  2641  FVDSDVETKDVVECLKLSHQSDIEVTGDSCNNYMLTYNKVENMTPRDLGACIDCSARHIN  2700

Query  2701  AQVAKSHNIALIWNVKDFMSLSEQLRKQIRSAAKKNNLPFKLTCATTRQVVNVVTTKIAL  2760
             AQVAKSHNIALIWNVKDFMSLSEQLRKQIRSAAKKNNLPFKLTCATTRQVVNVVTTKIAL
Sbjct  2701  AQVAKSHNIALIWNVKDFMSLSEQLRKQIRSAAKKNNLPFKLTCATTRQVVNVVTTKIAL  2760

Query  2761  KGGKIVNNWLKQLIKVTLVFLFVAAIFYLITPVHVMSKHTDFSSEIIGYKAIDGGVTRDI  2820
             KGGKIVNNWLKQLIKVTLVFLFVAAIFYLITPVHVMSKHTDFSSEIIGYKAIDGGVTRDI
Sbjct  2761  KGGKIVNNWLKQLIKVTLVFLFVAAIFYLITPVHVMSKHTDFSSEIIGYKAIDGGVTRDI  2820

Query  2821  ASTDTCFANKHADFDTWFSQRGGSYTNDKACPLIAAVITREVGFVVPGLPGTILRTTNGD  2880
             ASTDTCFANKHADFDTWFSQRGGSYTNDKACPLIAAVITREVGFVVPGLPGTILRTTNGD
Sbjct  2821  ASTDTCFANKHADFDTWFSQRGGSYTNDKACPLIAAVITREVGFVVPGLPGTILRTTNGD  2880

Query  2881  FLHFLPRVFSAVGNICYTPSKLIEYTDFATSACVLAAECTIFKDASGKPVPYCYDTNVLE  2940
             FLHFLPRVFSAVGNICYTPSKLIEYTDFATSACVLAAECTIFKDASGKPVPYCYDTNVLE
Sbjct  2881  FLHFLPRVFSAVGNICYTPSKLIEYTDFATSACVLAAECTIFKDASGKPVPYCYDTNVLE  2940

Query  2941  GSVAYESLRPDTRYVLMDGSIIQFPNTYLEGSVRVVTTFDSEYCRHGTCERSEAGVCVST  3000
             GSVAYESLRPDTRYVLMDGSIIQFPNTYLEGSVRVVTTFDSEYCRHGTCERSEAGVCVST
Sbjct  2941  GSVAYESLRPDTRYVLMDGSIIQFPNTYLEGSVRVVTTFDSEYCRHGTCERSEAGVCVST  3000

Query  3001  SGRWVLNNDYYRSLPGVFCGVDAVNLLTNMFTPLIQPIGALDISASIVAGGIVAIVVTCL  3060
             SGRWVLNNDYYRSLPGVFCGVDAVNLLTNMFTPLIQPIGALDISASIVAGGIVAIVVTCL
Sbjct  3001  SGRWVLNNDYYRSLPGVFCGVDAVNLLTNMFTPLIQPIGALDISASIVAGGIVAIVVTCL  3060

Query  3061  AYYFMRFRRAFGEYSHVVAFNTLLFLMSFTVLCLTPVYSFLPGVYSVIYLYLTFYLTNDV  3120
             AYYFMRFRRAFGEYSHVVAFNTLLFLMSFTVLCLTPVYSFLPGVYSVIYLYLTFYLTNDV
Sbjct  3061  AYYFMRFRRAFGEYSHVVAFNTLLFLMSFTVLCLTPVYSFLPGVYSVIYLYLTFYLTNDV  3120

Query  3121  SFLAHIQWMVMFTPLVPFWITIAYIICISTKHFYWFFSNYLKRRVVFNGVSFSTFEEAAL  3180
             SFLAHIQWMVMFTPLVPFWITIAYIICISTKHFYWFFSNYLKRRVVFNGVSFSTFEEAAL
Sbjct  3121  SFLAHIQWMVMFTPLVPFWITIAYIICISTKHFYWFFSNYLKRRVVFNGVSFSTFEEAAL  3180
```

```
Query  3181  CTFLLNKEMYLKLRSDVLLPLTQYNRYLALYNKYKYFSGAMDTTSYREAACCHLAKALND  3240
             CTFLLNKEMYLKLRSDVLLPLTQYNRYLALYNKYKYFSGAMDTTSYREAACCHLAKALND
Sbjct  3181  CTFLLNKEMYLKLRSDVLLPLTQYNRYLALYNKYKYFSGAMDTTSYREAACCHLAKALND  3240

Query  3241  FSNSGSDVLYQPPQTSITSAVLQSGFRKMAFPSGKVEGCMVQVTCGTTTLNGLWLDDVVY  3300
             FSNSGSDVLYQPPQTSITSAVLQSGFRKMAFPSGKVEGCMVQVTCGTTTLNGLWLDDVVY
Sbjct  3241  FSNSGSDVLYQPPQTSITSAVLQSGFRKMAFPSGKVEGCMVQVTCGTTTLNGLWLDDVVY  3300

Query  3301  CPRHVICTSEDMLNPNYEDLLIRKSNHNFLVQAGNVQLRVIGHSMQNCVLKLKVDTANPK  3360
             CPRHVICTSEDMLNPNYEDLLIRKSNHNFLVQAGNVQLRVIGHSMQNCVLKLKVDTANPK
Sbjct  3301  CPRHVICTSEDMLNPNYEDLLIRKSNHNFLVQAGNVQLRVIGHSMQNCVLKLKVDTANPK  3360

Query  3361  TPKYKFVRIQPGQTFSVLACYNGSPSGVYQCAMRPNFTIKGSFLNGSCGSVGFNIDYDCV  3420
             TPKYKFVRIQPGQTFSVLACYNGSPSGVYQCAMRPNFTIKGSFLNGSCGSVGFNIDYDCV
Sbjct  3361  TPKYKFVRIQPGQTFSVLACYNGSPSGVYQCAMRPNFTIKGSFLNGSCGSVGFNIDYDCV  3420

Query  3421  SFCYMHHMELPTGVHAGTDLEGNFYGPFVDRQTAQAAGTDTTITVNVLAWLYAAVINGDR  3480
             SFCYMHHMELPTGVHAGTDLEGNFYGPFVDRQTAQAAGTDTTITVNVLAWLYAAVINGDR
Sbjct  3421  SFCYMHHMELPTGVHAGTDLEGNFYGPFVDRQTAQAAGTDTTITVNVLAWLYAAVINGDR  3480

Query  3481  WFLNRFTTTLNDFNLVAMKYNYEPLTQDHVDILGPLSAQTGIAVLDMCASLKELLQNGMN  3540
             WFLNRFTTTLNDFNLVAMKYNYEPLTQDHVDILGPLSAQTGIAVLDMCASLKELLQNGMN
Sbjct  3481  WFLNRFTTTLNDFNLVAMKYNYEPLTQDHVDILGPLSAQTGIAVLDMCASLKELLQNGMN  3540

Query  3541  GRTILGSALLEDEFTPFDVVRQCSGVTFQSAVKRTIKGTHHWLLLTILTSLLVLVQSTQW  3600
             GRTILGSALLEDEFTPFDVVRQCSGVTFQSAVKRTIKGTHHWLLLTILTSLLVLVQSTQW
Sbjct  3541  GRTILGSALLEDEFTPFDVVRQCSGVTFQSAVKRTIKGTHHWLLLTILTSLLVLVQSTQW  3600

Query  3601  SLFFFLYENAFLPFAMGIIAMSAFAMMFVKHKHAFLCLFLLPSLATVAYFNMVYMPASWV  3660
             SLFFFLYENAFLPFAMGIIAMSAFAMMFVKHKHAFLCLFLLPSLATVAYFNMVYMPASWV
Sbjct  3601  SLFFFLYENAFLPFAMGIIAMSAFAMMFVKHKHAFLCLFLLPSLATVAYFNMVYMPASWV  3660

Query  3661  MRIMTWLDMVDTSLSGFKLKDCVMYASAVVLLILMTARTVYDDGARRVWTLMNVLTLVYK  3720
             MRIMTWLDMVDTSLSGFKLKDCVMYASAVVLLILMTARTVYDDGARRVWTLMNVLTLVYK
Sbjct  3661  MRIMTWLDMVDTSLSGFKLKDCVMYASAVVLLILMTARTVYDDGARRVWTLMNVLTLVYK  3720

Query  3721  VYYGNALDQAISMWALIISVTSNYSGVVTTVMFLARGIVFMCVEYCPIFFITGNTLQCIM  3780
             VYYGNALDQAISMWALIISVTSNYSGVVTTVMFLARGIVFMCVEYCPIFFITGNTLQCIM
Sbjct  3721  VYYGNALDQAISMWALIISVTSNYSGVVTTVMFLARGIVFMCVEYCPIFFITGNTLQCIM  3780

Query  3781  LVYCFLGYFCTCYFGLFCLLNRYFRLTLGVYDYLVSTQEFRYMNSQGLLPPKNSIDAFKL  3840
             LVYCFLGYFCTCYFGLFCLLNRYFRLTLGVYDYLVSTQEFRYMNSQGLLPPKNSIDAFKL
Sbjct  3781  LVYCFLGYFCTCYFGLFCLLNRYFRLTLGVYDYLVSTQEFRYMNSQGLLPPKNSIDAFKL  3840
```

```
Query  3841  NIKLLGVGGKPCIKVATVQSKMSDVKCTSVVLLSVLQQLRVESSSKLWAQCVQLHNDILL  3900
             NIKLLGVGGKPCIKVATVQSKMSDVKCTSVVLLSVLQQLRVESSSKLWAQCVQLHNDILL
Sbjct  3841  NIKLLGVGGKPCIKVATVQSKMSDVKCTSVVLLSVLQQLRVESSSKLWAQCVQLHNDILL  3900

Query  3901  AKDTTEAFEKMVSLLSVLLSMQGAVDINKLCEEMLDNRATLQAIASEFSSLPSYAAFATA  3960
             AKDTTEAFEKMVSLLSVLLSMQGAVDINKLCEEMLDNRATLQAIASEFSSLPSYAAFATA
Sbjct  3901  AKDTTEAFEKMVSLLSVLLSMQGAVDINKLCEEMLDNRATLQAIASEFSSLPSYAAFATA  3960

Query  3961  QEAYEQAVANGDSEVVLKKLKKSLNVAKSEFDRDAAMQRKLEKMADQAMTQMYKQARSED  4020
             QEAYEQAVANGDSEVVLKKLKKSLNVAKSEFDRDAAMQRKLEKMADQAMTQMYKQARSED
Sbjct  3961  QEAYEQAVANGDSEVVLKKLKKSLNVAKSEFDRDAAMQRKLEKMADQAMTQMYKQARSED  4020

Query  4021  KRAKVTSAMQTMLFTMLRKLDNDALNNIINNARDGCVPLNIIPLTTAAKLMVVIPDYNTY  4080
             KRAKVTSAMQTMLFTMLRKLDNDALNNIINNARDGCVPLNIIPLTTAAKLMVVIPDYNTY
Sbjct  4021  KRAKVTSAMQTMLFTMLRKLDNDALNNIINNARDGCVPLNIIPLTTAAKLMVVIPDYNTY  4080

Query  4081  KNTCDGTTFTYASALWEIQQVVDADSKIVQLSEISMDNSPNLAWPLIVTALRANSAVKLQ  4140
             KNTCDGTTFTYASALWEIQQVVDADSKIVQLSEISMDNSPNLAWPLIVTALRANSAVKLQ
Sbjct  4081  KNTCDGTTFTYASALWEIQQVVDADSKIVQLSEISMDNSPNLAWPLIVTALRANSAVKLQ  4140

Query  4141  NNELSPVALRQMSCAAGTTQTACTDDNALAYYNTTKGGRFVLALLSDLQDLKWARFPKSD  4200
             NNELSPVALRQMSCAAGTTQTACTDDNALAYYNTTKGGRFVLALLSDLQDLKWARFPKSD
Sbjct  4141  NNELSPVALRQMSCAAGTTQTACTDDNALAYYNTTKGGRFVLALLSDLQDLKWARFPKSD  4200

Query  4201  GTGTIYTELEPPCRFVTDTPKGPKVKYLYFIKGLNNLNRGMVLGSLAATVRLQAGNATEV  4260
             GTGTIYTELEPPCRFVTDTPKGPKVKYLYFIKGLNNLNRGMVLGSLAATVRLQAGNATEV
Sbjct  4201  GTGTIYTELEPPCRFVTDTPKGPKVKYLYFIKGLNNLNRGMVLGSLAATVRLQAGNATEV  4260

Query  4261  PANSTVLSFCAFAVDAAKAYKDYLASGGQPITNCVKMLCTHTGTGQAITVTPEANMDQES  4320
             PANSTVLSFCAFAVDAAKAYKDYLASGGQPITNCVKMLCTHTGTGQAITVTPEANMDQES
Sbjct  4261  PANSTVLSFCAFAVDAAKAYKDYLASGGQPITNCVKMLCTHTGTGQAITVTPEANMDQES  4320

Query  4321  FGGASCCLYCRCHIDHPNPKGFCDLKGKYVQIPTTCANDPVGFTLKNTVCTVCGMWKGYG  4380
             FGGASCCLYCRCHIDHPNPKGFCDLKGKYVQIPTTCANDPVGFTLKNTVCTVCGMWKGYG
Sbjct  4321  FGGASCCLYCRCHIDHPNPKGFCDLKGKYVQIPTTCANDPVGFTLKNTVCTVCGMWKGYG  4380

Query  4381  CSCDQLREPMLQSADAQSFLNGFAV  4405
             CSCDQLREPMLQSADAQSFLNGFAV
Sbjct  4381  CSCDQLREPMLQSADAQSFLNGFAV  4405
```

BLAST is a registered trademark of the National Library of Medicine

Support center Mailing list YouTube

- National Library Of Medicine
- National Institutes Of Health
- U.S. Department of Health & Human Services
- USA.gov

NCBI

National Center for Biotechnology Information, U.S. National Library of Medicine 8600 Rockville Pike, Bethesda MD, 20894 USA
Policies and Guidelines | Contact
